# Phosphine Oxide Indenoquinoline Derivatives: Synthesis and Biological Evaluation as Topoisomerase I Inhibitors and Antiproliferative Agents

**DOI:** 10.3390/molecules29245992

**Published:** 2024-12-19

**Authors:** Alba Rodriguez-Paniagua, Cinzia Tesauro, Birgitta R. Knudsen, Maria Fuertes, Concepción Alonso

**Affiliations:** 1 Departamento de Química Orgánica I, Facultad de Farmacia and Centro de Investigación Lascaray (Lascaray Research Center), Universidad del País Vasco/Euskal Herriko Unibertsitatea (UPV/EHU), Paseo de la Universidad 7, 01006 Vitoria-Gasteiz, Spain; alba.rodriguezp@ehu.eus; 2 Department of Molecular Biology and Genetics and Interdisciplinary Nanoscience Center (iNANO), Aarhus University, 8000 Aarhus, Denmark; ctesauro@mbg.au.dk (C.T.); brk@mbg.au.dk (B.R.K.)

**Keywords:** indenoquinolines, phosphine oxide, topoisomerase I, enzyme inhibition, antiproliferative agents

## Abstract

The synthesis of phosphorous indenoquinolines and their biological evaluation as topoisomerase 1 (TOP1) inhibitors and antiproliferative agents were performed. First, the preparation of new hybrid 5*H*-indeno[2,1-*c*]quinolines with a phosphine oxide group was performed by a two-step Povarov-type [4+2]-cycloaddition reaction between the corresponding phosphorated aldimines with indene in the presence of BF_3_·Et_2_O. Subsequent oxidation of the methylene present in the structure resulted in the corresponding indeno[2,1-*c*]quinolin-7-one phosphine oxides **10**. The synthesized derivatives were evaluated as TOP1 inhibitors showing higher inhibition values than CPT at prolonged incubation times (5 min). Inhibition of TOP1 was even observed after 30 min of incubation. The cytotoxic activities of these compounds were also studied against different cancer cell lines and a non-cancerous cell line. While some compounds showed cytotoxicity against some cancerous cells, none of the compounds showed any cytotoxicity against the non-cancerous cell line, MRC-5, in contrast to CPT, which exhibits high toxicity against this cell line. These results represent a very interesting advance since the heterocyclic phosphine oxide derivatives have important properties as TOP1 inhibitors and show an interesting cytotoxicity against different cell lines.

## 1. Introduction

**Figure 1:**
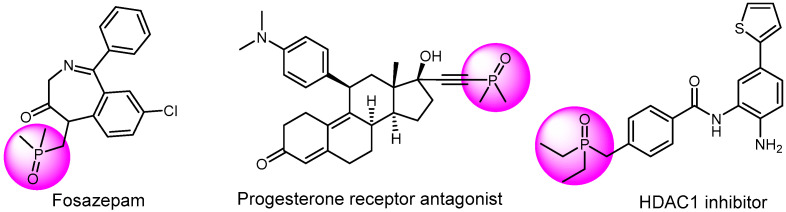
Some phosphine oxide derivatives with medicinal applications.

Phosphorus belongs to one of the most essential elements of life and is extensively distributed in nature. Furthermore, phosphorus-containing drugs constitute an important class of therapeutic agents targeting a wide range of diseases [1,2,3]. Indeed, organophosphorus compounds have several applications in agriculture [4,5], veterinary science [6], and medicine, as therapeutic agents targeting a wide range of diseases or as prodrugs to achieve higher selectivity and bioavailability [1]. Most of these organophosphorus compounds have phosphonate, phosphamide, or phosphate groups, whereas the phosphine oxides have been largely neglected in medicinal chemistry [7,8,9]. Compounds containing a pentavalent phosphorus atom binding directly to one or more carbon atoms feature different structure characteristics from phosphates, usually rendering a more hydrophilic surface and having better chemical stability; in addition, the phosphine oxide, from an electronic point of view, features a very strong H-bond acceptor in a tetrahedral center with three possible derivatization points which would give the molecule increased solubility and metabolic stability [10], such as fosazepam (Figure 1), a water-soluble derivative of diazepam, which is substituted with a dimethylphosphoryl group to improve its solubility in water [11].

Taking this into account, in recent years, some compounds with phosphine oxide in their structure have been described in medicinal chemistry (Figure 1), as progesterone receptor antagonists [12] or enzyme inhibitors (HDAC1) [13].

**Figure 2:**
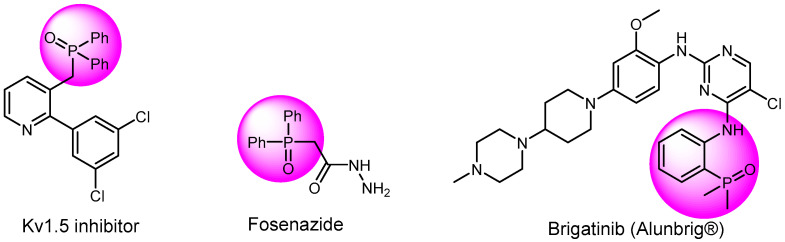
Structure of some phosphine oxide drugs.

Some compounds with a diphenylphosphoryl group are described as phosphine oxide drugs containing three P-C bonds (Figure 2) such as a Kv1.5 channel inhibitor [14] or fosenazide, a neurodrug with strong inhibiting activities of adrenaline and serotonin [15]. On the other hand, the development of anticancer drugs permitted the preparation of Brigatinib (Alunbrig^®^). This drug was approved by the Food and Drug Administration (FDA) as an anticancer drug, which acts as an anaplastic lymphoma kinase and epithelial growth factor receptor inhibitor, and it is also used in targeted cancer therapy [16].

Among the antiproliferative compounds studied for cancer therapy, it is worth highlighting the inhibitors of TOP1, an overexpressed target enzyme in tumor cells. This nuclear enzyme reduces superhelical stress as well as other topological problems generated upon the separation of DNA strands in metabolic processes such as replication, transcription, and recombination [17]. Human TOP1 is the target of several poisons such as camptothecin (CPT) and its derivatives, such as topotecan or irinotecan, which are used to treat a variety of human cancers, including colon, lung, ovarian, and small-cell lung cancer [18,19,20,21]. However, these derivatives show some drawbacks, such as lactone ring instability in physiological medium and cancer cell resistance [22].

These compounds present fused nitrogen heterocycles in their structure, which seems to have relevant importance in their antiproliferative activity, possibly due to the π-π stacking interaction with the DNA base pairs [23]. Normally, the preparation of nitrogenous polycyclic compounds involves a large number of steps, so the hetero-Diels–Alder reaction, a high-atom economic tool, seems a good alternative for the preparation of these derivatives [24,25,26]. In fact, among them, the Povarov reaction may represent a direct route for the preparation of quinoline derivatives with excellent regioselectivity [27,28,29,30].

It is noteworthy that quinoline-containing compounds play an important role in bioorganic and medicinal chemistry [31,32,33]. This structural scaffold is present in many agents of pharmaceutical interest and exhibits a wide range of biological activities, such as being anti-inflammatory [34], neurotropic [35], antibiotic [36,37,38], antipsychotic [39,40], antitubercular [36,41], estrogenic receptors [42,43], and anti-oxidant [34,44], among others. Moreover, several studies have reported the cytotoxic activity of quinoline derivatives on lymphomas, liver, breast, and lung cancer cells [45].

Given the importance of quinoline’s core structure in drug discovery and also its high impact on the development of anticancer therapeutic agents [46], polycyclic quinoline structure is a privileged core used as a structure-based drug design to improve the biological activities of compounds [47]. In fact, CPT, a known TOP1 inhibitor, belongs to the class of quinoline alkaloids and it is precisely its quinoline structure fused to other heterocycles that impedes enzymatic activity [18,19,20,21].

**Scheme 1:**
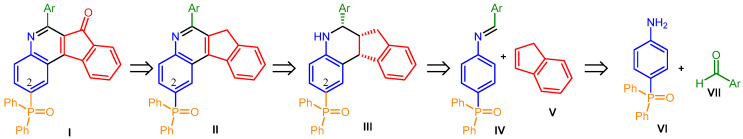
Retrosynthetic pathway.

With all this in mind, in this work, we prepare new indenoquinoline **I**–**III** derivatives (Scheme 1) with a phosphine oxide substituent in position 2, since, as mentioned above, the important TOP1 inhibitor CPT presents quinolines fused to other heterocycles in the structure.

Heterocycles **III** could be obtained using a Povarov-type reaction between the corresponding phosphorated aldimines **IV**, obtained in situ by the reaction of phosphorated aniline **VI** and aromatic aldehydes **VII**, with indene **V**. In addition, the oxidation of indenoquinoline derivatives **III** would afford compounds **I** and **II** with a more planar structure. Following their synthetic preparation, their biological evaluation as TOP1 inhibitors and as antiproliferative agents against different cancerous cell lines was studied.

## 2. Results

### 2.1. Chemistry

**Scheme 2:**
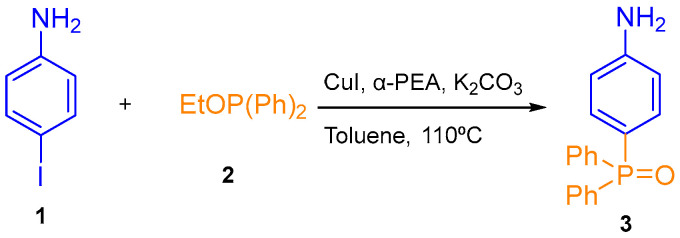
Synthesis of (4-aminophenyl)diphenylphosphine oxide **3**.

We first started with the preparation of the non-commercial (4-aminophenyl)diphenylphosphine oxide **3** (Scheme 2). The preparation of the starting material was finally accomplished efficiently by coupling ethoxydiphenylphosphane **2** with *para*-iodoaniline **1** in the presence of copper(I)iodide as a catalyst and α-phenylethylamine as a ligand [48].

**Scheme 3:**
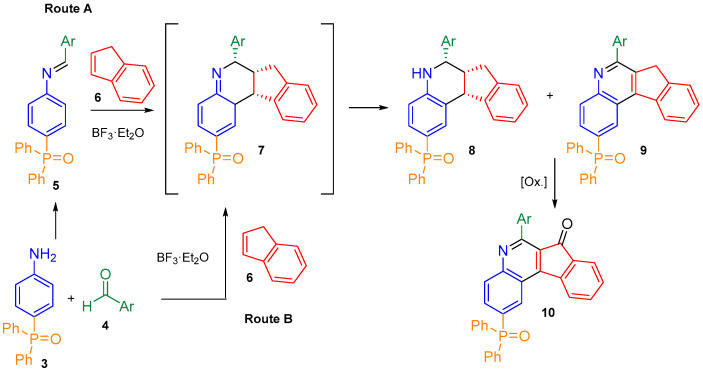
Synthesis of diphenyl(tetrahydro-5*H*-indeno[2,1-*c*]quinolin-2-yl)phosphine oxides **8**, 7*H*-indeno[2,1-*c*]quinolin-2-ylphosphine oxides **9**, and 7-oxo-7*H*-indeno[2,1-*c*]quinolinylphosphine oxides **10**.

Then, once the starting phosphorylated aniline **3** was prepared, it was reacted with different aromatic aldehydes **4** with both electron-withdrawing and -donor substituents, such as fluorine, bromine, and methoxy groups. The condensation between the *para*-phosphorylated aniline **3** and the aromatic aldehydes **4** resulted in the corresponding aldimines **5** (Scheme 3, route A). These compounds **5**, unstable by recrystallization or column chromatography, were used in situ in the next steps.

**Chart 1:**
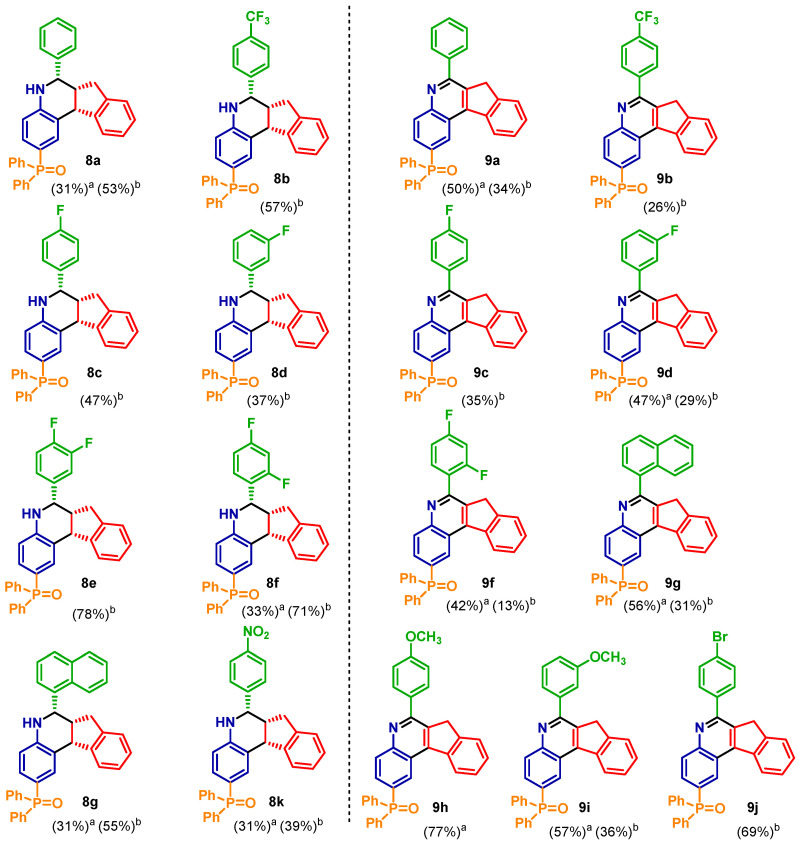
Structures of diphenyl(tetrahydro-5*H*-indeno[2,1-*c*]quinolin-2-yl)phosphine oxides **8** and diphenyl(7*H*-indeno[2,1-*c*]quinolin-2-yl)phosphine oxides **9**. ^a^ Isolated yield obtained by stepwise route (Route A). ^b^ Isolated yield obtained by multicomponent route (Route B).

Aldimines **5** reacted in situ with indene **6** in the presence of two equivalents of a Lewis acid, BF_3_·Et_2_O, in refluxing chloroform, to produce a mixture of the corresponding diphenyl *endo*-tetrahydro-5*H*-indeno[2,1-*c*]quinolin-2-ylphosphine oxides **8** and diphenyl(7*H*-indeno[2,1-*c*]quinolin-2-yl)phosphine oxides **9** (Scheme 3), in a regio- and stereoselective way (Chart 1). The formation of these derivatives could be explained by a [4+2] cycloaddition reaction resulting in derivative **7** followed by a prototropic tautomerization to produce compound **8**, and, finally, compound **9** after the loss of hydrogen.

These compounds **8** and **9** could be prepared by a multicomponent (MCR) protocol (Scheme 3, route B). Thus, the three components (phosphine oxide aniline **3**, aromatic aldehydes **4**, and indene **6**) were reacted in the presence of the Lewis acid (BF_3_·Et_2_O) resulting in the expected derivatives (Chart 1).

It should be noted that the proportion of compounds **8** and **9** obtained differed according to the methodology used, either the stepwise or multicomponent method (Table 1). In general, mixtures of both compounds **8** and **9** were obtained, although when the reaction was carried out in a stepwise manner, mainly the compounds **9**, the more aromatized compounds, were obtained. On the other hand, when the reaction was carried out using a multicomponent methodology, compound **8** was chiefly obtained. When using aromatic aldehydes with bromine or methoxy groups, only the aromatized compounds **9h** (Ar = *p*-MeOC_6_H_4_), **9i** (Ar = *m*-MeOC_6_H_4_), and **9j** (Ar = *p*-MeOC_6_H_4_)) were isolated (Table 1, entries 9–11).

In order to increase the molecular diversity of these compounds and taking into account the presence of a methylene group in the structure of compounds **8** and **9**, we planned its oxidation. Among various oxidants, the selenium derivatives, mainly selenium(IV)oxide, play an important role in the preparation of oxidated organic compounds. Selenium dioxide-mediated oxidation is regarded as one of the most reliable and predictable methods for allylic hydrogenation [7,49,50,51,52].

In our case, in the oxidation with SeO_2_, the diphenyl(7-oxo-7*H*-indeno[2,1-*c*]quinolin-2-yl)phosphine oxides **10** (Scheme 3) were obtained when the diphenyl(tetrahydro-5*H*-indeno[2,1-*c*]quinolin-2-yl)phosphine oxides **8** were used, by aromatization, and by the subsequent oxidation of the methylene group at position 7, or when, separately, the diphenyl(7*H*-indeno[2,1-*c*]quinolin-2-yl)phosphine oxides **9** were utilized, by the oxidation of the methylene group at position 7.

**Chart 2:**
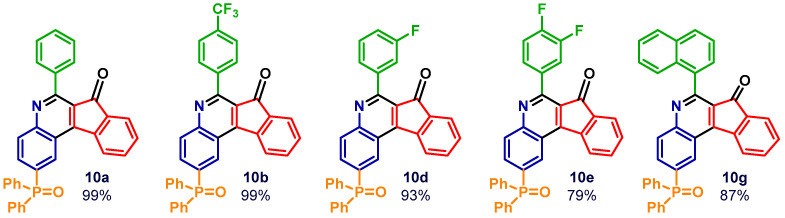
Structures of synthesized diphenyl(7-oxo-7*H*-indeno[2,1-*c*]quinolin-2-yl)phosphine oxides **10**.

Taking into account that the oxidation with the SeO_2_ of either derivatives **8** or derivatives **9** produces the carbonylic compounds **10**, the scope of the carbonyl derivatives **10** was extended by using the mixtures of derivatives **8** and **9** obtained directly in the Povarov reaction (Scheme 3); this is also more efficient. Thus, the oxidation of the mixtures of diphenyl(tetrahydro-5*H*-indeno[2,1-*c*]quinolin-2-yl)phosphine oxides **8** and diphenyl(7*H*-indeno[2,1-*c*]quinolin-2-yl)phosphine oxides **9** derivatives with SeO_2_ in refluxing dioxane produced the corresponding diphenyl(7-oxo-7*H*-indeno[2,1-*c*]quinolin-2-yl)phosphine oxides **10** (Chart 2). The formation of compounds **10** was determined by ^1^H-NMR spectroscopy where signals corresponding to the protons of the tetrahydroquinoline ring of the starting compounds **8** or the methylene signal of the more aromatic compounds **9** disappeared, and only aromatic signals were observed. Furthermore, in the ^13^C-RMN, the presence of a carbonyl group at 180–200 ppm was clearly observed.

### 2.2. Biological Results

Once diphenyl(tetrahydro-5*H*-indeno[2,1-*c*]quinolin-2-yl)phosphine oxides **8**, diphenyl(7*H*-indeno[2,1-*c*]quinolin-2-yl)phosphine oxides **9**, and diphenyl(7-oxo-7*H*-indeno[2,1-*c*]quinolin-2-yl)phosphine oxides **10** were synthesized, their activity as TOP1 inhibitors and their cytotoxicity against different cell lines were studied.

#### 2.2.1. Inhibition of Topoisomerase 1

**Figure 3:**
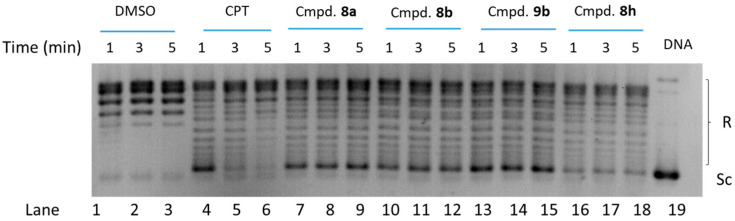
Inhibition of TOP1 relaxation activity in a time course (1, 3, and 5 min) by compounds **8a**, **8b**, **9b**, **8h**, and CPT at 100 μM: lanes 1–3: plasmid DNA + TOP1 + DMSO; lanes 4–6: plasmid DNA + TOP1 + CPT 100 μM; lanes 7–9: plasmid DNA + TOP1 + **8a** 100 μM; lanes 10–12: plasmid DNA + TOP1+ **8b** 100 μM; lanes 13–15: plasmid DNA + Top1 + **9b** 100 μM; lanes 16–18: plasmid DNA + TOP1 + **8h** 100 μM; lane 19: control plasmid DNA. Reaction samples were mixed with the enzyme at 37 °C before adding the supercoiled plasmid DNA substrate and separated by electrophoresis on a 1% agarose gel, and then stained with gel red, and photographed under UV light as described in the TOP1-mediated DNA relaxation assay. (For interpretation of the references to color in this figure legend, the reader is referred to the Web version of this article). Sc = supercoiled, R = relaxed.

The effect of the previously prepared compounds as TOP1 inhibitors was studied. A conventional supercoiled plasmid relaxation assay was used to determine if the newly fused tetrahydroindenoquinolines **8**, more aromatic indenoquinolines **9**, and carbonyl derivatives **10** with phosphine oxide substituents inhibit human TOP1 activity by converting supercoiled plasmid DNA to its relaxed form. In these experiments, compound samples were mixed with the enzyme followed by the addition of a supercoiled plasmid DNA substrate, and incubation continued for increasing time intervals (1 min, 3 min, and 5 min). The reaction was terminated by the addition of SDS. DNA relaxation products were then resolved by 1% agarose electrophoresis gel and visualized by gel red staining. CPT was used as a positive control (Figure 3).

The TOP1 inhibitory activity of the new compounds was tested in comparison to the TOP1 inhibitory activity of CPT by detecting the conversion of supercoiled DNA (Sc, Figure 3, lane 19) to its more relaxed forms in the presence of the compounds (Table 2). As shown in Figure 3, TOP1 rapidly relaxed supercoiled DNA in the presence of DMSO which was used as a solvent for the compounds and for CPT (Figure 3, lanes 1–3). CPT inhibited the degree of relaxation observed after 1 min of incubation, as indicated by the increased intensity of the band corresponding to the supercoiled DNA (Figure 3, lane 4). However, this effect disappeared after longer reaction time intervals (Figure 3, assays with synthetic compounds are shown in lanes 7–18). In contrast to this, compounds **8a**, **8b**, **9a**, and **8h** caused a more persistent inhibition of TOP1 (lanes 7–18).

**Figure 4:**
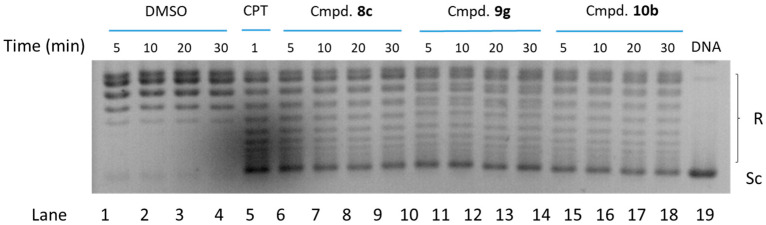
Inhibition of TOP1 relaxation activity in a time course (5 min, 10 min, 20 min, and 30 min) of compounds **8c**, **9g**, **10b**, and CPT at 100 μM: lanes 1–4: plasmid DNA + TOP1 + DMSO; lane 5: plasmid DNA + TOP1 + CPT 100 μM; lanes 6–9: plasmid DNA + TOP1 + **8c** 100 μM; lanes 10–13: plasmid DNA + Top1+ **9g** 100 μM; lanes 14–17: plasmid DNA + TOP1 + **10b** 100 μM; lane 18: control plasmid DNA. Reaction samples were mixed with the enzyme at 37 °C before adding the supercoiled plasmid DNA substrate and separated by electrophoresis on a 1% agarose gel, and then stained with gel red, and photographed under UV light as described in the TOP1-mediated DNA relaxation assay. (For interpretation of the references to color in this figure legend, the reader is referred to the Web version of this article). Sc = supercoiled; R = relaxed.

Taking into account that inhibition persisted after 5 min of enzymatic reaction, we tested the TOP1 inhibitory activity of some compounds at longer time intervals from 5 to 30 min (Figure 4, Table 2, last column).

**Figure 5:**
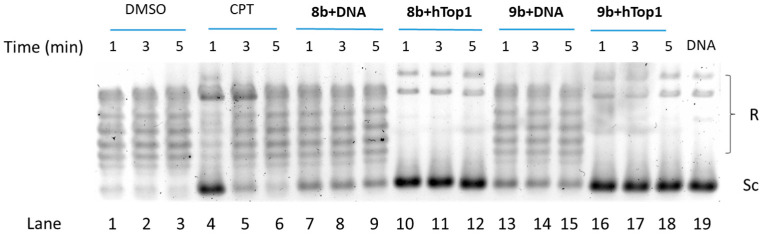
Inhibition of TOP1 relaxation activity in a time course (1 min, 3 min, and 5 min) by compounds **8b** and **9b** and CPT at 100 μM: lanes 1–3: plasmid DNA + TOP1 + DMSO; lanes 4–6: plasmid DNA + TOP1 + CPT 100 μM; lanes 7–9: the plasmid DNA was preincubated with compound **8b** at 100 μM for 15 min at 37 °C before TOP1 was added; lanes 10–12: the enzyme TOP1 was preincubated with compound **8b** at 100 μM for 15 min at 37 °C before DNA was added; lanes 13–15: the plasmid DNA was preincubated with compound **9b** at 100 μM for 15 min at 37 °C before TOP1 was added; lanes 16–18: the enzyme TOP1 was preincubated with compound **9b** at 100 μM for 15 min at 37 °C before DNA was added; lane 19: control DNA. Reaction samples were mixed and separated by electrophoresis on a 1% agarose gel, and then stained with gel red, and photographed under UV light as described in the TOP1-mediated DNA relaxation assay. (For interpretation of the references to color in this figure legend, the reader is referred to the Web version of this article). Sc = supercoiled; R = relaxed.

In order to study the possible mode of action of the compounds as inhibitors of TOP1, an additional study addressing the effect of the preincubation of the compounds with either the enzyme or DNA was carried out (Figure 5).

Therefore, the phosphorated derivatives **8b** and **9b**, with an inhibitory effect even after 30 min (Table 2, entries 3 and 11), were incubated with the DNA for 15 min before TOP1 was added (Figure 5, lanes 7–9 for **8b**, lanes 13–15 for **9b**). On the other hand, the compounds **8b** and **9b** were incubated with TOP1 for 15 min and then DNA was added (Figure 5, lanes 10–12 for **8b**, lanes 16–18 for **9b**). As evident from Figure 5, the preincubation of TOP1 with either of the compounds resulted in a more effective inhibition of enzyme activity (lanes 10–12 and 16–18) than the preincubation of the compounds with DNA (lanes 7–9 and 13–18).

#### 2.2.2. In Vitro Cytotoxicity

The cytotoxic effects of the newly synthesized diphenyl(tetrahydro-5*H*-indeno[2,1-*c*]quinolin-2-yl)phosphine oxides **8**, diphenyl(7*H*-indeno[2,1-*c*]quinolin-2-yl)phosphine oxides **9**, and diphenyl(7-oxo-7*H*-indeno[2,1-*c*]quinolin-2-yl)phosphine oxides **10** derivatives were studied against several human cell lines: A549 (carcinomic human alveolar basal epithelial cell), SKOV3 (human ovarian carcinoma), and HEK293 (human embryonic kidney). Furthermore, in order to analyze the selective cytotoxicity facing exclusively cancerous cell lines, toxicity against a non-cancerous cell line (MRC-5, lung fibroblast cells) was also evaluated (Table 3). The cell counting kit (CCK-8) assay was employed to assess the growth inhibition and cell proliferation inhibitory activities of the compounds, which are described in Table 3 as IC_50_ values.

### 2.3. Computational ADME Properties

In silico ADME studies involving the use of computational models and algorithms to predict the absorption, distribution, metabolism, and excretion of a drug are of vital importance in the drug discovery and development process, as they ensure the characterization of drug candidates at an early stage and can significantly reduce overall drug development costs.

With the aim of predicting these molecular properties of the previously synthesized compounds, some parameters were studied, such as the partition coefficient (LogP_o/w_), molecular weight, hydrogen bond donors and acceptors, topological polar surface area (TPSA), and viol. of Lipinski’s rule of five and Veber’s rule (Table 4).

Lipinski’s rule of five states that a compound with a molecular mass under 500 g/mol (MW), with no more than five hydrogen bond donors (HBD) and no more than 10 hydrogen bond acceptors (HBA), could be a good drug candidate. Veber’s rule says that a compound with 10 or fewer rotatable bonds (RB) and a polar surface area (TPSA) no greater than 140 Å^2^ should present good oral bioavailability [53].

## 3. Discussion

There is a wide spectrum of medicinal compounds with significant biological activity, such as antiviral, antibiotic, and antitumor drugs, with a nitrogen heterocycle in their structure which is important to improve interactions in biological systems [54,55,56,57]. Among the different heterocyclic scaffolds, quinoline serves as a privileged medicinal block showing a broad range of pharmacological properties including antiproliferative and anticancer activity [58].

Therefore, in the drug discovery process, the development of accessible synthetic routes that allow for the preparation of this type of molecule in a simple way stands out. In fact, among them, the Povarov reaction may represent a direct route for the preparation of nitrogen containing heterocyclic derivatives with excellent regioselectivity [27,28,29,30].

In this work, we use the Povarov-type reaction to prepare phosphorylated quinoline derivatives, demonstrating that this methodology is adequate for this purpose and tolerates a wide range of electron-releasing and electron-withdrawing substituents in aromatic aldehydes, even fluorinated ones, interesting substrates from a biological point of view [59]. As far as we know, this methodology represents the first example for the preparation of hybrid fused tetrahydroquinolines **8**, quinolines **9**, and quinolin-7-ones **10** containing a phosphine oxide group. The quasi-planar structure may be important for the inhibitory activity of the topoisomerase 1 (TOP1) enzyme [60]. Therefore, the next step was to perform the biological study of these compounds as TOP1 inhibitors and as antiproliferative agents against different cancerous and non-cancerous cell lines.

As observed in Table 2, some compounds present similar or higher inhibition than CPT at short enzymatic reaction times (1 min) such as **8a**, **8b**, **8c**, **8d**, **8e**, **9b**, **9f**, **9g**, **9h**, **9i**, **10b**, and **10e** (Table 2, entries 2–6, 11, 14–17, 20, and 22). In the relaxation tests of the new compounds **8**, **9**, and **10** with TOP1, we observed that all of them showed a sustained inhibition up to 5 min (Table 2). Taking into account this result, we additionally studied the inhibition of some of the compounds at longer incubation times (Figure 4) and observed that, unlike CPT which has a short-term reversible effect, the enzymatic inhibition of the new compounds was maintained over time up to 30 min, with the same intensity during the 30 min.

This led us to investigate the possible mode of action of the compounds as TOP1 inhibitors, for which we carried out an additional study addressing the effect of their preincubation either with the enzyme or with the DNA (Figure 5). Thus, those phosphorated compounds derived from *p*-trifluoromethylbenzaldehyde (Ar = *p*-CF_3_C_6_H_4_) **8b** and **9b**, which presented a strong inhibitory effect even after 30 min (Table 2, entries 3 and 11), were incubated with DNA for 15 min before TOP1 was added. In this case, the TOP1 enzyme exhibited similar behavior to that observed previously without preincubation with DNA, partially relaxing the DNA (Figure 5, lanes 7–9 for **8b**, lanes 13–15 for **9b**). On the other hand, when the compounds were incubated with TOP1 for 15 min and then DNA was added, a completely different behavior was observed. During the 30 min of enzymatic reaction, the enzyme was inactive, not being able to relax the DNA, as only the supercoiled plasmid signal (Sc) was observed in the gel (Figure 5, lanes 10–12 for **8b** and lanes 16–18 for **9b**). This suggests that the compounds inhibit TOP1 activity by interacting directly with the enzyme rather than intercalating DNA or interacting in the interface between the enzyme and DNA as is the case of CPT.

**Figure 6:**
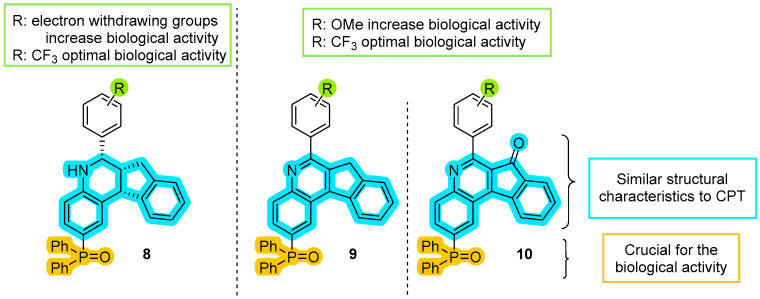
Structure–activity relationship analysis.

Very interesting results were obtained in the cytotoxicity study carried out as shown in Figure 6. Some derivatives **8** showed cytotoxicity against cancer cells (A549 and SKOV3). Thus, compound **8a** (Ar = C_6_H_5_) shows the highest cytotoxicity against SKOV3 with IC_50_ = 6.35 ± 1.73 µM and moderate cytotoxicity against A549 with IC_50_ = 40.18 ± 7.13 µM (see Table S1 in Supplementary Materials). Compounds **8b** (Ar = *p*-CF_3_C_6_H_4_), **8c** (Ar = *p*-FC_6_H_4_), and **8d** (Ar = *m*-FC_6_H_4_) showed cytotoxicity against the A549 lung cancer line (IC_50_ = 25.82 ± 6.98 µM, 19.94 ± 3.35 µM, and 43.10 ± 12.14 µM, respectively) and compounds **8e** (Ar = 3,4-F_2_C_6_H_3_) and **8f** (Ar = 2,4-F_2_C_6_H_3_) against the ovarian cancer line SKOV3 (IC_50_ = 13.19 ± 2.79 µM and 31.04 ± 3.17 µM, respectively). Interestingly, none of the compounds show cytotoxicity against non-cancer cell lines such as HEK293 and MRC5 (except **8c** (Ar = *p*-FC_6_H_4_) with IC_50_ = 10.71 ± 0.43 µM against MRC5).

For the dehydrogenated derivatives **9** and the oxidized derivatives **10**, the cytotoxicity is very low against all the tested cell lines, cancerous or non-cancerous. Only slight cytotoxicity was observed for **9f** (Ar = *m*-CH_3_OC_6_H_4_) and **9j** (Ar = *m*-CH_3_OC_6_H_4_) against A549 (Table 3, entries 14 and 17).

Taking into account the biological results obtained and the structural differences of the compounds prepared, a Structure–Activity Relationship (SAR) study has been carried out (Figure 6).

Based on the biological results obtained, it has been observed that diphenyl *endo*-tetrahydro-5*H*-indeno[2,1-*c*]quinolin-2-ylphosphine oxides derivatives **8** with an electron-withdrawing group (R) in their structure increases the biological activity of the compounds. On the other hand, for diphenyl(7*H*-indeno[2,1-*c*]quinolin-2-yl)phosphine oxides **9** derivatives, a methoxy group (R) resulted in a higher activity. It should be noted that in synthesized **8**, **9**, and **10** derivatives, the presence of a trifluoromethyl group (Ar = *p*-CF_3_C_6_H_4_) increases the topoisomerase I inhibition considerably.

These findings offer important insights into the structure–activity relationships (SAR) of the studied compounds, enhancing our understanding of the key factors that influence their biological activity. By identifying the specific structural elements that contribute to their potency and selectivity, the results pave the way for the rational design and optimization of future compounds with enhanced bioactive properties. This knowledge not only guides the development of more effective therapeutic agents but also supports the strategic improvement of compound libraries for targeted drug discovery.

These results are encouraging, as the cytotoxicity can be modulated depending on the structure of the fused heterocycles, whether it is partly aromatized/oxidized or not, and presenting low or no toxicity against non-cancerous cells, unlike CPT (reference compound) which is cytotoxic against both cancerous and non-cancerous cell lines (Table 3, entry 1).

In general, all the products present high lipid solubility (LogP_o/w_) and a polarity surface (TPSA) under 140 Å, which could be beneficial for the absorption, distribution, and metabolism of the drug candidates, including having good oral bioavailability. Additionally, all the compounds were predicted to have drug-likeliness properties achieving the Veber’s rule and two or fewer violations of the Lipinsky’s rule [61].

As it can be seen in Table S1, the aqueous solubility (Log S) of all the compounds ranges from −4.821 to −3.155 log mol/L, which shows a moderate solubility in water. All the synthesized compounds present 100% intestinal absorption, which is interesting because most orally administrated drugs are absorbed mainly through the small intestine due its large surface area [62]. On the other hand, the Caco-2 permeability has been studied, which is the most used parameter to evaluate in vitro gastrointestinal permeability, and almost all the derivatives have values near 0.90 (this value is considered to be high if >0.90, Table S1), even higher in some cases except in the case of derivative **8k** with a nitro substituent (Ar = *p*-NO_2_C_6_H_4_) in the structure, which shows a very low permeability.

The volume of distribution (VDss) was studied and, considering that Log VDss > 0.45 L/Kg indicates a high volume of drug distribution, we can say that all our compounds have low distribution in tissue.

P-glycopotein (PGP) is an ATP-binding cassette multidrug transporter and is expressed broadly throughout the human body, such as in the gastrointestinal tract, liver, and kidneys. Numerous structurally diverse therapeutic agents are being recognized as substrates to PGP, and it reportedly holds back their absorption and permeability by exuding them out of cells, protecting the cells against toxicity or unwanted side effects. As shown in Table S1, all the compounds **8** present the quality of being PGP substrates, but with the oxidation of the compounds, this quality decreases; thus, derivatives **10** are not substrates. On the other hand, all the compounds are inhibitors of PGP, except in the case of **9d** and **9i** which are not inhibitors of the PGP I enzyme.

Further, the assessment of the drug candidates to inhibit or inactivate CYP 450 enzymes (CYP1A2, CYP2C19, CYP2C9, CYP2D6, and CYP3A4) is an essential part of therapeutic drug discovery and development. Inhibition or inactivation of these enzymes may also result in clinical drug–drug interactions leading to dangerous side effects as well as reduced clearance of the drugs [63]. Of note, all the tetrahydro-5*H*-indeno[2,1-*c*]quinolin-2-ylphosphine oxides **8** derivatives and most of the 7*H*-indeno[2,1-*c*]quinolin-2-ylphosphine oxides **9** and 7-oxo-7*H*-indeno[2,1-*c*]quinolinylphosphine oxides **10** show the ability of inhibiting the CYP1A2, except compounds **9a**, **9b**, **9g**, **9j**, **10e**, and **10g**.

Moreover, the total clearance is primarily a combination of hepatic and renal clearance and is measured by the proportionality constant total CL in log (mL/min/Kg). The theoretical values of these compounds ranged between −0.692 and 0.151, indicating a low clearance.

The interesting biochemical and biological features found for these derivatives provide a promising basis for the further development of biologically active indenoquinolinylphosphine oxides.

## 4. Materials and Methods

### 4.1. Chemistry

#### General Experimental Information

All reagents from commercial suppliers were used without further purification. All solvents were freshly distilled before use from appropriate drying agents. All other reagents were recrystallized or distilled when necessary. Reactions were performed under a dry nitrogen atmosphere. Analytical TLCs were performed with silica gel 60 F254 plates. Visualization was accomplished by UV light.

Column chromatography was carried out using silica gel 60 (230ε400 mesh ASTM). Melting points were determined with an Electrothermal IA9100 Digital Melting Point Apparatus without correction. NMR spectra were obtained on a Bruker Avance 400 MHz spectrometer and recorded at 25 °C. Chemical shifts for ^1^H NMR spectra are reported in ppm downfield from TMS, chemical shifts for ^13^C NMR spectra are recorded in ppm relative to internal deuterated chloroform (δ = 77.2 ppm for ^13^C), and chemical shifts for ^19^F NMR are reported in ppm downfield from fluorotrichloromethane (CFCl_3_). Coupling constants (*J*) are reported in Hertz. The terms m, s, d, t, q refer to multiplet, singlet, doublet, triplet, quartet, respectively. ^13^C NMR and ^19^F NMR were broadband decoupled from hydrogen nuclei. High resolution mass spectra (HRMS) were measured by the EI method with a Agilent LC-Q-TOF-MS 6520 spectrometer.

Compounds purity analysis

All synthesized compounds were analyzed by HPLC to determine their purity. The analyses were performed on the Agilent 1260 infinity HPLC system (C-18 column, Hypersil, BDS, 5 μm, 0.4 mm × 25 mm). All tested compounds were dissolved in dichloromethane, and 5 μL of the sample was loaded onto the column. Ethanol and heptane were used as the mobile phase, and the flow rate was set at 1 mL/min. The maximal absorbance at the range of 190–400 nm was used as the detection wavelength. The purity of all the tested compounds (compounds **8**, **9** and **10**) is >95%, which meets the purity requirement.

Synthesis of (4-aminophenyl)diphenylphosphine oxide **3**

In a flame-dried Schlenk flask, copper iodide (1.5 mmol, 0.29 g, 10 mol %) was added under inert atmosphere. Then, (S)-α-phenylethylamine (3 mmol, 0.38 mL, 20 mol %) was added. After 5 min, toluene (20 mL) was added followed by the addition of 4-iodoaniline **1** (15 mmol, 3.3 g, 1 equiv.), diphenylphosphine oxide **2** (18 mmol, 3.7 g, 1.2 equiv.), and anhydrous MgSO_4_ as the drying agent. After the mixture was stirred for 5 min, potassium carbonate (30 mmol, 4.15 g, 2 equiv.) was added and the reaction mixture was stirred at 110 °C overnight. Afterward, the mixture was allowed to cool to room temperature and was filtered through a short pad of celite under reduced pressure. The celite was washed three times with DCM and the filtrate was evaporated under reduced pressure. The residue was recrystallized in diethyl ether affording 13.7 g (91%) of a white solid identified as (4-aminophenyl)diphenylphosphine oxide **3**, mp 265–268 °C. ^1^H-NMR (400 MHz, CDCl_3_) δ (ppm): 7.69–7.63 (m, 4H), 7.53–7.49 (m,2H), 7.45–7.37 (m, 6H), 6.68–6.66 (m, 2H), 4.06 (s, 2H); ^13^C {^1^H} NMR (75 MHz, CDCl_3_) δ (ppm): 149.8 (d, ^4^*J*_CP_ = 2.4 Hz, C), 133.8 (d, ^3^*J*_CP_ = 11.2 Hz, 2CH), 133.4 (d, ^1^*J*_CP_ = 103.9 Hz, 2C), 132.1 (d, ^3^*J*_CP_ = 9.8 Hz, 4CH), 131.6 (d, ^4^*J*_CP_ = 2.6 Hz, 2CH), 128.3 (d, ^2^*J*_CP_ = 12.0 Hz, 4CH), 120.1 (d, ^1^*J*_CP_ = 113.4 Hz, C), 114.3 (d, ^2^*J*_CP_ = 13.1 Hz, 2CH); ^31^P {^1^H} NMR (162 MHz, CDCl_3_) δ (ppm): 29.4. ESI-HRMS (CI) *m/z* calculated for C_18_H_17_NOP [M+H]^+^ 293.0970; found 293.0976.

Synthesis of diphenyl(tetrahydro-5*H*-indeno[2,1-*c*]quinolin-2-yl)phosphine oxide derivatives **8** and diphenyl(7*H*-indeno[2,1-*c*]quinolin-2-yl)phosphine oxide derivatives **9**

General procedure A.

To a solution of phosphine oxide aniline **3** (1 mmol, 0.3 g) in CHCl_3_ (20 mL), the corresponding freshly distilled aldehyde **4** (1 mmol) was added in the presence of molecular sieves (4 Å). The mixture was stirred under nitrogen at the opportune temperature until consumption of the starting materials was checked by ^1^H NMR and ^31^P NMR spectroscopy. To the solution, indene **6** (1.5 mmol, 0.17 mL, 1.2 equiv) and two equivalents of BF_3_·Et_2_O (2 mmol, 0.19 mL) were added, stirred, and heated to reflux until TLC and ^1^H NMR analysis indicated the consumption of the starting materials. The molecular sieves were removed by filtration and the resulting solution was washed with a solution of NaOH 2M (50 mL) and with water (50 mL), extracted with dicloromethane (2 × 20 mL), and dried (MgSO_4_). After the removal of the solvent under vacuum produced an oil that was purified by silica gel column chromatography (hexane/ethyl acetate), a mix of products **8** and **9** was obtained.

General procedure B.

A mixture of phosphine oxide aniline **3** (1 mmol, 0.3 g), aldehyde (1 mmol) in CHCl_3_ (10 mL), indene (1.5 mmol, 0.17 mL, 1.2 equiv), and two equivalents of BF_3_·Et_2_O (2 mmol, 0.19 mL) in the presence of molecular sieves (4 Å) was stirred and heated to reflux. The suspension was stirred and heated to reflux until TLC and ^1^H NMR analysis indicated the consumption of the starting materials. The molecular sieves were removed by filtration and the resulting solution was washed with a solution of NaOH 2M (50 mL) and with water (50 mL), extracted with dicloromethane (2 × 20 mL), and dried (MgSO_4_). The removal of the solvent under vacuum led to an oil that was purified by column chromatography on silica gel using 40–60 ethyl acetate in hexane to produce a mix of compounds **8** and **9**.

**Diphenyl(6-phenyl-6,6a,7,11b-tetrahydro-5*H*-indeno[2,1-*c*]quinolin-2-yl)phosphine oxide (8a).** The general *procedure A* was followed using (4-aminophenyl)diphenylphosphine oxide **3** (1 mmol, 0.3 g) and benzaldehyde **4a** (1 mmol, 0.1 mL); after refluxing in CHCl_3_ 24 h, indene **6** (1.5 mmol) and BF_3_·Et_2_O were added and stirred for 48 h. The product was purified by column chromatography (silica gel, hexane:EtOAc 40:60) resulting in 0.15 g (31%) of a gray solid identified as **8a** with a mp of 232–235 °C. When the general *procedure B* was followed, 0.25 g (31%) of compound **8a** was obtained. ^1^H-NMR (400 MHz, CDCl_3_) δ (ppm): 7.69–7.60 (m, 4H), 7.55–7.49 (m,3H), 7.48–7.39 (m, 8H), 7.36–7.33 (m, 1H), 7.21–7.18 (m, 1H), 7.13–7.07 (m, 2H), 7.05–7.00 (m, 2H), 6.62 (dd, *J* = 8.2, 2.8 Hz, 1H), 4.77 (d, *J* = 2.9 Hz, 1H), 4.48 (d, *J* = 7.7 Hz, 1H), 4.30 (s, 1H), 3.27–3.21 (m, 1H), 3.19–3.12 (m, 1H), 2.38 (dd, *J* = 14.9, 7.1 Hz, 1H); ^13^C {^1^H} NMR (75 MHz, CDCl_3_) δ (ppm): 148.5 (d, ^4^*J*_CP_ = 2.4 Hz, C), 145.6 (C), 142.3 (C), 141.7 (C), 134.3 (d, ^2^*J*_CP_ = 11.5 Hz, CH), 133.2 (d, ^1^*J*_CP_ = 104.4 Hz, 2C), 132.1 (d, ^3^*J*_CP_ = 10.1 Hz, 2CH), 132.0 (d, ^3^*J*_CP_ = 10.4 Hz, 2CH), 131.5 (d, ^4^*J*_CP_ = 2.9 Hz, CH), 131.5 (d, ^4^*J*_CP_ = 3.0 Hz, CH), 130.7 (d, ^2^*J*_CP_ = 11.0 Hz, CH), 128.7 (2CH), 128.3 (d, ^2^*J*_CP_ = 12.0 Hz, 2CH), 128.2 (d, ^2^*J*_CP_ = 11.9 Hz, 2CH), 127.7 (CH), 127.0 (CH), 126.5 (CH), 126.4 (2CH), 124.8 (CH), 124.7 (CH), 123.4 (d, ^3^*J*_CP_ = 12.7 Hz, C), 120.3 (d, ^1^*J*_CP_ = 112.4 Hz, C), 115.2 (d, ^3^*J*_CP_ = 13.1 Hz, CH), 57.1 (CH), 47.6 (CH), 45.7 (CH), 31.2 (CH_2_); ^31^P {^1^H} NMR (162 MHz, CDCl_3_) δ (ppm): 29.3. ESI-HRMS (CI) *m/z* calculated for C_34_H_29_NOP [M+H]^+^ 498. 1987; found 498.1966.

**Diphenyl(6-(4-(trifluoromethyl)phenyl)-6,6a,7,11b-tetrahydro-5*H*-indeno[2,1-*c*]quinolin-2-yl)phosphine oxide (8b).** The general *procedure B* was followed using (4-aminophenyl)diphenylphosphine oxide **3** (1 mmol, 0.3 g), 4-(trifluoromethyl)benzaldehyde **4b** (1 mmol, 0.11 mL), indene **6** (1.5 mmol), and BF_3_·Et_2_O and stirred for 48 h at reflux in CHCl_3_. The product was purified by column chromatography (silica gel, hexane:EtOAc 60:40) resulting in 0.32 g (57%) of a gray solid identified as **8b** with a mp of 223–226 °C. ^1^H-NMR (400 MHz, CDCl_3_) δ (ppm): 7.67–7.63 (m, 4H), 7.62–7.57 (m, 4H) 7.55–7.49 (m,3H), 7.45–7.39 (m, 4H), 7.23–7.17 (m, 1H), 7.12–7.07 (m, 2H), 7.04–7.02 (m, 2H), 6.66 (dd, *J* = 8.3, 2.7 Hz, 1H), 4.81 (d, *J* = 2.8 Hz, 1H), 4.48 (d, *J* = 7.6 Hz, 1H), 4.45 (s, 1H), 3.24–3.12 (m, 2H), 2.33 (dd, *J* = 14.3, 6.6 Hz, 1H); ^13^C {^1^H} NMR (75 MHz, CDCl_3_) δ (ppm): 148.1 (d, ^4^*J*_CP_ = 2.5 Hz, C), 145.8 (d, ^5^*J*_CF_ = 1.1 Hz, C), 145.4 (C), 141.9 (C), 134.3 (d, ^2^*J*_CP_ = 11.6 Hz, CH), 133.2 (d, ^1^*J*_CP_ = 103.7 Hz, C), 133.2 (d, ^1^*J*_CP_ = 104.5 Hz, C), 132.1 (d, ^3^*J*_CP_ = 10.0 Hz, 2CH), 132.0 (d, ^3^*J*_CP_ = 9.9 Hz, 2CH), 131.5 (d, ^4^*J*_CP_ = 2.9 Hz, CH), 131.4 (d, ^4^*J*_CP_ = 2.8 Hz, CH), 130.8 (d, ^2^*J*_CP_ = 10.9 Hz, CH), 129.9 (q, ^2^*J*_CF_ = 32.5 Hz, C), 128.3 (d, ^2^*J*_CP_ = 12.1 Hz, 2CH), 128.3 (d, ^2^*J*_CP_ = 12.1 Hz, 2CH), 127.1 (CH), 126.9 (2CH), 126.6 (CH), 125.6 (q, ^3^*J*_CF_ = 3.7 Hz, 2CH), 124.8 (CH), 124.7 (CH), 124.3 (q, ^1^*J*_CF_ = 271.8 Hz, CF3), 123.3 (d, ^3^*J*_CP_ = 12.7 Hz, C), 120.9 (d, ^1^*J*_CP_ = 112.3 Hz, C), 115.5 (d, ^3^*J*_CP_ = 13.2 Hz, CH), 56.9 (CH), 47.3 (CH), 45.6 (CH), 31.1 (CH2); ^31^P {^1^H} NMR (162 MHz, CDCl_3_) δ (ppm): 29.1. ^19^F NMR (376 MHz, CDCl_3_) δ −62.5. ESI-HRMS (CI) *m/z* calculated for C_35_H_28_F_3_NOP [M+H]^+^ 566.1861; found 566.1850. 

**Diphenyl(6-(4-fluorophenyl)-6,6a,7,11b-tetrahydro-5*H*-indeno[2,1-*c*]quinolin-2-yl)phosphine oxide (8c).** The general *procedure B* was followed using (4-aminophenyl)diphenylphosphine oxide **3** (1 mmol, 0.3 g), 4-fluorobenzaldehyde **4c** (1 mmol, 0.11 mL), indene **6** (1.5 mmol), and BF_3_·Et_2_O and stirred for 48 h at reflux in CHCl_3_. The product was purified by column chromatography (silica gel, hexane:EtOAc 60:40) resulting in 0.24 g (47%) of a gray solid identified as **8c** with a mp of 245–247 °C. ^1^H-NMR (400 MHz, CDCl_3_) δ (ppm): 7.68–7.59 (m, 4H), 7.56–7.49 (m, 3H) 7.46–7.38 (m, 6H), 7.23–7.18 (m, 1H), 7.12–7.02 (m, 6H), 6.62 (dd, *J* = 8.3, 2.7 Hz, 1H), 4.75 (d, *J* = 3.0 Hz, 1H), 4.47 (d, *J* = 7.8 Hz, 1H), 4.27 (s, 1H), 3.24–3.18 (m, 1H), 3.14–3.07 (m, 1H), 2.37 (dd, *J* = 15.0, 7.2 Hz, 1H); ^13^C {^1^H} NMR (75 MHz, CDCl_3_) δ (ppm): 162.2 (d, ^1^*J*_CF_ = 246.1 Hz, CF), 148.4 (d, ^4^*J*_CP_ = 2.5 Hz, C), 145.6 (C), 142.1 (C), 137.5 (d, ^4^*J*_CF_ = 3.0 Hz, C), 134.3 (d, ^2^*J*_CP_ = 11.4 Hz, CH), 133.2 (d, ^1^*J*_CP_ = 103.9 Hz, C), 133.2 (d, ^1^*J*_CP_ = 103.9 Hz, C), 132.2 (d, ^3^*J*_CP_ = 10.0 Hz, CH), 132.1 (d, ^3^*J*_CP_ = 10.1 Hz, 2CH), 132.0 (d, ^3^*J*_CP_ = 9.9 Hz, 2CH), 131.5 (d, ^4^*J*_CP_ = 2.8 Hz, CH), 131.5 (d, ^4^*J*_CP_ = 2.8 Hz, CH), 130.8 (d, ^3^*J*_CF_ = 10.9 Hz, 2CH), 128.3 (d, ^2^*J*_CP_ = 12.0 Hz, 2CH), 128.3 (d, ^2^*J*_CP_ = 12.0 Hz, 2CH), 127.0 (CH), 126.6 (CH), 124.9 (CH), 124.7 (CH), 123.4 (d, ^3^*J*_CP_ = 12.7 Hz, C), 120.6 (d, ^1^*J*_CP_ = 112.4 Hz, C), 115.5 (d, ^2^*J*_CF_ = 21.2 Hz, 2CH), 115.3 (d, ^2^*J*_CP_ = 13.0 Hz, CH), 56.6 (CH), 47.6 (CH), 45.7 (CH), 31.1 (CH2); ^31^P {^1^H} NMR (162 MHz, CDCl_3_) δ (ppm): 29.1. ^19^F NMR (376 MHz, CDCl_3_) δ −114.7. ESI-HRMS (CI) *m/z* calculated for C_34_H_28_FNOP [M+H]^+^ 516.1893; found 516.1881. 

**Diphenyl(6-(3-fluorophenyl)-6,6a,7,11b-tetrahydro-5*H*-indeno[2,1-*c*]quinolin-2-yl)phosphine oxide (8d).** The general *procedure B* was followed using (4-aminophenyl)diphenylphosphine oxide **3** (1 mmol, 0.3 g), 3-fluorobenzaldehyde **4d** (1 mmol, 0.11 mL), indene **6** (1.5 mmol), and BF_3_·Et_2_O and stirred for 48 h at reflux in CHCl_3_. The product was purified by column chromatography (silica gel, hexane:EtOAc 65:35) resulting in 0.19 g (37%) of a yellow solid identified as **8d** with a mp of 178–181 °C. ^1^H-NMR (400 MHz, CDCl_3_) δ (ppm): 7.82–7.74 (m, 1H), 7.69–7.60 (m, 4H) 7.56–7.34 (m,8H), 7.25–7.19 (m, 3H), 7.13–7.01 (m, 4H), 6.63 (dd, *J* = 8.2, 2.7 Hz, 1H), 4.78 (d, *J* = 2.8 Hz, 1H), 4.48 (d, *J* = 7.5 Hz, 1H), 4.21 (d, *J* = 10.5 Hz, 1H), 3.25–3.13 (m, 2H), 2.39 (dd, *J* = 14.3, 6.6 Hz, 1H), 1.60 (s, 1H); ^13^C {^1^H} NMR (75 MHz, CDCl_3_) δ (ppm): 163.1 (d, ^1^*J*_CF_ = 244.9 Hz, CF), 148.1 (d, ^4^*J*_CP_ = 2.3 Hz, C), 145.5 (C), 144.5 (d, ^3^*J*_CF_ = 6.9 Hz, C), 142.1 (C), 134.4 (d, ^2^*J*_CP_ = 11.6 Hz, CH), 133.2 (d, ^1^*J*_CP_ = 103.9 Hz, 2C), 132.2 (d, ^3^*J*_CP_ = 10.1 Hz, 2CH), 132.1 (d, ^3^*J*_CP_ = 9.9 Hz, 2CH), 131.5 (d, ^4^*J*_CP_ = 2.8 Hz, CH), 131.5 (d, ^4^*J*_CP_ = 2.9 Hz, CH), 130.8 (d, ^3^*J*_CP_ = 11.3 Hz, CH), 130.3 (d, ^3^*J*_CG_ = 8.6 Hz, CH), 128.3 (d, ^2^*J*_CP_ = 12.1 Hz, 2CH), 128.3 (d, ^2^*J*_CP_ = 12.1 Hz, 2CH), 127.1 (CH), 126.6 (CH), 124.9 (CH), 124.7 (CH), 123.5 (d, ^3^*J*_CP_ = 12.8 Hz, C), 122.1 (d, ^4^*J*_CF_ = 2.8 Hz, CH), 121.0 (d, ^1^*J*_CP_ = 112.2 Hz, C), 115.4 (d, ^2^*J*_CP_ = 13.1 Hz, CH), 114.6 (d, ^2^*J*_CF_ = 21.2 Hz, CH), 113.4 (d, ^2^*J*_CF_ = 22.4 Hz, CH), 56.8 (d, ^4^*J*_CF_ = 1.5 Hz, CH), 47.5 (CH), 45.7 (CH), 31.1 (CH2); ^31^P {^1^H} NMR (162 MHz, CDCl_3_) δ (ppm): 29.3. ^19^F NMR (376 MHz, CDCl_3_) δ −112.3. ESI-HRMS (CI) *m/z* calculated for C_34_H_28_FNOP [M+H]^+^ 516.1893; found 516.1881.

**Diphenyl(6-(3,4-difluorophenyl)-6,6a,7,11b-tetrahydro-5*H*-indeno[2,1-*c*]quinolin-2-yl)phosphine oxide (8e).** The general *procedure B* was followed using (4-aminophenyl)diphenylphosphine oxide **3** (1 mmol, 0.3 g), 3,4-difluorobenzaldehyde **4e** (1 mmol, 0.1 mL), indene **6** (1.5 mmol), and BF_3_·Et_2_O and stirred for 48 h at reflux in CHCl_3_. The product was purified by column chromatography (silica gel, hexane:EtOAc 50:50) resulting in 0.42 g (78%) of a yellow solid identified as **8e** with a mp of 121–124 °C. ^1^H-NMR (400 MHz, CDCl_3_) δ (ppm): 7.67–7.58 (m, 4H), 7.56–7.49 (m, 3H), 7.46–7.39 (m,4H), 7.33–7.28 (m, 1H), 7.23–7.17 (m, 3H), 7.12–7.01 (m, 4H), 6.64 (dd, *J* = 8.2, 2.7 Hz, 1H), 4.72 (s, 1H), 4.46 (d, *J* = 7.6 Hz, 1H), 4.31 (s, 1H), 3.22–3.08 (m, 2H), 2.37 (dd, *J* = 14.8, 7.0 Hz, 1H); ^13^C {^1^H} NMR (75 MHz, CDCl_3_) δ (ppm): 150.3 (dd, ^1^*J*_CF_ = 248.4 Hz, ^2^*J*_CF_ = 12.8 Hz, CF), 149.5 (dd, ^1^*J*_CF_ = 248.1 Hz, ^2^*J*_CF_ = 12.5 Hz, CF), 148.2 (d, ^4^*J*_CP_ = 2.6 Hz, C), 145.4 (C), 141.9 (C), 138.9 (dd, ^3^*J*_CF_ = 4.9 Hz, ^4^*J*_CF_ = 3.8 Hz, C),134.2 (d, ^2^*J*_CP_ = 11.5 Hz, CH), 133.0 (d, ^1^*J*_CP_ = 104.2 Hz, 2C), 132.0 (d, ^3^*J*_CP_ = 9.9 Hz, 2CH), 131.9 (d, ^3^*J*_CP_ = 10.0 Hz, 2CH), 131.5 (d, ^4^*J*_CP_ = 3.2 Hz, CH), 131.5 (d, ^4^*J*_CP_ = 3.2 Hz, CH), 130.6 (d,^3^*J*_CP_ = 10.8 Hz, CH), 128.2 (d, ^2^*J*_CP_ = 12.1 Hz, 2CH), 128.2 (d, ^2^*J*_CP_ = 12.0 Hz, 2CH), 127.0 (CH), 126.5 (CH), 124.7 (CH), 124.6 (CH), 123.0 (d, ^3^*J*_CP_ = 12.7 Hz, C), 122.4 (dd, ^3^*J*_CF_ = 6.2 Hz, ^4^*J*_CF_ = 3.5 Hz, CH), 120.3 (d, ^1^*J*_CP_ = 112.6 Hz, C), 117.3 (d, ^2^*J*_CF_ = 17.2 Hz, CH), 115.5 (d, ^2^*J*_CP_ = 13.1 Hz, CH), 115.4 (d, ^2^*J*_CF_ = 17.8 Hz, CH), 56.1 (CH), 47.3 (CH), 45.4 (CH), 31.0 (CH2); ^31^P {^1^H} NMR (162 MHz, CDCl_3_) δ (ppm): 29.3. ^19^F NMR (376 MHz, CDCl_3_) δ −136.75 (d, *J* = 21.3 Hz), −139.00 (d, *J* = 20.7 Hz). ESI-HRMS (CI) *m/z* calculated for C_34_H_27_F_2_NOP [M+H]^+^ 534.1798; found 534.1787.

**Diphenyl(6-(2,4-difluorophenyl)-6,6a,7,11b-tetrahydro-5*H*-indeno[2,1-*c*]quinolin-2-yl)phosphine oxide (8f).** The general *procedure A* was followed using (4-aminophenyl)diphenylphosphine oxide **3** (1 mmol, 0.3 g) and 2,4-difluorobenzaldehyde **4f** (1 mmol, 0.1 mL); after refluxing in CHCl_3_ 24 h, indene **6** (1.5 mmol) and BF_3_·Et_2_O were added and stirred for 48 h. The product was purified by column chromatography (silica gel, hexane:EtOAc 65:35) resulting in 0.18 g (33%) of a yellow solid identified as **8f** with a mp of 248–250 °C. When the general *procedure B* was followed, 0.38 g (71%) of compound **8f** was obtained. ^1^H-NMR (400 MHz, CDCl_3_) δ (ppm): 7.67–7.59 (m, 5H), 7.54–7.49 (m, 3H) 7.46–7.39 (m, 4H), 7.24–7.19 (m, 1H), 7.11–7.07 (m, 2H), 7.04–7.00 (m, 1H), 6.87–6.81 (m, 1H), 6.54 (dd, *J* = 8.3, 2.7 Hz, 1H), 5.06 (s, 1H), 4.48 (d, *J* = 7.4 Hz, 1H), 4.22 (s, 1H), 3.27–3.15 (m, 2H), 2.38 (dd, *J* = 13.8, 6.1 Hz, 1H); ^13^C {^1^H} NMR (75 MHz, CDCl_3_) δ (ppm): 162.1 (dd, ^1^*J*_CF_ = 248.9 Hz, ^3^*J*_CF_ = 12.2 Hz, CF), 160.6 (dd, ^1^*J*_CF_ = 249.2 Hz, ^3^*J*_CF_ = 11.8 Hz, CF), 148.4 (d, ^4^*J*_CP_ = 2.5 Hz, C), 145.5 (C), 142.0 (C), 134.2 (d, ^2^*J*_CP_ = 11.7 Hz, CH), 133.1 (d, ^1^*J*_CP_ = 104.1 Hz, C), 133.1 (d, ^1^*J*_CP_ = 104.2 Hz, C), 132.1 (d, ^3^*J*_CP_ = 9.3 Hz, 2CH), 132.0 (d, ^3^*J*_CP_ = 9.4 Hz, 2CH), 131.5 (d, ^4^*J*_CP_ = 2.9 Hz, CH), 131.5 (d, ^4^*J*_CP_ = 3.0 Hz, CH), 130.7 (d,^3^*J*_CP_ = 10.9 Hz, CH), 128.3 (d, ^2^*J*_CP_ = 12.2 Hz, 2CH), 128.2 (d, ^2^*J*_CP_ = 12.1 Hz, 2CH), 128.2 (t, ^3^*J*_CF_ = 6.2 Hz, CH), 127.0 (CH), 126.7 (CH), 124.9 (dd, ^2^*J*_CF_ = 12.8 Hz, ^4^*J*_CF_ = 3.6 Hz, C), 124.8 (CH), 124.6 (CH), 123.4 (d, ^3^*J*_CP_ = 12.7 Hz, C), 120.7 (d, ^1^*J*_CP_ = 112.5 Hz, C), 115.6 (d, ^2^*J*_CP_ = 13.2 Hz, CH), 111.3 (dd, ^2^*J*_CF_ = 21.0 Hz, ^4^*J*_CF_ = 3.5 Hz, CH), 103.9 (t, ^2^*J*_CF_ = 25.6 Hz, CH), 49.6 (d, ^4^*J*_CF_ = 2.1 Hz, CH), 45.4 (CH), 45.1 (CH), 31.3 (CH2); ^31^P {^1^H} NMR (162 MHz, CDCl_3_) δ (ppm): 29.2. ^19^F NMR (376 MHz, CDCl_3_) δ −111.36 (d, *J* = 7.4 Hz), −115.31 (d, *J* = 6.9 Hz). ESI-HRMS (CI) *m/z* calculated for C_34_H_27_F_2_NOP [M+H]^+^ 534.1798; found 534.1790.

**Diphenyl(6-(naphthalen-1-yl)-6,6a,7,11b-tetrahydro-5*H*-indeno[2,1-*c*]quinolin-2-yl)phosphine oxide (8g).** The general *procedure A* was followed using (4-aminophenyl)diphenylphosphine oxide **3** (1 mmol, 0.3 g) and 1-naphthaldehyde **4g** (1 mmol, 0.14 mL); after refluxing in CHCl_3_ 24 h, indene **6** (1.5 mmol) and BF_3_·Et_2_O were added and stirred for 48 h. The product was purified by column chromatography (silica gel, hexane:EtOAc 60:40), resulting in 0.17 g (31%) of a brown solid identified as **8g** with a mp of 291–294 °C. When the general *procedure B* was followed, 0.30 g (55%) of compound **8g** was obtained. ^1^H-NMR (400 MHz, CDCl_3_) δ (ppm): 8.11–8.08 (m, 1H), 7.93–7.91 (m, 1H), 7.87–7.84 (m, 2H), 7.71–7.63 (m, 4H), 7.60–7.50 (m, 6H), 7.47–7.42 (m, 4H), 7.15 (d, *J* = 7.4 Hz, 1H), 7.08–6.96 (m, 3H), 6.70 (dd, *J* = 8.3, 2.7 Hz, 1H), 5.61 (d, *J* = 2.4 Hz, 1H), 4.63 (d, *J* = 8.0 Hz, 1H), 4.25 (s, 1H), 3.51–3.45 (m, 1H), 3.29–3.23 (m, 1H), 2.19 (dd, *J* = 15.7, 7.8 Hz, 1H), 1.61 (s, 1H); ^13^C {^1^H} NMR (75 MHz, CDCl_3_) δ (ppm): 149.0 (d, ^4^*J*_CP_ = 1.9 Hz, C), 145.7 (C), 142.2 (C), 137.1 (C), 134.3 (d, ^2^*J*_CP_ = 11.4 Hz, CH), 133.8 (C), 133.3 (d, ^1^*J*_CP_ = 103.1 Hz, 2C), 132.2 (d, ^3^*J*_CP_ = 9.2 Hz, 2CH), 132.1 (d, ^3^*J*_CP_ = 9.6 Hz, 2CH), 131.5 (d, ^4^*J*_CP_ = 3.1 Hz, CH), 131.5 (d, ^4^*J*_CP_ = 3.0 Hz, CH), 130.7 (d, ^2^*J*_CP_ = 11.0 Hz, CH), 130.4 (C), 129.1 (CH), 128.3 (d, ^2^*J*_CP_ = 12.1 Hz, 2CH), 128.3 (d, ^2^*J*_CP_ = 11.9 Hz, 2CH), 128.1 (CH), 127.0 (CH), 126.5 (CH), 126.4 (CH), 125.8 (CH), 125.4 (CH), 124.8 (CH), 124.7 (d, ^3^*J*_CP_ = 17.1 Hz, CH), 124.0 (d, ^3^*J*_CP_ = 12.7 Hz, C), 122.7 (CH), 122.2 (CH), 120.5 (d, ^1^*J*_CP_ = 112.7 Hz, C), 115.6 (d, ^3^*J*_CP_ = 13.2 Hz, CH), 52.8 (CH), 45.7 (CH), 45.5 (CH), 31.6 (CH2); ^31^P {^1^H} NMR (162 MHz, CDCl_3_) δ (ppm): 29.2. ESI-HRMS (CI) *m/z* calculated for C_38_H_31_NOP [M+H]^+^ 548.2143; found 548.2134. 

**Diphenyl(6-(4-nitrophenyl)-6,6a,7,11b-tetrahydro-5*H*-indeno[2,1-*c*]quinolin-2-yl)phosphine oxide (8k).** The general *procedure A* was followed using (4-aminophenyl)diphenylphosphine oxide **3** (1 mmol, 0.3 g) and 4-nitrobenzaldehyde **4k** (1 mmol, 0.15 mL); after refluxing in CHCl_3_ 24 h, indene **6** (1.5 mmol) and BF_3_·Et_2_O were added and stirred for 48 h. The product was purified by column chromatography (silica gel, hexane:EtOAc 60:40), resulting in 0.17 g (31%) of a brown solid identified as **8k** with a mp of 219–222 °C. When the general *procedure B* was followed, 0.21 g (39%) of compound **8k** was obtained. ^1^H-NMR (400 MHz, CDCl_3_) δ (ppm): 8.27–8.23 (m, 2H), 7.65–7.49 (m, 9H), 7.45–7.38 (m, 4H), 7.21–7.16 (m, 1H), 7.13–7.07 (m, 2H), 7.04–7.00 (m, 2H), 6.65 (dd, *hJ* = 8.3, 2.7 Hz, 1H), 4.88 (d, *J* = 2.5 Hz,1H), 4.50–4.46 (m, 2H), 3.22–3.12 (m, 2H), 2.36–2.27 (m, 1H); ^13^C {^1^H} NMR (75 MHz, CDCl_3_) δ (ppm): 149.2 (C), 147.8 (d, ^4^*J*_CP_ = 2.6 Hz, C), 147.4 (C), 145.2 (C), 141.6 (C), 134.3 (d, ^2^*J*_CP_ = 11.4 Hz, CH), 133.0 (d, ^1^*J*_CP_ = 104.9 Hz, C), 132.9 (d, ^1^*J*_CP_ = 104.2 Hz, C), 132.1 (d, ^3^*J*_CP_ = 10.1 Hz, 2CH), 132.0 (d, ^3^*J*_CP_ = 10.2 Hz, 2CH), 131.6 (d, ^4^*J*_CP_ = 5.1 Hz, CH), 131.6 (d, ^4^*J*_CP_ = 5.1 Hz, CH), 130.8 (d, ^2^*J*_CP_ = 10.9 Hz, CH), 128.3 (d, ^2^*J*_CP_ = 12.0 Hz, 2CH), 128.3 (d, ^2^*J*_CP_ = 12.1 Hz, 2CH), 127.3 (2CH), 127.2 (CH), 126.7 (CH), 124.8 (CH), 124.7 (CH), 123.9 (2CH), 123.2 (d, ^3^*J*_CP_ = 12.7 Hz, C), 121.1 (d, ^1^*J*_CP_ = 111.7 Hz, C), 115.6 (d, ^3^*J*_CP_ = 13.1 Hz, CH), 56.8 (CH), 47.1 (CH), 45.5 (CH), 31.1 (CH2); ^31^P {^1^H} NMR (162 MHz, CDCl_3_) δ (ppm): 29.1. ESI-HRMS (CI) *m/z* calculated for C_34_H_28_N_2_O_3_P [M+H]^+^ 543.1838; found 543.1828.

**Diphenyl(6-phenyl-7*H*-indeno[2,1-*c*]quinolin-2-yl)phosphine oxide (9a).** The general *procedure A* was followed using (4-aminophenyl)diphenylphosphine oxide **3** (1 mmol, 0.3 g) and benzaldehyde **4a** (1 mmol, 0.1 mL); after refluxing in CHCl_3_ 24 h, indene **6** (1.5 mmol) and BF_3_·Et_2_O were added and stirred for 48 h. The product was purified by column chromatography (silica gel, hexane:EtOAc 30:70) resulting in 0.25g (50%) of a yellow solid identified as **9a** with a mp of 182–185 °C. When the general *procedure B* was followed, 0.17 g (34%) of compound **9a** was obtained. ^1^H-NMR (400 MHz, CDCl_3_) δ (ppm): 9.18 (dd, *J* = 13.9, 1.7 Hz, 1H), 8.35 (dd, *J* = 8.7, 2.9 Hz, 1H), 8.12 (dd, *J* = 7.1, 1.5 Hz, 1H), 7.96–7.93 (m, 2H), 7.92–7.87 (m, 1H), 7.82–7.77 (m, 4H), 7.67–7.63 (m, 2H), 7.63–7.61 (m, 1H), 7.61–7.59 (m, 1H), 7.58–7.57 (m, 1H), 7.56–7.55 (m, 2H), 7.54–7.53 (m, 2H), 7.52–7.51 (m, 1H), 7.47–7.43 (m, 2H), 4.19 (s, 2H); ^13^C {^1^H} NMR (75 MHz, CDCl_3_) δ (ppm): 158.4 (C), 149.4 (d, ^4^*J*_CP_ = 2.5 Hz, C), 146.6 (C), 145.0 (C), 139.9 (d, ^4^*J*_CP_ = 2.8 Hz, C), 135.3 (C), 132.5 (d, ^1^*J*_CP_ = 103.6 Hz, 2C), 132.3 (d, ^3^*J*_CP_ = 10.0 Hz, 4CH), 132.1 (d, ^4^*J*_CP_ = 2.6 Hz, 2CH), 130.9 (d, ^2^*J*_CP_ = 12.0 Hz, CH), 130.3 (d, ^1^*J*_CP_ = 104.4 Hz, C), 130.0 (d, ^2^*J*_CP_ = 10.7 Hz, CH), 129.8 (d, ^3^*J*_CP_ = 10.5 Hz, CH), 129.2 (CH), 128.8 (2CH), 128.7 (d, ^2^*J*_CP_ = 12.2 Hz, 4CH), 128.7 (CH), 128.6 (2CH), 127.5 (CH), 125.1 (CH), 124.6 (CH), 123.2 (C), 123.1 (C), 37.8 (CH_2_); ^31^P {^1^H} NMR (162 MHz, CDCl_3_) δ (ppm): 29.4. ESI-HRMS (CI) *m/z* calculated for C_34_H_25_NOP [M+H]^+^ 494.1674; found 494.1667

**Diphenyl(6-(4-(trifluoromethyl)phenyl)-7*H*-indeno[2,1-*c*]quinolin-2-yl)phosphine oxide (9b).** The general *procedure B* was followed using (4-aminophenyl)diphenylphosphine oxide **3** (1 mmol, 0.3 g), 4-(trifluoromethyl)benzaldehyde **4b** (1 mmol, 0.11 mL), indene **6** (1.5 mmol), and BF_3_·Et_2_O and stirred for 48h at reflux in CHCl_3_. The product was purified by column chromatography (silica gel, hexane:EtOAc 30:70) resulting in 0.15 g (26%) of a yellow solid identified as **9b** with a mp of 201–203 °C. ^1^H-NMR (400 MHz, CDCl_3_) δ (ppm): 9.20 (dd, *J* = 13.8, 1.7 Hz, 1H), 8.34 (dd, *J* = 8.7, 2.9 Hz, 1H), 8.14 (d, *J* = 7.6Hz, 1H), 8.07 (d, *J* = 8.3 Hz, 2H), 7.94–7.89 (m, 1H), 7.85–7.82 (m, 3H), 7.80–7.77 (m, 3H), 7.67–7.60 (m, 3H), 7.56–7.51 (m, 4H), 7.50–7.42 (m, 2H), 4.17 (s, 2H); ^13^C {^1^H} NMR (75 MHz, CDCl_3_) δ (ppm): 156.7 (C), 149.3 (d, ^4^*J*_CP_ = 2.8 Hz, C), 147.1 (C), 144.0 (C), 143.3 (q, ^5^*J*_CF_ = 1.1 Hz, C), 139.7 (C), 135.0 (d, ^4^*J*_CP_ = 1.1 Hz, C), 132.2 (d, ^1^*J*_CP_ = 104.6 Hz, 2C), 132.2 (d, ^3^*J*_CP_ = 9.9 Hz, 4CH), 132.2 (d, ^4^*J*_CP_ = 2.8 Hz, 2CH), 131.1 (q, ^2^*J*_CF_ = 32.6 Hz, C), 131.0 (d, ^1^*J*_CP_ = 103.0 Hz, C), 131.0 (d, ^2^*J*_CP_ = 12.0 Hz, CH), 130.0 (d, ^2^*J*_CP_ = 10.5 Hz, CH), 130.0 (d, ^3^*J*_CP_ = 10.5 Hz, CH), 129.2 (2CH), 129.0 (CH), 128.7 (d, ^2^*J*_CP_ = 12.2 Hz, 4CH), 127.7 (CH), 125.7 (q, ^3^*J*_CF_ = 3.7 Hz, 2CH), 125.2 (CH), 124.7 (CH), 123.7 (q, ^1^*J*_CF_ = 272.9 Hz, CF3), 123.3 (d, ^3^*J*_CP_ = 13.8 Hz, C), 36.7 (CH2); ^31^P {^1^H} NMR (162 MHz, CDCl_3_) δ (ppm): 29.2. ^19^F NMR (376 MHz, CDCl_3_) δ −62.6. ESI-HRMS (CI) *m/z* calculated for C_35_H_24_F_3_NOP [M+H]^+^ 562.1548; found 562.1535.

**Diphenyl(6-(3-fluorophenyl)-7*H*-indeno[2,1-*c*]quinolin-2-yl)phosphine oxide (9d).** The general *procedure A* was followed using (4-aminophenyl)diphenylphosphine oxide **3** (1 mmol, 0.3 g) and 3-fluorobenzaldehyde **4d** (1 mmol, 0.11 mL); after refluxing in CHCl_3_ 24 h, indene **6** (1.5 mmol) and BF_3_·Et_2_O were added and stirred for 48 h. The product was purified by column chromatography (silica gel, hexane:EtOAc 40:60) resulting in 0.24 g (47%) of a yellow solid identified as **9d** with a mp of 119–122 °C. When the general *procedure B* was followed, 0.15 g (29%) of compound **9d** was obtained. ^1^H-NMR (400 MHz, CDCl_3_) δ (ppm): 9.19 (dd, *J* = 14.1, 1.7 Hz, 1H), 8.34 (dd, *J* = 8.6, 2.8 Hz, 1H), 8.12 (d, *J* = 7.6 Hz, 1H), 7.93–7.88 (m, 1H), 7.82–7.77 (m, 4H), 7.76–7.74 (m, 1H), 7.70–7.63 (m, 3H), 7.63–7.60 (m, 2H), 7.56–7.53 (m, 4H), 7.52–7.51 (m, 1H) 7.50–7.42 (m, 4H), 7.24–7.19 (m, 1H), 4.19 (s, 2H); ^13^C {^1^H} NMR (75 MHz, CDCl_3_) δ (ppm): 162.9 (d, ^1^*J*_CF_ = 246.7 Hz, CF), 156.7 (d, ^4^*J*_CF_ = 2.4 Hz, C), 149.2 (d, ^4^*J*_CP_ = 2.6 Hz, C), 146.9 (C), 144.8 (C), 142.0 (d, ^3^*J*_CF_ = 7.3 Hz, C), 139.7 (C), 135.0 (C), 132.3 (d, ^1^*J*_CP_ = 104.6 Hz, 2C), 132.2 (d, ^3^*J*_CP_ = 9.8 Hz, 4CH), 132.2 (d, ^4^*J*_CP_ = 2.8 Hz, 2CH), 130.6 (d, ^1^*J*_CP_ = 104.2 Hz, C), 130.8 (d, ^2^*J*_CP_ = 12.0 Hz, CH), 130.2 (d, ^3^*J*_CF_ = 8.3 Hz, CH), 130.0 (d, ^3^*J*_CP_ = 10.5 Hz, CH), 129.9 (d, ^2^*J*_CP_ = 10.7 Hz, CH), 128.9 (CH), 128.7 (d, ^2^*J*_CP_ = 12.1 Hz, 4CH), 127.6 (CH), 125.1 (CH), 124.6 (CH), 124.5 (d, ^4^*J*_CF_ = 2.9 Hz, CH), 123.2 (d, ^3^*J*_CP_ = 14.1 Hz, C), 116.1 (d, ^2^*J*_CF_ = 21.3 Hz, CH), 115.9 (d, ^2^*J*_CF_ = 22.5 Hz, CH), 37.7 (CH2); ^31^P {^1^H} NMR (162 MHz, CDCl_3_) δ (ppm): 29.3. ^19^F NMR (376 MHz, CDCl_3_) δ −112.4. ESI-HRMS (CI) *m/z* calculated for C_34_H_27_FNOP [M+H]^+^ 512.1580; found 512.1570.

**Diphenyl(6-(2,4-difluorophenyl)-7*H*-indeno[2,1-*c*]quinolin-2-yl)phosphine oxide (9f).** The general *procedure A* was followed using (4-aminophenyl)diphenylphosphine oxide **3** (1 mmol, 0.3 g) and 2,4-difluorobenzaldehyde **4f** (1 mmol, 0.1 mL); after refluxing in CHCl_3_ 24 h, indene **6** (1.5 mmol) and BF_3_·Et_2_O were added and stirred for 48 h. The product was purified by column chromatography (silica gel, hexane:EtOAc 45:55) resulting in 0.22 g (42%) of a yellow solid identified as **9f** with a mp of 98–101 °C. When the general *procedure B* was followed, 0.07 g (13%) of compound **9f** was obtained. ^1^H-NMR (400 MHz, CDCl_3_) δ (ppm): 9.20 (dd, *J* = 13.9, 1.6 Hz, 1H), 8.33 (dd, *J* = 8.7, 2.9 Hz, 1H), 8.12 (d, *J* = 6.9 Hz, 1H), 7.93–7.88 (m, 1H), 7.81–7.76 (m, 4H), 7.70–7.65 (m, 1H), 7.64–7.60 (m, 3H), 7.55–7.51 (m, 4H), 7.47–7.43 (m, 2H), 7.11–6.99 (m, 2H) 4.01 (s, 2H); ^13^C {^1^H} NMR (75 MHz, CDCl_3_) δ (ppm): 163.4 (dd, ^1^*J*_CF_ = 251.2 Hz, ^3^*J*_CF_ = 11.8 Hz, CF), 159.9 (dd, ^1^*J*_CF_ = 250.7 Hz, ^3^*J*_CF_ = 12.1 Hz, CF), 153.5 (C), 149.0 (d, ^4^*J*_CP_ = 2.6 Hz, C), 146.0 (C), 144.8 (C), 139.5 (C), 136.6 (C), 132.4 (dd, ^3^*J*_CF_ = 5.1, 4.9 Hz, C), 132.1 (d, ^1^*J*_CP_ = 104.7 Hz, 2C), 132.0 (d, ^3^*J*_CP_ = 9.9 Hz, 4CH), 132.0 (d, ^4^*J*_CP_ = 2.7 Hz, 2CH), 130.8 (d, ^1^*J*_CP_ = 103.1 Hz, C), 130.7 (d, ^2^*J*_CP_ = 12.0 Hz, CH), 129.9 (d, ^3^*J*_CP_ = 10.2 Hz, CH), 129.7 (d, ^2^*J*_CP_ = 10.7 Hz, CH), 128.7 (CH), 128.5 (d, ^2^*J*_CP_ = 12.2 Hz, 4CH), 127.3 (CH), 125.0 (CH), 124.5 (CH), 123.8 (dd, ^2^*J*_CF_ = 15.9 Hz, ^4^*J*_CF_ = 3.8 Hz, C), 123.1 (d, ^3^*J*_CP_ = 13.9 Hz, C), 111.9 (dd, ^2^*J*_CF_ = 21.3 Hz, ^4^*J*_CF_ = 3.5 Hz, C), 104.2 (t, ^2^*J*_CF_ = 25.7 Hz, CH), 36.2 (d, ^5^*J*_CF_ = 6.3 Hz CH2); ^31^P {^1^H} NMR (162 MHz, CDCl_3_) δ (ppm): 29.4. ^19^F NMR (376 MHz, CDCl_3_) δ −108.1 (d, *J* = 8.4 Hz), −109.2 (d, *J* = 8.4 Hz). ESI-HRMS (CI) *m/z* calculated for C_34_H_23_F_2_NOP [M+H]^+^ 530.1485; found 530.1476.

**Diphenyl(6-(naphthalen-1-yl)-7*H*-indeno[2,1-*c*]quinolin-2-yl)phosphine oxide (9g).** The general *procedure A* was followed using (4-aminophenyl)diphenylphosphine oxide **3** (1 mmol, 0.3 g) and 1-naphthaldehyde **4g** (1 mmol, 0.14 mL); after refluxing in CHCl_3_ 24 h, indene **6** (1.5 mmol) and BF_3_·Et_2_O were added and stirred for 48 h. The product was purified by column chromatography (silica gel, hexane:EtOAc 30:70) resulting in 0.30 g (56%) of a brown solid identified as **9g** with a mp of 223–225 °C. When the general *procedure B* was followed, 0.17 g (31%) of compound **9g** was obtained. ^1^H-NMR (400 MHz, CDCl_3_) δ (ppm): 9.28 (dd, *J* = 13.9, 1.7 Hz, 1H), 8.37 (dd, *J* = 8.6, 3.0 Hz, 1H), 8.20–8.18 (m, 1H), 7.98 (dd, *J* = 17.5, 7.9 Hz, 2H), 7.90 (t, *J* = 9.3 Hz, 1H), 7.84–7.79 (m, 4H), 7.67–7.61 (m, 4H), 7.56–7.48 (m, 7H), 7.44–7.42 (m, 2H), 7.37–7.34 (m, 1H), 3.79 (s, 2H); ^13^C {^1^H} NMR (75 MHz, CDCl_3_) δ (ppm): 159.1 (C), 149.2 (d, ^4^*J* = 2.6 Hz, C), 146.0 (C), 145.1 (2C), 140.0 (C), 137.3 (d, ^4^*J* = 4.6 Hz, CH), 133.8 (C), 132.6 (d, ^1^*J* = 104.7 Hz, 2C), 132.3 (2CH), 132.2 (3CH), 132.2 (CH), 131.0 (d, ^2^*J* = 11.8 Hz, CH), 130.5 (d, ^1^*J* = 103.5 Hz, C), 130.1 (d, ^2^*J* = 10.9 Hz, CH), 129.9 (d, ^2^*J* = 10.9 Hz, CH), 129.2 (CH), 128.8 (CH), 128.8 (2CH), 128.7 (2CH), 128.5 (CH), 127.6 (CH), 126.6 (CH), 126.6 (CH), 126.1 (CH), 125.5 (CH), 125.5 (CH), 125.3 (CH), 124.8 (CH), 123.4 (d, ^3^*J* = 13.9Hz, 1C), 36.9 (CH2);^31^P {^1^H} NMR (162 MHz, CDCl_3_) δ (ppm): 29.2. ESI-HRMS (CI) *m/z* calculated for C_38_H_27_NOP [M+H]^+^ 544.1830; found 544.1819. 

**Diphenyl(6-(4-methoxyphenyl)-7*H*-indeno[2,1-*c*]quinolin-2-yl)phosphine oxide (9h).** The general *procedure A* was followed using (4-aminophenyl)diphenylphosphine oxide **3** (1 mmol, 0.3 g) and 4-methoxybenzaldehyde **4h** (1 mmol, 0.12 mL); after refluxing in CHCl_3_ 24 h, indene **6** (1.5 mmol) and BF_3_·Et_2_O were added and stirred for 48 h. The product was purified by column chromatography (silica gel, hexane:EtOAc 30:70) resulting in 0.40 g (77%) of a yellow solid identified as **9h** with a mp of 97–100 °C. ^1^H-NMR (400 MHz, CDCl_3_) δ (ppm): 9.14 (d, *J* = 14.3, 1H), 8.32 (dd, *J* = 8.7, 3.0 Hz, 1H), 8.10 (d, *J* = 7.5 Hz, 1H), 7.97–7.95 (m, 2H), 7.90–7.85 (m, 1H), 7.82–7.76 (m, 4H), 7.68–7.59 (m, 3H), 7.55–7.51 (m, 4H), 7.46–7.42 (m, 2H), 7.11–7.07 (m, 2H), 4.20 (s, 2H), 3.91 (s, 3H); ^13^C {^1^H} NMR (75 MHz, CDCl_3_) δ (ppm): 160.5 (CO), 157.9 (C), 149.4 (d, ^4^*J*_CP_ = 2.5 Hz, C), 146.5 (C), 145.0 (C), 140.0 (C), 135.1 (C), 133.4 (d, ^1^*J*_CP_ = 104.4 Hz, 2C), 132.4 (C), 132.3 (d, ^3^*J*_CP_ = 9.9 Hz, 4CH), 132.1 (d, ^4^*J*_CP_ = 2.6 Hz, 2CH), 130.8 (d, ^2^*J*_CP_ = 12.1 Hz, CH), 130.3 (2CH), 130.0 (d, ^3^*J*_CP_ = 10.3 Hz, CH), 129.7 (d, ^2^*J*_CP_ = 10.8 Hz, CH), 128.8 (d, ^1^*J*_CP_ = 101.6 Hz, C), 128.7 (d, ^2^*J*_CP_ = 12.4 Hz, 4CH), 127.5 (CH), 125.0 (CH), 124.6 (CH), 123.0 (d, ^3^*J*_CP_ = 14.0 Hz, C), 114.2 (CH), 114.1 (2CH), 55.4 (CH3), 38.0 (CH2); ^31^P {^1^H} NMR (162 MHz, CDCl_3_) δ (ppm): 29.4. ESI-HRMS (CI) *m/z* calculated for C_35_H_27_NO_2_P [M+H]^+^ 524.1779; found 524.1773. 

**Diphenyl(6-(3-methoxyphenyl)-7*H*-indeno[2,1-*c*]quinolin-2-yl)phosphine oxide (9i).** The general *procedure A* was followed using (4-aminophenyl)diphenylphosphine oxide **3** (1 mmol, 0.3 g) and 3-methoxybenzaldehyde **4i** (1 mmol, 0.12 mL); after refluxing in CHCl_3_ 24 h, indene **6** (1.5 mmol) and BF_3_·Et_2_O were added and stirred for 48 h. The product was purified by column chromatography (silica gel, hexane:EtOAc 40:60) resulting in 0.30 g (57%) of a yellow solid identified as **9i** with a mp of 124–127 °C. When the general *procedure B* was followed, 0.19 g (36%) of compound **9i** was obtained. ^1^H-NMR (400 MHz, CDCl_3_) δ (ppm): 9.18 (dd, *J* = 14.0, 1.6 Hz, 1H), 8.35 (dd, *J* = 8.6, 2.7 Hz, 1H), 8.12 (d, *J* = 7.2Hz, 1H), 7.92–7.87 (m, 1H), 7.82–7.77 (m, 4H), 7.67–7.64 (m, 1H), 7.63–7.60 (m, 2H), 7.56–7.49 (m, 6H), 7.48–7.47 (m, 2H), 7.46–7.44 (m, 1H), 7.08–7.05 (m, 1H), 4.19 (s, 2H), 3.92 (s, 3H); ^13^C {^1^H} NMR (75 MHz, CDCl_3_) δ (ppm): 159.8 (CO), 158.2 (C), 149.3 (d, ^4^*J*_CP_ = 2.9 Hz, C), 146.6 (C), 145.0 (C), 141.2 (C), 139.9 (C), 135.3 (d, ^4^*J*_CP_ = 1.1 Hz, C), 132.3 (d, ^1^*J*_CP_ = 104.8 Hz, 2C), 132.2 (d, ^3^*J*_CP_ = 10.1 Hz, 4CH), 132.2 (d, ^4^*J*_CP_ = 2.9 Hz, 2CH), 130.9 (d, ^2^*J*_CP_ = 12.0 Hz, CH), 130.8 (d, ^1^*J*_CP_ = 104.0 Hz, C), 130.0 (d, ^3^*J*_CP_ = 10.2 Hz, CH), 129.8 (d, ^2^*J*_CP_ = 10.7 Hz, CH), 129.7 (CH), 128.7 (d, ^2^*J*_CP_ = 12.1 Hz, 4CH), 128.7 (CH), 127.5 (CH), 125.1 (CH), 124.6 (CH), 123.1 (d, ^3^*J*_CP_ = 13.8 Hz, C), 121.2 (CH), 114.9 (CH), 114.4 (CH), 55.4 (OCH3), 37.8 (CH2); ^31^P {^1^H} NMR (162 MHz, CDCl_3_) δ (ppm): 29.5. ESI-HRMS (CI) *m/z* calculated for C_35_H_27_NO_2_P [M+H]^+^ 524.1779; found 524.1767.

**Diphenyl(6-(4-bromophenyl)-7*H*-indeno[2,1-*c*]quinolin-2-yl)phosphine oxide (9j).** The general *procedure B* was followed using (4-aminophenyl)diphenylphosphine oxide **3** (1 mmol, 0.3 g), 4-bromobenzaldehyde **4i** (1 mmol, 0.19 mL), indene **6** (1.5 mmol), and BF_3_·Et_2_O and stirred for 48 h at reflux in CHCl_3_. The product was purified by column chromatography (silica gel, hexane:EtOAc 30:70) resulting in 0.40 g (69%) of a yellow solid identified as **9j** with a mp of 178–181 °C. ^1^H-NMR (400 MHz, CDCl_3_) δ (ppm): 8.99 (d, *J* = 14.0 Hz, 1H), 8.16 (dd, *J* = 8.6 Hz, *J* = 2.8 Hz, 1H), 7.92 (d, *J* = 7.7 Hz, 1H), 7.80–7.75 (m, 1H), 7.72–7.65 (m, 3H), 7.57–7.55 (m, 2H), 7.51–7.47 (m, 3H), 7.41–7.38 (m, 3H), 7.34–7.25 (m, 4H), 6.92 (d, *J* = 8.1 Hz, 1H), 3.98 (s, 2H); ^13^C {^1^H} NMR (75 MHz, CDCl_3_) δ (ppm): 156.7 (C), 149.1 (d, ^4^*J* = 2.6 Hz, C), 146.7 (C), 144.7 (C), 139.5 (C), 138.5 (C), 134.8 (C), 132.5 (CH), 132.1 (d, ^4^*J* = 2.8 Hz, CH), 132.1 (CH), 132.0 (d, ^1^*J* = 93.6 Hz, 2C), 132.0 (CH), 131.7 (3CH), 131.5 (d, ^3^*J* = 6.4 Hz, CH), 130.7 (d, ^2^*J* = 12.0 Hz, CH), 130.4 (3CH), 130.3 (d, ^1^*J* = 104.1 Hz, C), 129.8 (d, ^3^*J* = 7.2 Hz, CH), 129.8 (d, ^2^*J* = 7.4 Hz, CH), 128.8 (CH), 128.7 (CH), 128.6 (CH), 128.3–128.1 (CH), 127.5 (CH), 125.0 (CH), 124.5 (CH), 123.7 (C), 122.9 (d, ^3^*J* = 14.1 Hz, 1C), 37.6 (CH2); ^31^P {^1^H} NMR (162 MHz, CDCl_3_) δ (ppm): 29.4. ESI-HRMS (CI) *m/z* calculated for C_34_H_24_BrNOP [M+H]^+^ 572.0779; found 574.0754.

Synthesis of 7*H*-indeno[2,1-*c*]quinolin-7-one phosphine oxide **10**

General procedure.

A mixture of tetrahydro-5*H*-indeno[2,1-*c*]quinolin-2-yl derivatives **8** or 7*H*-indeno[2,1-*c*]quinolin-2-yl phosphine oxide **9** (1 mmol) and SeO_2_ (6 mmol, 6 equiv.) was stirred and heated to reflux in dioxane for 48 h when TLC and ^1^H NMR analysis indicated the consumption of the starting materials. The resulting solution was washed with a saturated solution of NaHCO_3_ (50 mL) and with water (50 mL), extracted with dicloromethane (2 × 20 mL), and dried (MgSO_4_). Removal of the solvent under vacuum led to an oil that was purified by column chromatography on silica gel using 20:80 mixture of ethyl ether and EtOAc to produce the compounds **10**.

**Diphenyl(7-oxo-6-phenyl-7*H*-indeno[2,1-*c*]quinolin-2-yl)phosphine oxide (10a).** The general procedure was followed using diphenyl(6-phenyl-6,6a,7,11b-tetrahydro-5*H*-indeno[2,1-*c*]quinolin-2-yl)phosphine oxide **8a** (1 mmol, 0.50 g). The product was purified by column chromatography (silica gel, ethyl ether:EtOAc 20:80) resulting in 0.50 g (99%) of a yellow solid identified as **10a**, mp 252–253 °C. ^1^H-NMR (400 MHz, CDCl_3_) δ (ppm): 9.06 (dd, *J* = 13.7, 1.6 Hz, 1H), 8.24 (dd, *J* = 8.7, 2.6 Hz, 1H), 7.95–7.90 (m, 2H), 7.86–7.84 (m, 2H), 7.81–7.75 (m, 4H), 7.73–7.71 (m, 1H), 7.66–7.61 (m, 2H), 7.57–7.51 (m, 8H), 7.50–7.46 (m, 1H); ^13^C {^1^H} NMR (75 MHz, CDCl_3_) δ (ppm): 191.7 (CO), 158.9 (C), 154.3 (C), 152.8 (d, ^4^*J*_CP_ = 2.3 Hz, C), 140.9 (C), 137.2 (C), 134.8 (CH), 133.6 (C), 133.1 (d, ^2^*J*_CP_ = 10.8 Hz, CH), 132.4 (d, ^4^*J*_CP_ = 2.8 Hz, 2CH), 132.2 (d, ^1^*J*_CP_ = 102.1 Hz, C), 132.2 (d, ^2^*J*_CP_ = 10.0 Hz, 4CH), 131.9 (d, ^1^*J*_CP_ = 105.0 Hz, 2C), 131.6 (CH), 131.3 (d, ^3^*J*_CP_ = 3.1 Hz, CH), 131.2 (CH), 129.8 (2CH), 129.8 (CH), 128.8 (d, ^2^*J*_CP_ = 12.3 Hz, 4CH), 127.9 (2CH), 125.0 (CH), 124.5 (CH), 123.2 (C), 122.3 (d, ^3^*J*_CP_ = 13.9 Hz, C); ^31^P {^1^H} NMR (162 MHz, CDCl_3_) δ (ppm): 28.5. ESI-HRMS (CI) *m/z* calculated for C_34_H_22_NO_2_P [M+H]^+^ 508.1466; found 508.1477.

**Diphenyl(7-oxo-(6-(4-trifluoromethyl)phenyl)-7*H*-indeno[2,1-*c*]quinolin-2-yl)phosphine oxide (10b).** The general procedure was followed using diphenyl(6-(4-(trifluoromethyl)phenyl)-6,6a,7,11b-tetrahydro-5*H*-indeno[2,1-*c*]quinolin-2-yl)phosphine oxide **8b** (1 mmol, 0.57 g). The product was purified by column chromatography (silica gel, ethyl ether:EtOAc 20:80) resulting in 0.57 g (99%) of a yellow solid identified as **10b**, mp 217–219 °C. ^1^H-NMR (400 MHz, CDCl_3_) δ (ppm): 9.09 (dd, *J* = 13.7, 1.6 Hz, 1H), 8.23 (dd, *J* = 8.7, 2.8 Hz, 1H), 7.98–7.92 (m, 4H), 7.81–7.72 (m, 7H), 7.66–7.62 (m, 2H), 7.59–7.48 (m, 6H); ^13^C {^1^H} NMR (75 MHz, CDCl_3_) δ (ppm): 191.5 (CO), 157.1 (C), 154.5 (C), 152.7 (d, ^4^*J*_CP_ = 2.5 Hz, C), 140.9 (C), 140.6 (q, ^5^*J*_CF_ = 1.1 Hz, C), 135.0 (CH), 133.4 (d, ^2^*J*_CP_ = 10.4 Hz, CH), 133.3 (C), 132.5 (d, ^4^*J*_CP_ = 2.6 Hz, 2CH), 132.2 (d, ^3^*J*_CP_ = 9.9 Hz, 4CH), 131.8 (d, ^1^*J*_CP_ = 95.8 Hz, C), 131.8 (CH), 131.7 (d, ^1^*J*_CP_ = 105.0 Hz, 2C), 131.5 (q, ^2^*J*_CF_ = 32.6 Hz, CH), 131.3 (d, ^2^*J*_CP_ = 11.5 Hz, CH), 131.2 (d, ^3^*J*_CP_ = 9.5 Hz, CH), 130.3 (2CH), 128.9 (d, ^2^*J*_CP_ = 12.4 Hz, 4CH), 125.2 (CH), 124.9 (q, ^3^*J*_CF_ = 3.7 Hz, 2CH), 124.7 (CH), 124.1 (q, ^1^*J*_CF_ = 272.1 Hz, CF_3_), 123.2 (C), 122.5 (d, ^3^*J*_CP_ = 13.7 Hz, C); ^31^P {^1^H} NMR (162 MHz, CDCl_3_) δ (ppm): 28.4. ^19^F NMR (376 MHz, CDCl_3_) δ −62.5. ESI-HRMS (CI) *m/z* calculated for C_35_H_22_F_3_NO_2_P [M+H]^+^ 576.1340; found 576.1329.

**(6-(3-Fluorophenyl)-7-oxo-7*H*-indeno[2,1-*c*]quinolin-2-yl)diphenylphosphine oxide (10d).** The general procedure was followed using (6-(3-fluorophenyl)-7*H*-indeno[2,1-*c*]quinolin-2-yl)diphenylphosphine oxide **9d** (1 mmol, 0.56 g). The product was purified by column chromatography (silica gel, ethyl ether:EtOAc 20:80) resulting in 0.49 g (93%) of a yellow solid identified as **10d**, mp 234–237 °C. ^1^H-NMR (400 MHz, CDCl_3_) δ (ppm): 9.06 (dd, *J* = 13.7, 1.6 Hz, 1H), 8.23 (dd, *J* = 8.8, 2.8 Hz, 1H), 7.96–7.90 (m, 2H), 7.80–7.75 (m, 4H), 7.71 (dd, *J* = 7.3, 1.3 Hz, 1H), 7.66–7.61 (m, 3H), 7.57–7.46 (m, 8H), 7.22 (tdd, *J* = 8.4, 2.6, 1.0 Hz, 1H); ^13^C {^1^H} NMR (75 MHz, CDCl_3_) δ (ppm): 191.4 (CO), 162.4 (d, ^1^*J*_CF_ = 245.6 Hz, CF), 157.2 (d, ^4^*J*_CF_ = 2.4 Hz, C), 154.4 (C), 152.6 (d, ^4^*J*_CP_ = 2.5 Hz, C), 140.8 (C), 139.2 (d, ^3^*J*_CF_ = 7.7 Hz, C), 134.9 (CH), 133.5 (C), 133.2 (d, ^2^*J*_CP_ = 10.8 Hz, CH), 132.5 (d, ^1^*J*_CP_ = 101.8 Hz, C), 132.4 (d, ^4^*J*_CP_ = 2.6 Hz, 2CH), 132.1 (d, ^3^*J*_CP_ = 10.1 Hz, 4CH), 131.7 (d, ^1^*J*_CP_ = 105.3 Hz, CH), 131.7 (CH), 131.2 (d, ^2^*J*_CP_ = 11.6 Hz, CH), 131.2 (d, ^3^*J*_CP_ = 9.8 Hz, CH), 129.4 (d, ^3^*J*_CF_ = 8.0 Hz, CH), 128.8 (d, ^2^*J*_CP_ = 12.3 Hz, 4CH), 125.7 (d, ^4^*J*_CF_ = 2.9 Hz, CH), 125.1 (CH), 124.6 (CH), 123.1 (C), 122.3 (d, ^3^*J*_CP_ = 13.8 Hz, C), 116.9 (d, ^2^*J*_CF_ = 23.1 Hz, CH), 116.7 (d, ^2^*J*_CF_ = 2.1 Hz, CH); ^31^P {^1^H} NMR (162 MHz, CDCl_3_) δ (ppm): 28.3. ^19^F NMR (376 MHz, CDCl_3_) δ −111.5. ESI-HRMS (CI) *m/z* calculated for C_34_H_22_FNO_2_P [M+H]^+^ 526.1372; found 526.1360.

**(7-Oxo-(6-(4-trifluoromethyl)phenyl)-7*H*-indeno[2,1-c]quinolin-2-yl)diphenylphosphine oxide (10e).** The general procedure was followed using (6-(3,4-difluorophenyl)-6,6a,7,11b-tetrahydro-5*H*-indeno[2,1-*c*]quinolin-2-yl)diphenylphosphine oxide **8e** (1 mmol, 0.53 g). The product was purified by column chromatography (silica gel, ethyl ether:EtOAc 20:80) resulting in 0.43 g (79%) of a yellow solid identified as **10e**, mp 209–211 °C. ^1^H-NMR (400 MHz, CDCl_3_) δ (ppm): 9.07 (dd, *J* = 13.7, 1.4 Hz, 1H), 8.21 (dd, *J* = 8.7, 2.9 Hz, 1H), 7.96–7.91 (m, 2H), 7.81–7.73 (m, 6H), 7.67–7.62 (m, 3H), 7.58–7.48 (m, 6H), 7.34–7.27 (m, 1H);^13^C {^1^H} NMR (75 MHz, CDCl_3_) δ (ppm): 191.5 (CO), 156.2 (d, ^4^*J*_CF_ = 1.6 Hz, C), 154.6 (C), 152.6 (d, ^4^*J*_CP_ = 2.5 Hz, C), 151.6 (dd, ^1^*J*_CF_ = 251.8 Hz, ^2^*J*_CF_ =12.8 Hz, CF), 149.9 (dd, ^1^*J*_CF_ = 248.1 Hz, ^2^*J*_CF_ = 12.9 Hz, CF), 140.8 (C), 134.9 (CH), 134.0 (dd, ^3^*J*_CF_ = 6.0 Hz, ^4^*J*_CF_ =3.8 Hz, C), 133.4 (C), 133.3 (d, ^2^*J*_CP_ = 10.8 Hz, CH), 132.7 (d, ^1^*J*_CP_ = 100.6 Hz, C), 132.5 (d, ^4^*J*_CP_ = 2.9 Hz, 2CH), 132.1 (d, ^3^*J*_CP_ = 10.2 Hz, 4CH), 131.8 (CH), 131.7 (d, ^1^*J*_CP_ = 105.3 Hz, 2C), 131.2 (d, ^3^*J*_CP_ = 9.7 Hz, CH), 131.1 (d, ^2^*J*_CP_ = 11.6 Hz, CH), 128.8 (d, ^2^*J*_CP_ = 12.3 Hz, 4CH), 126.6 (dd, ^3^*J*_CF_ = 6.9 Hz, ^4^*J*_CF_ = 3.6 Hz, CH), 125.1 (CH), 124.6 (CH), 122.9 (d, ^4^*J*_CP_ = 1.0 Hz, C), 122.3 (d, ^3^*J*_CP_ = 13.5 Hz, C), 119.3 (dd, ^2^*J*_CF_ = 19.1 Hz, ^3^*J*_CF_ =1.1 Hz, CH), 116.7 (d, ^2^*J*_CF_ = 17.7 Hz, CH); ^31^P {^1^H} NMR (162 MHz, CDCl_3_) δ (ppm): 28.4. ^19^F NMR (376 MHz, CDCl_3_) δ −135.8 (d, *J* = 21.0 Hz, 1F), −138.1 (d, *J* = 21.4 Hz, 1F). ESI-HRMS (CI) *m/z* calculated for C_34_H_21_F_2_NO_2_P [M+H]^+^ 544.1278; found 544.1267.

**(6-(Naphth-1-yl)-7-oxo-7*H*-indeno[2,1-*c*]quinolin-2-yl)diphenylphosphine oxide (10g).** The general procedure was followed using (6-(naphthalen-1-yl)-7*H*-indeno[2,1-*c*]quinolin-2-yl)diphenylphosphine oxide **9g** (1 mmol, 0.54 g). The product was purified by column chromatography (silica gel, ethyl ether:EtOAc 20:80) resulting in 0.48 g (87%) of a yellow solid identified as **10g**, mp 271–272 °C. ^1^H-NMR (400 MHz, CDCl_3_) δ (ppm): 9.18 (d, *J* = 13.7 Hz, 1H), 8.28 (dd, *J* = 8.7, 2.9 Hz, 1H), 8.03–7.99 (m, 2H), 7.97–7.92 (m 2H), 7.83–7.78 (m, 4H), 7.66–7.62 (m, 3H), 7.61–7.54 (m, 7H), 7.50–7.44 (m, 3H), 7.35 (t, *J* = 7.7 Hz, 1H); ^13^C {^1^H} NMR (75 MHz, CDCl_3_) δ (ppm): 191.1 (CO), 158.3 (C), 153.5 (C), 152.7 (d, ^4^*J*_CP_ = 2.5 Hz, C), 141.1 (C), 135.2 (C), 134.8 (CH), 133.7 (C), 133.2 (d, ^2^*J*_CP_ = 10.9 Hz, CH), 133.0 (d, ^1^*J*_CP_ = 122.1 Hz, 2C), 132.5 (d, ^1^*J*_CP_ = 102.2 Hz, C), 132.5 (d, ^4^*J*_CP_ = 2.8 Hz, 2CH), 132.2 (d, ^3^*J*_CP_ = 10.0 Hz, 4CH), 131.7 (CH), 131.5 (C), 131.5 (C), 131.3 (d, ^2^*J*_CP_ = 9.6 Hz, CH), 131.2 (CH), 129.6 (2CH), 128.9 (d, ^2^*J*_CP_ = 12.4 Hz, 4CH), 128.5 (CH), 127.2 (CH), 126.4 (CH), 125.9 (CH), 125.1 (CH), 125.0 (d, ^3^*J*_CP_ = 3.2 Hz, CH), 124.7 (C), 124.5 (CH), 122.6 (d, ^3^*J*_CP_ = 13.7 Hz, C); ^31^P {^1^H} NMR (162 MHz, CDCl_3_) δ (ppm): 28.3. ESI-HRMS (CI) *m/z* calculated for C_38_H_25_NO_2_P [M+H]^+^ 558.1623; found 558.1612.

Biology

Materials

Reagents and solvents were used as purchased without further purification. Camptothecin was purchased from Merck. All stock solutions of the investigated compounds were prepared by dissolving the powdered materials in appropriate amounts of DMSO. The final concentration of DMSO never exceeded 10% (*v*/*v*) in the reactions. Under these conditions, DMSO was also used in the controls and was not seen to affect TOP1 activity. The stock solution was stored at 5 °C until it was used.

Expression and purification of human topoisomerase IB

The yeast *Saccaromyces cerevisiae* TOP1 null strain RS190, which was used for the expression of recombinant human TOP1, was a kind gift from R. Sternglanz (State University of New York, Stony Brook, NY, USA). Plasmid pHT143, for the expression of recombinant TOP1 under the control of an inducible GAL promoter, was described [64]. The plasmids pHT143 were transformed into the yeast *S. cerevisiae* strain RS190. The proteins were expressed and purified by affinity chromatography essentially as described [65]. The protein concentrations were estimated from Coomassie blue-stained SDS/polyacrylamide gels by comparison to serial dilutions of bovine serum albumin (BSA).

DNA relaxation assays

TOP1 activity was assayed using a DNA relaxation assay by incubating 110 ng/mL of TOP1 with 0.5 µg of negatively supercoiled pUC18 in 20 mL of reaction buffer (20 mM Tris HCl, 0.1 mM Na_2_EDTA, 10 mM MgCl_2_, 50 mg/mL acetylated BSA, and 150 mM KCl, pH 7.5). The effect of the synthesized phosphorylated derivatives **8**, **9**, and **10** on the TOP1 activity was measured by adding different concentrations of the compounds, at different time points, as indicated in the text. The reactions were performed at 37 °C stopped by the addition of 0.5% SDS after the indicated time intervals. The samples were protease-digested and electrophoresed in a horizontal 1% agarose gel in 1xTBE (50 mM Tris, 45 mM boric acid, 1 mM EDTA) at 25V during 18h. The gel was stained with gel red (BIOTIUM, 5 mg/mL), destained with water, and photographed under UV illumination. Since all drugs were dissolved in dimethyl sulfoxide (DMSO), a positive control sample containing the same DMSO concentration as the samples incubated with the drugs was included in all experiments. As a control for drug inhibition, the well-known TOP1-specific drug CPT was included.

Cytotoxicity assays

Cells were cultured according to the supplier’s instructions. Cells were seeded in 96-well plates at a density of 1.5–3 × 10^3^ cells per well and incubated overnight in 0.1 mL of media supplied with 10% Fetal Bovine Serum (Lonza) in a 5% CO_2_ incubator at 37 °C. On day 2, drugs were added and the samples were incubated for 48h. After treatment, 10 μL of the cell counting kit-8 was added into each well for an additional 2h incubation at 37 °C. The absorbance of each well was determined by an Automatic Elisa Reader System at 450 nm wavelength (deducting 620 nm). CPT was used as the positive control.

Computational assay—ADME properties

The physicochemical and pharmacokinetics properties of the tested compounds were calculated using swissADME [66] and pkCSM [67] online web servers. Chemical structures were imported in swissADME and pkCSM tools to calculate the molecular as well as the ADME properties of the compounds.

## 5. Conclusions

In summary, a series of novel tetrahydroquinoline derivatives **8** and quinoline derivatives **9**, with a phosphine oxide substituent in position 2, have been synthesized using a Povarov-type [4+2] cycloaddition reaction from phosphine oxide aldimines **5**, synthesized by a previous reaction of aniline **3** and aldehydes **4,** with indene **6**. In addition, compounds **8** and **9** were oxidated to produce the corresponding carbonyl derivatives **10**.

The synthesized derivatives were evaluated for biochemical activity as topoisomerase I inhibitors and cellular responses against four human cell lines including two cancerous cell lines (A549 and SKOV3) and an embryonic cell line (Hek293) and non-cancerous cells (MRC-5). The TOP1 inhibition assay shows that all studied derivatives show higher inhibitory activity than the reference compound (CPT) at 5 min. Moreover, biological studies suggest a different mechanism of action compared to CPT, as inhibition is observed even at 30 min, which may reflect an irreversible inhibition of TOP1.

Finally, the synthesized derivatives **8**, **9**, and **10** were screened for their antiproliferative activity and, in general, the newly synthesized phosphorated compounds exhibited no antiproliferative activity towards MCR-5 non-malignant lung fibroblasts, while some of them (**8a** (Ar = C_6_H_5_), **8b** (Ar = *p*-CF_3_C_6_H_4_), **8d** (Ar = *m*-FC_6_H_4_), **8e** (Ar = 3,4-F_2_C_6_H_3_), **8f** (Ar = 2,4-F_2_C_6_H_3_), **9i** (Ar = *m*-CH_3_OC_6_H_4_), and **9j** (Ar = *p*-BrC_6_H_4_)) showed cytotoxicity against SKOV3 and A549.

## Figures and Tables

**Table 1:** Diphenyl(tetrahydro-5*H*-indeno[2,1-*c*]quinolin-2-yl)phosphine oxides **8** and diphenyl(7*H*-indeno[2,1-*c*]quinolin-2-yl)phosphine oxides **9** obtained by stepwise and multicomponent routes.

Entry	Comp.	Ar.	Yield 8/9 (%) Stepwise Route ^a^	Yield 8/9 (%) Multicomponent Route ^a^
1	**8a**/**9a**	C_6_H_5_	31/50	53/34
2	**8b**/**9b**	*p*-CF_3_C_6_H_4_	-	57/26
3	**8c**/**9c**	*p*-FC_6_H_4_	-	47/35
4	**8d**/**9d**	*m*-FC_6_H_4_	0/27	37/29
5	**8e**/**9e**	3,4-F_2_C_6_H_3_	-	78/0
6	**8f**/**9f**	2,4-F_2_C_6_H_3_	3/42	71/13
7	**8g**/**9g**	1-naphthyl	31/56	55/31
8	**8k**	*p*-NO_2_C_6_H_4_	31/-	39/-
9	**9h**	*p*-CH_3_OC_6_H_4_	0/77	-
10	**9i**	*m*-CH_3_OC_6_H_4_	0/57	0/36
11	**9j**	*p*-BrC_6_H_4_	-	0/69

^a^ Isolated yield of compounds **8** and **9**.

**Table 2:** TOP1 inhibitory activity of diphenyl(tetrahydro-5*H*-indeno[2,1-*c*]quinolin-2-yl)phosphine oxides **8**, diphenyl(7*H*-indeno[2,1-*c*]quinolin-2-yl)phosphine oxides **9**, and diphenyl(7-oxo-7*H*-indeno[2,1-*c*]quinolin-2-yl)phosphine oxides **10**.

Entry	Comp.	Ar.	TOP1 Inhibition ^a^			
			1 min	3 min	5 min	30 min
1	CPT		++	−	−	−
2	**8a**	C_6_H_5_	++	++	++	++
3	**8b**	*p*-CF_3_C_6_H_4_	+++	+++	+++	+++
4	**8c**	*p*-FC_6_H_4_	++	++	++	++
5	**8d**	*m*-FC_6_H_4_	++	++	++	nd
6	**8e**	3,4-F_2_C_6_H_3_	++	++	++	nd
7	**8f**	2,4-F_2_C_6_H_3_	+	+	+	nd
8	**8g**	1-naphthyl	+	+	+	+
9	**8h**	*p*-NO_2_C_6_H_4_	+	+	+	+
10	**9a**	C_6_H_5_	+	++	++	nd
11	**9b**	*p*-CF_3_C_6_H_4_	+++	+++	+++	+++
12	**9c**	*p*-FC_6_H_4_	+	+	+	nd
13	**9d**	*m*-FC_6_H_4_	+	+	+	nd
14	**9f**	2,4-F_2_C_6_H_3_	++	++	++	nd
15	**9g**	1-naphthyl	++	++	++	++
16	**9h**	*p*-CH_3_OC_6_H_4_	++	++	++	nd
17	**9i**	*m*-CH_3_OC_6_H_4_	++	++	++	++
18	**9j**	*p*-BrC_6_H_4_	+	+	+	nd
19	**10a**	C_6_H_5_	+	++	++	++
20	**10b**	*p*-CF_3_C_6_H_4_	++	++	++	++
21	**10d**	*m*-FC_6_H_4_	+	+	+	nd
22	**10e**	3,4-F_2_C_6_H_3_	++	++	++	nd
23	**10g**	1-naphthyl	+	+	+	+

^a^ The activity of the compounds to inhibit TOP1 relaxation was expressed semiquantitatively as follows: −, no inhibition; +, weak inhibition; ++ similar activity to CPT inhibition; +++ strong inhibition; nd: no determined.

**Table 3:** Antiproliferative activity of tetrahydro-5*H*-indeno[2,1-*c*]quinolin-2-ylphosphine oxides **8**, 7*H*-indeno[2,1-*c*]quinolin-2-ylphosphine oxides **9**, and 7-oxo-7*H*-indeno[2,1-*c*]quinolinylphosphine oxides **10** derivatives.

Entry	Comp.	Ar.	IC_50_ (µM)			
			MRC5	A549	SKOV3	HEK293
**1**	CPT		0.11 ± 0.02	(1.0 ± 0.06)·10^−3^	(5.5 ± 0.01)·10^−3^	-
**2**	**8a**	C_6_H_5_	>50	40.18 ± 7.13	6.35 ± 1.73	-
**3**	**8b**	*p*-CF_3_C_6_H_4_	>50	25.82 ± 6.98	>50	>50
**4**	**8c**	*p*-FC_6_H_4_	10.71 ± 0.43	19.94 ± 3.35	>50	>50
**5**	**8d**	*m*-FC_6_H_4_	>50	43.10 ± 12.14	>50	>50
**6**	**8e**	3,4-F_2_C_6_H_3_	>50	>50	13.19 ± 2.79	>50
**7**	**8f**	2,4-F_2_C_6_H_3_	>50	>50	31.04 ± 3.17	>50
**8**	**8g**	1-naphthyl	>50	>50	>50	>50
**9**	**8h**	*p*-NO_2_C_6_H_4_	>50	>50	>50	>50
**10**	**9a**	C_6_H_5_	>50	>50	>50	>50
**11**	**9b**	*p*-CF_3_C_6_H_4_	>50	>50	>50	>50
**12**	**9c**	*p*-FC_6_H_4_	>50	>50	>50	>50
**13**	**9d**	*m*-FC_6_H_4_	>50	>50	>50	>50
**14**	**9f**	2,4-F_2_C_6_H_3_	>50	41.70 ± 7.25	>50	>50
**15**	**9g**	1-naphthyl	>50	>50	>50	>50
**16**	**9i**	*p*-CH_3_OC_6_H_4_	>50	>50	>50	>50
**17**	**9j**	*m*-CH_3_OC_6_H_4_	>50	42.26 ± 2.09	>50	>50
**18**	**9k**	*p*-BrC_6_H_4_	>50	>50	>50	>50
**19**	**10a**	C_6_H_5_	>50	>50	>50	>50
**20**	**10b**	*p*-CF_3_C_6_H_4_	>50	>50	>50	>50
**21**	**10d**	*m*-FC_6_H_4_	>50	>50	>50	>50
**22**	**10e**	3,4-F_2_C_6_H_3_	>50	>50	>50	>50
**23**	**10g**	1-naphthyl	>50	>50	>50	>50

**Table 4:** Physicochemical properties and drug likeliness for tetrahydro-5*H*-indeno[2,1-*c*]quinolin-2-ylphosphine oxides **8**, 7*H*-indeno[2,1-*c*]quinolin-2-ylphosphine oxides **9**, and 7-oxo-7*H*-indeno[2,1-*c*]quinolinylphosphine oxides **10** derivatives.

Entry	Comp.	M.W (g/mol)	Cons. LogP_o/w_	TPSA (Å)	Lipinski	Veber	HBA/HBD	RB
**1**	**CPT**	348.35	2.20	81.42	0 viol.	Yes	5/1	1
**2**	**8a**	497.57	5.98	38.91	1 viol. ^a^	Yes	1/1	4
**3**	**8b**	565.56	7.04	38.91	2 viol. ^b^	Yes	4/1	5
**4**	**8c**	515.56	6.29	38.91	2 viol. ^b^	Yes	2/1	4
**5**	**8d**	515.56	6.29	38.91	2 viol. ^b^	Yes	2/1	4
**6**	**8e**	533.55	6.61	38.91	2 viol. ^b^	Yes	3/1	4
**7**	**8f**	533.55	6.61	38.91	2 viol. ^b^	Yes	3/1	4
**8**	**8g**	547.62	6.84	38.91	2 viol. ^b^	Yes	1/1	4
**9**	**8k**	542.56	5.23	84.73	2 viol. ^b^	Yes	3/1	5
**10**	**9a**	493.53	6.53	39.77	1 viol. ^a^	Yes	2/0	4
**11**	**9b**	561.53	7.59	39.77	2 viol. ^b^	Yes	5/0	5
**12**	**9c**	511.52	6.83	39.77	2 viol. ^b^	Yes	3/0	4
**13**	**9d**	511.52	6.83	39.77	2 viol. ^b^	Yes	3/0	4
**14**	**9f**	529.52	7.12	39.77	2 viol.^b^	Yes	4/0	4
**15**	**9g**	543.59	7.38	39.77	2 viol. ^b^	Yes	2/0	4
**16**	**9h**	523.56	6.56	49.00	2 viol. ^b^	Yes	3/0	5
**17**	**9i**	523.56	6.54	49.00	2 viol. ^b^	Yes	3/0	5
**18**	**9j**	572.43	7.13	39.77	2 viol. ^b^	Yes	2/0	4
**19**	**10a**	507.52	6.04	56.84	2 viol. ^b^	Yes	3/0	4
**20**	**10b**	575.52	7.07	56.84	2 viol. ^b^	Yes	6/0	5
**21**	**10d**	525.51	6.40	56.84	2 viol. ^b^	Yes	4/0	4
**22**	**10e**	547.53	6.05	55.98	2 viol. ^b^	Yes	4/1	4
**23**	**10g**	557.58	6.90	56.84	2 viol. ^b^	Yes	3/0	4

^a^ MLOGP > 4.15; ^b^ MW > 500, MLOGP > 4.15.

## Data Availability

Data are contained within the article and Supplementary Materials.

## Supplementary Materials

The following supporting information can be downloaded at: https://www.mdpi.com/article/10.3390/molecules29245992/s1, Table S1: Antiproliferative activity of tetrahydro-5*H*-indeno[2,1-*c*]quinolin-2-ylphosphine oxides **8**, 7*H*-indeno[2,1-*c*]quinolin-2-ylphosphine oxides **9**, and 7-oxo-7*H*-indeno[2,1-*c*]quinolinylphosphine oxides **10** derivatives.
