# Supplementary material for: Phosphine Oxide Indenoquinoline Derivatives: Synthesis and Biological Evaluation as Topoisomerase I Inhibitors and Antiproliferative Agents
Source: Molecules. 2024 Dec 19;29(24):5992. doi: 10.3390/molecules29245992 (PMC11678328; doi:10.3390/molecules29245992)
Supplement: Supplementary file 1 [file molecules-29-05992-s001.zip › molecules-3358271-supplementary.pdf]

Supplementary information for:

Phosphine Oxide Indenoquinoline Derivatives: Synthesis and Biological Evaluation as Topoisomerase I Inhibitors and Antiproliferative agents.

Alba Rodriguez-Paniagua 1, Cinzia Tesauro 2, Birgitta R. Knudsen 2, Maria Fuertes 1,* and Concepción Alonso 1,*

1 Departamento de Química Orgánica I, Facultad de Farmacia and Centro de Investigación Lascaray (Lascaray Research Center), Universidad del País Vasco/Euskal Herriko Unibertsitatea (UPV/EHU), Paseo de la Universidad 7, 01006 Vitoria-Gasteiz, Spain; alba.rodriguezp@ehu.eus

2 Department of Molecular Biology and Genetics and Interdisciplinary Nanoscience Center (iNANO), Aarhus University, 8000 Aarhus, Denmark; ctesauro@mbg.au.dk (C.T.); brk@mbg.au.dk (B.R.K.)

* Correspondence: maria.fuertes@ehu.eus (M.F.); concepcion.alonso@ehu.eus (C.A.)

Table of contents

Supplementary Table S1 3

NMR spectra 4

Table S1. Results of the ADME predictive study for diphenylphosphine oxide derivatives.

Entry	Comp.	Log S (mol/L)	Caco-2 Perm. (Log Paap in 10^{-6}cm/s)	Int. Abs. (% abs)	VDss (L/Kg)	Fract. Unb. (Fu.)	BBB Permeability (Log BB)	PGP subs.	PGP I inhib.	PGP II inhib.	CYP1A2 Inhib.	CYP2C19 Inhib.	CYP2C9 Inhib.	CYP2D6 inhib.	CYP3A4 Inhib.	Total Clearence numeric (Log mL/min/Kg)
**1**	**CPT**	-3.01	1.305	99.865	0.448	0.126	no	yes	yes	no	yes	no	yes	no	no	0.629
**2**	**6a**	-4.821	0.911	100	-0.9	0.321	yes	yes	yes	yes	yes	no	no	no	no	-0.506
**3**	**6b**	-4.676	0.911	100	-0.994	0.315	yes	yes	yes	yes	yes	no	no	no	no	-0.584
**4**	**6c**	-4.65	0.946	100	-1.049	0.325	yes	yes	yes	yes	yes	no	no	no	no	-0.652
**5**	**6d**	-4.607	0.948	100	-1.059	0.326	yes	yes	yes	yes	yes	no	no	no	no	-0.646
**6**	**6e**	-4.114	0.962	100	-1.204	0.338	yes	yes	yes	yes	yes	no	no	no	no	-0.692
**7**	**6f**	-4.092	0.992	100	-1.199	0.346	yes	yes	yes	yes	yes	no	no	no	no	-0.555
**8**	**6g**	-3.6	0.876	100	-0.489	0.362	yes	yes	yes	yes	yes	no	no	no	no	-0.542
**9**	**6k**	-4.049	-0.029	100	-0.853	0.329	yes	yes	yes	yes	yes	no	no	no	no	-0.485
**10**	**7a**	-4.376	0.897	100	-0.427	0.371	yes	yes	yes	yes	no	no	no	no	no	-0.106
**11**	**7b**	-4.353	0.899	100	-0.487	0.37	yes	yes	yes	yes	no	no	no	no	no	-0.179
**12**	**7c**	-3.964	0.933	100	-0.476	0.373	yes	no	yes	yes	yes	no	no	no	no	-0.361
**13**	**7d**	-3.898	0.943	100	-0.483	0.373	yes	no	no	yes	yes	no	no	no	no	-0.239
**14**	**7f**	-3.444	0.989	100	-0.521	0.38	yes	no	yes	yes	yes	no	no	no	no	-0.262
**15**	**7g**	-3.189	0.853	100	-0.172	0.38	yes	no	yes	yes	no	no	no	no	no	-0.138
**16**	**7h**	-3.98	0.857	100	-0.507	0.372	no	no	yes	yes	yes	no	no	no	no	-0.181
**17**	**7i**	-3.948	0.906	100	-0.522	0.372	no	no	no	yes	yes	no	no	no	no	-0.057
**18**	**7j**	-4.418	0.884	100	-0.456	0.37	yes	yes	yes	yes	no	no	no	no	no	-0.253
**19**	**8a**	-4.040	0.939	100	-0.570	0.372	no	no	yes	yes	yes	no	no	no	no	0.105
**20**	**8b**	-3.995	0.941	100	-0.633	0.372	no	no	yes	yes	yes	no	no	no	no	0.028
**21**	**8d**	-3.481	0.988	100	-0.614	0.378	no	no	yes	yes	yes	no	no	no	no	-0.03
**22**	**8e**	-3.163	1.004	100	-0.637	0.385	no	no	yes	yes	no	no	no	no	no	-0.133
**23**	**8g**	-3.155	0.899	100	-0.263	0.379	no	no	yes	yes	no	no	no	no	no	0.067

^{1}H-RMN

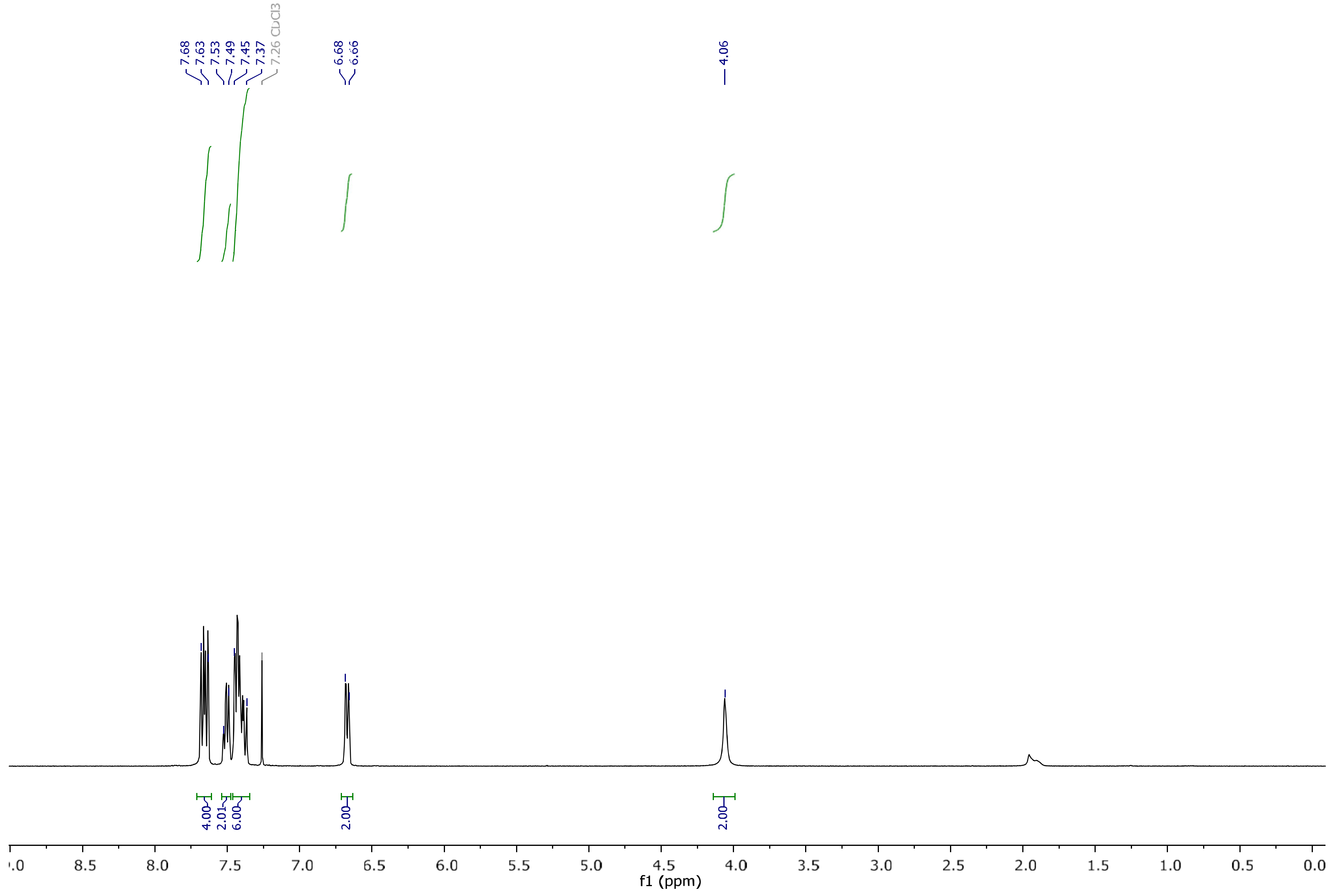

7.68
7.63
7.53
7.49
7.45
7.37
7.26 CDCl3
6.68
6.66
4.06
4.00
2.01
6.00
2.00
2.00
9.0
8.5
8.0
7.5
7.0
6.5
6.0
5.5
5.0
4.5
4.0
3.5
3.0
2.5
2.0
1.5
1.0
0.5
0.0
f1 (ppm)

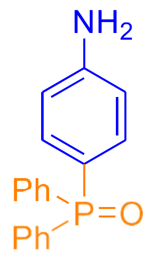

NH2
Ph
P=O
Ph

3

^{31}P-RMN

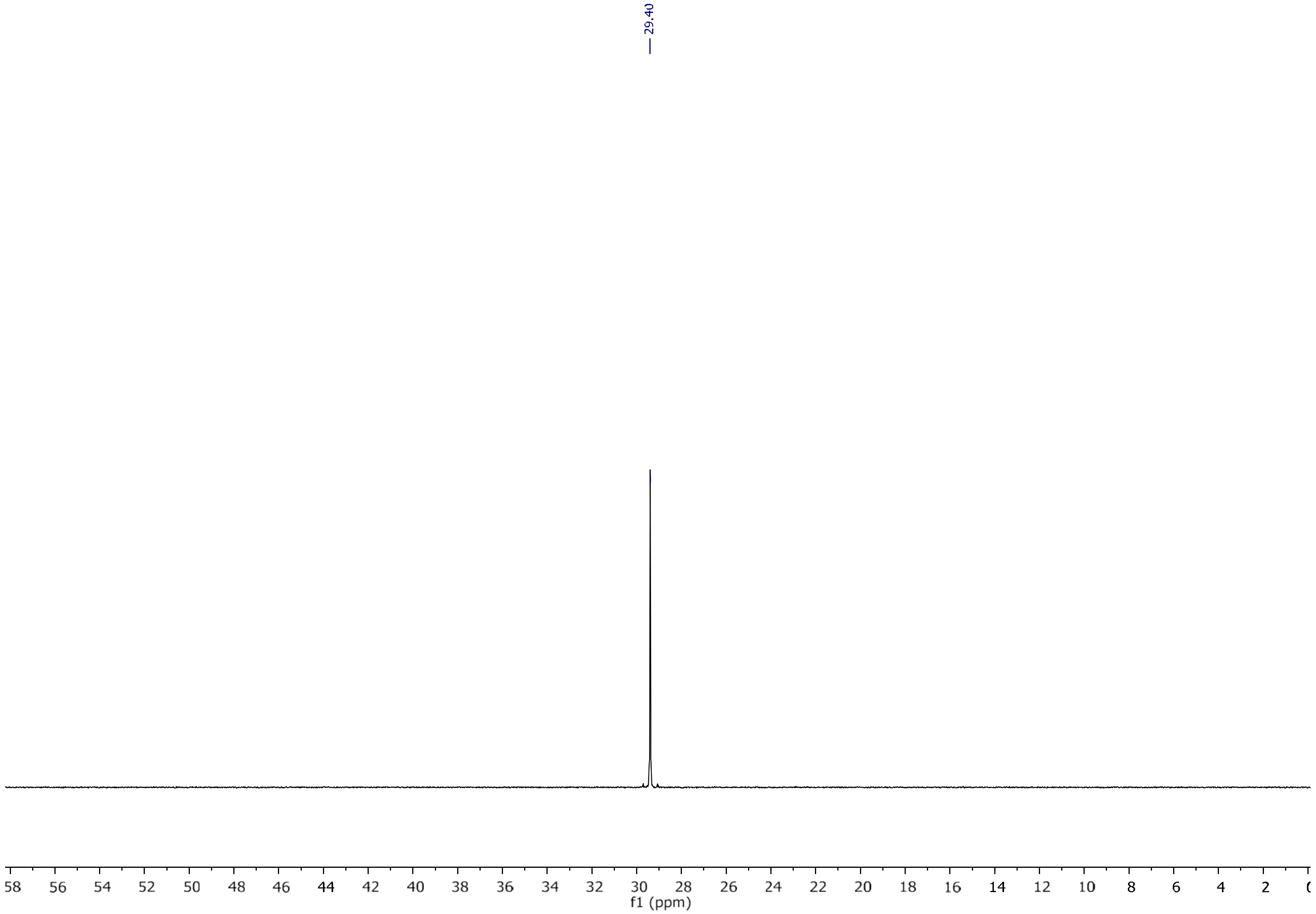
29.40
58 56 54 52 50 48 46 44 42 40 38 36 34 32 30 28 26 24 22 20 18 16 14 12 10 8 6 4 2
f1 (ppm)

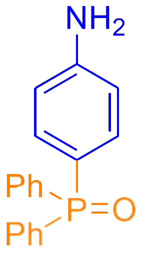
NH2
Ph
P=O
Ph

3

^{13}C-RMN

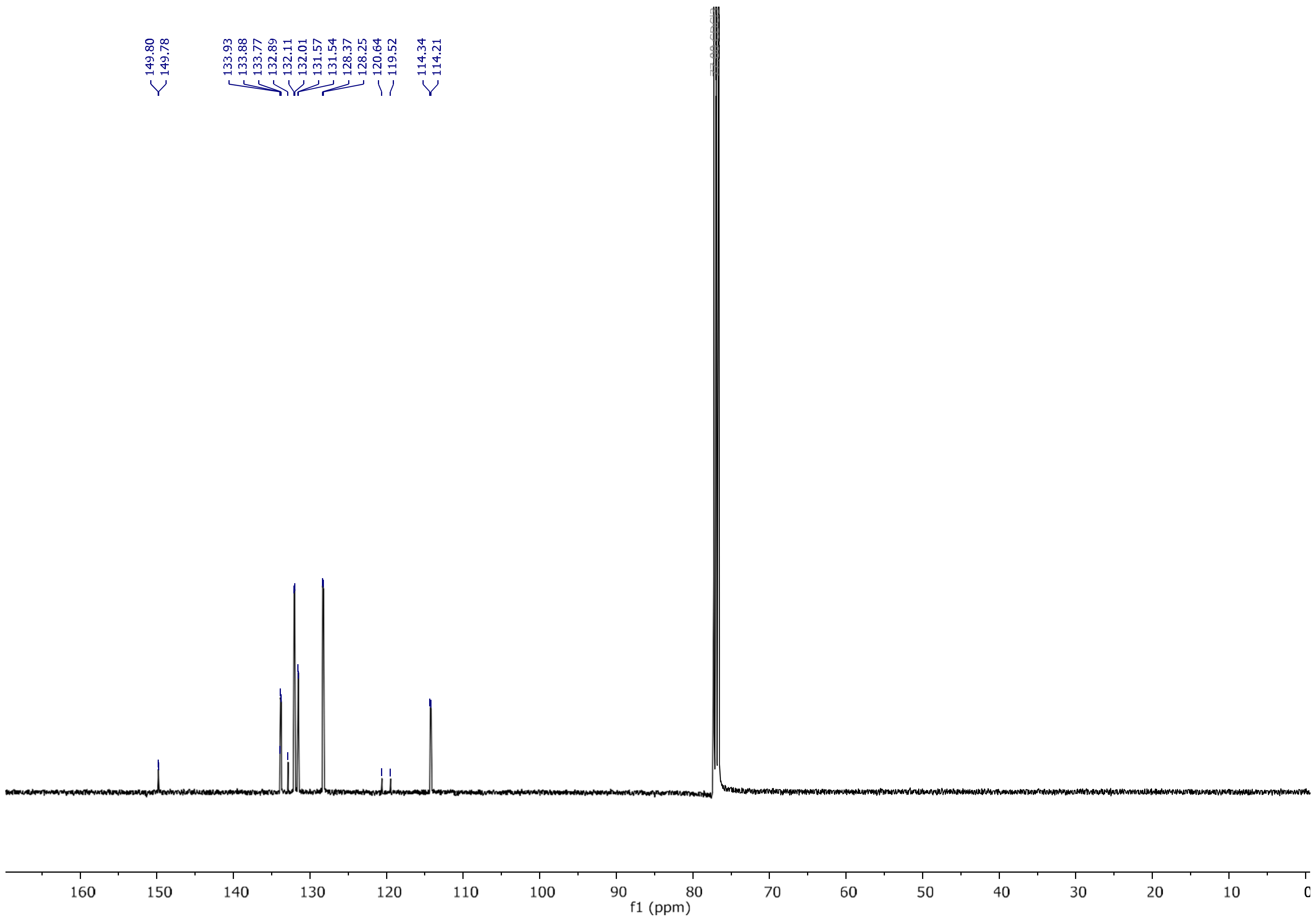
149.80
149.78
133.93
133.88
133.77
132.89
132.11
132.01
131.57
131.54
128.37
128.25
120.64
119.52
114.34
114.21
160
150
140
130
120
110
100
90
80
70
60
50
40
30
20
10
0
f1 (ppm)

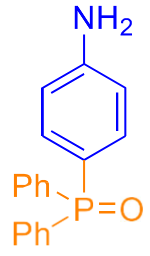
NH2
Ph
P=O
Ph

3

^{1}H-RMN

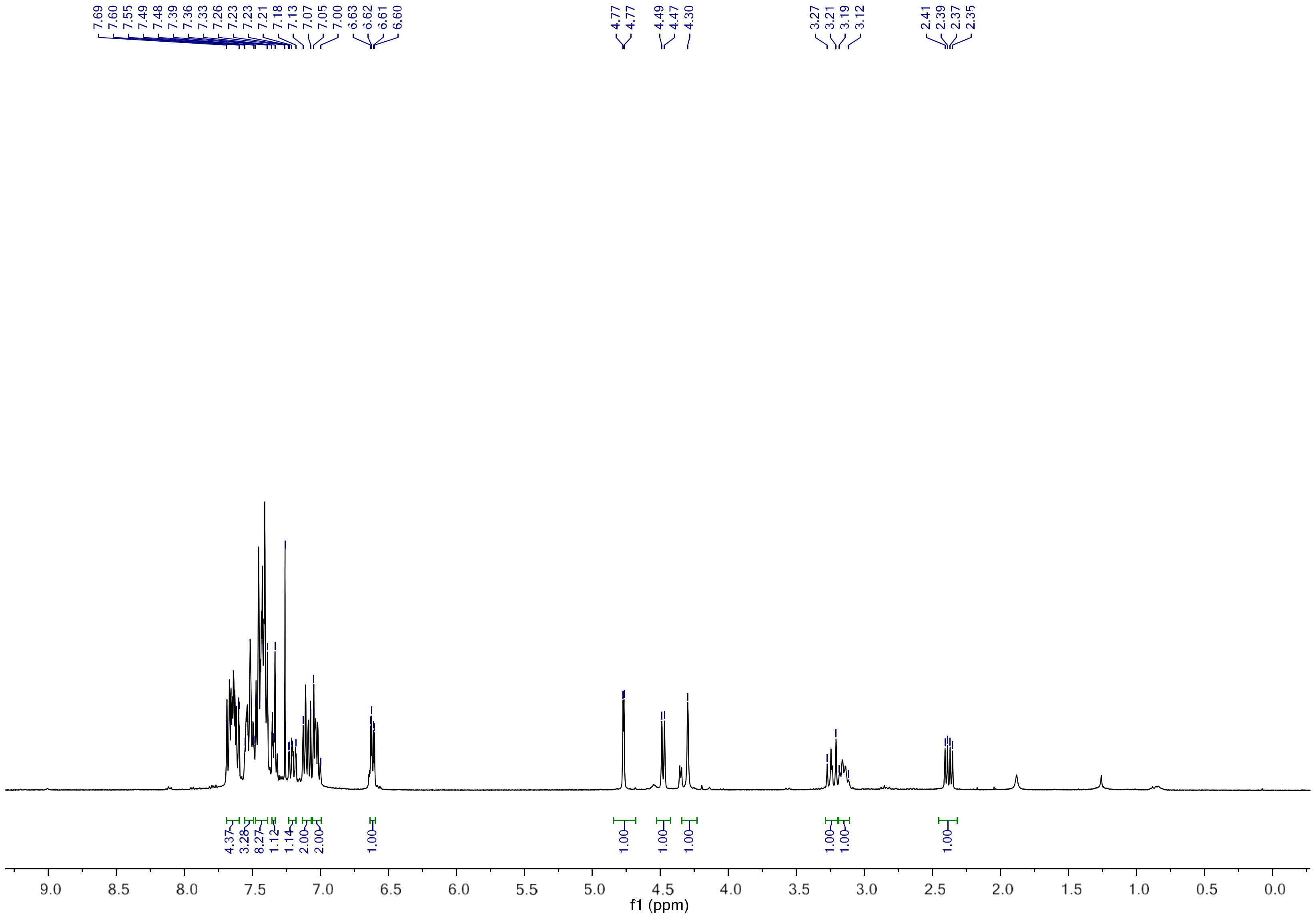

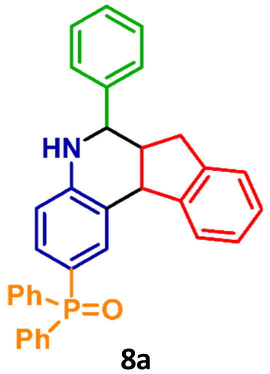

8a

^{31}P-RMN

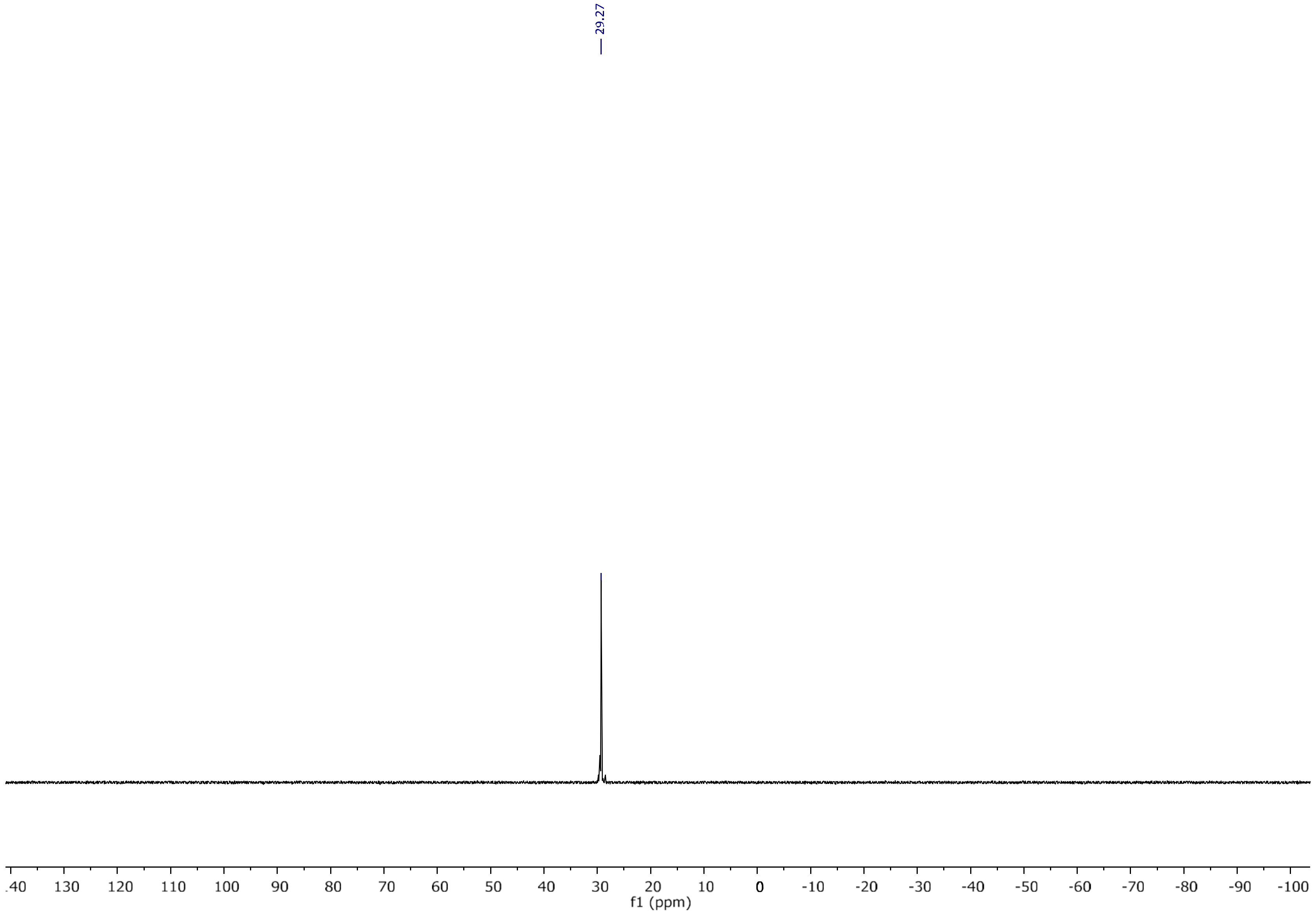
29.27
.40 130 120 110 100 90 80 70 60 50 40 30 20 10 0 -10 -20 -30 -40 -50 -60 -70 -80 -90 -100
f1 (ppm)

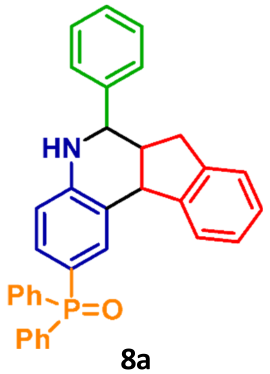
HN
Ph
P=O
Ph

8a

^{13}C-RMN

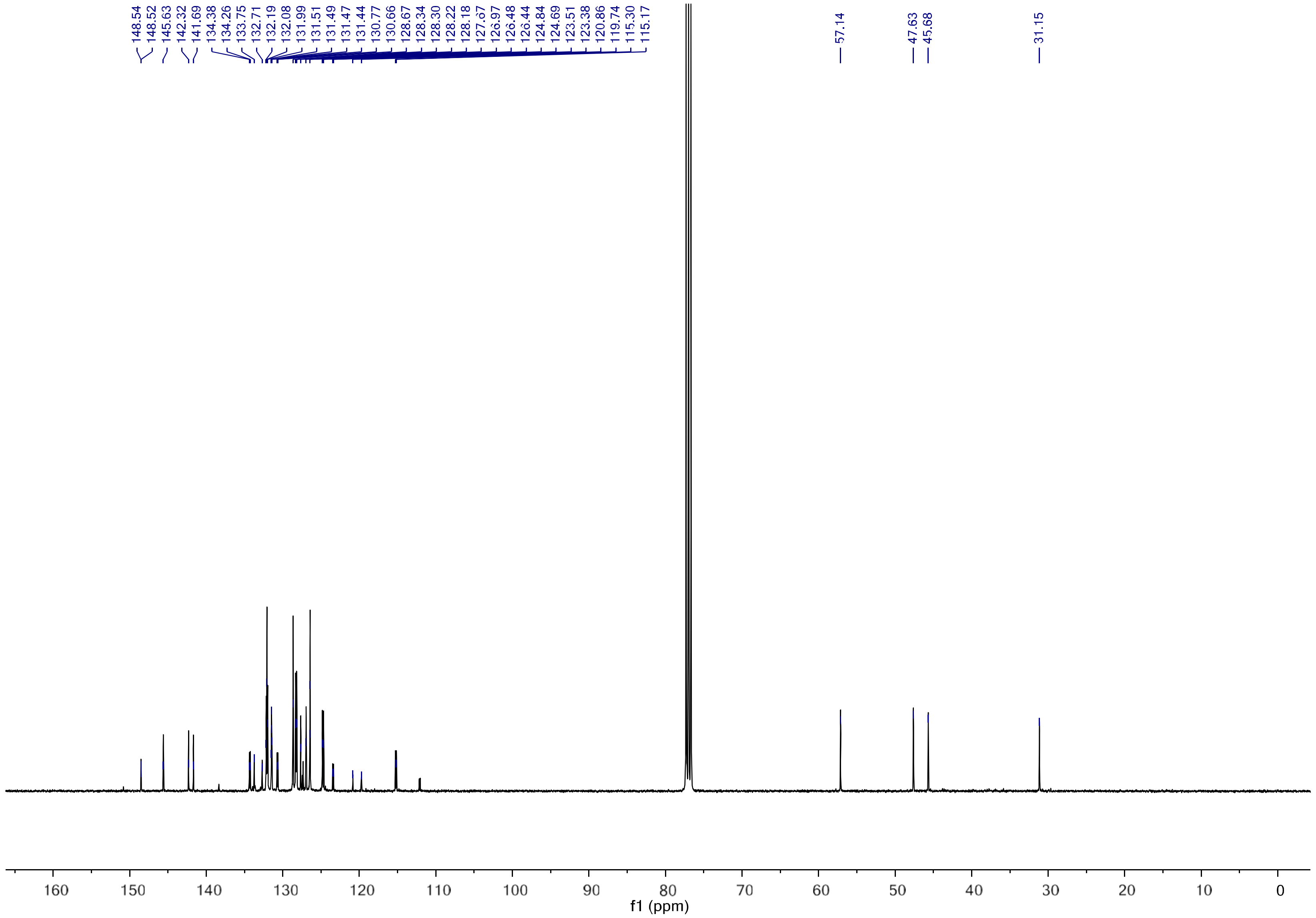
148.54
148.52
145.63
142.32
141.69
134.38
134.26
133.75
132.71
132.19
132.08
131.99
131.51
131.49
131.47
131.44
130.77
130.66
128.67
128.34
128.30
128.22
128.18
127.67
126.97
126.48
126.44
124.84
124.69
123.51
123.38
120.86
119.74
115.30
115.17
57.14
47.63
45.68
31.15
160
150
140
130
120
110
100
90
80
70
60
50
40
30
20
10
0
f1 (ppm)

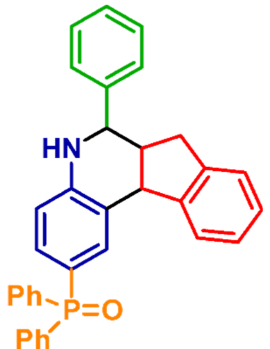
HN
Ph
P=O
Ph

8a

^{1}H-RMN

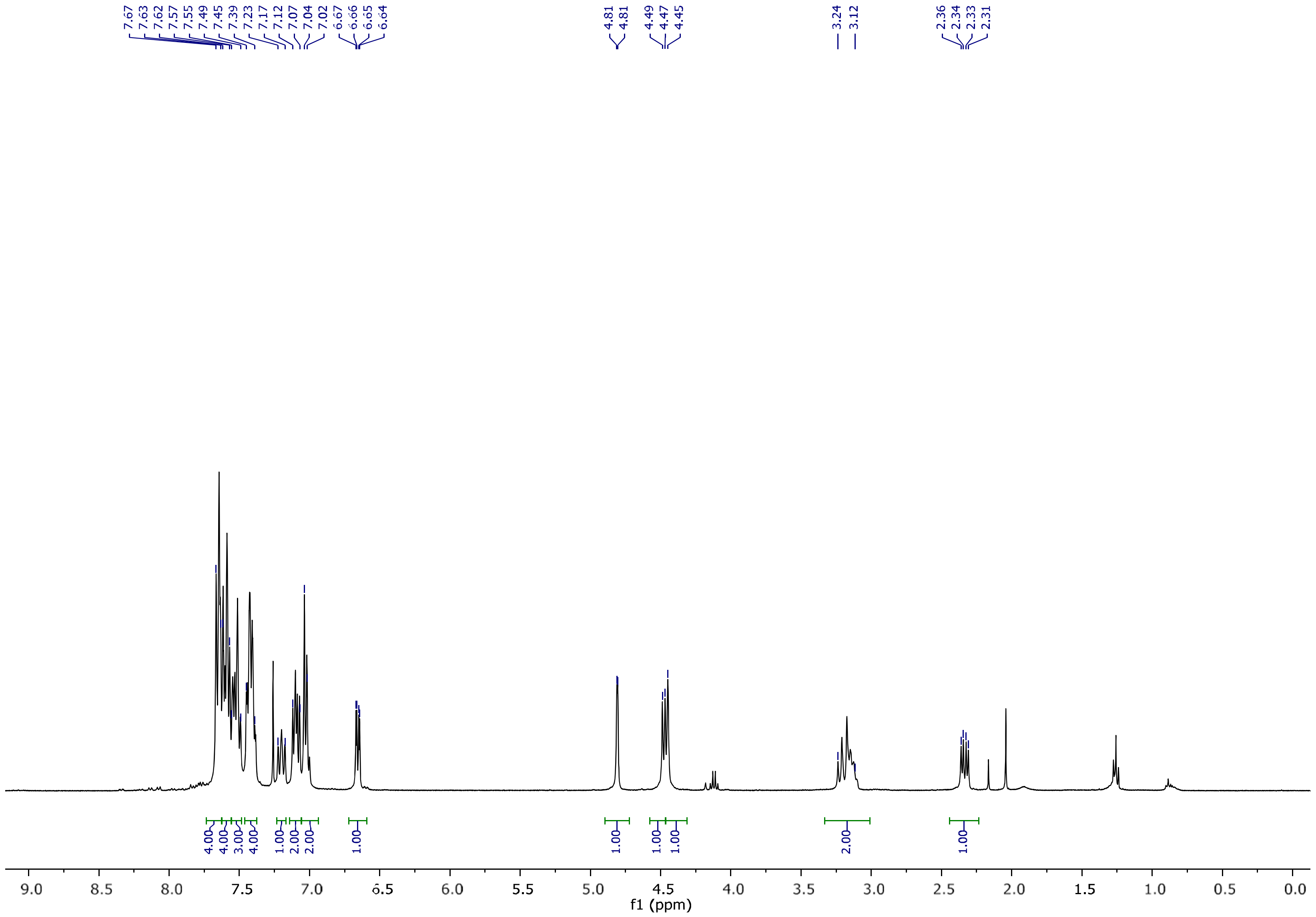

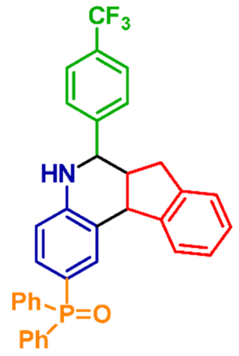

8b

^{31}P-RMN

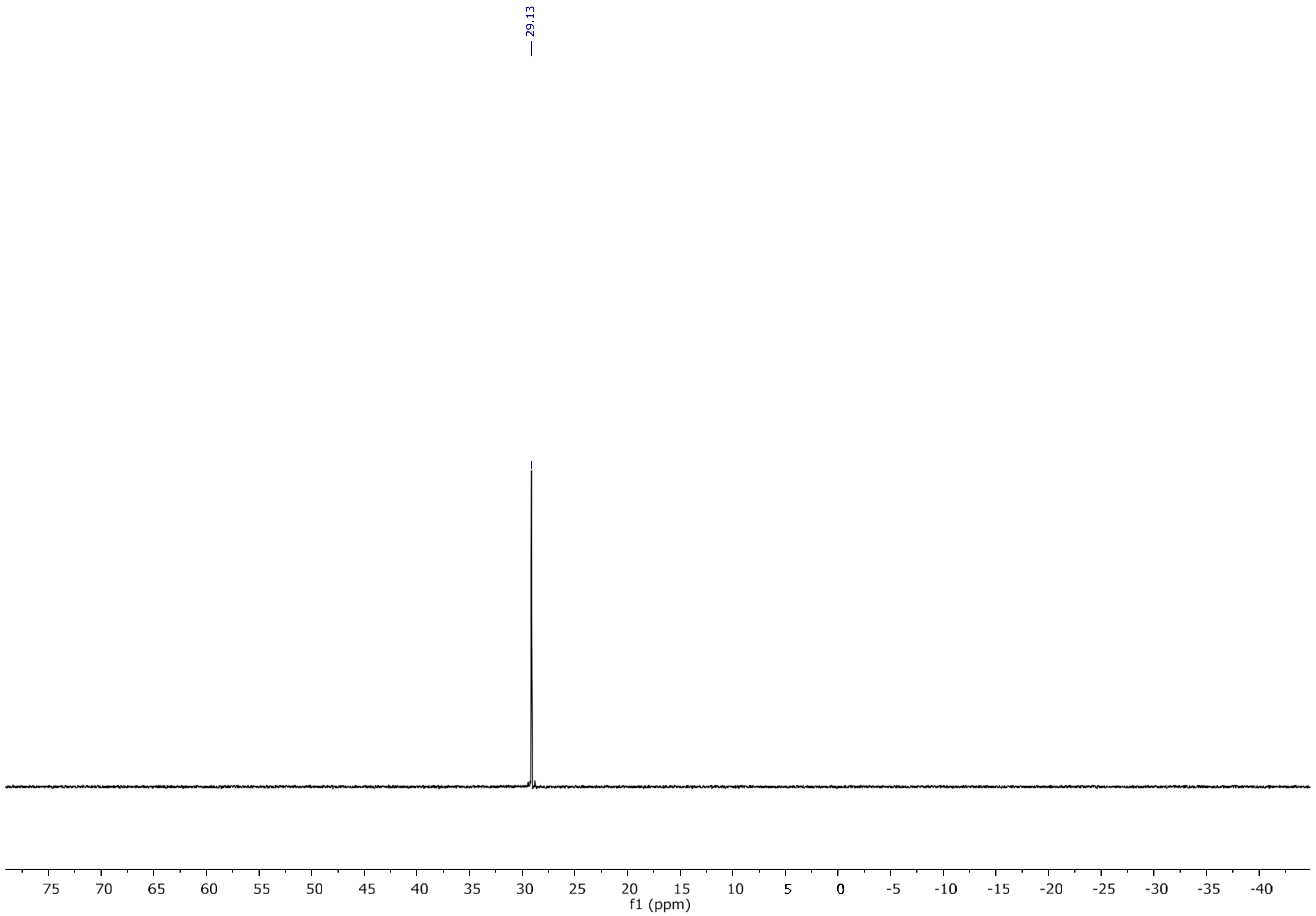
29.13
75 70 65 60 55 50 45 40 35 30 25 20 15 10 5 0 -5 -10 -15 -20 -25 -30 -35 -40
f1 (ppm)

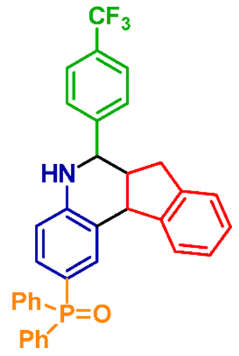
CF3
HN
Ph
P=O
Ph

8b

^{19}F-RMN

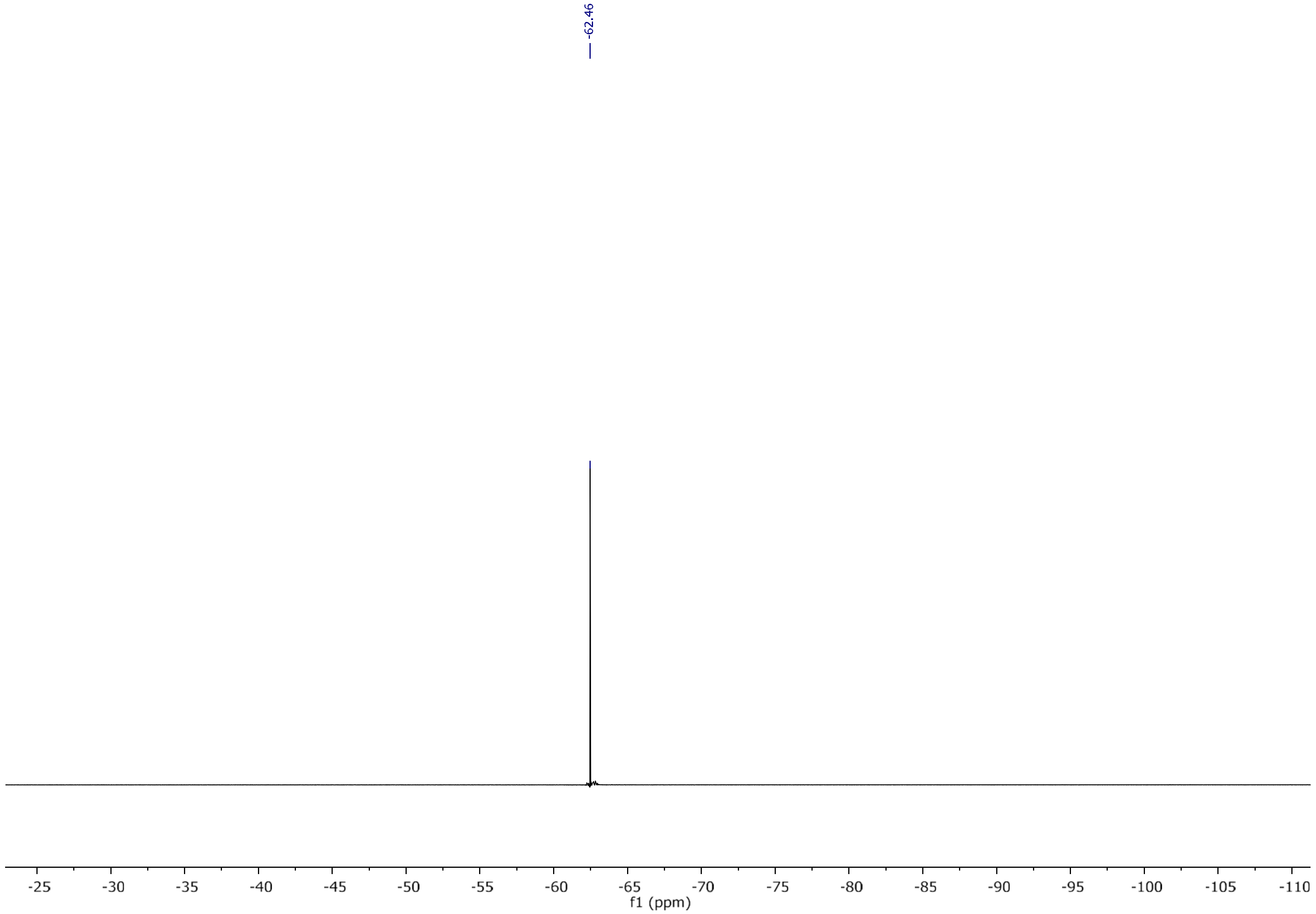

-62.46
-25 -30 -35 -40 -45 -50 -55 -60 -65 -70 -75 -80 -85 -90 -95 -100 -105 -110
f1 (ppm)

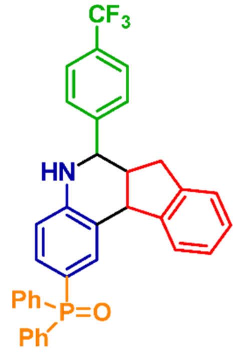

CF3
HN
Ph
P=O
Ph

8b

^{13}C-RMN

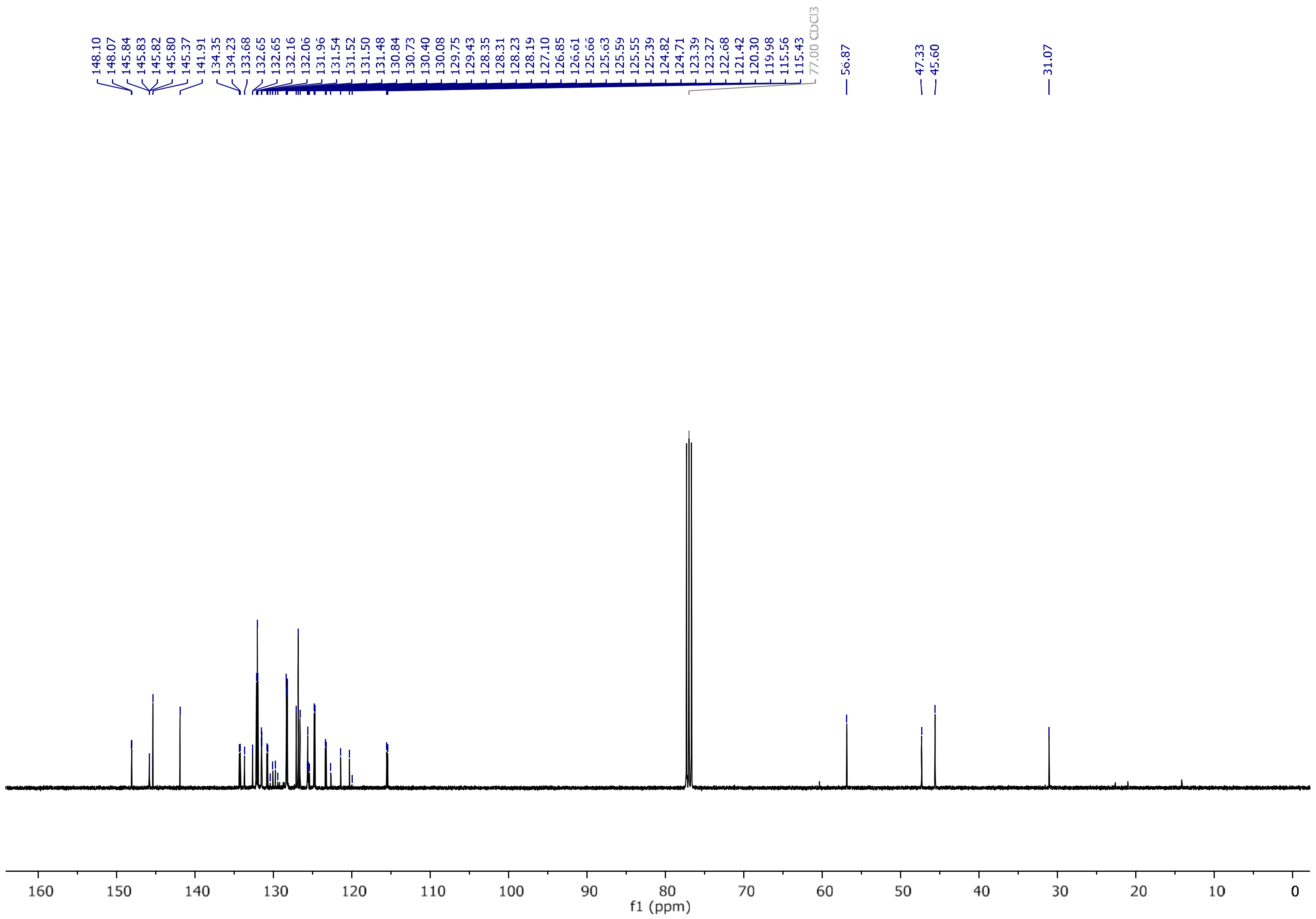
148.10
148.07
145.84
145.83
145.82
145.80
145.37
141.91
134.35
134.23
133.68
132.65
132.65
132.16
132.06
131.96
131.54
131.52
131.50
131.48
130.84
130.73
130.40
130.08
129.75
129.43
128.35
128.31
128.23
128.19
127.10
126.85
126.61
125.66
125.63
125.59
125.55
125.39
124.82
124.71
123.39
123.27
122.68
121.42
120.30
119.98
115.56
115.43
77.00 CDCl3
56.87
47.33
45.60
31.07
160 150 140 130 120 110 100 90 80 70 60 50 40 30 20 10 0
f1 (ppm)

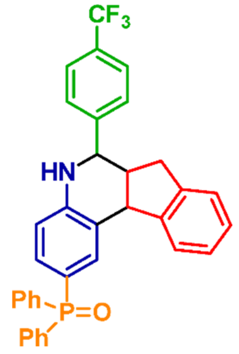
CF3
HN
Ph
P=O
Ph

8b

^{1}H-RMN

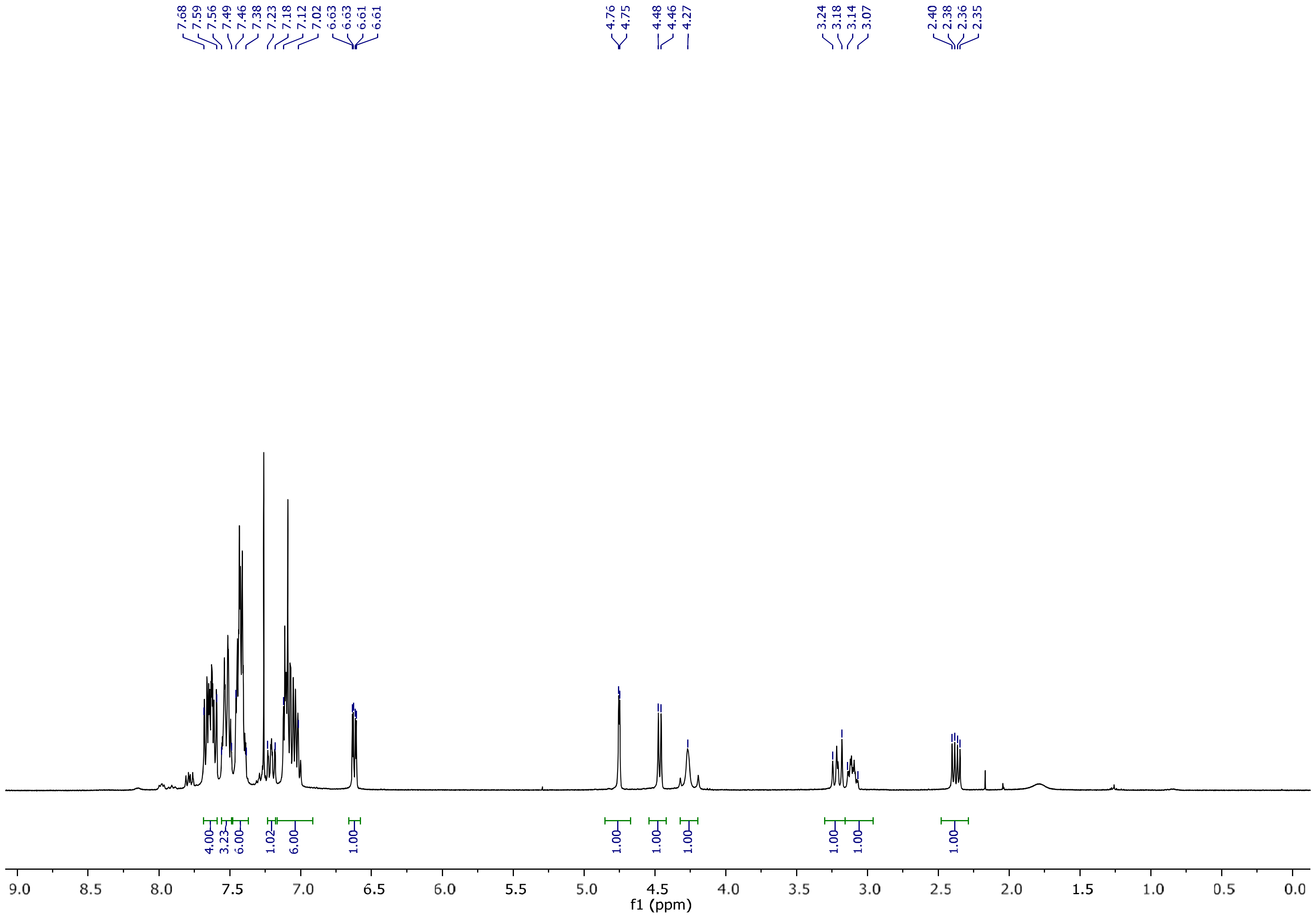

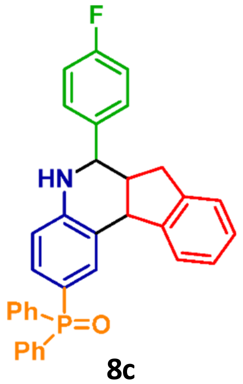

8c

^{31}P-RMN

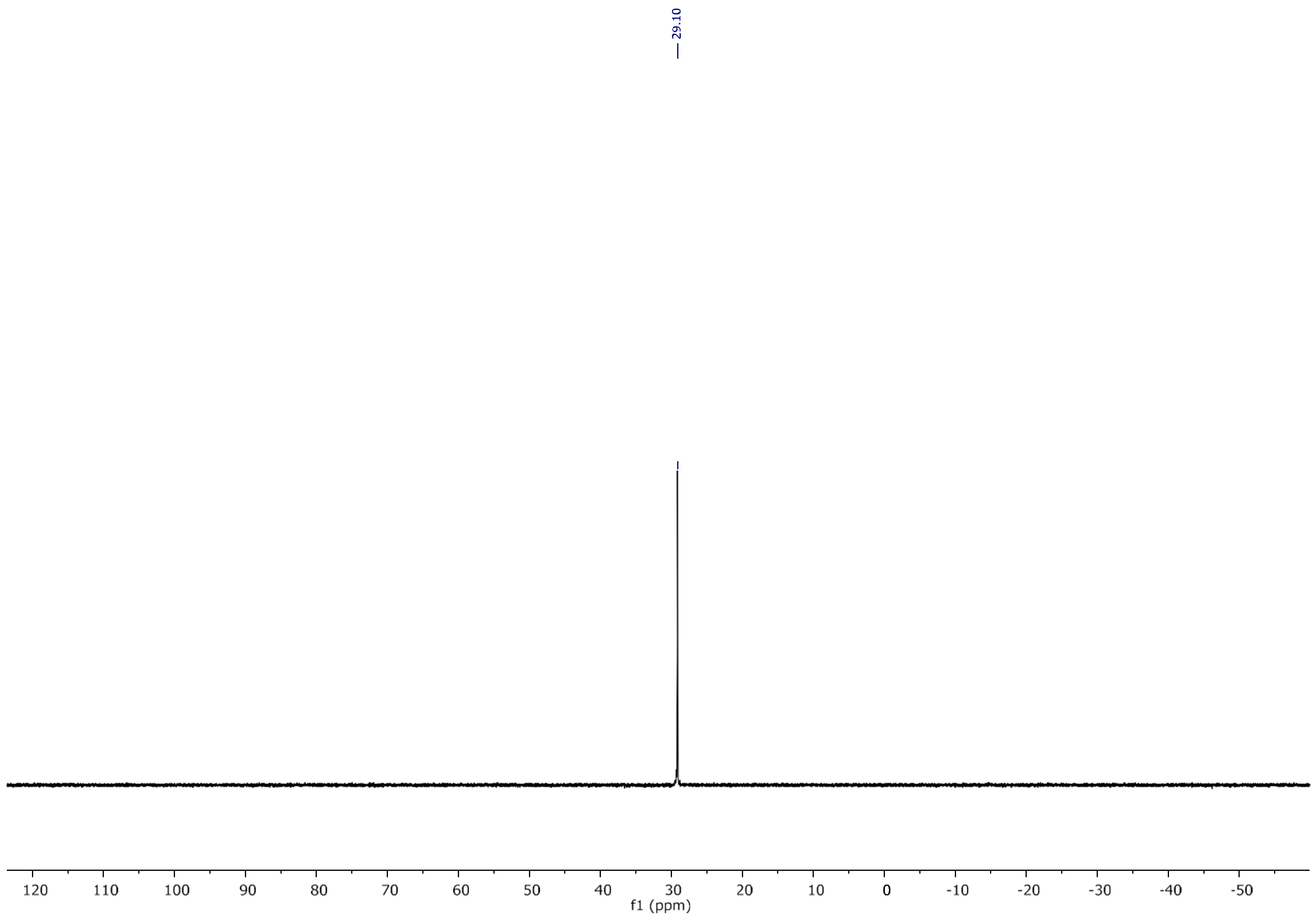

29.10
120
110
100
90
80
70
60
50
40
30
20
10
0
-10
-20
-30
-40
-50
f1 (ppm)

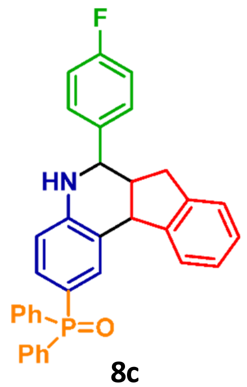

F
HN
Ph
P=O
Ph

8c

^{19}F-RMN

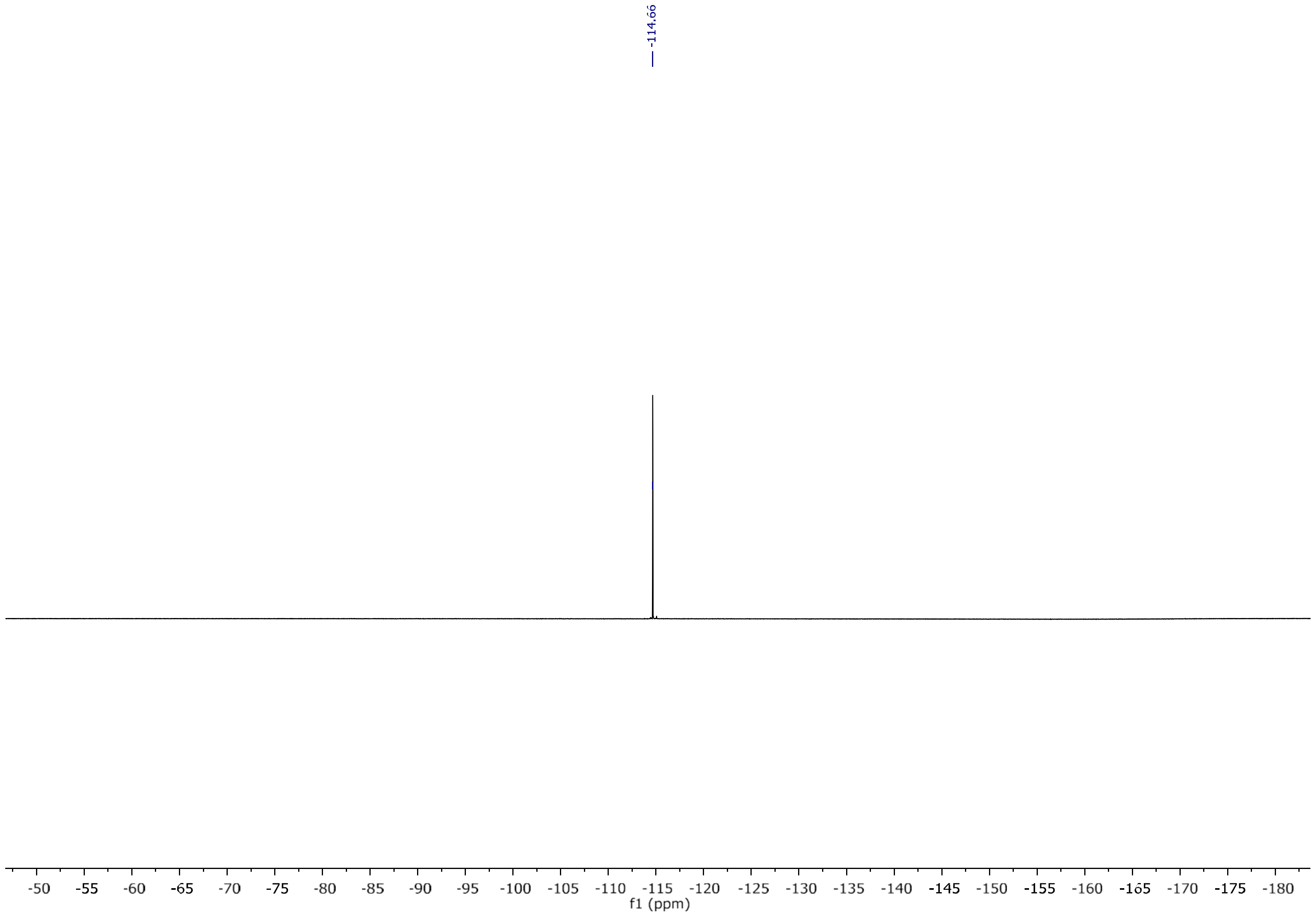

-114.66
-50
-55
-60
-65
-70
-75
-80
-85
-90
-95
-100
-105
-110
-115
-120
-125
-130
-135
-140
-145
-150
-155
-160
-165
-170
-175
-180
f1 (ppm)

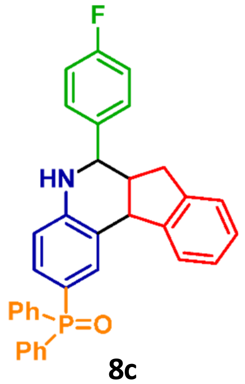

F
HN
Ph
P=O
Ph

8c

^{13}C-RMN

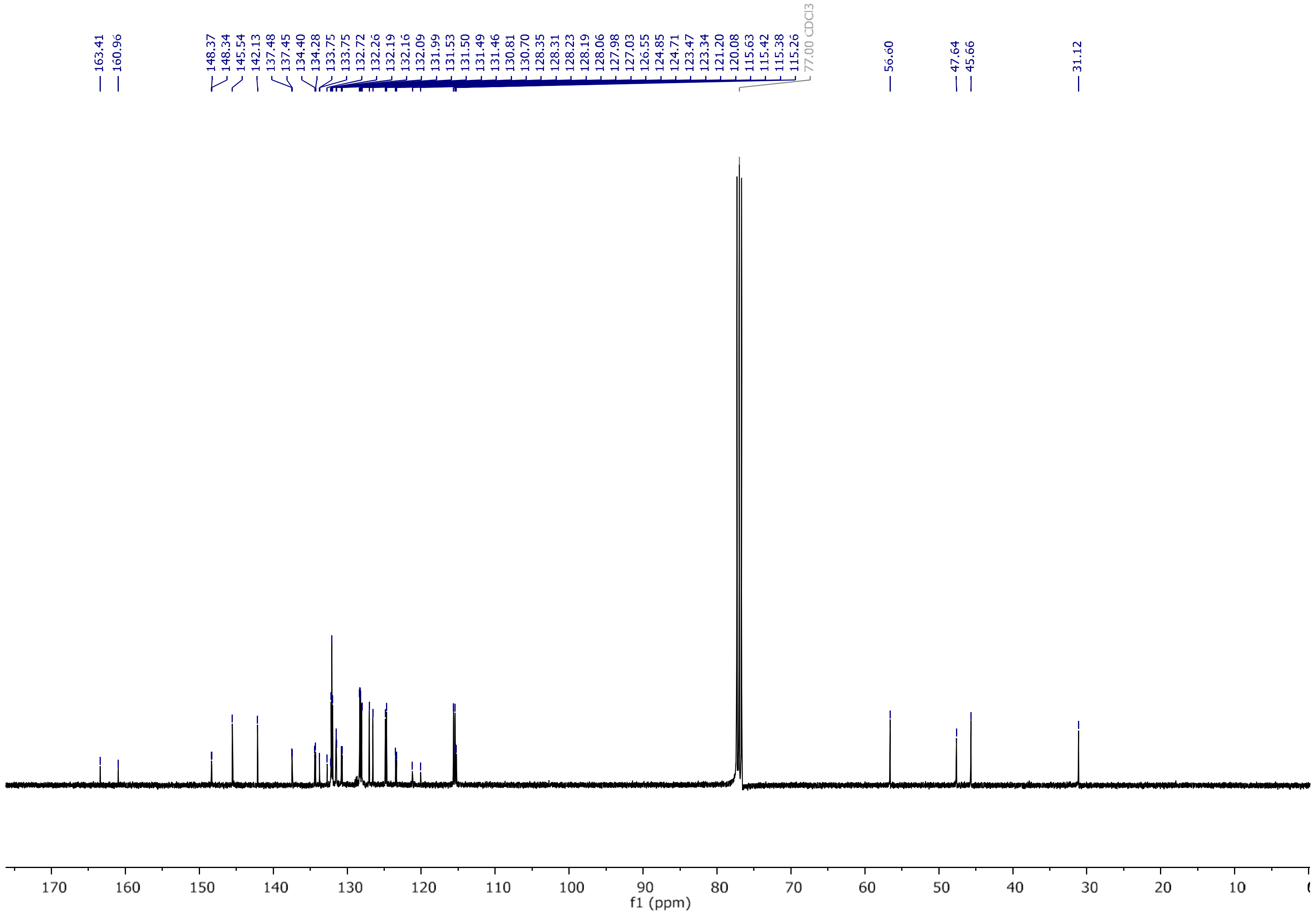
163.41
160.96
148.37
148.34
145.54
142.13
137.48
137.45
134.40
134.28
133.75
133.75
132.72
132.26
132.19
132.16
132.09
131.99
131.53
131.50
131.49
131.46
130.81
130.70
128.35
128.31
128.23
128.19
128.06
127.98
127.03
126.55
124.85
124.71
123.47
123.34
121.20
120.08
115.63
115.42
115.38
115.26
77.00 CDCl3
56.60
47.64
45.66
31.12
170
160
150
140
130
120
110
100
90
80
70
60
50
40
30
20
10
f1 (ppm)

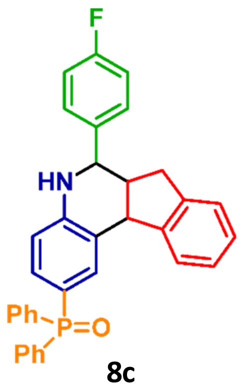
F
HN
Ph
P=O
Ph

8c

^{1}H-RMN

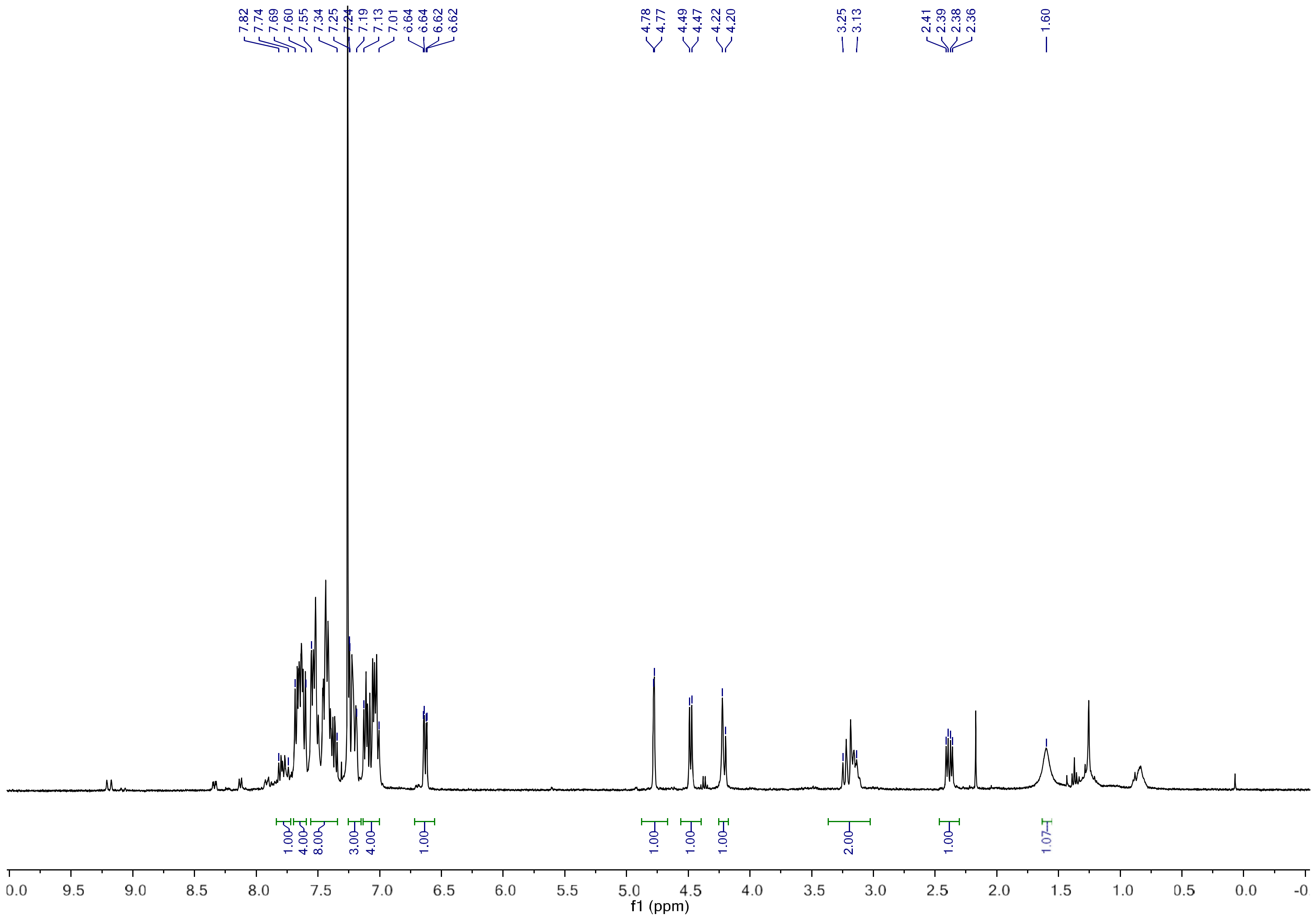

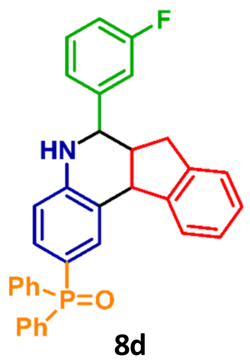

8d

^{31}P-RMN

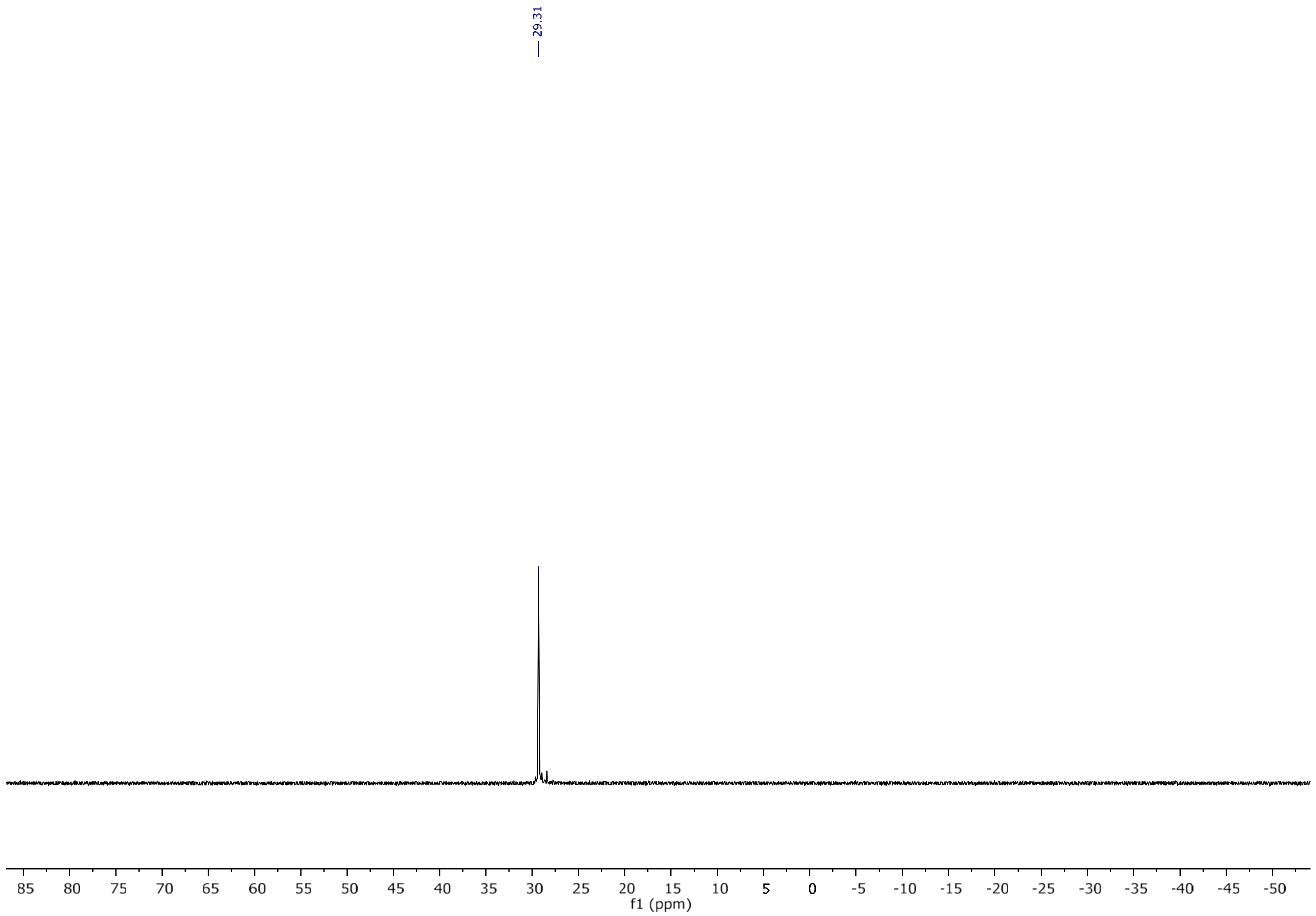

29.31
85 80 75 70 65 60 55 50 45 40 35 30 25 20 15 10 5 0 -5 -10 -15 -20 -25 -30 -35 -40 -45 -50
f1 (ppm)

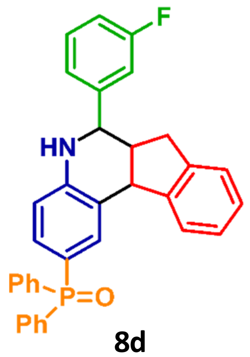

F
HN
Ph
P=O
Ph

8d

^{19}F-RMN

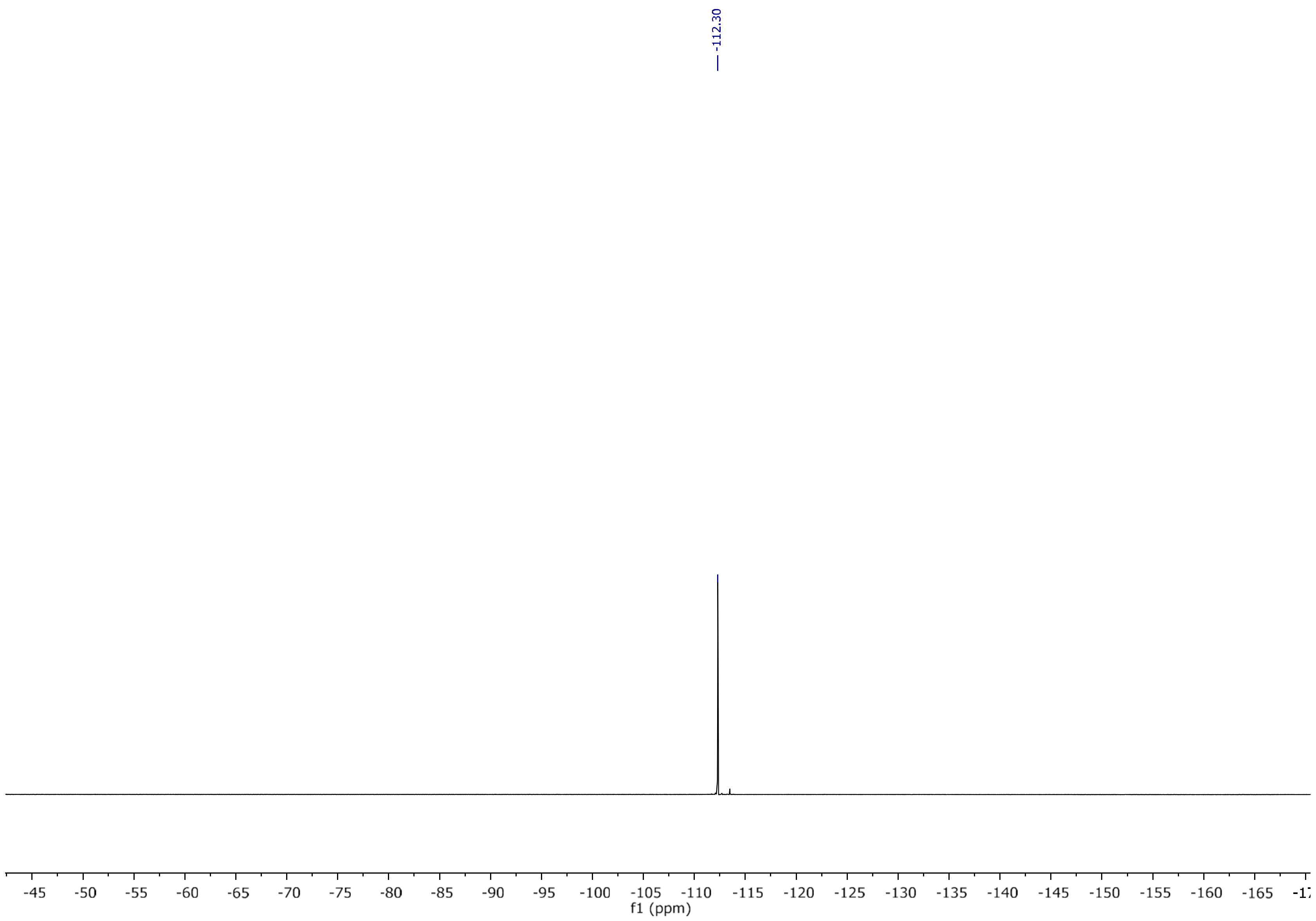

-112.30
-45
-50
-55
-60
-65
-70
-75
-80
-85
-90
-95
-100
-105
-110
-115
-120
-125
-130
-135
-140
-145
-150
-155
-160
-165
f1 (ppm)

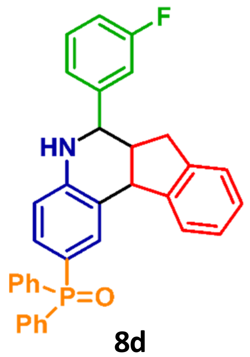

F
HN
Ph
P=O
Ph

8d

^{13}C-RMN

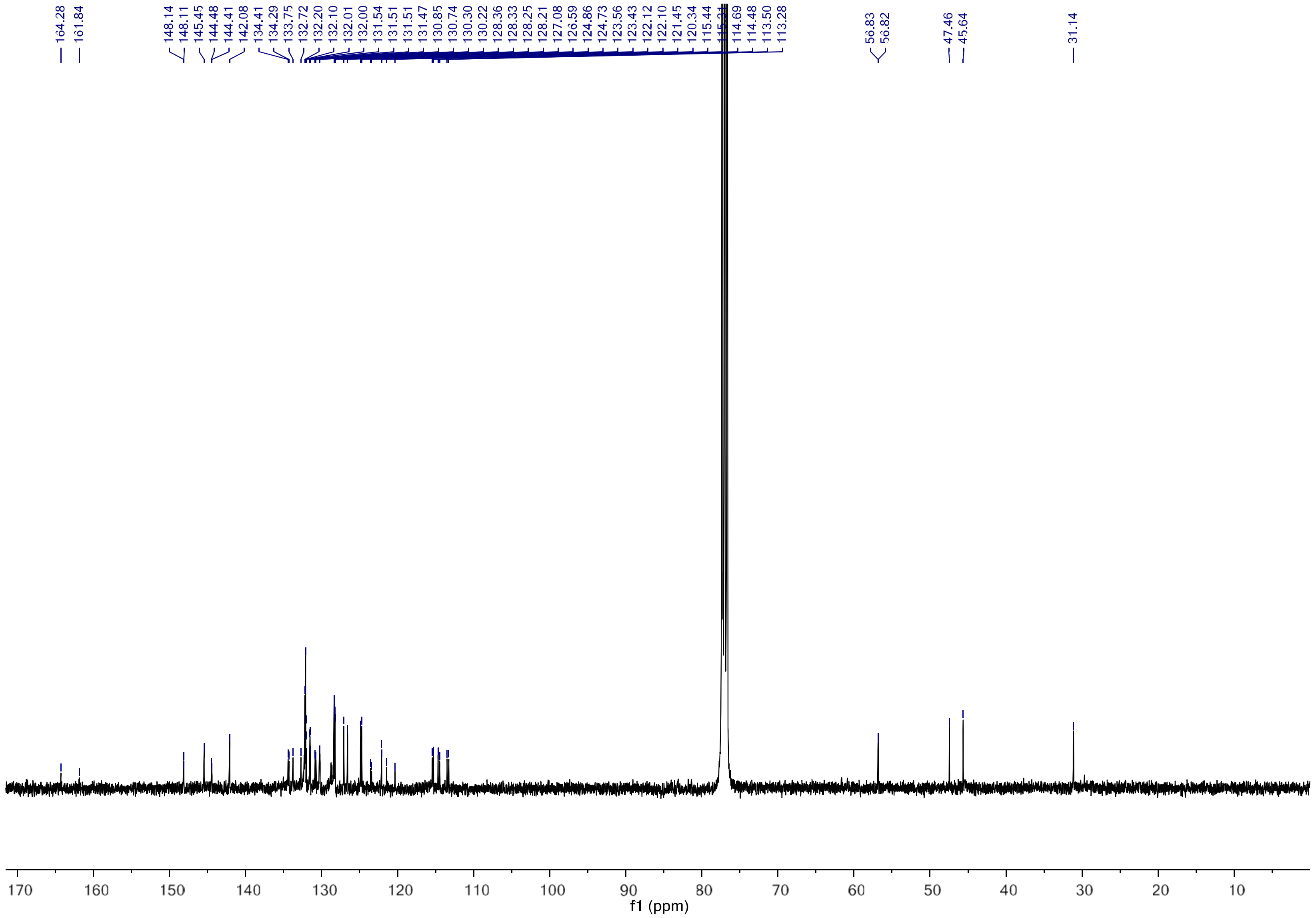
164.28
161.84
148.14
148.11
145.45
144.48
144.41
142.08
134.41
134.29
133.75
132.72
132.20
132.10
132.01
132.00
131.54
131.51
131.51
131.47
130.85
130.74
130.30
130.22
128.36
128.33
128.25
128.21
127.08
126.59
124.86
124.73
123.56
123.43
122.12
122.10
121.45
120.34
115.44
114.69
114.48
113.50
113.28
56.83
56.82
47.46
45.64
31.14
170
160
150
140
130
120
110
100
90
80
70
60
50
40
30
20
10
f1 (ppm)

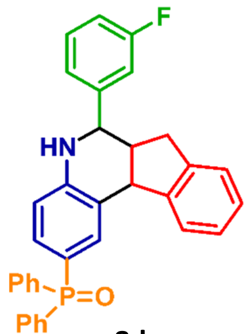
F
HN
Ph
P=O
Ph

8d

^{1}H-RMN

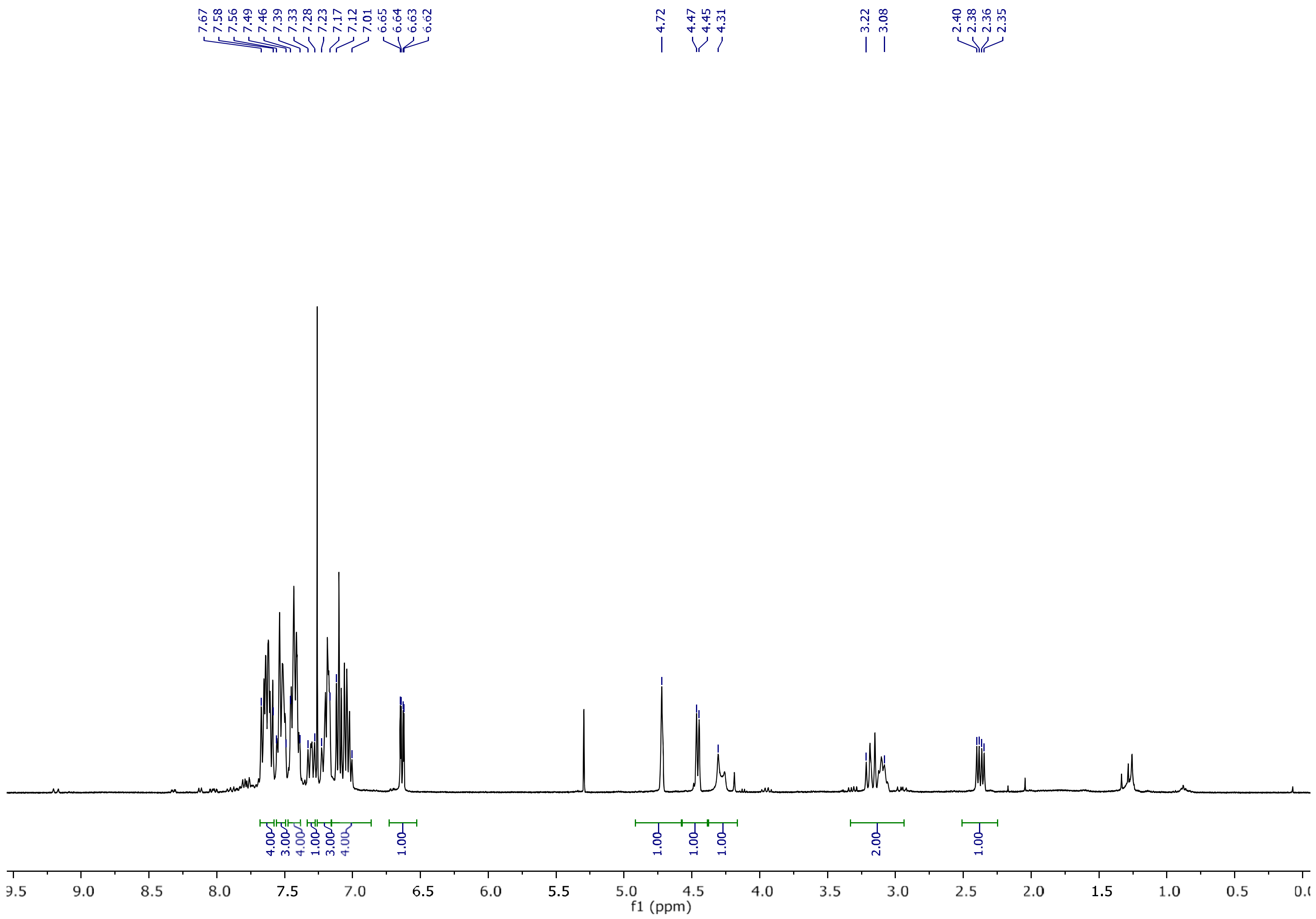

7.67
7.58
7.56
7.49
7.46
7.39
7.33
7.28
7.23
7.17
7.12
7.01
6.65
6.64
6.63
6.62
4.72
4.47
4.45
4.31
3.22
3.08
2.40
2.38
2.36
2.35
4.00
3.00
4.00
1.00
3.00
4.00
1.00
1.00
1.00
1.00
2.00
1.00
9.0
8.5
8.0
7.5
7.0
6.5
6.0
5.5
5.0
4.5
4.0
3.5
3.0
2.5
2.0
1.5
1.0
0.5
f1 (ppm)

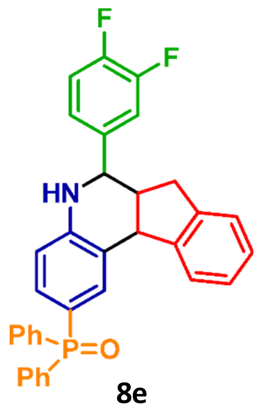

F
F
HN
Ph
P=O
Ph

8e

^{31}P-RMN

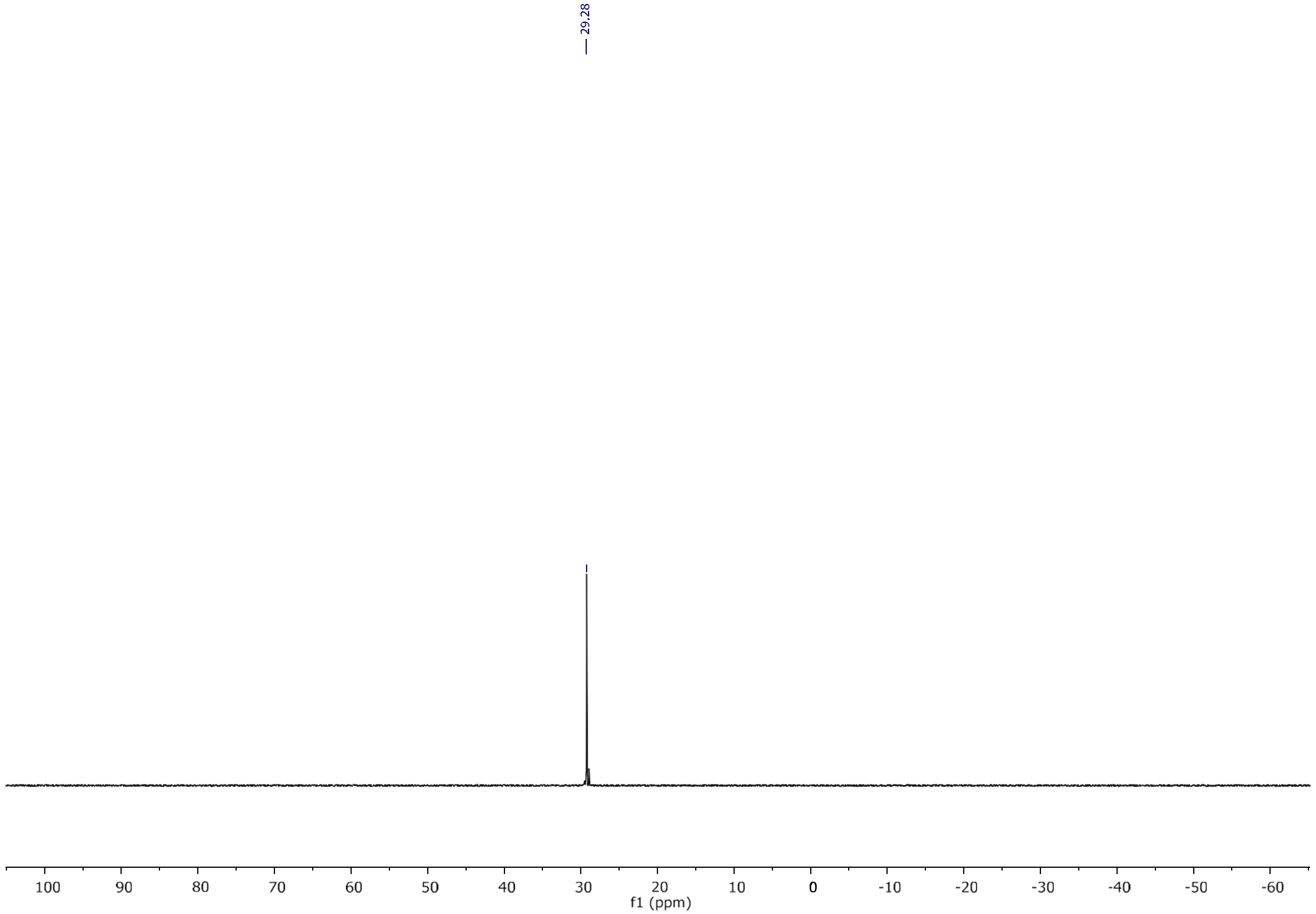
29.28
100
90
80
70
60
50
40
30
20
10
0
-10
-20
-30
-40
-50
-60
f1 (ppm)

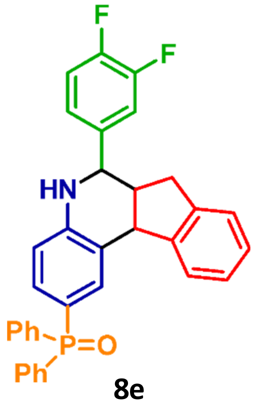
F
F
HN
Ph
P=O
Ph

8e

^{19}F-RMN

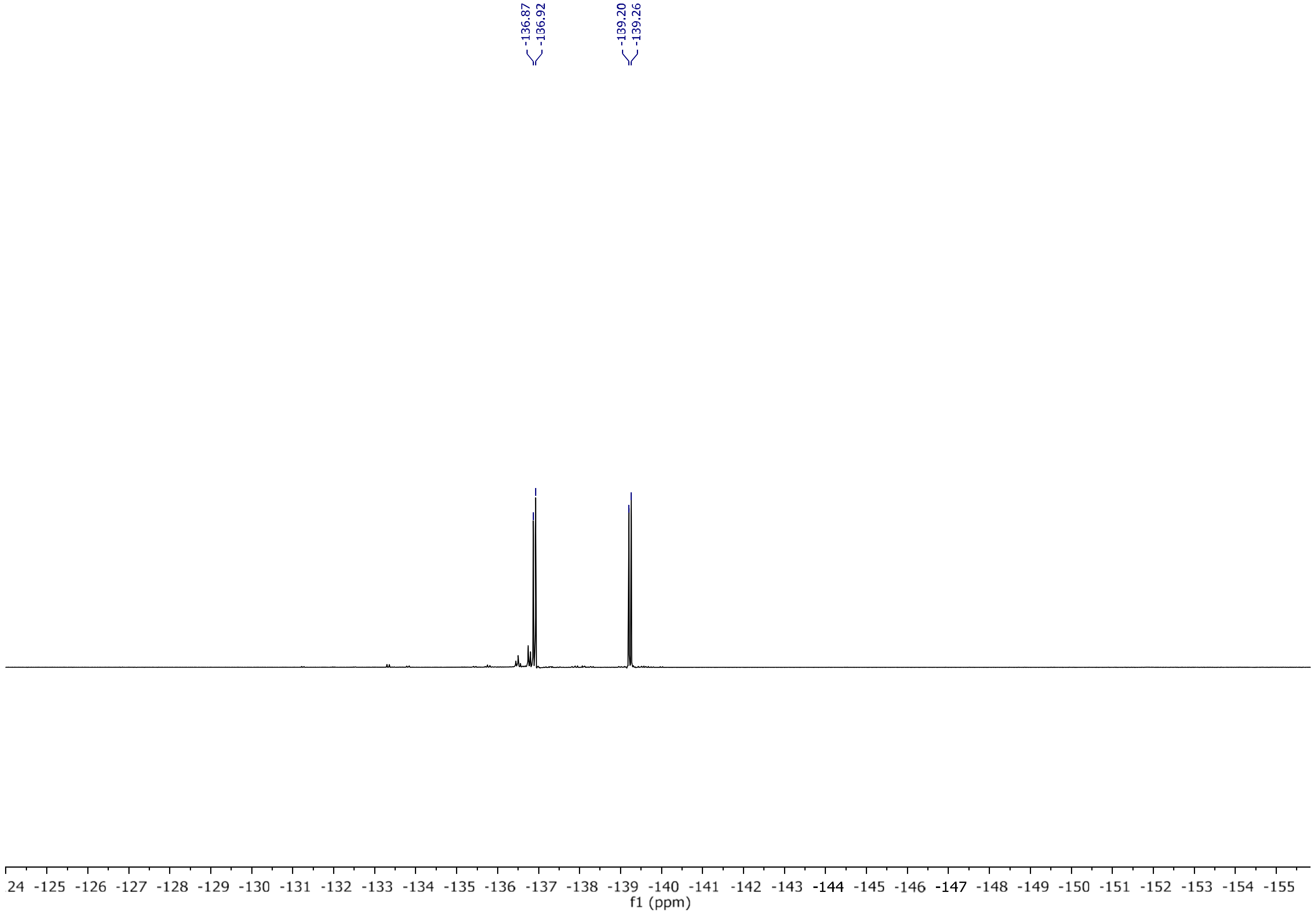
-136.87
-136.92
-139.20
-139.26
24 -125 -126 -127 -128 -129 -130 -131 -132 -133 -134 -135 -136 -137 -138 -139 -140 -141 -142 -143 -144 -145 -146 -147 -148 -149 -150 -151 -152 -153 -154 -155
f1 (ppm)

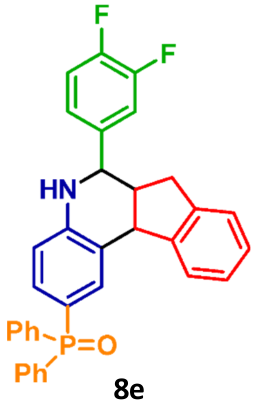
F
F
HN
Ph
P=O
Ph

8e

^{13}C-RMN

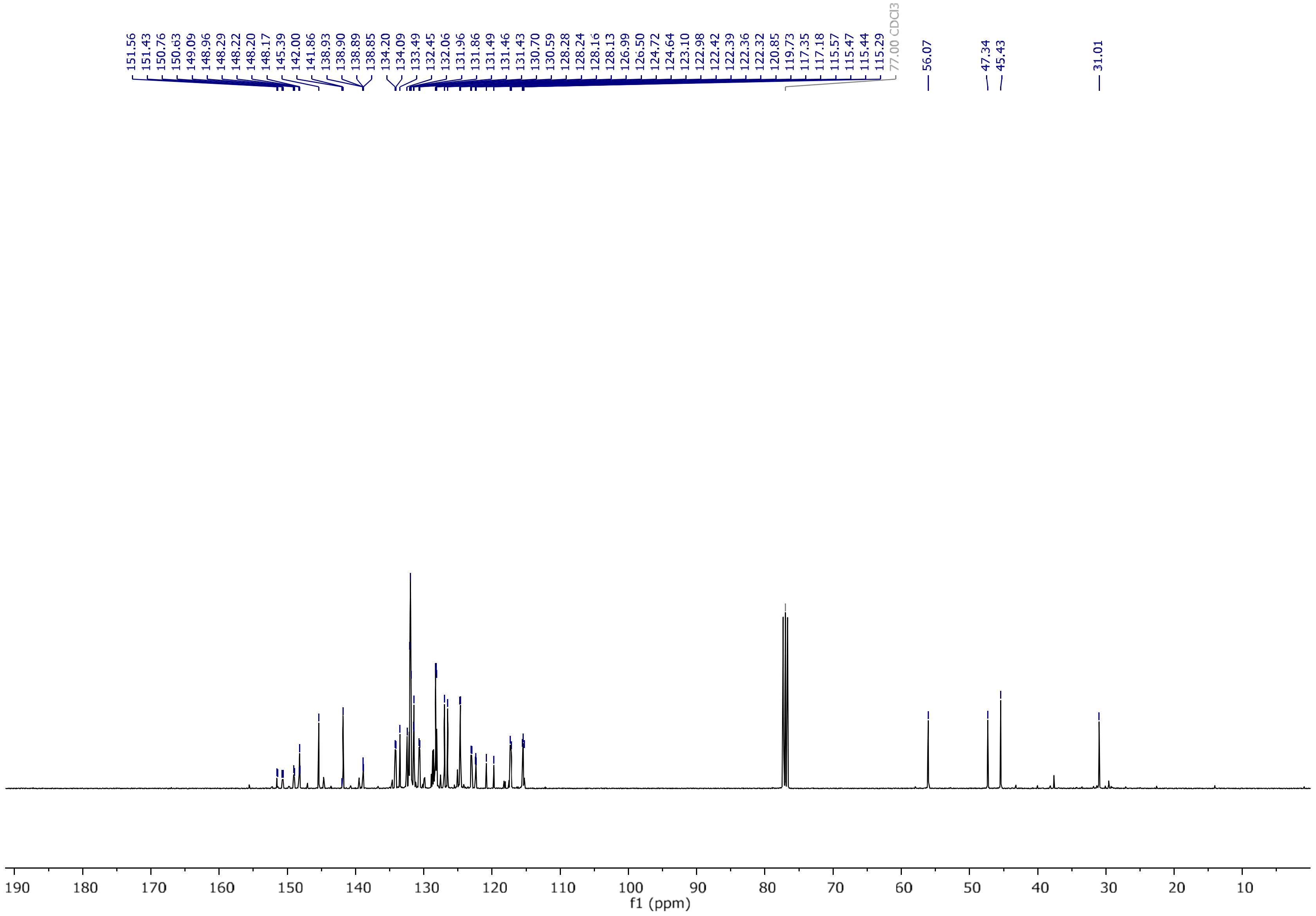
151.56
151.43
150.76
150.63
149.09
148.96
148.29
148.22
148.20
148.17
145.39
142.00
141.86
138.93
138.90
138.89
138.85
134.20
134.09
133.49
132.45
132.06
131.96
131.86
131.49
131.46
131.43
130.70
130.59
128.28
128.24
128.16
128.13
126.99
126.50
124.72
124.64
123.10
122.98
122.42
122.39
122.36
122.32
120.85
119.73
117.35
117.18
115.57
115.47
115.44
115.29
77.00 CDCl3
56.07
47.34
45.43
31.01
190 180 170 160 150 140 130 120 110 100 90 80 70 60 50 40 30 20 10
f1 (ppm)

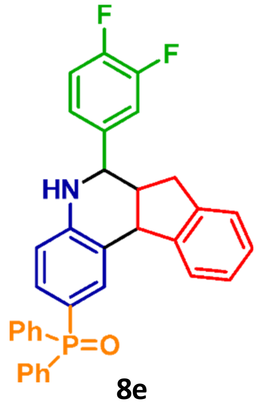
F
F
HN
Ph
P=O
Ph

8e

^{1}H-RMN

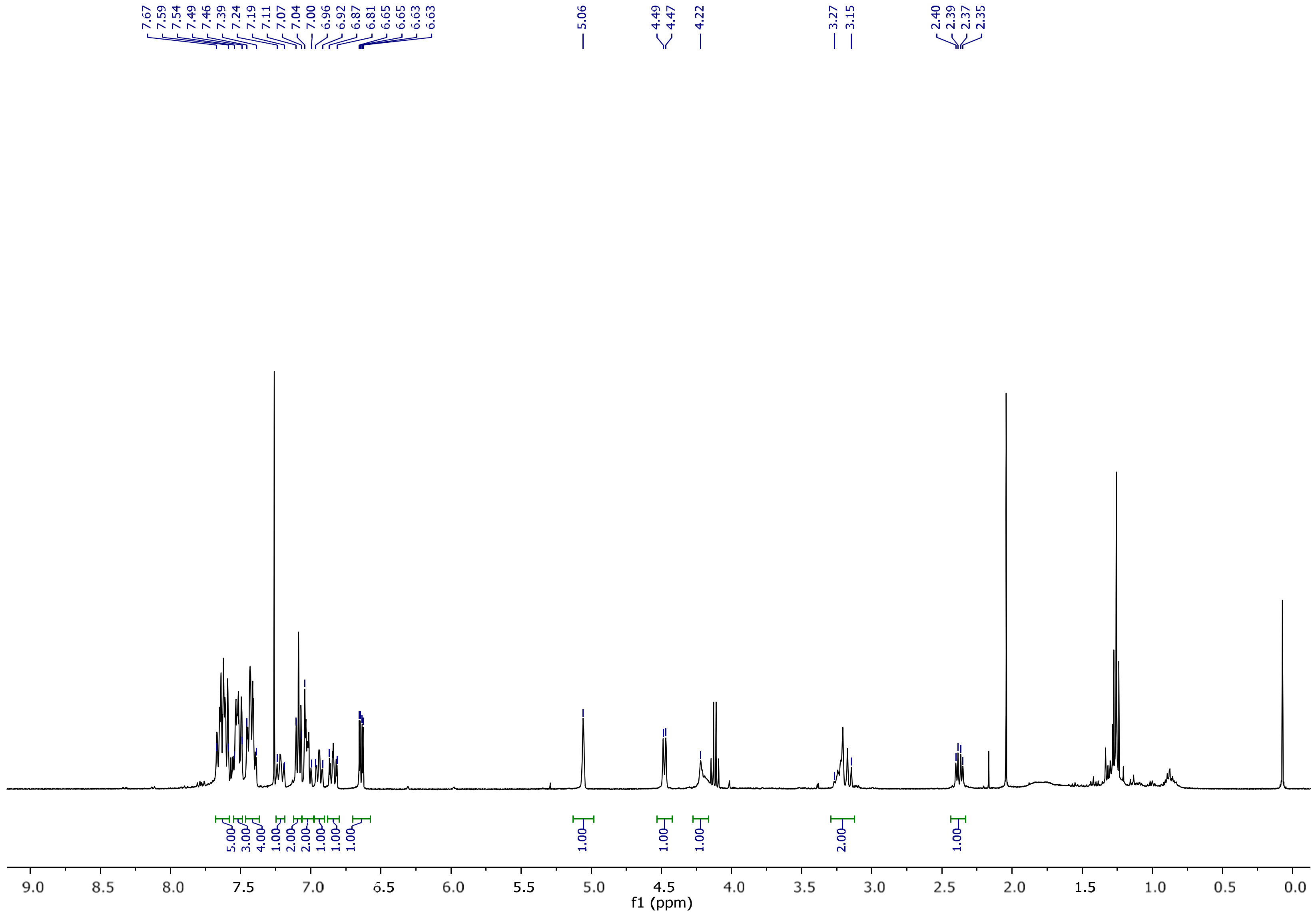

7.67 7.59 7.54 7.49 7.46 7.39 7.24 7.19 7.11 7.07 7.04 7.00 6.96 6.92 6.87 6.81 6.65 6.65 6.63 6.63
5.06
4.49 4.47
4.22
3.27 3.15
2.40 2.39 2.37 2.35
5.00 3.00 4.00 1.00 2.00 2.00 1.00 1.00 1.00 1.00
1.00
1.00
1.00
2.00
1.00
9.0 8.5 8.0 7.5 7.0 6.5 6.0 5.5 5.0 4.5 4.0 3.5 3.0 2.5 2.0 1.5 1.0 0.5 0.0
f1 (ppm)

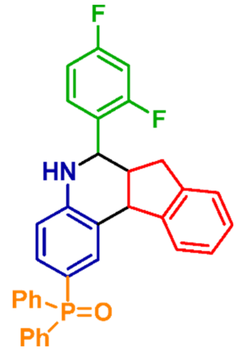

F
F
HN
Ph
P=O
Ph

8f

^{31}P-RMN

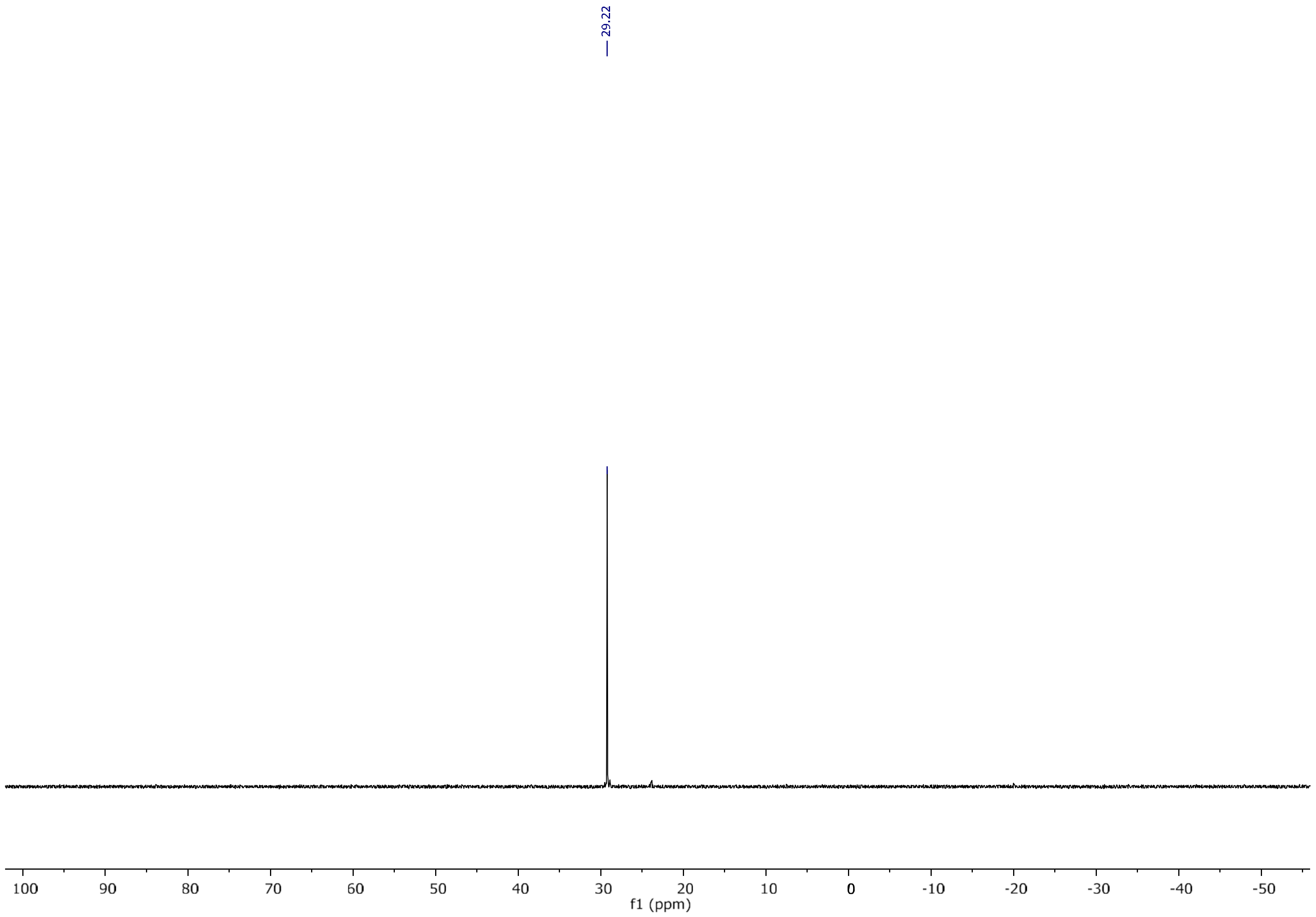
29.22
100 90 80 70 60 50 40 30 20 10 0 -10 -20 -30 -40 -50
f1 (ppm)

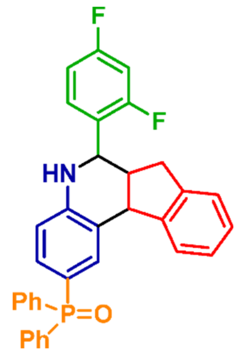
F
F
HN
Ph
P=O
Ph

8f

^{19}F-RMN

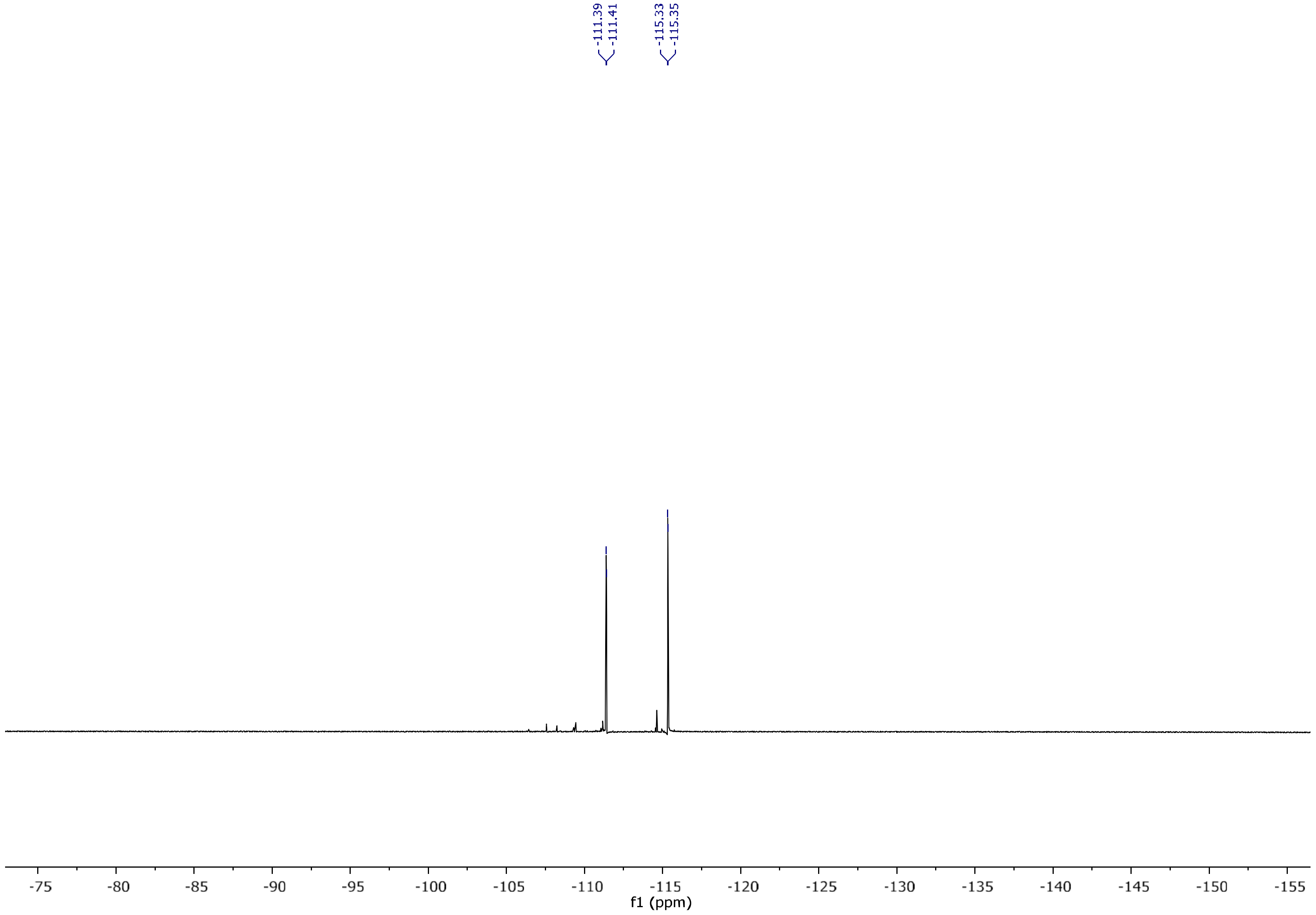

-111.39
-111.41
-115.33
-115.35
-75
-80
-85
-90
-95
-100
-105
-110
-115
-120
-125
-130
-135
-140
-145
-150
-155
f1 (ppm)

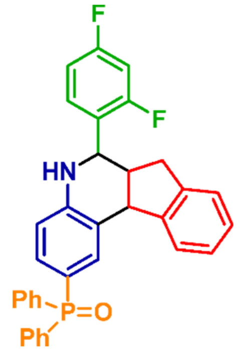

F
F
HN
Ph
P=O
Ph

8f

^{13}C-RMN

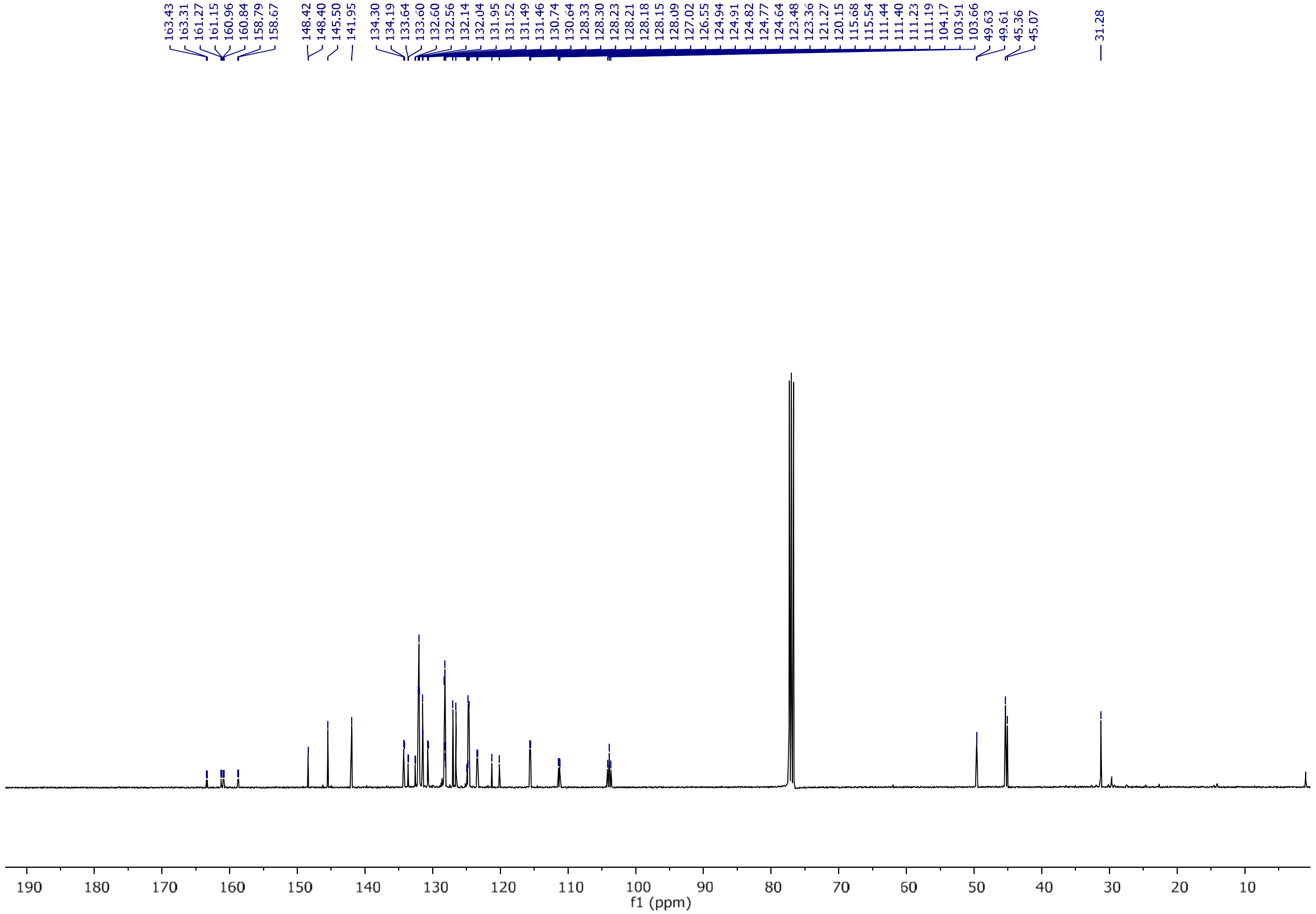

163.43
163.31
161.27
161.15
160.96
160.84
158.79
158.67
148.42
148.40
145.50
141.95
134.30
134.19
133.64
133.60
132.60
132.56
132.14
132.04
131.95
131.52
131.49
131.46
130.74
130.64
128.33
128.30
128.23
128.21
128.18
128.15
128.09
127.02
126.55
124.94
124.91
124.82
124.77
124.64
123.48
123.36
121.27
120.15
115.68
115.54
111.44
111.40
111.23
111.19
104.17
103.91
103.66
49.63
49.61
45.36
45.07
31.28
190 180 170 160 150 140 130 120 110 100 90 80 70 60 50 40 30 20 10
f1 (ppm)

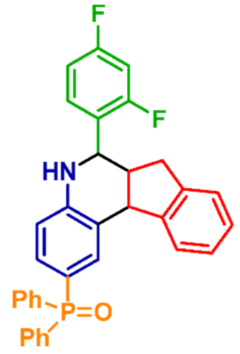

F
F
HN
Ph
P=O
Ph

8f

^{1}H-RMN

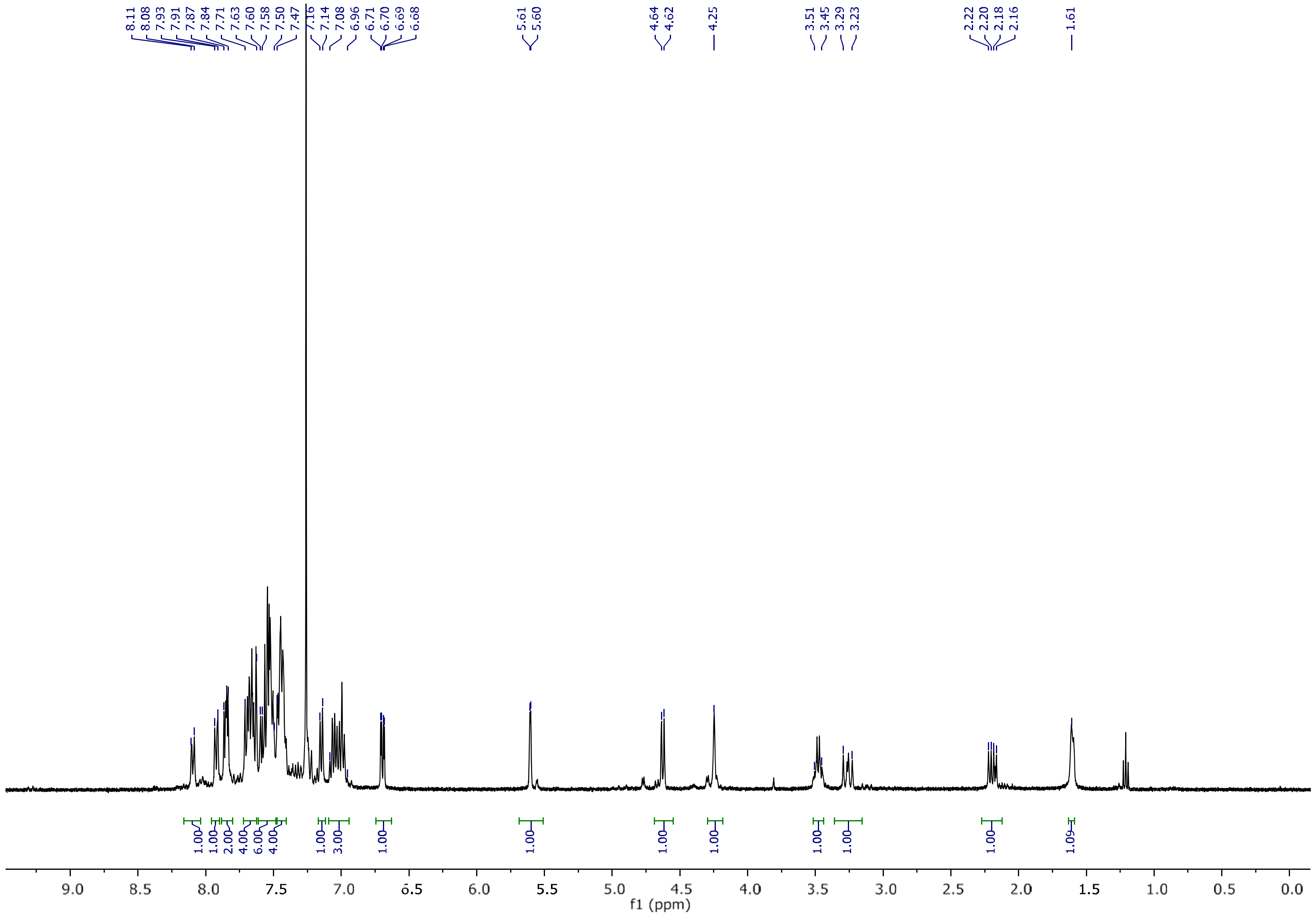

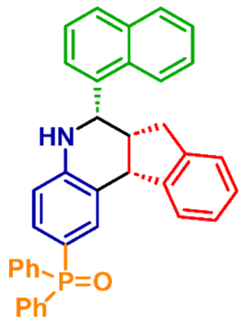

8g

^{31}P-RMN

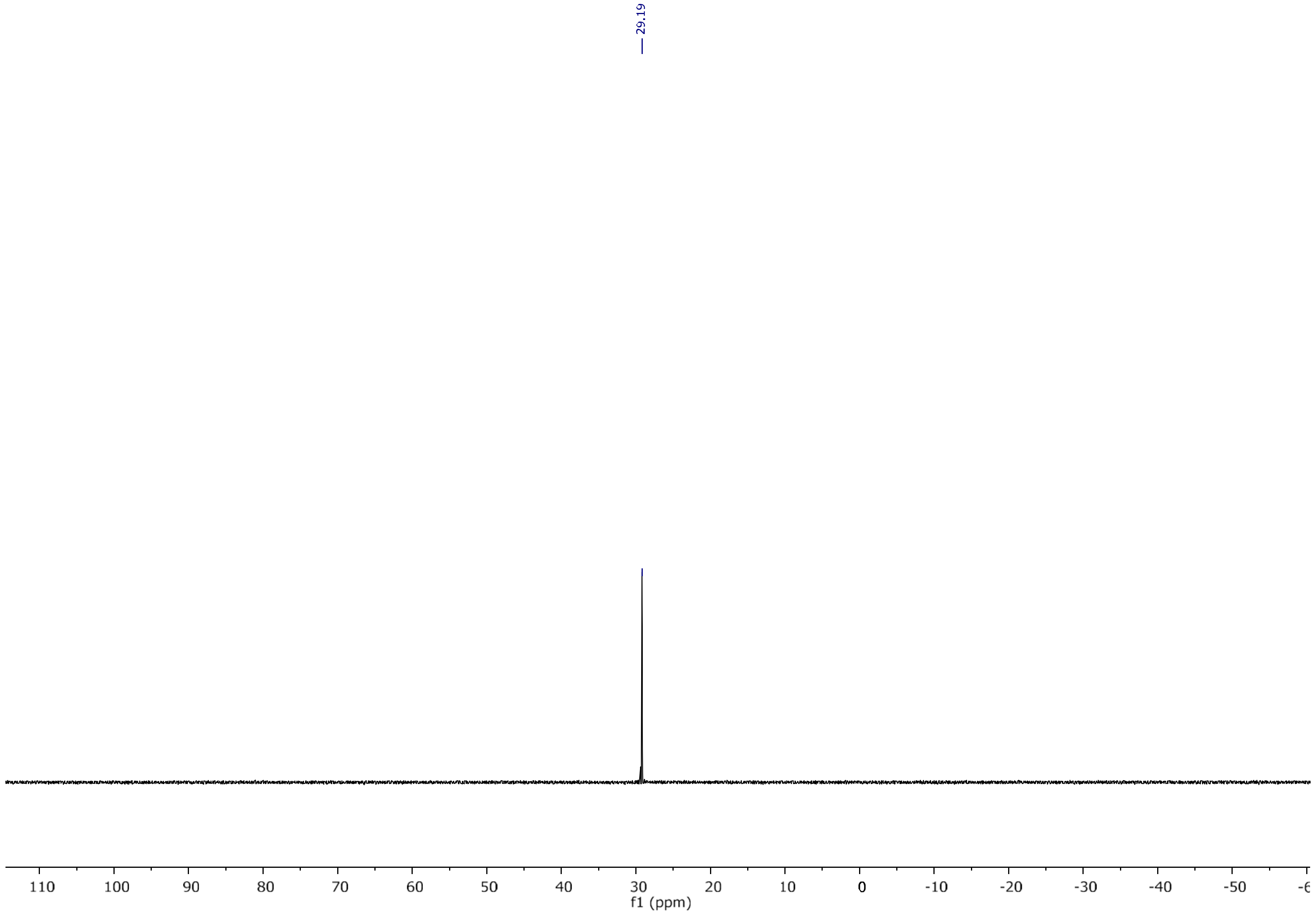
29.19
110
100
90
80
70
60
50
40
30
20
10
0
-10
-20
-30
-40
-50
f1 (ppm)

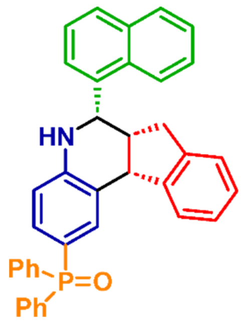
HN
Ph
P=O
Ph

8g

^{13}C-RMN

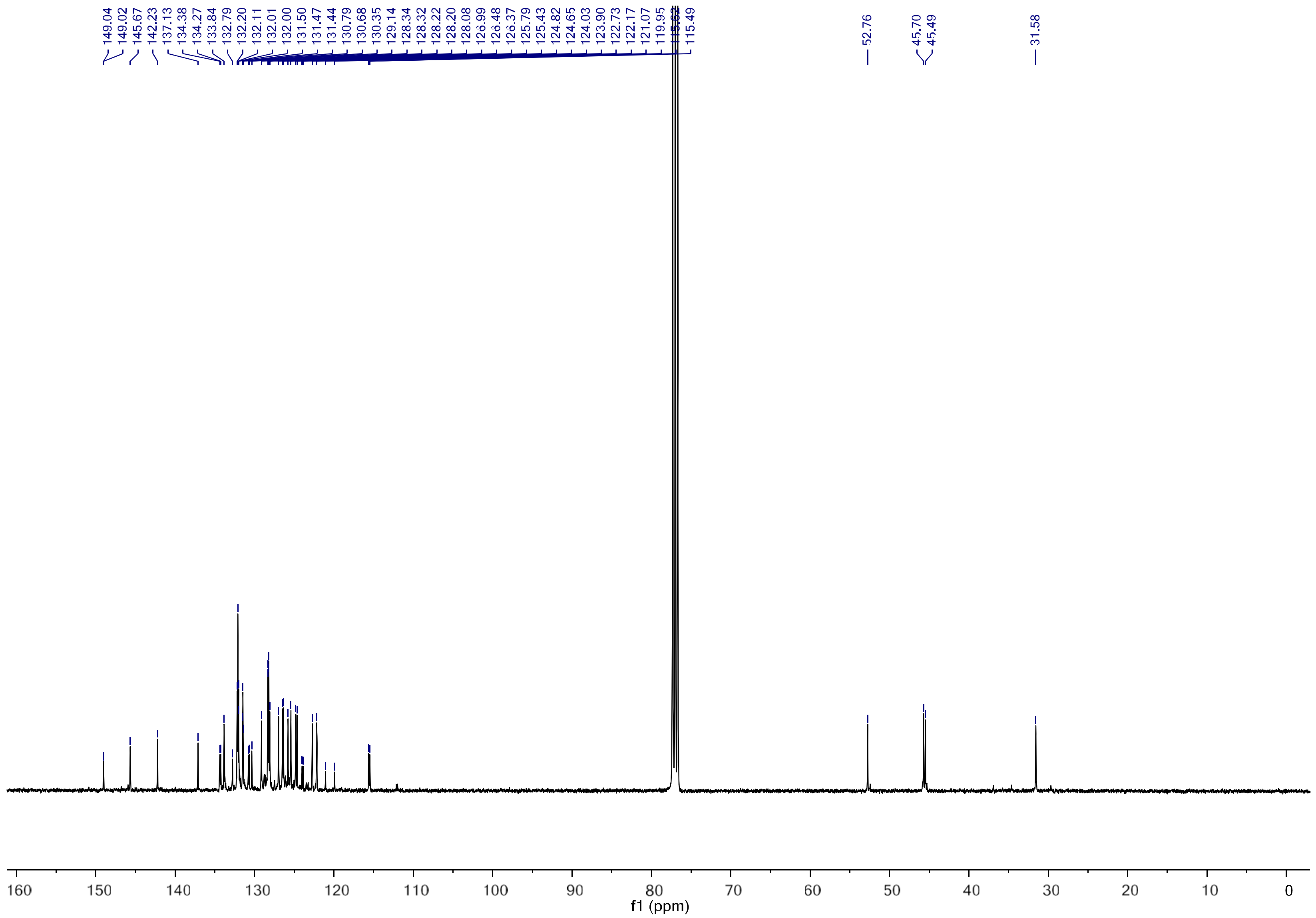
149.04
149.02
145.67
142.23
137.13
134.38
134.27
133.84
132.79
132.20
132.11
132.01
132.00
131.50
131.47
131.44
130.79
130.68
130.35
129.14
128.34
128.32
128.22
128.20
128.08
126.99
126.48
126.37
125.79
125.43
124.82
124.65
124.03
123.90
122.73
122.17
121.07
119.95
115.49
52.76
45.70
45.49
31.58
160 150 140 130 120 110 100 90 80 70 60 50 40 30 20 10 0
f1 (ppm)

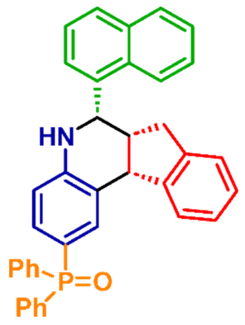
HN
Ph
P=O
Ph

8g

^{1}H-RMN

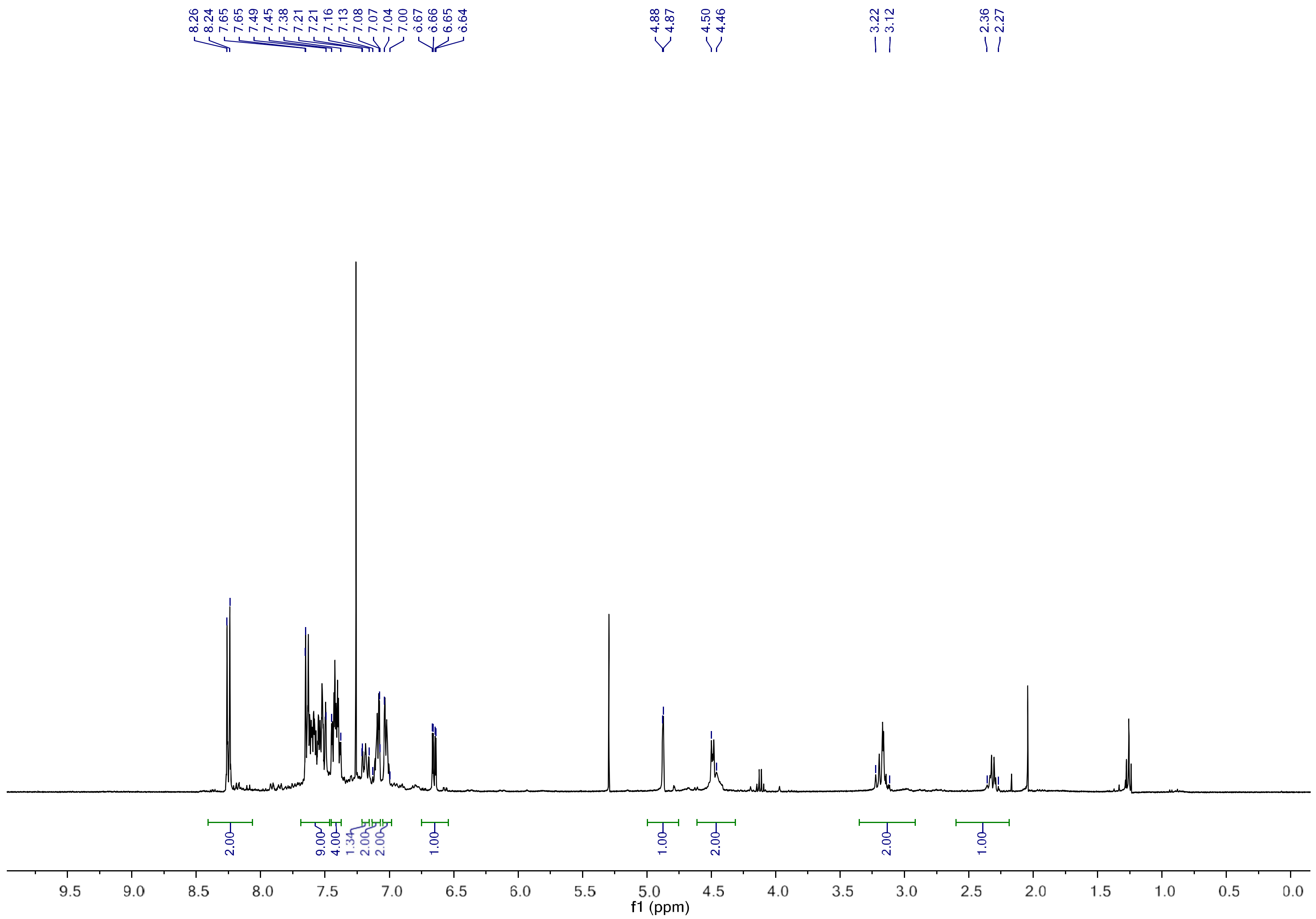

8.26
8.24
7.65
7.49
7.45
7.38
7.21
7.21
7.16
7.13
7.08
7.07
7.04
7.00
6.67
6.66
6.65
6.64
4.88
4.87
4.50
4.46
3.22
3.12
2.36
2.27
2.00
9.00
4.00
1.34
2.00
2.00
1.00
1.00
2.00
2.00
1.00
9.5 9.0 8.5 8.0 7.5 7.0 6.5 6.0 5.5 5.0 4.5 4.0 3.5 3.0 2.5 2.0 1.5 1.0 0.5 0.0
f1 (ppm)

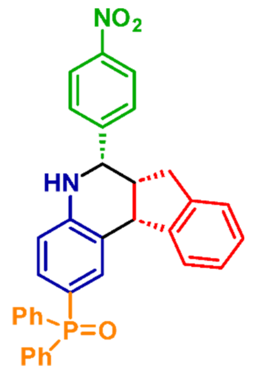

NO2
HN
Ph
P=O
Ph

8h

^{31}P-RMN

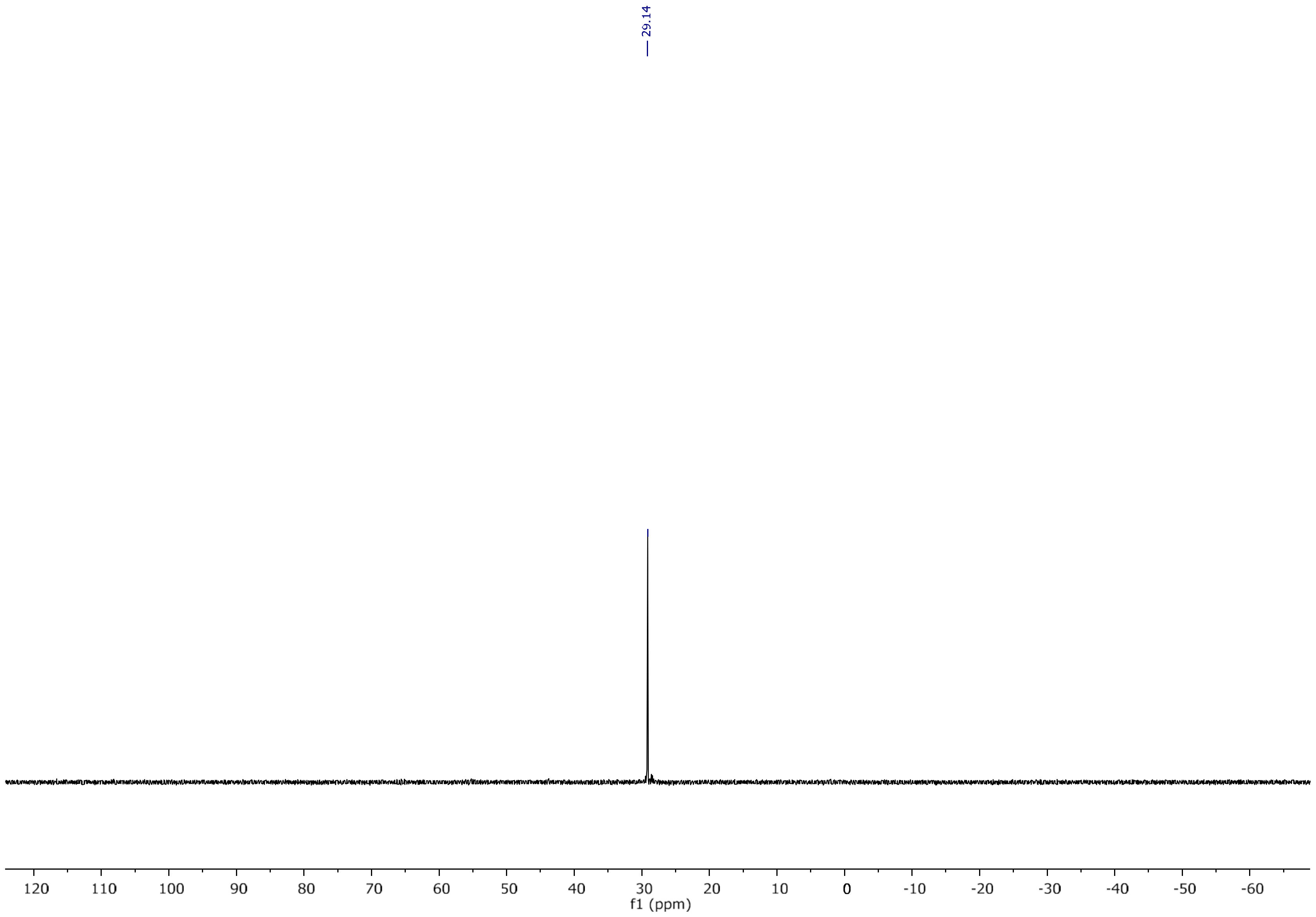
29.14
120
110
100
90
80
70
60
50
40
30
20
10
0
-10
-20
-30
-40
-50
-60
f1 (ppm)

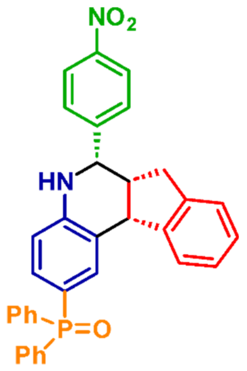
NO2
HN
Ph
P=O
Ph

8h

^{13}C-RMN

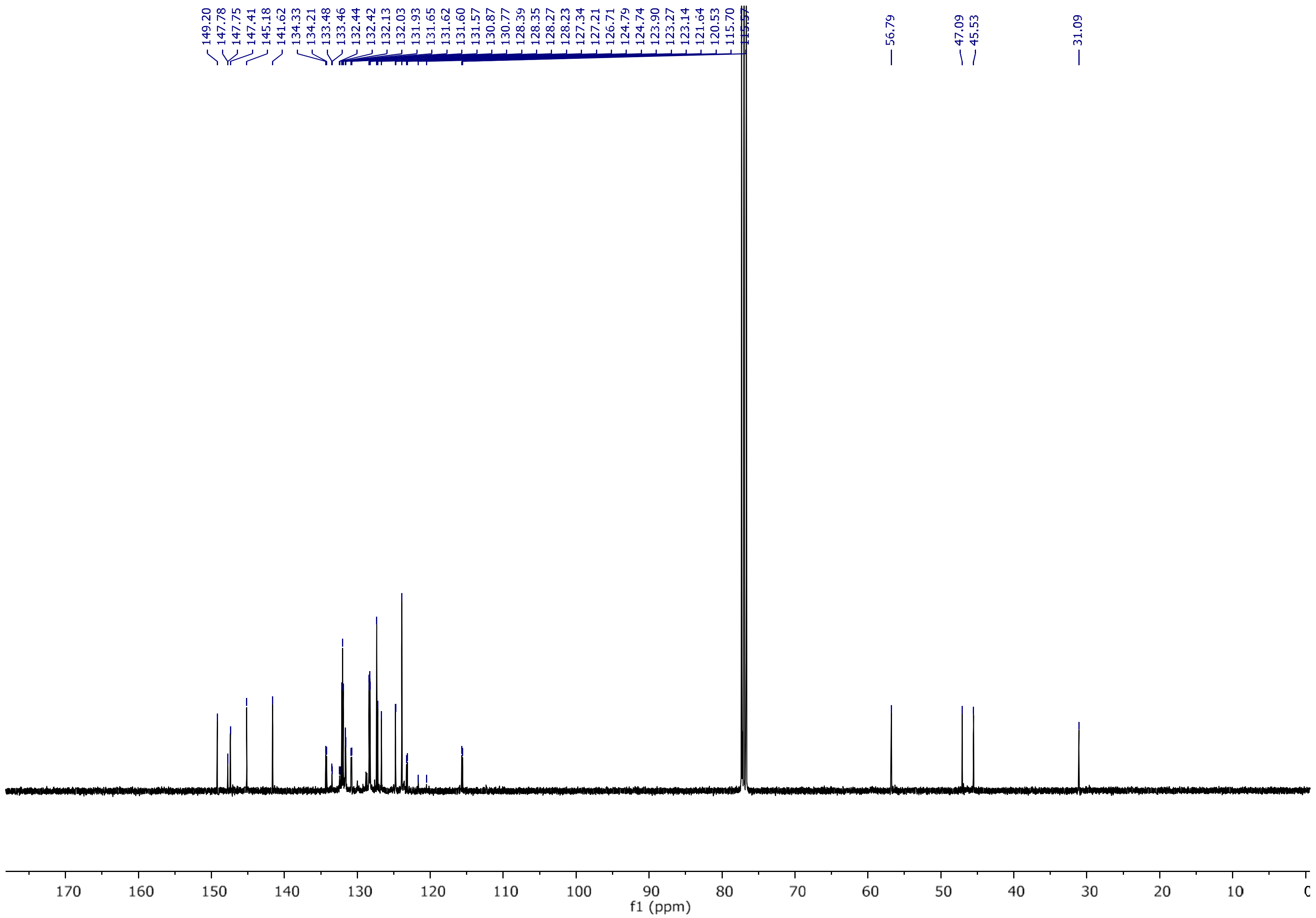
149.20
147.78
147.75
147.41
145.18
141.62
134.33
134.21
133.48
133.46
132.44
132.42
132.13
132.03
131.93
131.65
131.62
131.60
131.57
130.87
130.77
128.39
128.35
128.27
128.23
127.34
127.21
126.71
124.79
124.74
123.90
123.27
123.14
121.64
120.53
115.70
56.79
47.09
45.53
31.09
170
160
150
140
130
120
110
100
90
80
70
60
50
40
30
20
10
f1 (ppm)

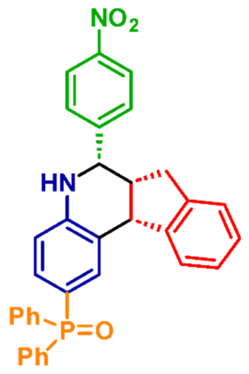
NO2
HN
Ph
P=O
Ph

8h

^{1}H-RMN

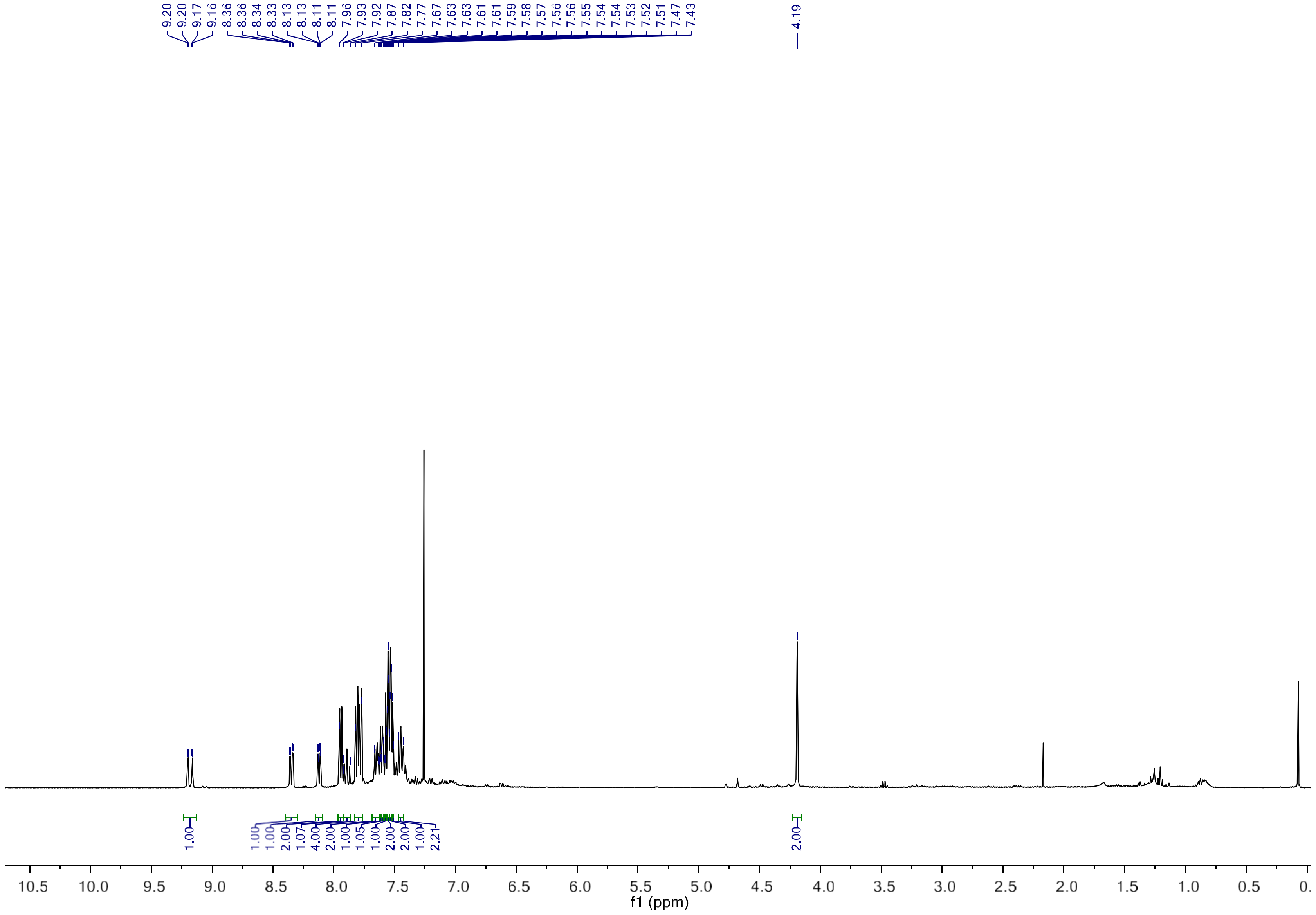

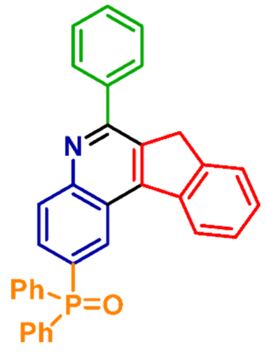

9a

^{31}P-RMN

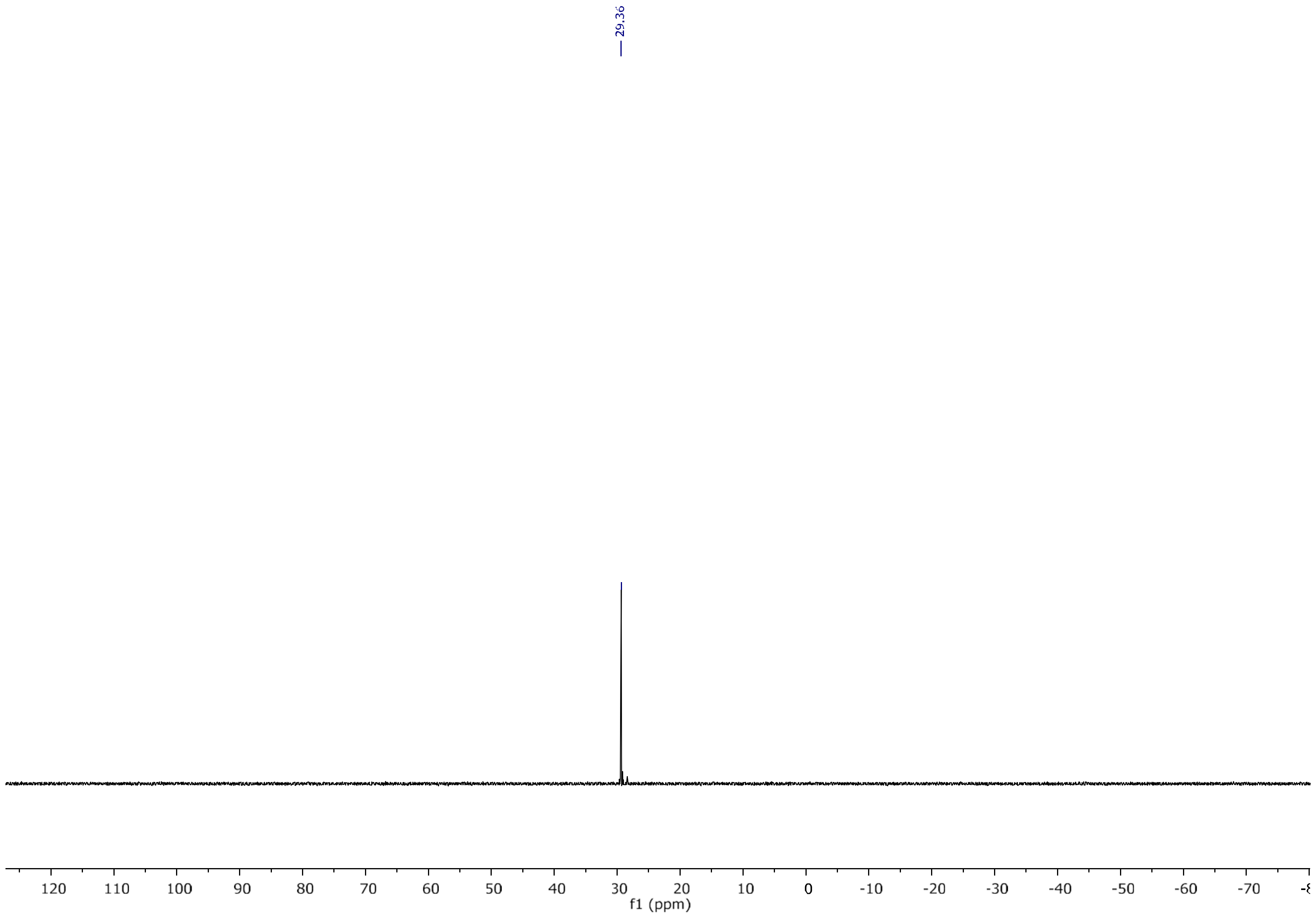
29.36
120 110 100 90 80 70 60 50 40 30 20 10 0 -10 -20 -30 -40 -50 -60 -70
f1 (ppm)

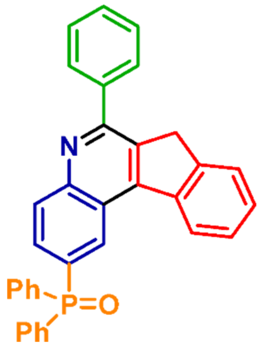
N
Ph
P=O
Ph

9a

^{13}C-RMN

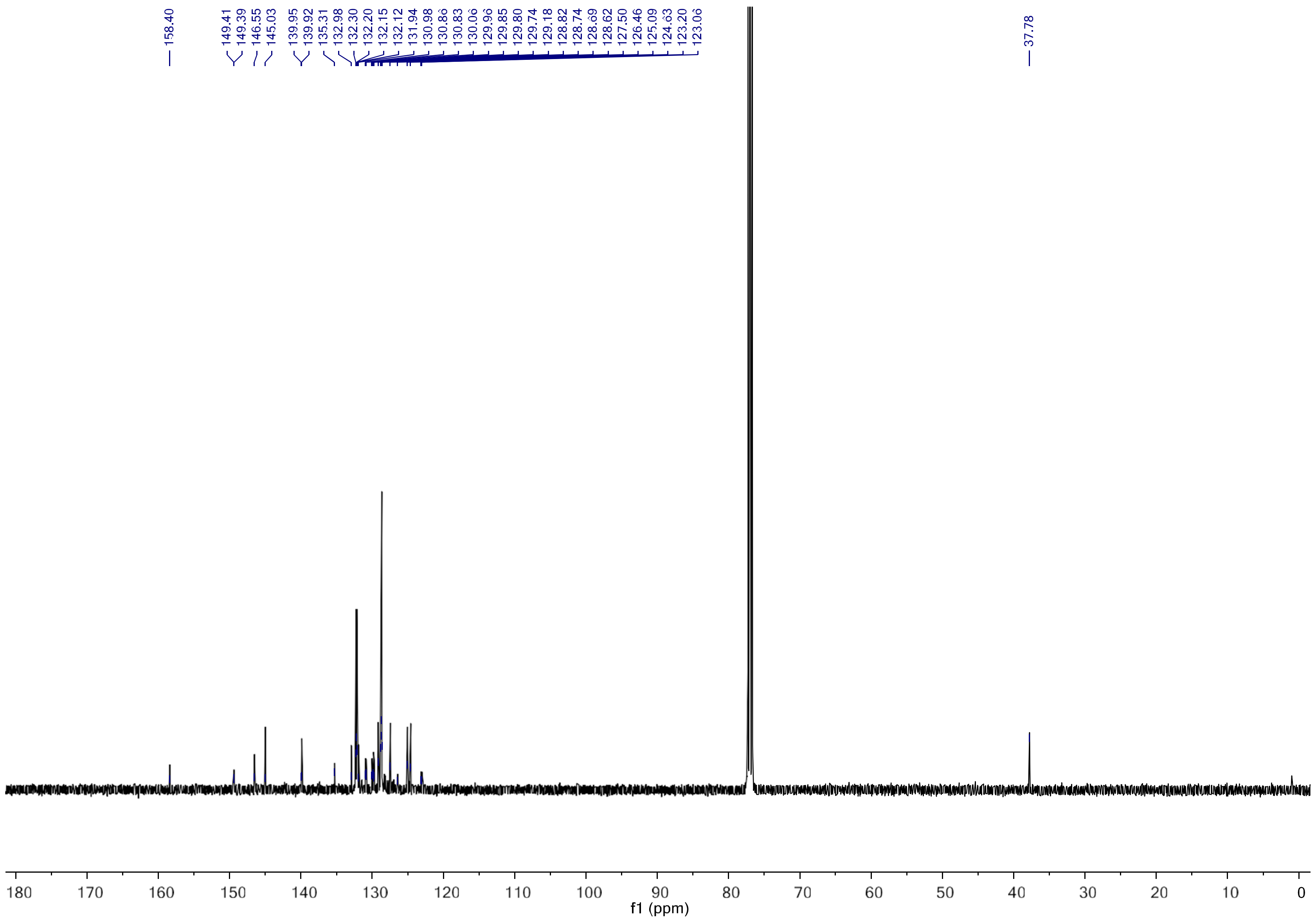
158.40
149.41
149.39
146.55
145.03
139.95
139.92
135.31
132.98
132.30
132.20
132.15
132.12
131.94
130.98
130.86
130.83
130.06
129.96
129.85
129.80
129.74
129.18
128.82
128.74
128.69
128.62
127.50
126.46
125.09
124.63
123.20
123.06
37.78
180 170 160 150 140 130 120 110 100 90 80 70 60 50 40 30 20 10 0
f1 (ppm)

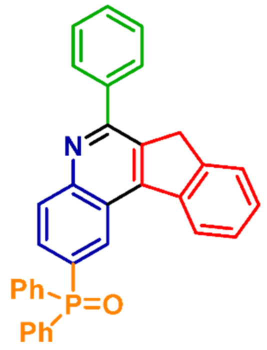
N
Ph
P=O
Ph

9a

^{1}H-RMN

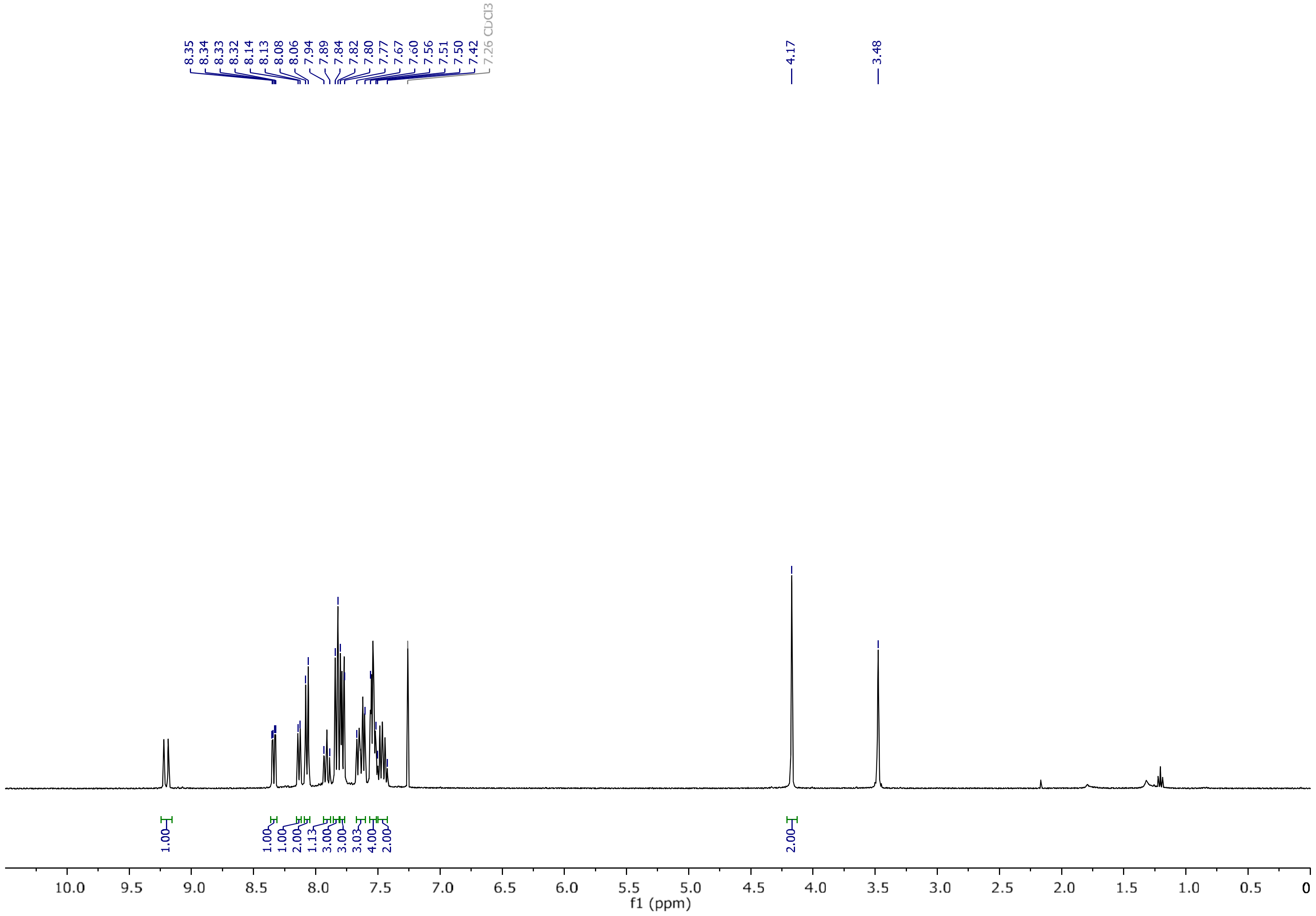
8.35
8.34
8.33
8.32
8.14
8.13
8.08
8.06
7.94
7.89
7.84
7.82
7.80
7.77
7.67
7.60
7.56
7.51
7.50
7.42
7.26 CDCl3
4.17
3.48
1.00
1.00
1.00
2.00
1.13
3.00
3.00
3.03
4.00
2.00
2.00
10.0
9.5
9.0
8.5
8.0
7.5
7.0
6.5
6.0
5.5
5.0
4.5
4.0
3.5
3.0
2.5
2.0
1.5
1.0
0.5
0
f1 (ppm)

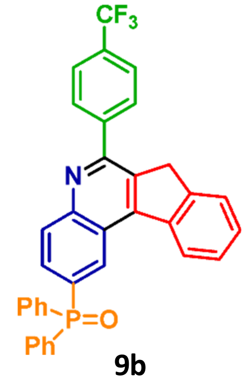
CF3
N
Ph
P=O
Ph

9b

^{31}P-RMN

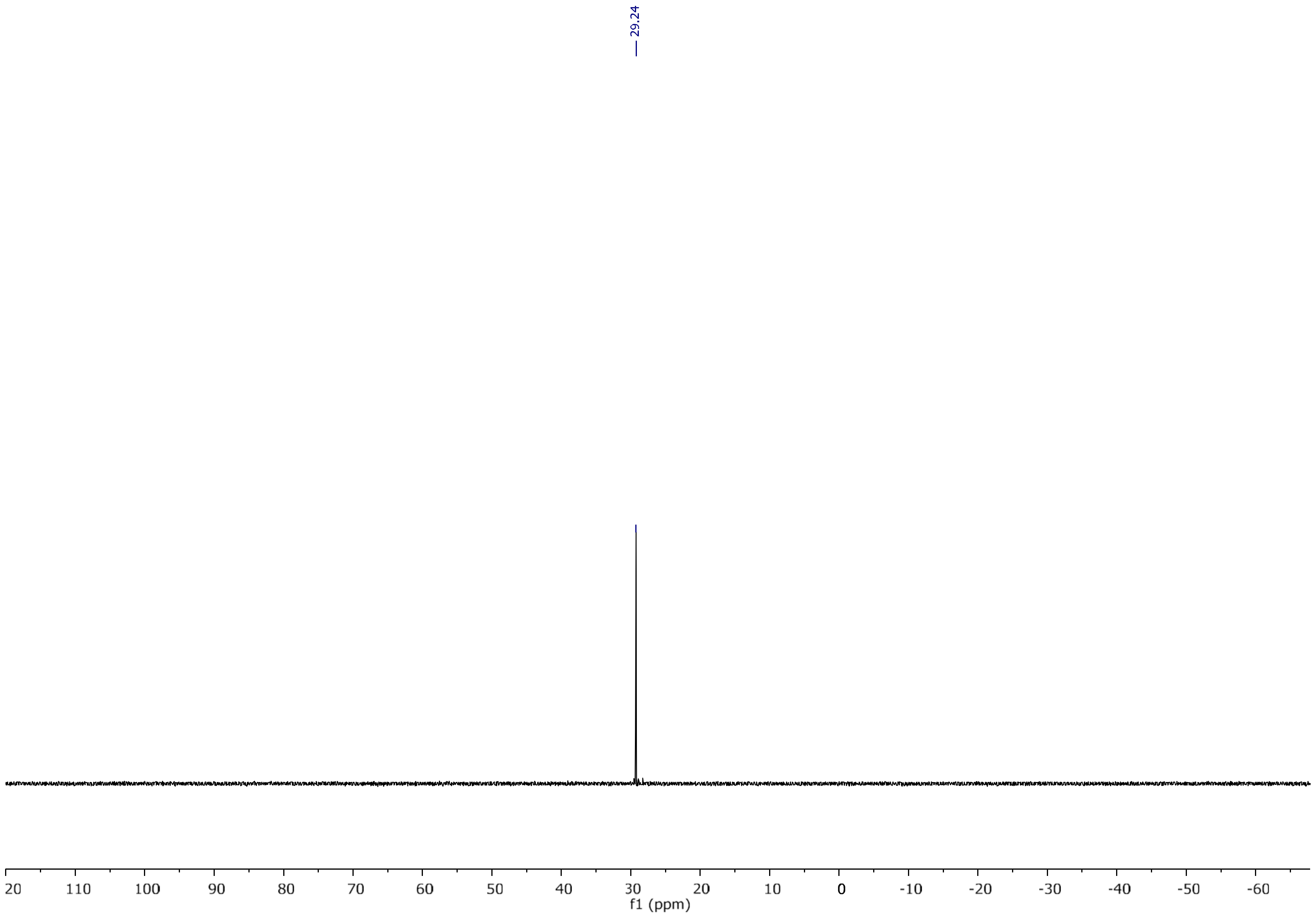

29,24
20 110 100 90 80 70 60 50 40 30 20 10 0 -10 -20 -30 -40 -50 -60
f1 (ppm)

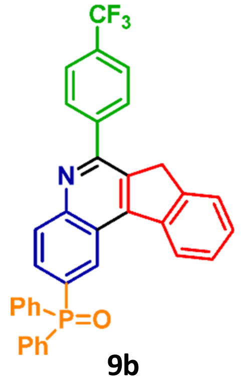

CF3
N
Ph
P=O
Ph

9b

^{19}F-RMN

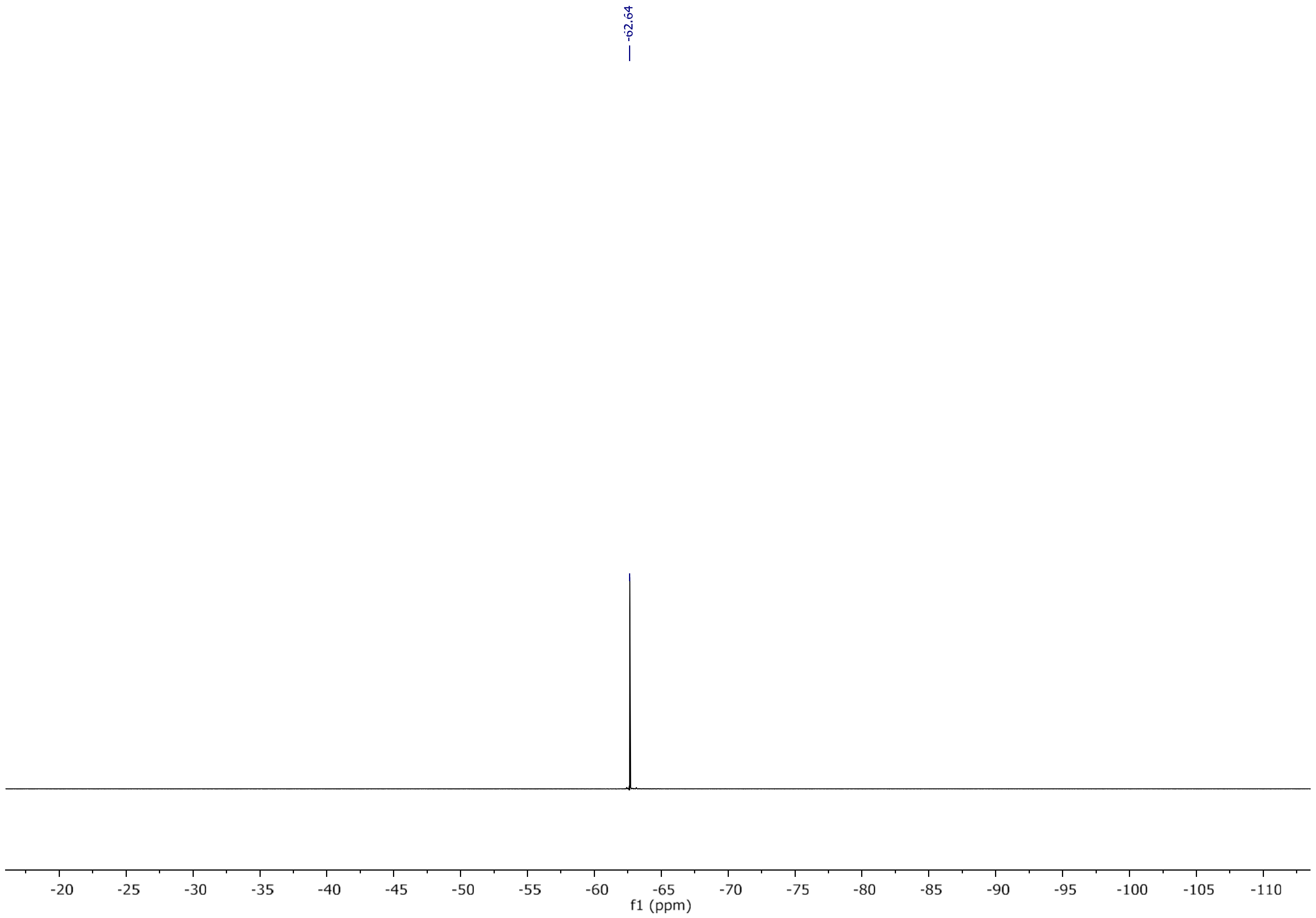

-62.64
-20 -25 -30 -35 -40 -45 -50 -55 -60 -65 -70 -75 -80 -85 -90 -95 -100 -105 -110
f1 (ppm)

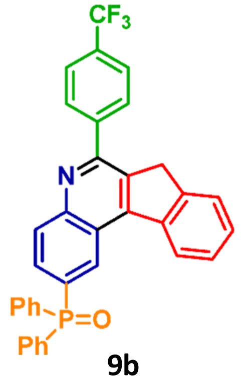

CF3
N
Ph
P=O
Ph

9b

^{13}C-RMN

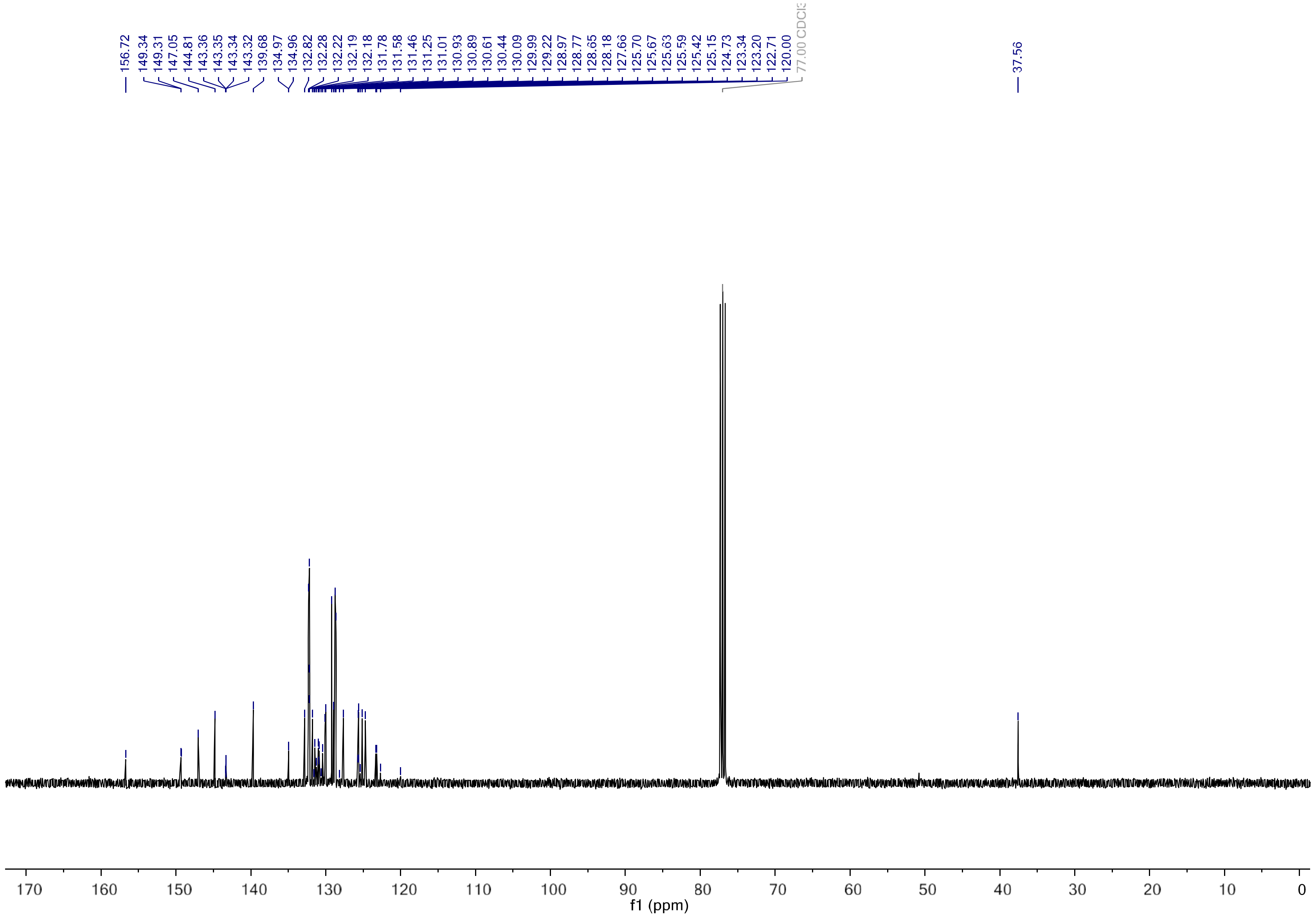
156.72
149.34
149.31
147.05
144.81
143.36
143.35
143.34
143.32
139.68
134.97
134.96
132.82
132.28
132.22
132.19
132.18
131.78
131.58
131.46
131.25
131.01
130.93
130.89
130.61
130.44
130.09
129.99
129.22
128.97
128.77
128.65
128.18
127.66
125.70
125.67
125.63
125.59
125.42
125.15
124.73
123.34
123.20
122.71
120.00
77.00 CDCl3
37.56
170 160 150 140 130 120 110 100 90 80 70 60 50 40 30 20 10 0
f1 (ppm)

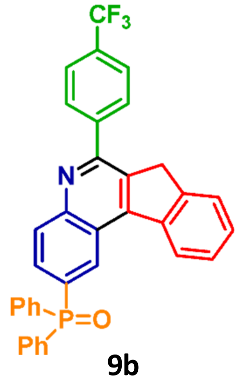
CF3
N
Ph
P=O
Ph

9b

^{1}H-RMN

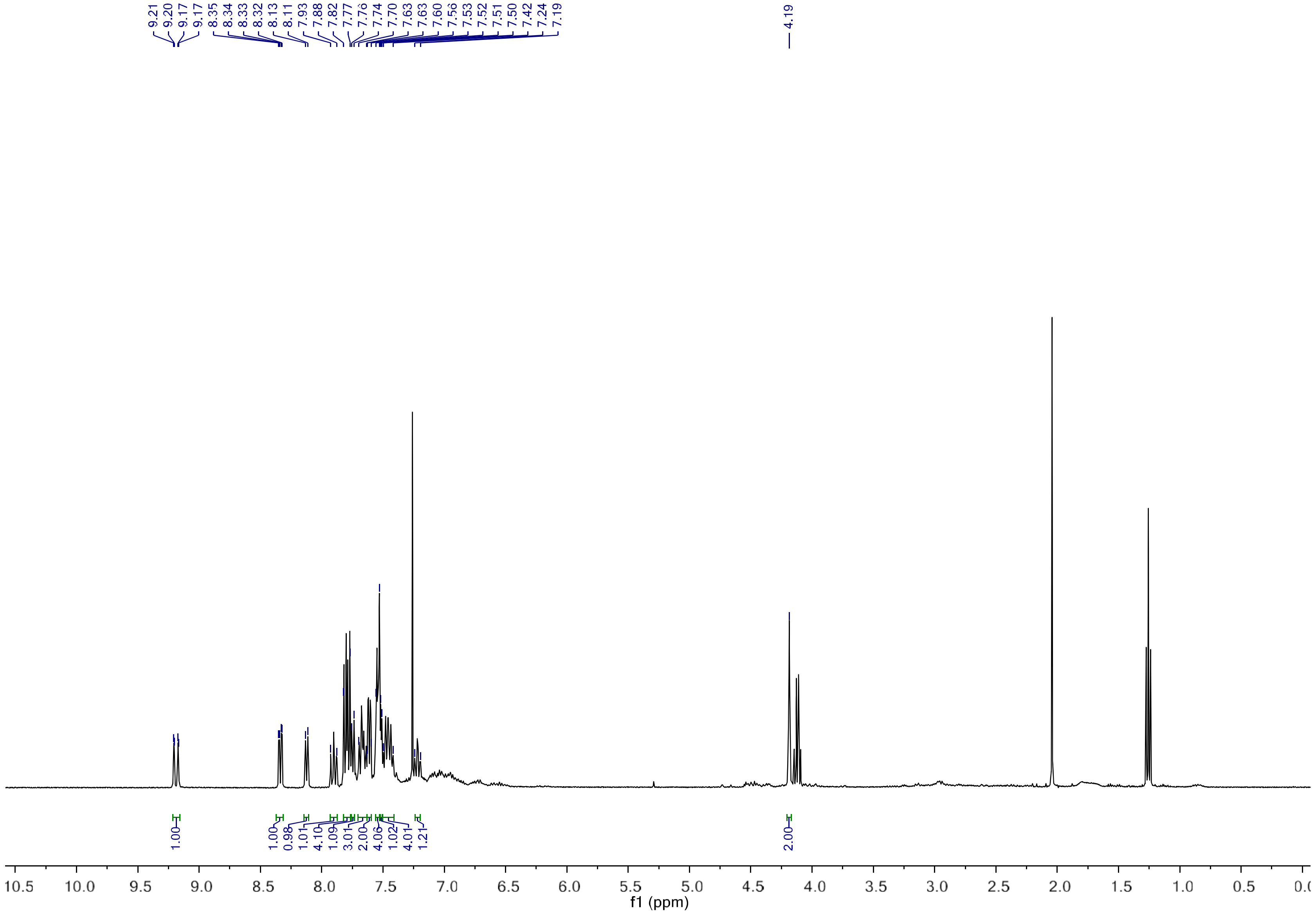

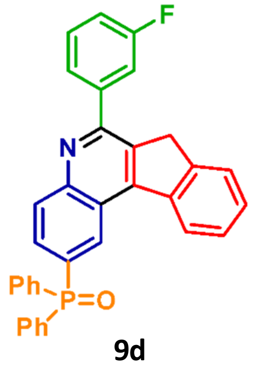

9d

^{31}P-RMN

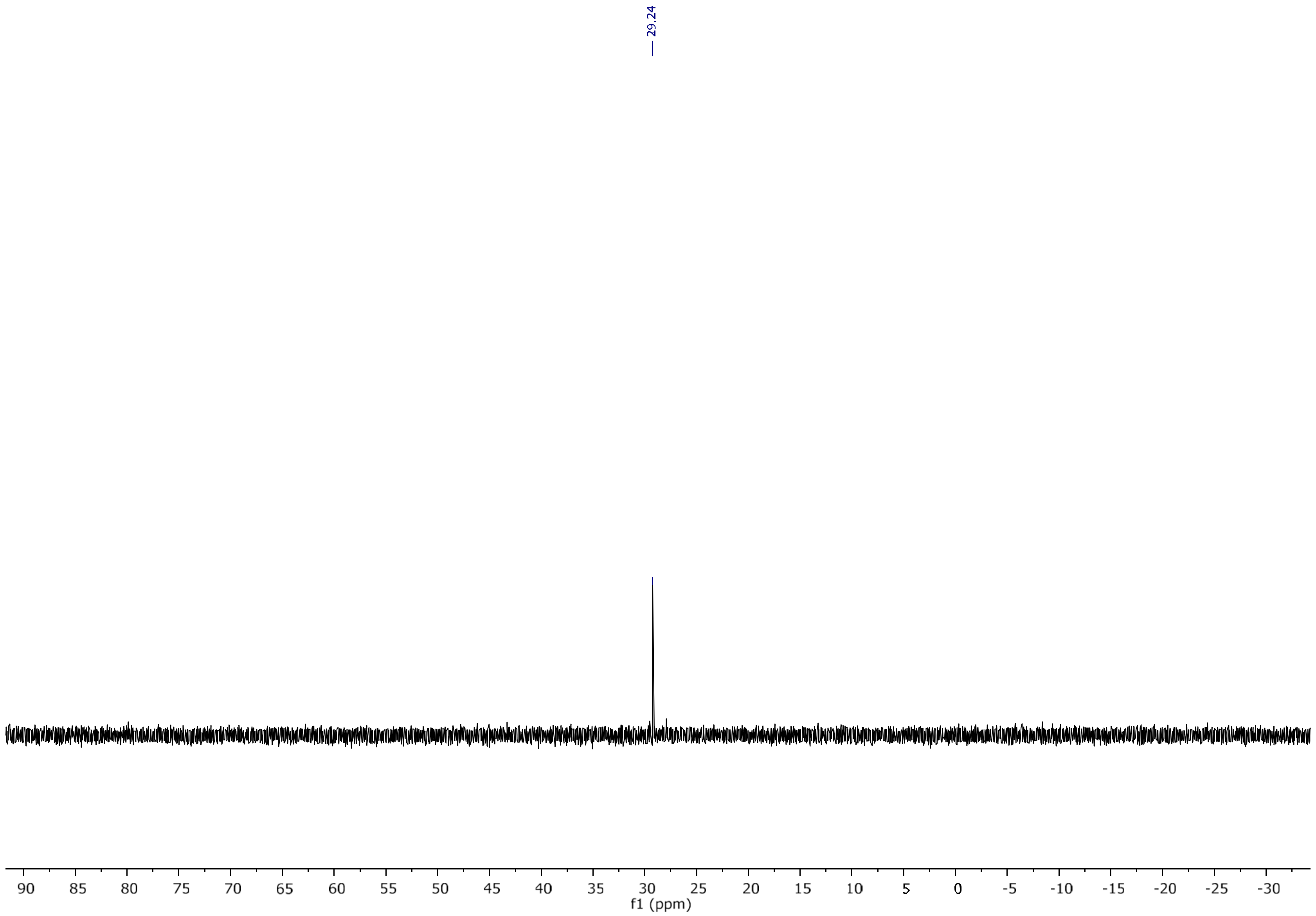
29.24
90 85 80 75 70 65 60 55 50 45 40 35 30 25 20 15 10 5 0 -5 -10 -15 -20 -25 -30
f1 (ppm)

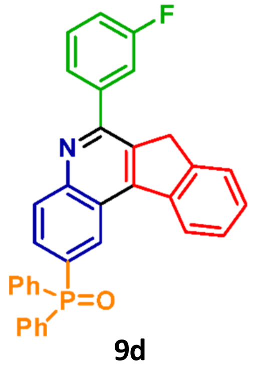
F
N
Ph
P=O
Ph

9d

^{19}F-RMN

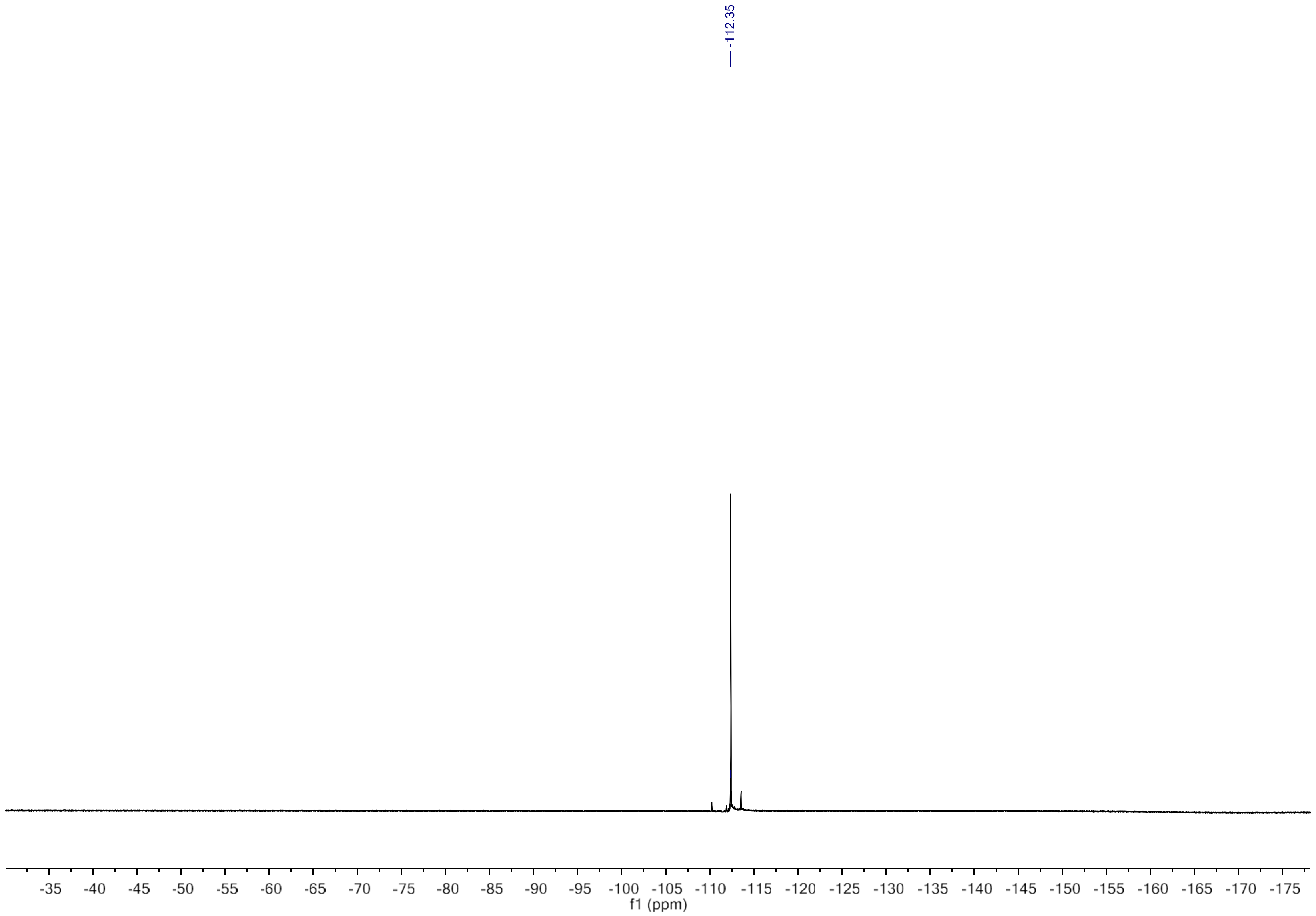
-112.35
-35
-40
-45
-50
-55
-60
-65
-70
-75
-80
-85
-90
-95
-100
-105
-110
-115
-120
-125
-130
-135
-140
-145
-150
-155
-160
-165
-170
-175
f1 (ppm)

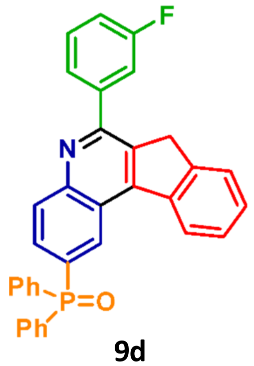
F
N
Ph
P=O
Ph

9d

^{13}C-RMN

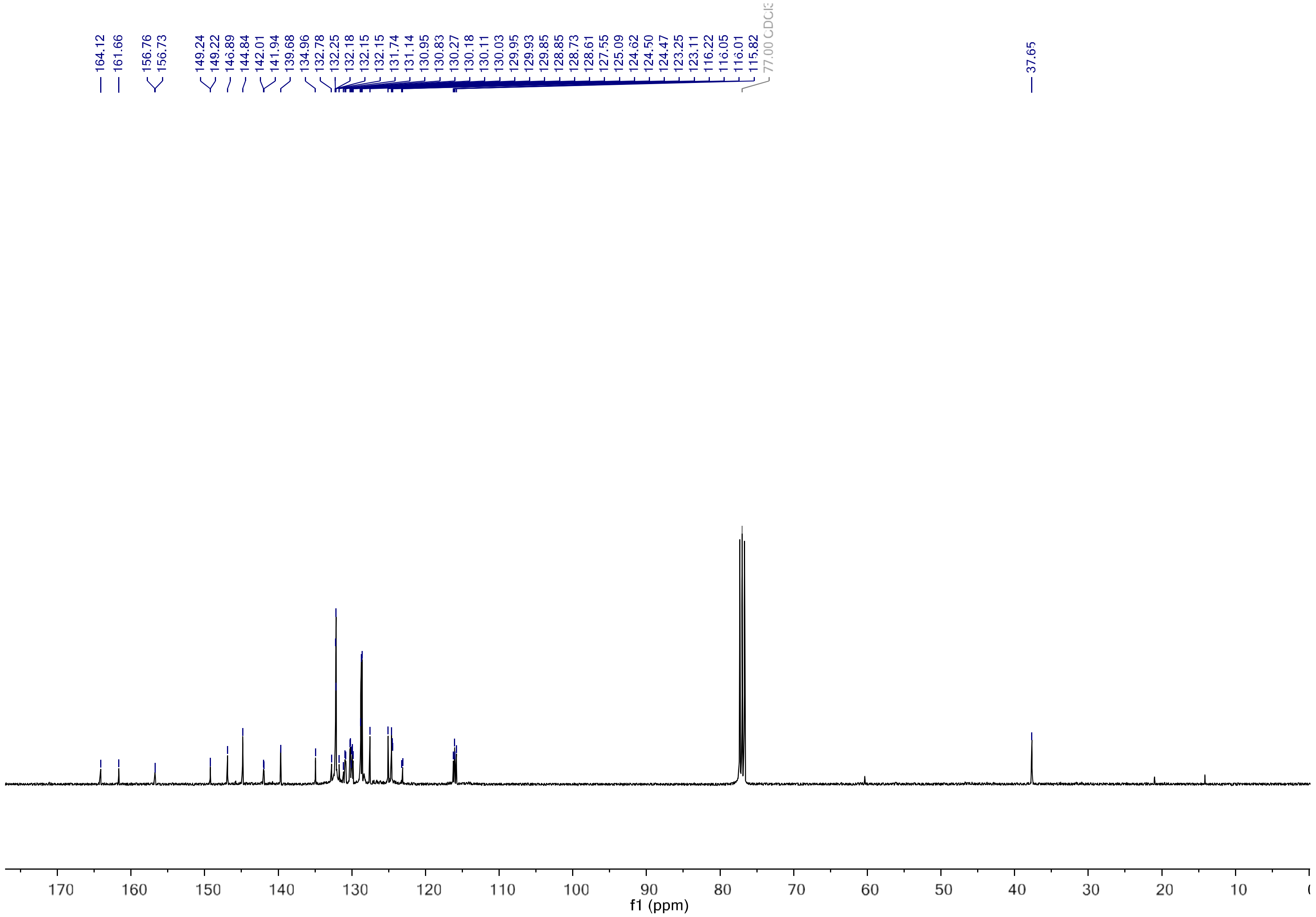
164.12
161.66
156.76
156.73
149.24
149.22
146.89
144.84
142.01
141.94
139.68
134.96
132.78
132.25
132.18
132.15
132.15
131.74
131.14
130.95
130.83
130.27
130.18
130.11
130.03
129.95
129.93
129.85
128.85
128.73
128.61
127.55
125.09
124.62
124.50
124.47
123.25
123.11
116.22
116.05
116.01
115.82
77.00 CDCl3
37.65
170
160
150
140
130
120
110
100
90
80
70
60
50
40
30
20
10
f1 (ppm)

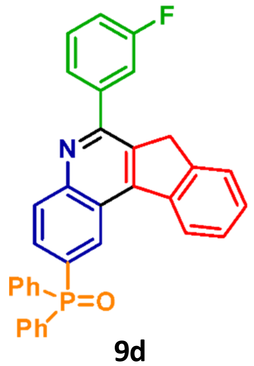
F
N
Ph
P=O
Ph

9d

^{1}H-RMN

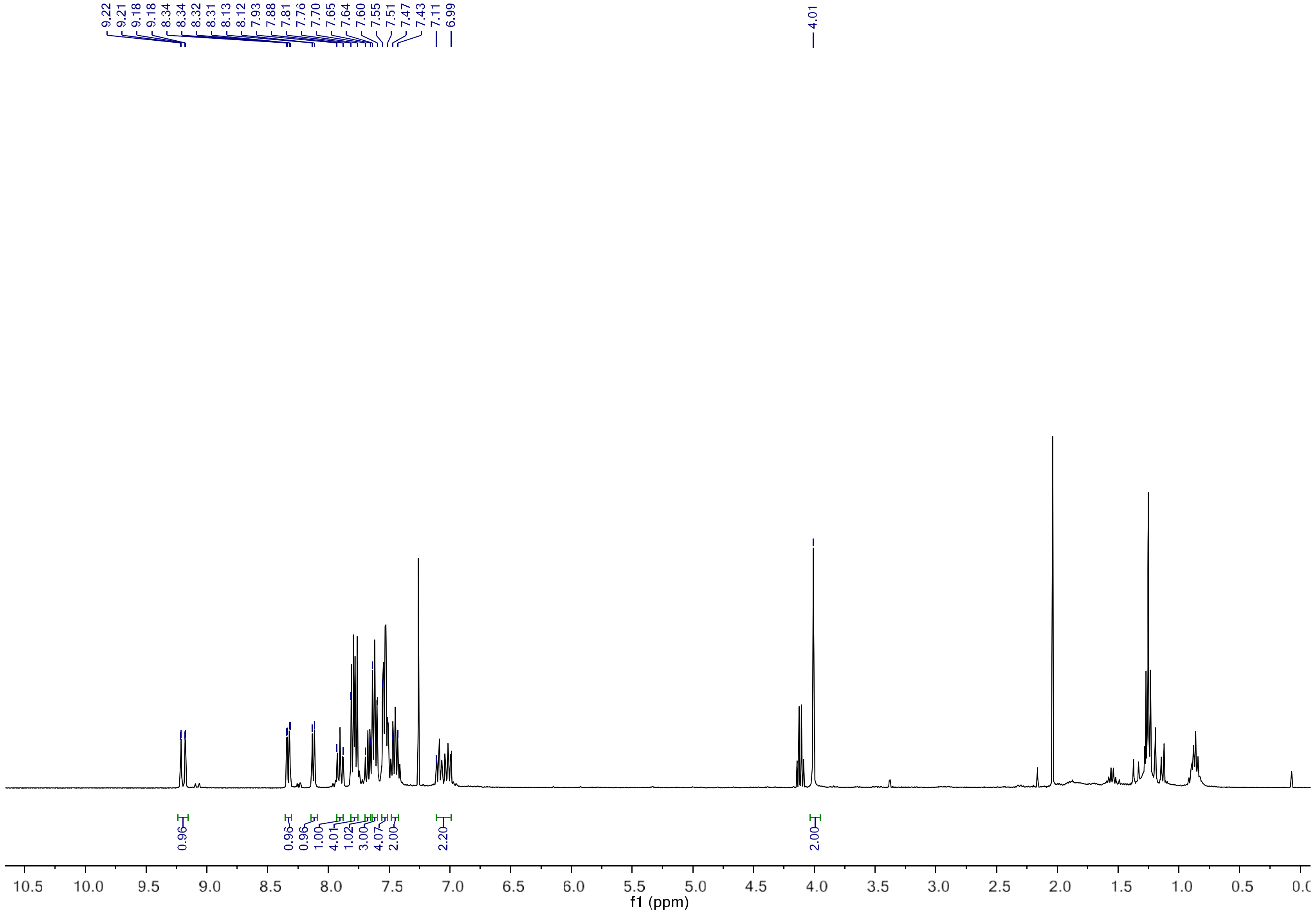

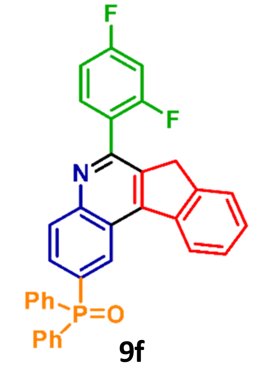

9f

^{31}P-RMN

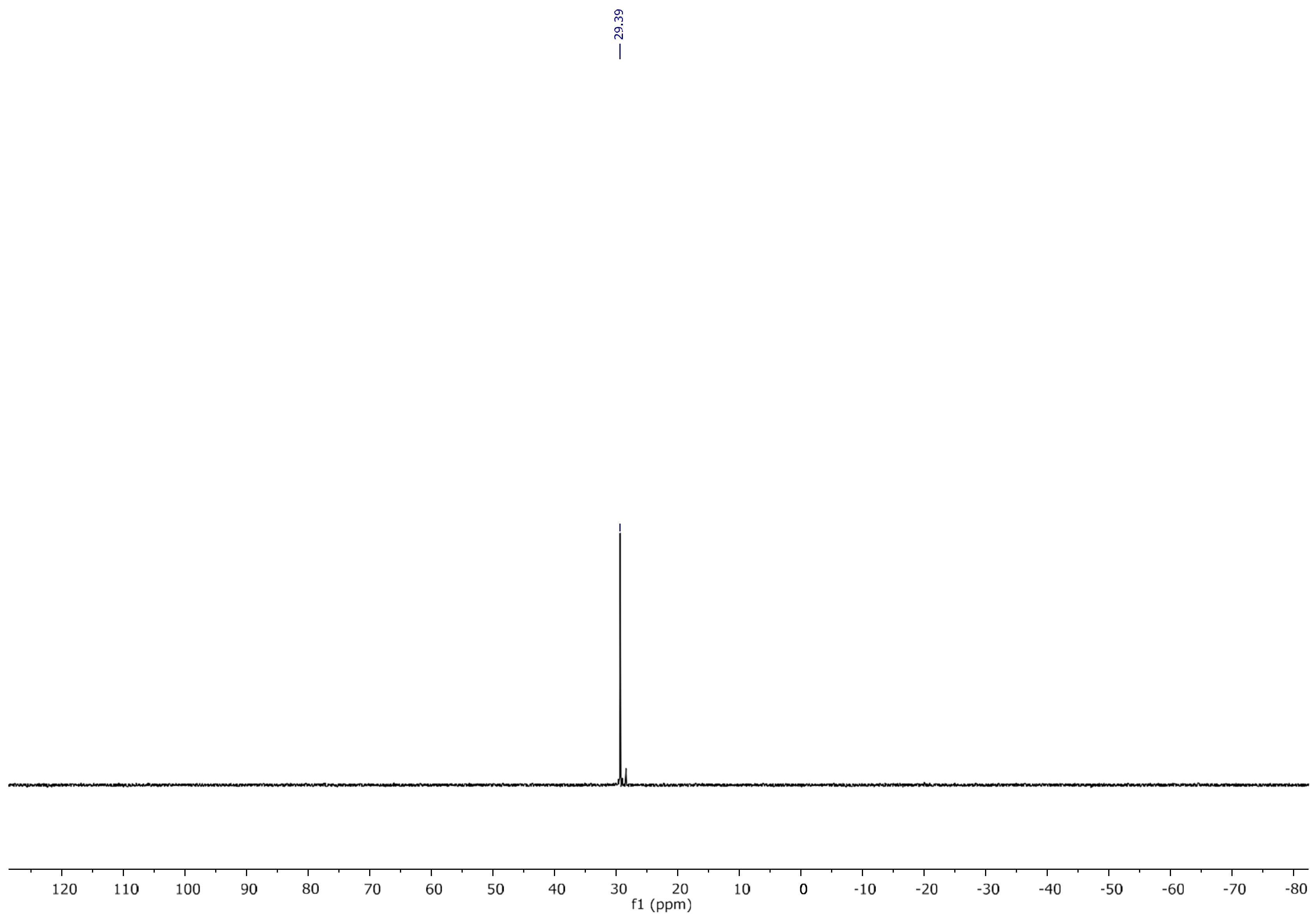

29.39
120
110
100
90
80
70
60
50
40
30
20
10
0
-10
-20
-30
-40
-50
-60
-70
-80
f1 (ppm)

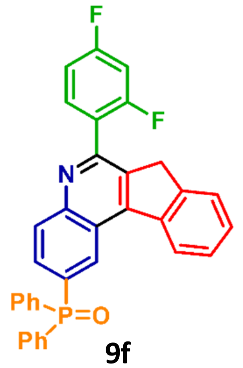

F
F
N
Ph
P=O
Ph

9f

^{19}F-RMN

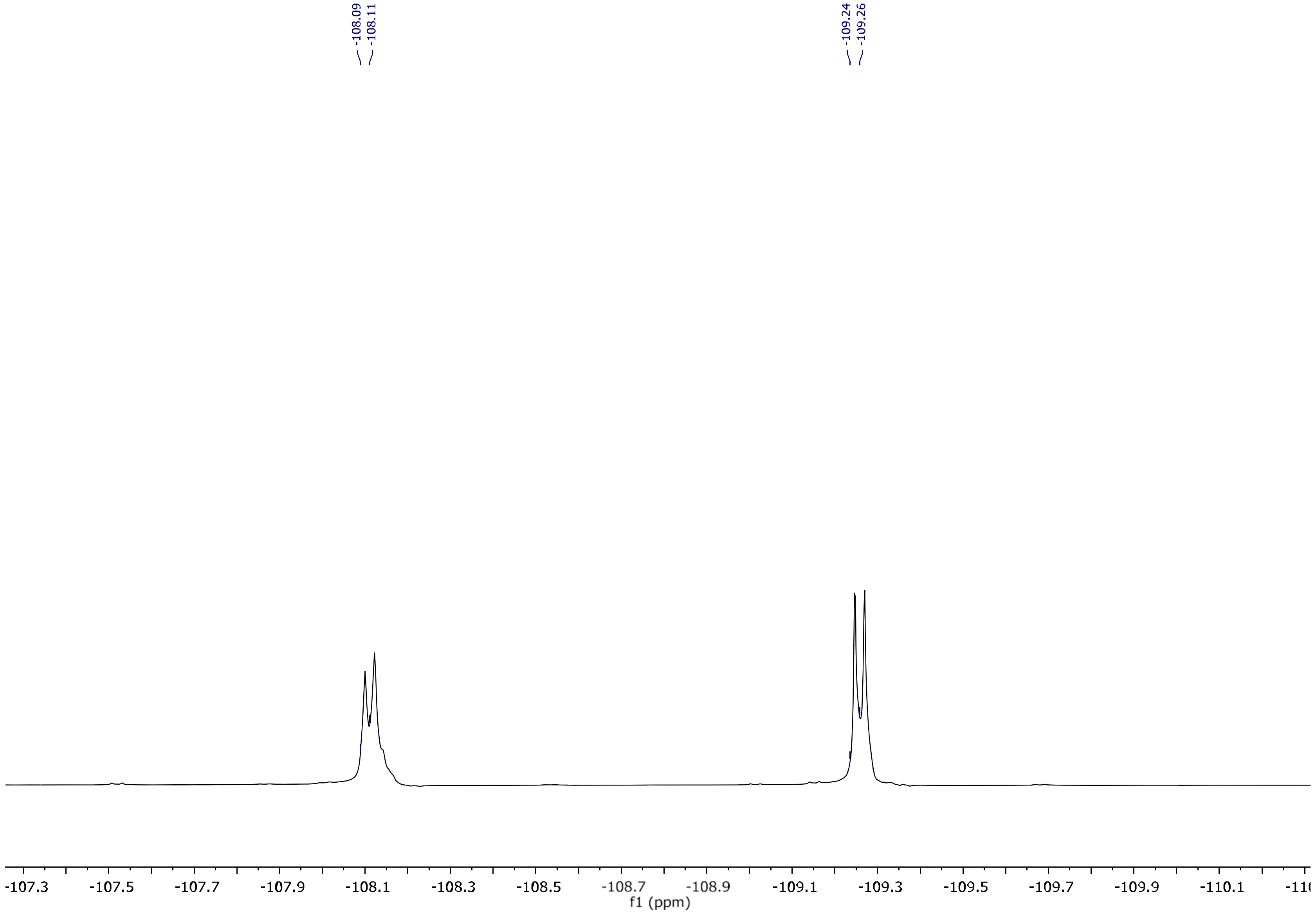

-108.09
-108.11
-109.24
-109.26
-107.3
-107.5
-107.7
-107.9
-108.1
-108.3
-108.5
-108.7
-108.9
-109.1
-109.3
-109.5
-109.7
-109.9
-110.1
f1 (ppm)

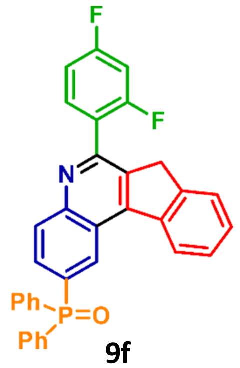

F
F
N
Ph
P=O
Ph

9f

^{13}C-RMN

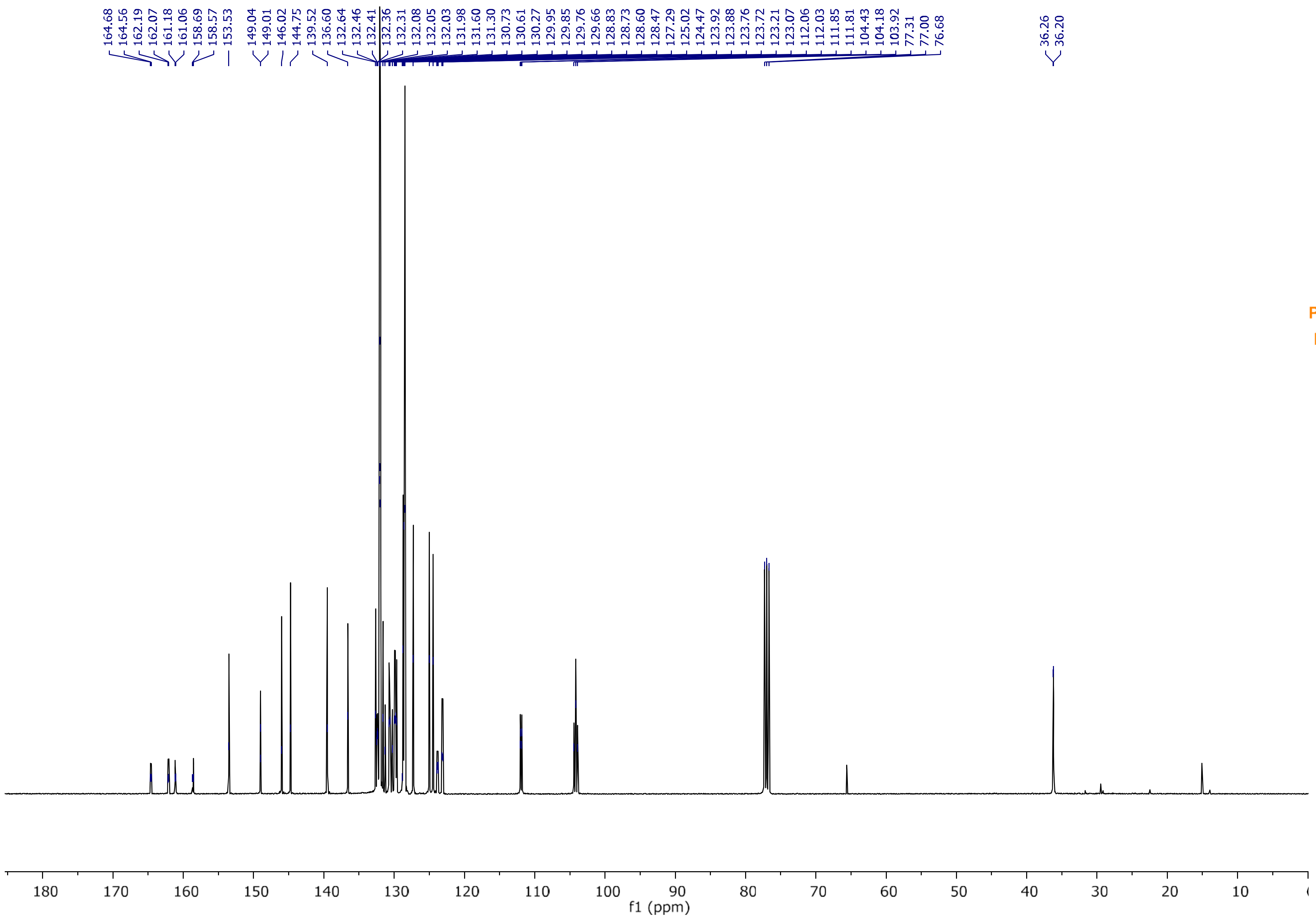
164.68
164.56
162.19
162.07
161.18
161.06
158.69
158.57
153.53
149.04
149.01
146.02
144.75
139.52
136.60
132.64
132.46
132.41
132.36
132.31
132.08
132.05
132.03
131.98
131.60
131.30
130.73
130.61
130.27
129.95
129.85
129.76
129.66
128.83
128.73
128.60
128.47
127.29
125.02
124.47
123.92
123.88
123.76
123.72
123.21
123.07
112.06
112.03
111.85
111.81
104.43
104.18
103.92
77.31
77.00
76.68
36.26
36.20
180
170
160
150
140
130
120
110
100
90
80
70
60
50
40
30
20
10
f1 (ppm)

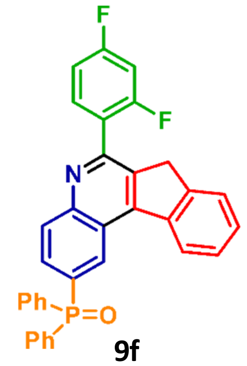
F
F
N
Ph
P=O
Ph
9f

^{1}H-RMN

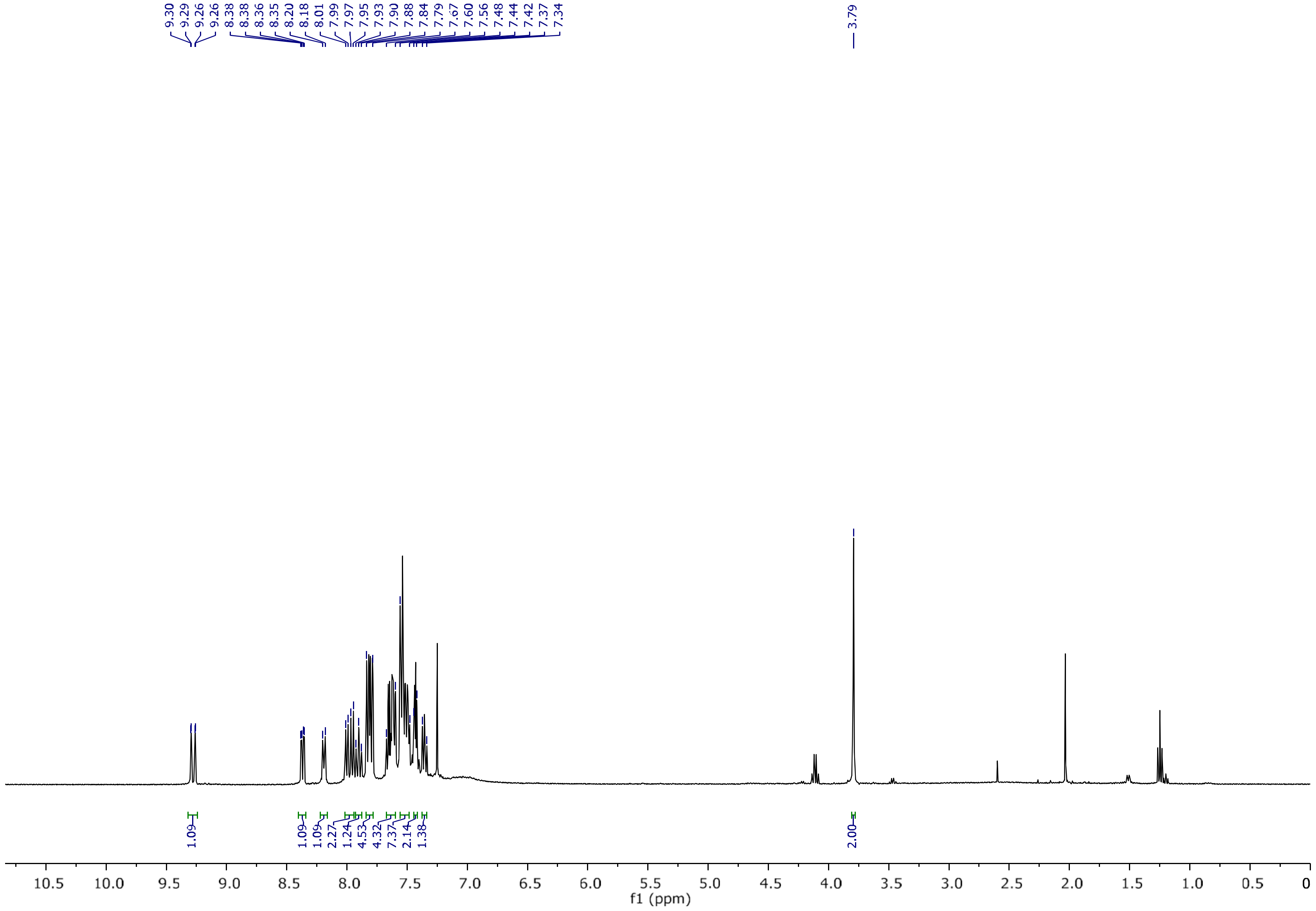

9.30
9.29
9.26
9.26
8.38
8.38
8.36
8.35
8.20
8.18
8.01
7.99
7.97
7.95
7.93
7.90
7.88
7.84
7.79
7.67
7.60
7.56
7.48
7.44
7.42
7.37
7.34
3.79
1.09
1.09
1.09
2.27
1.24
4.53
4.32
7.37
2.14
1.38
2.00
10.5 10.0 9.5 9.0 8.5 8.0 7.5 7.0 6.5 6.0 5.5 5.0 4.5 4.0 3.5 3.0 2.5 2.0 1.5 1.0 0.5 0
f1 (ppm)

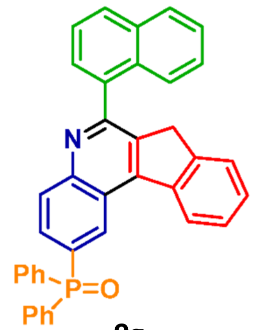

N
Ph
P=O
Ph

9g

^{31}P-RMN

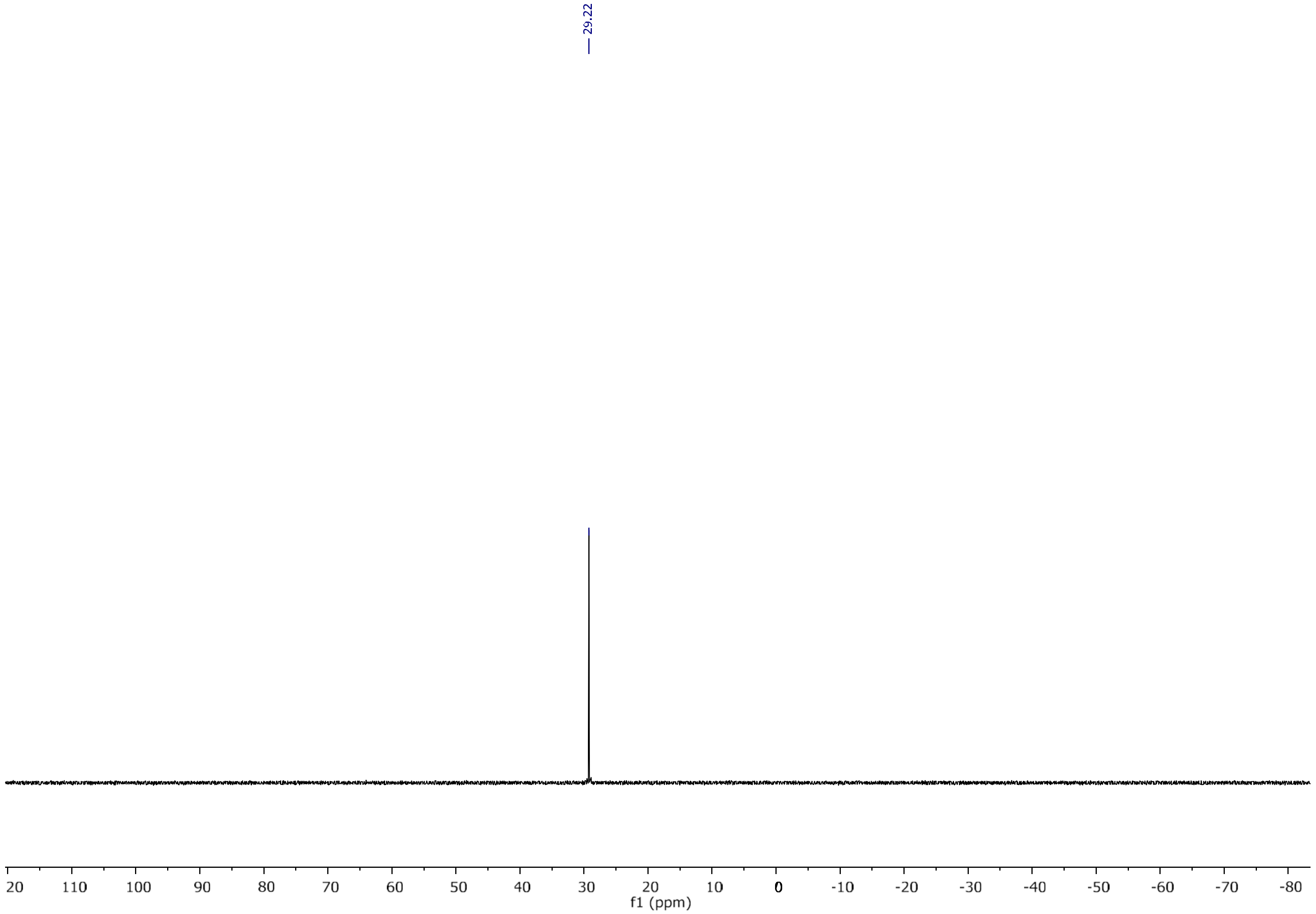

29.22
20
110
100
90
80
70
60
50
40
30
20
10
0
-10
-20
-30
-40
-50
-60
-70
-80
f1 (ppm)

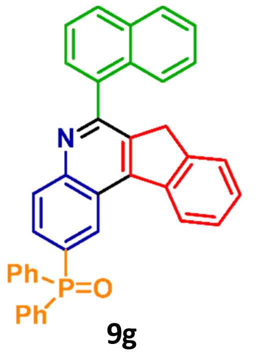

N
Ph
Ph
P=O

9g

^{13}C-RMN

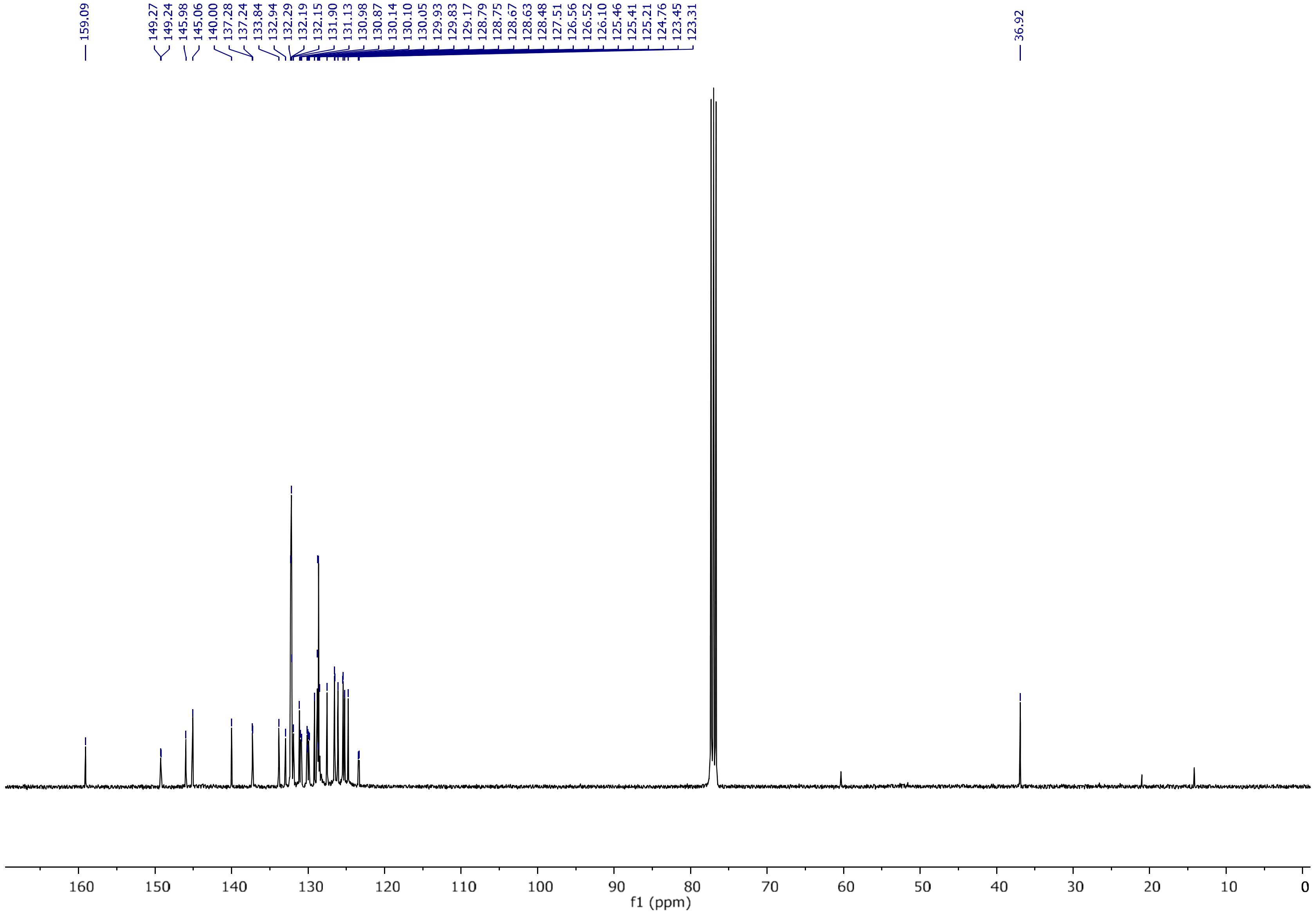
159.09
149.27
149.24
145.98
145.06
140.00
137.28
137.24
133.84
132.94
132.29
132.19
132.15
131.90
131.13
130.98
130.87
130.14
130.10
130.05
129.93
129.83
129.17
128.79
128.75
128.67
128.63
128.48
127.51
126.56
126.52
126.10
125.46
125.41
125.21
124.76
123.45
123.31
36.92
160 150 140 130 120 110 100 90 80 70 60 50 40 30 20 10 0
f1 (ppm)

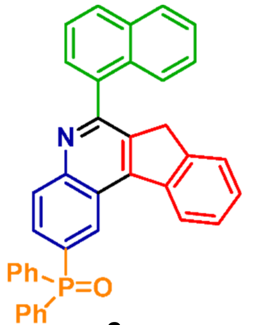
N
Ph
P=O
Ph

9g

^{1}H-RMN

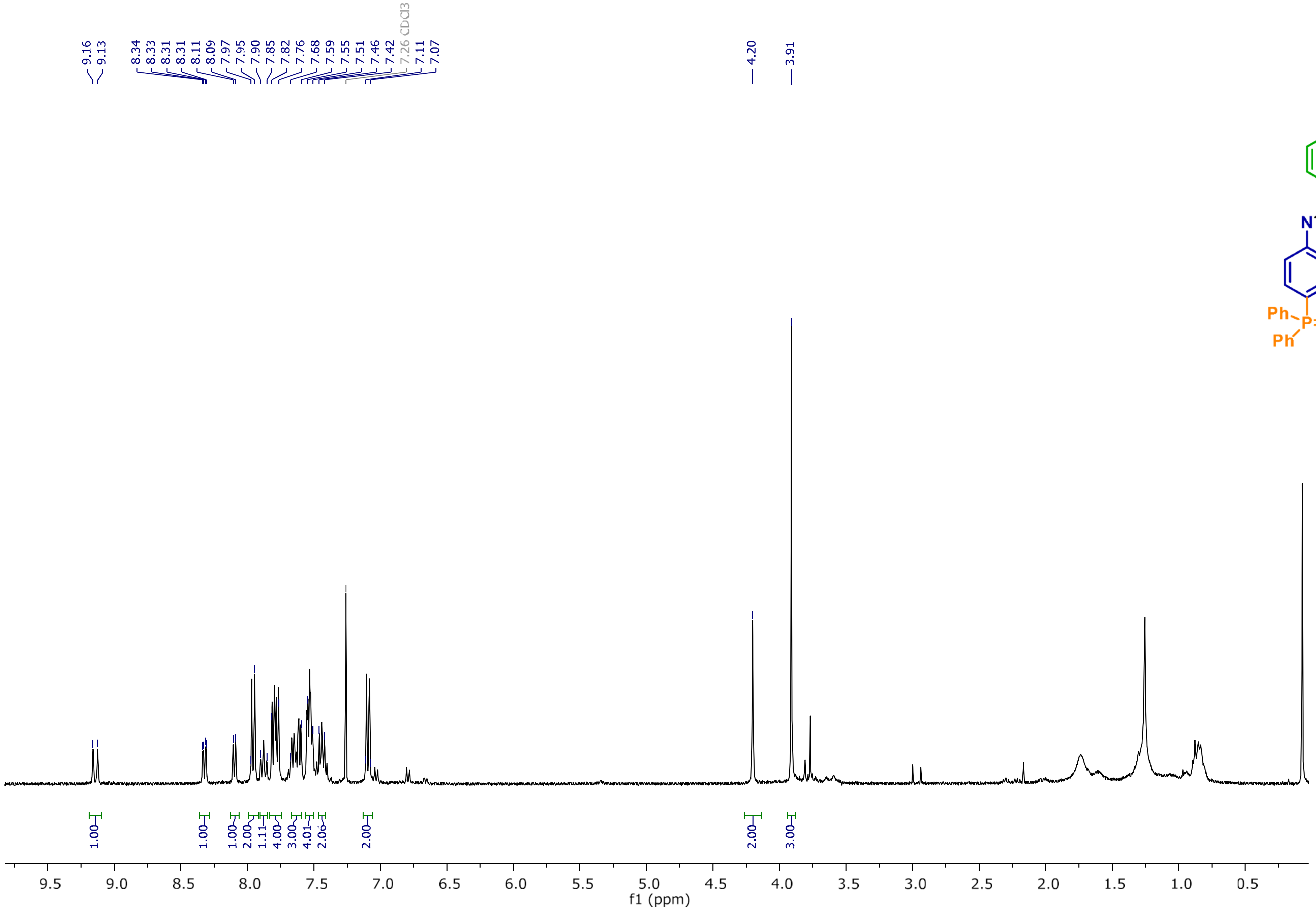
9.16
9.13
8.34
8.33
8.31
8.31
8.11
8.09
7.97
7.95
7.90
7.85
7.82
7.76
7.68
7.59
7.55
7.51
7.46
7.42
7.26 CDCl3
7.11
7.07
4.20
3.91
1.00
1.00
1.00
2.00
1.11
4.00
3.00
4.01
2.06
2.00
2.00
3.00
f1 (ppm)

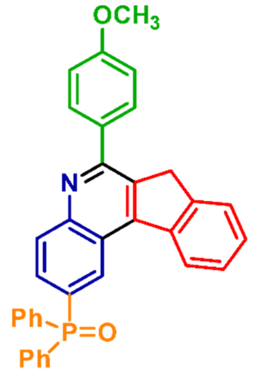
OCH3
N
Ph
P=O
Ph

9i

^{31}P-RMN

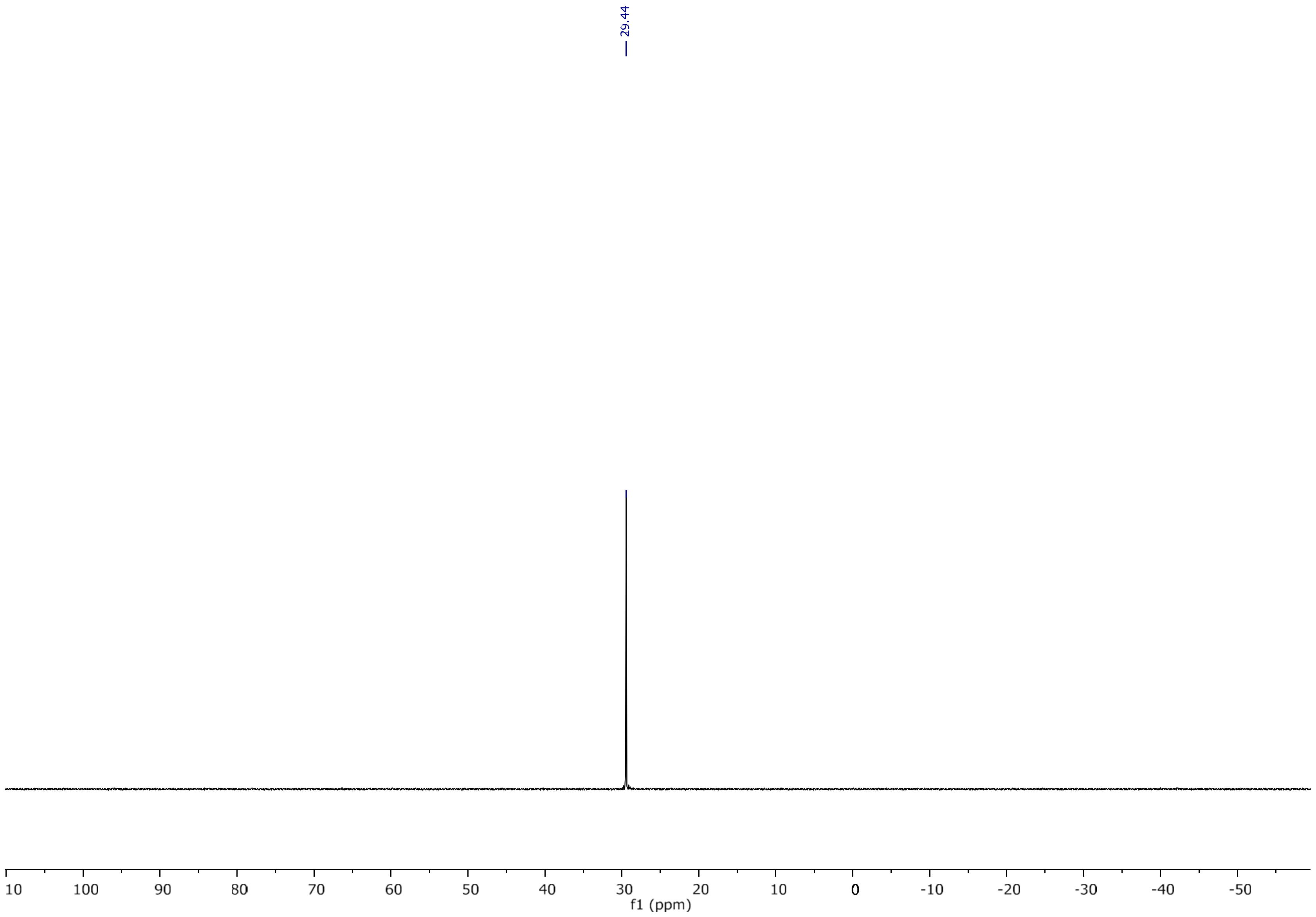

29.44
10 100 90 80 70 60 50 40 30 20 10 0 -10 -20 -30 -40 -50
f1 (ppm)

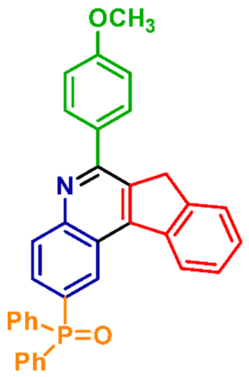

OCH3
N
Ph
P=O
Ph

9i

^{13}C-RMN

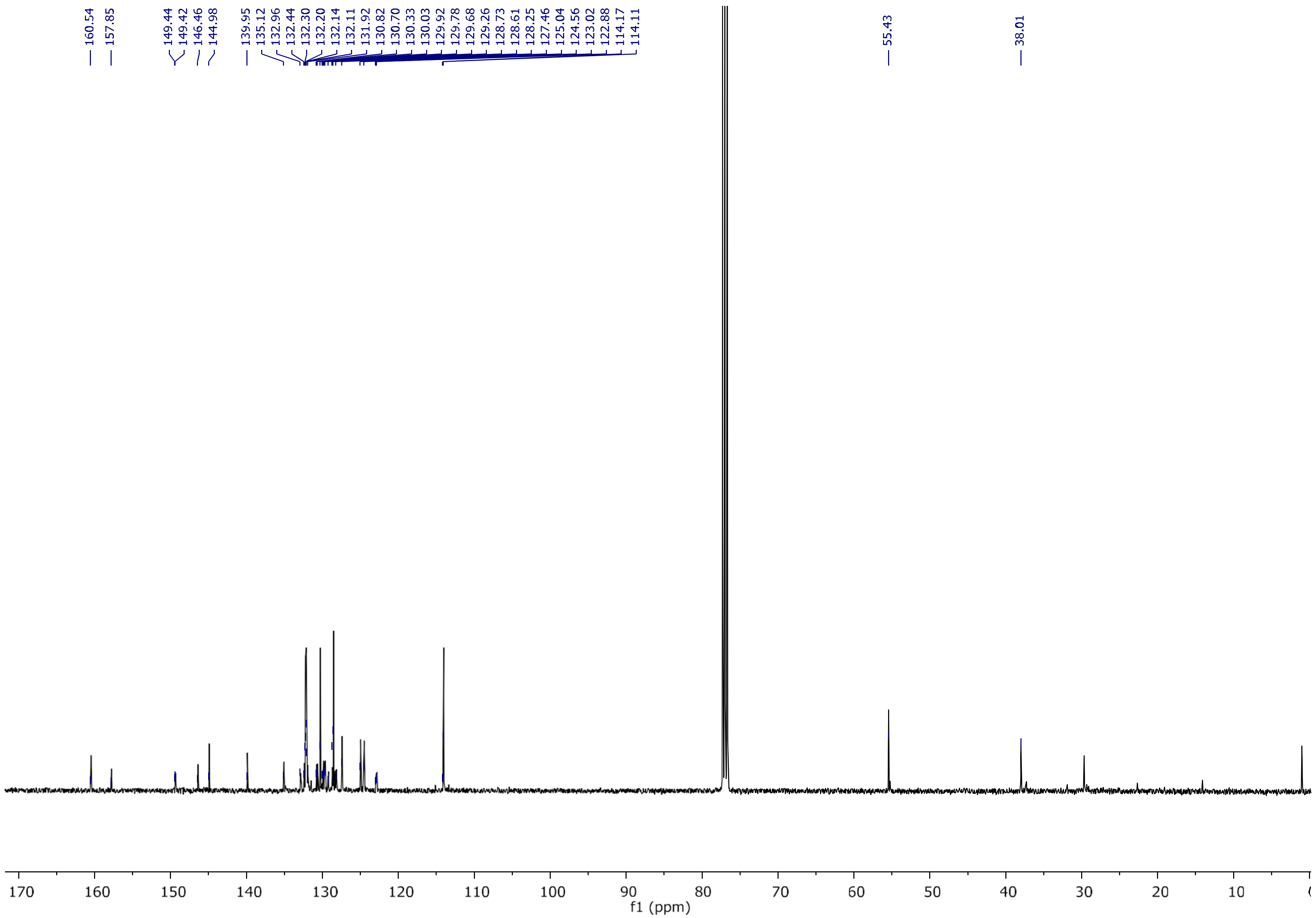

160.54
157.85
149.44
149.42
146.46
144.98
139.95
135.12
132.96
132.44
132.30
132.20
132.14
132.11
131.92
130.82
130.70
130.33
130.03
129.92
129.78
129.68
129.26
128.73
128.61
128.25
127.46
125.04
124.56
123.02
122.88
114.17
114.11
55.43
38.01
170 160 150 140 130 120 110 100 90 80 70 60 50 40 30 20 10
f1 (ppm)

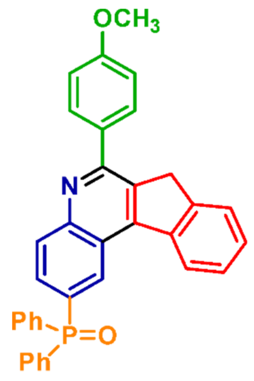

OCH3
N
Ph
P=O
Ph

9i

^{1}H-RMN

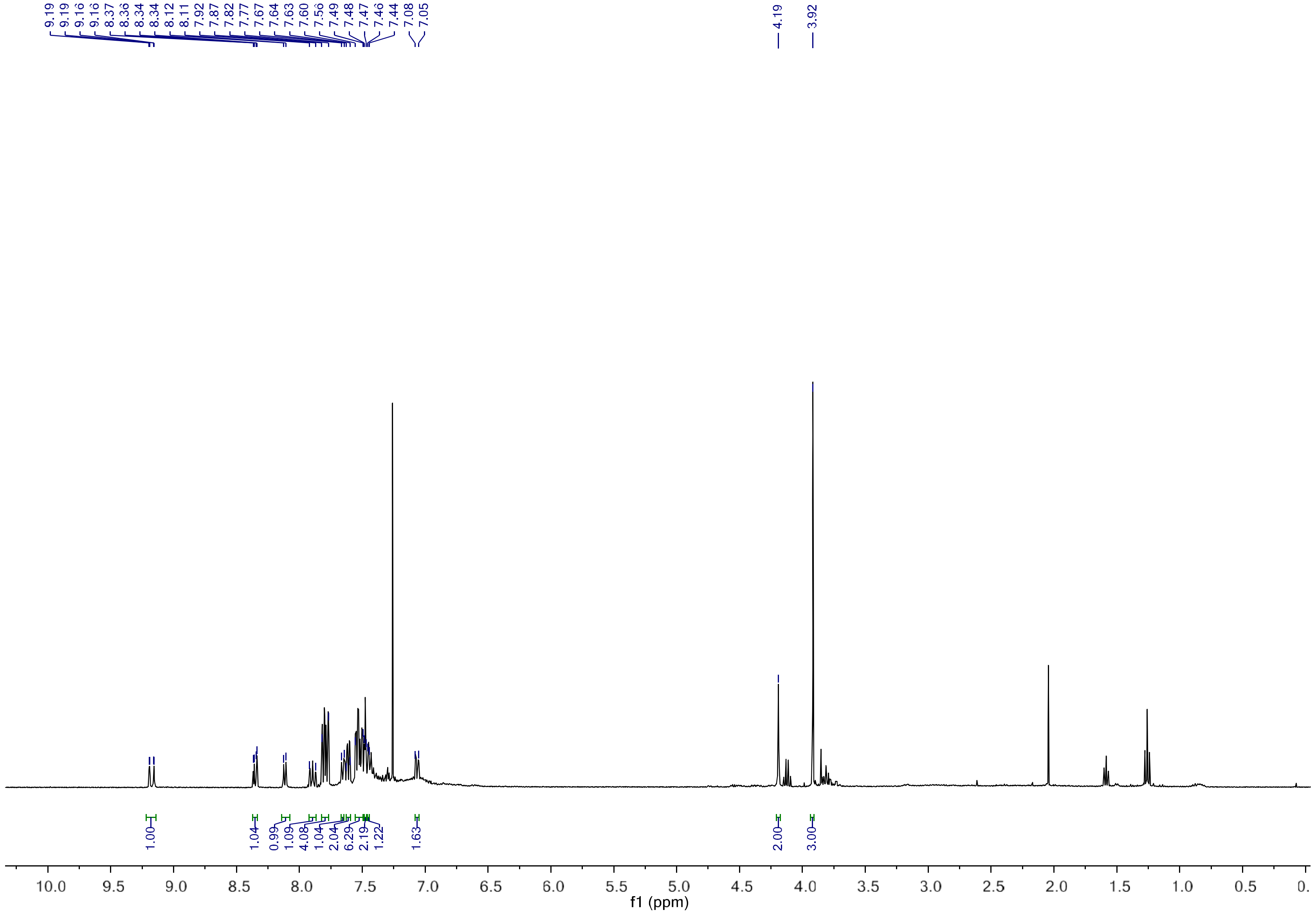
9.19
9.19
9.16
9.16
8.37
8.36
8.34
8.34
8.12
8.11
7.92
7.87
7.82
7.77
7.67
7.64
7.63
7.60
7.56
7.49
7.48
7.47
7.46
7.44
7.08
7.05
4.19
3.92
1.00
1.04
0.99
1.09
4.08
1.04
2.04
6.29
2.19
1.22
1.63
2.00
3.00
10.0 9.5 9.0 8.5 8.0 7.5 7.0 6.5 6.0 5.5 5.0 4.5 4.0 3.5 3.0 2.5 2.0 1.5 1.0 0.5 0.
f1 (ppm)

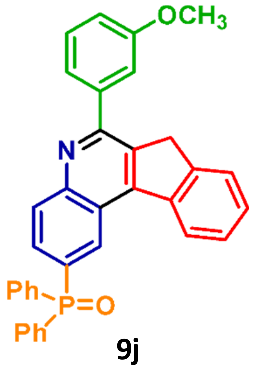
OCH3
N
Ph
P=O
Ph

9j

^{31}P-RMN

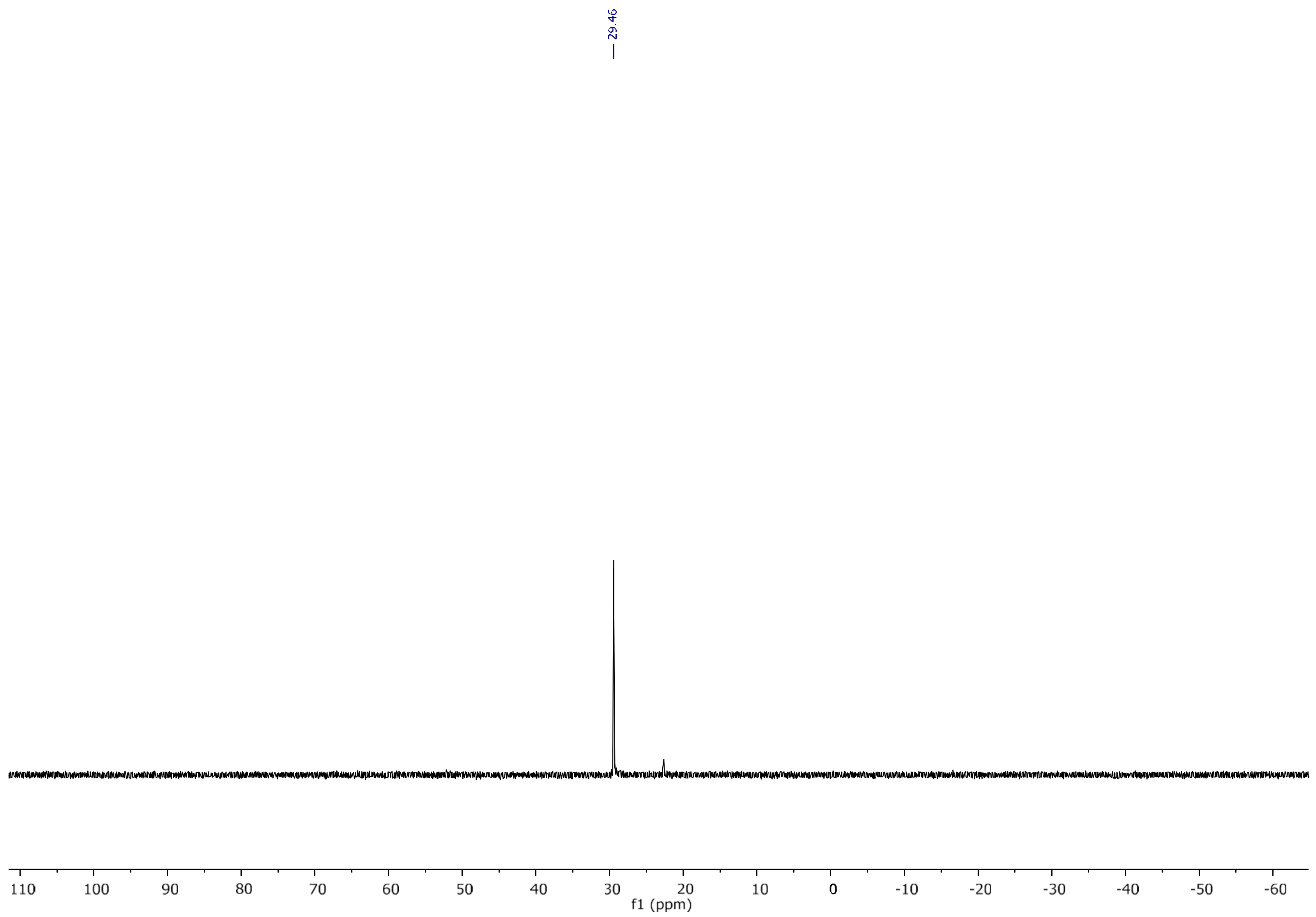

29.46
110
100
90
80
70
60
50
40
30
20
10
0
-10
-20
-30
-40
-50
-60
f1 (ppm)

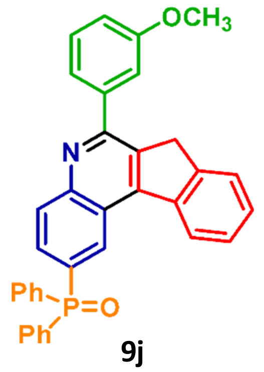

OCH3
N
Ph
P=O
Ph

9j

^{13}C-RMN

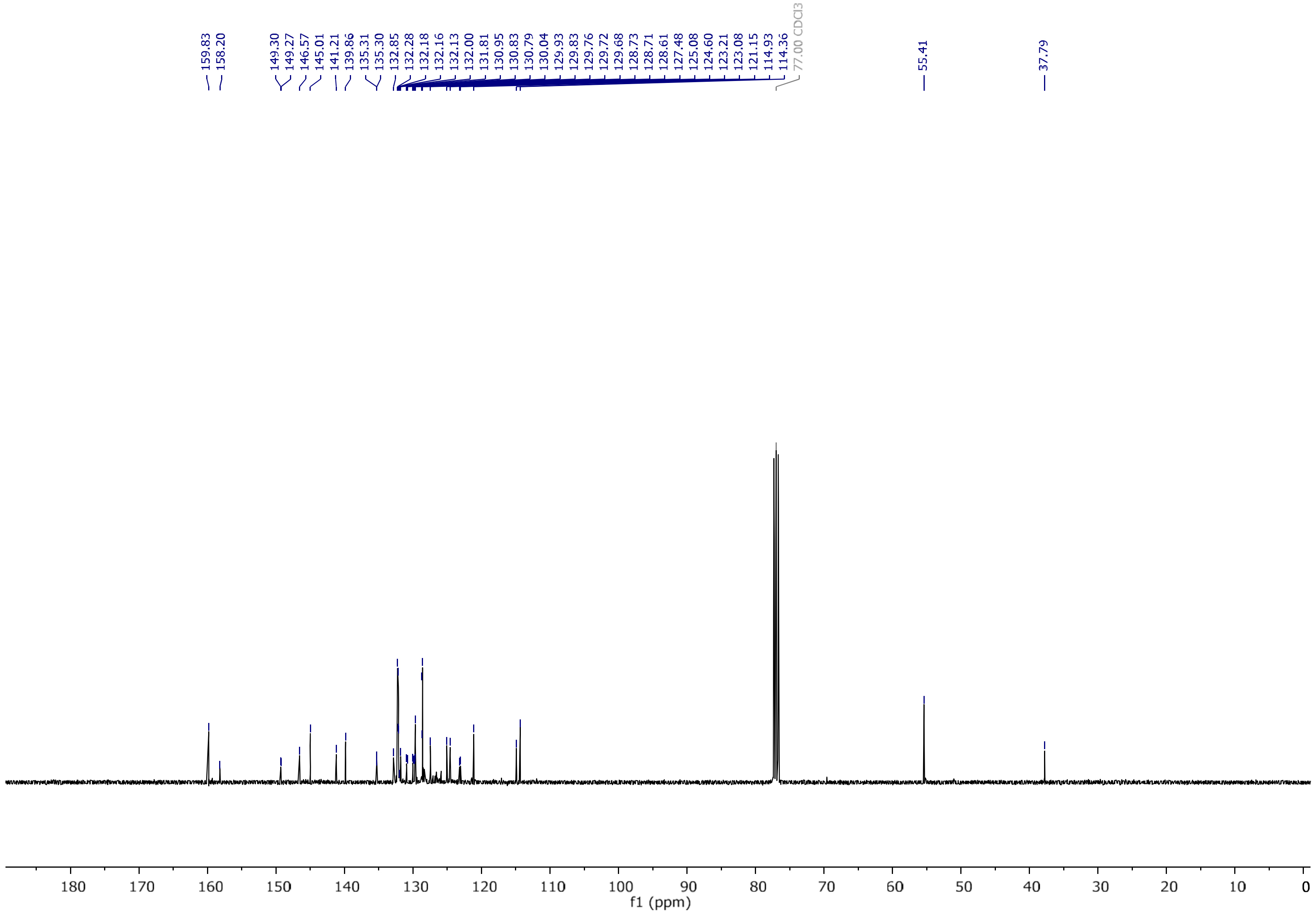

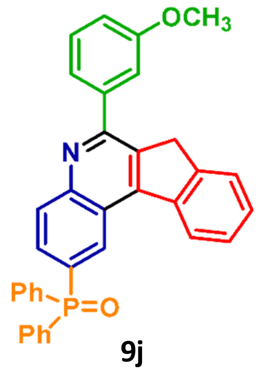

9j

^{1}H-RMN

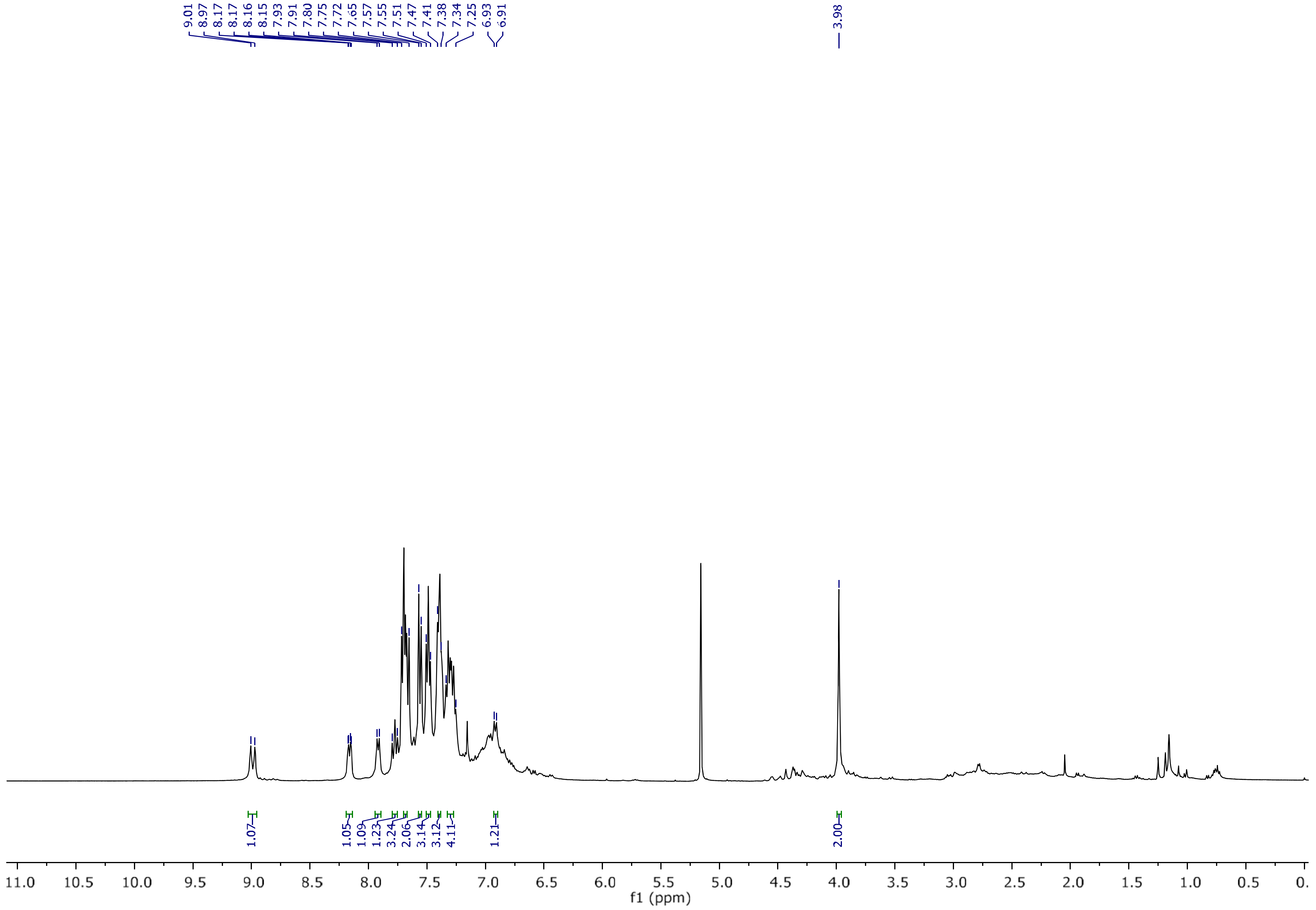

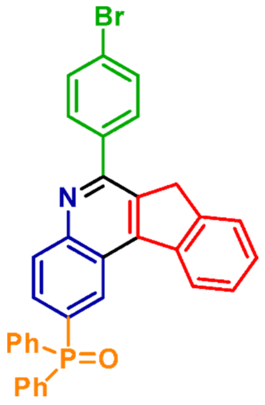

9k

^{31}P-RMN

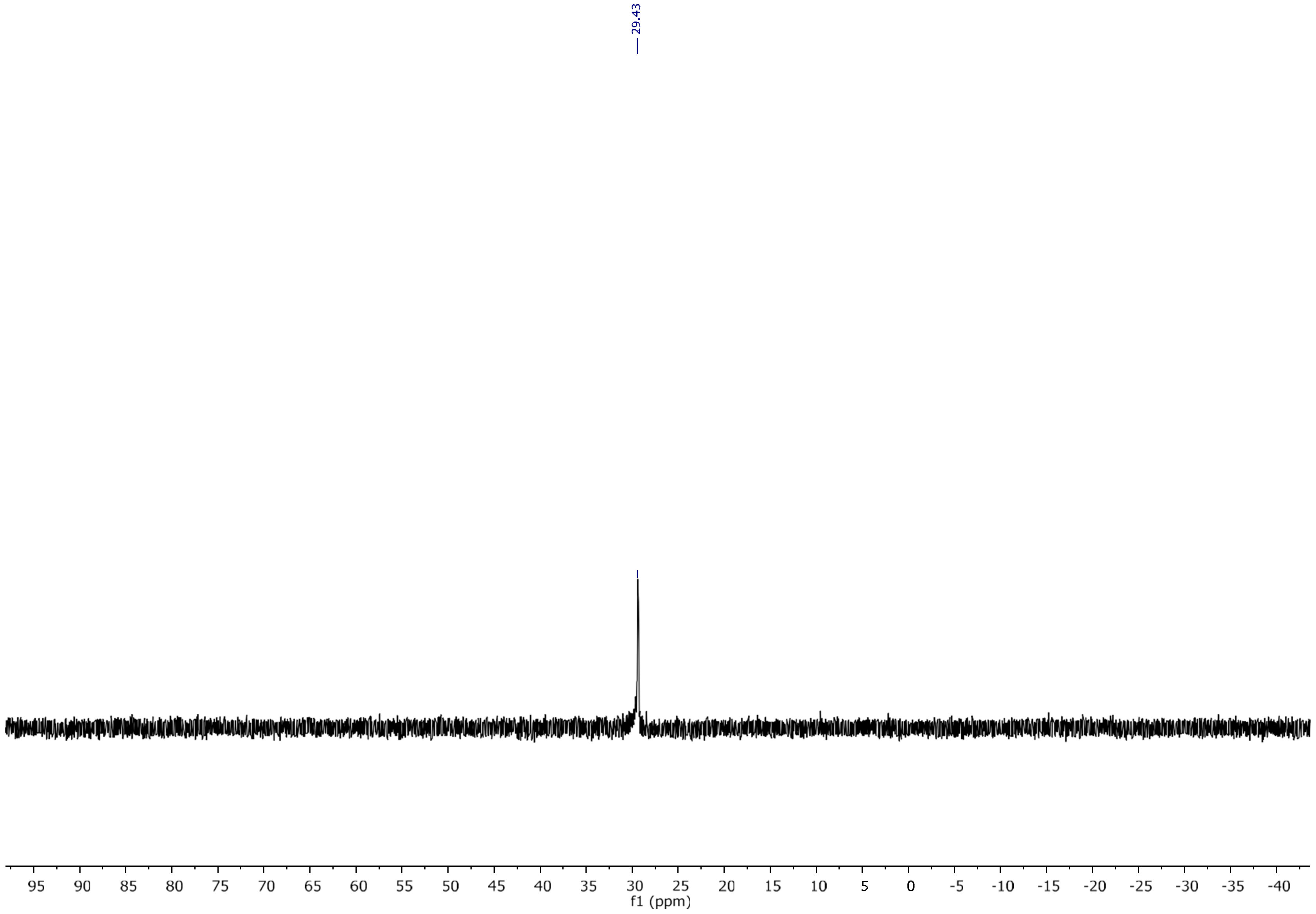

29.43
95 90 85 80 75 70 65 60 55 50 45 40 35 30 25 20 15 10 5 0 -5 -10 -15 -20 -25 -30 -35 -40
f1 (ppm)

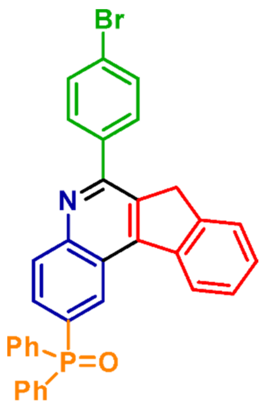

Br
N
Ph
P=O
Ph

9k

^{13}C-RMN

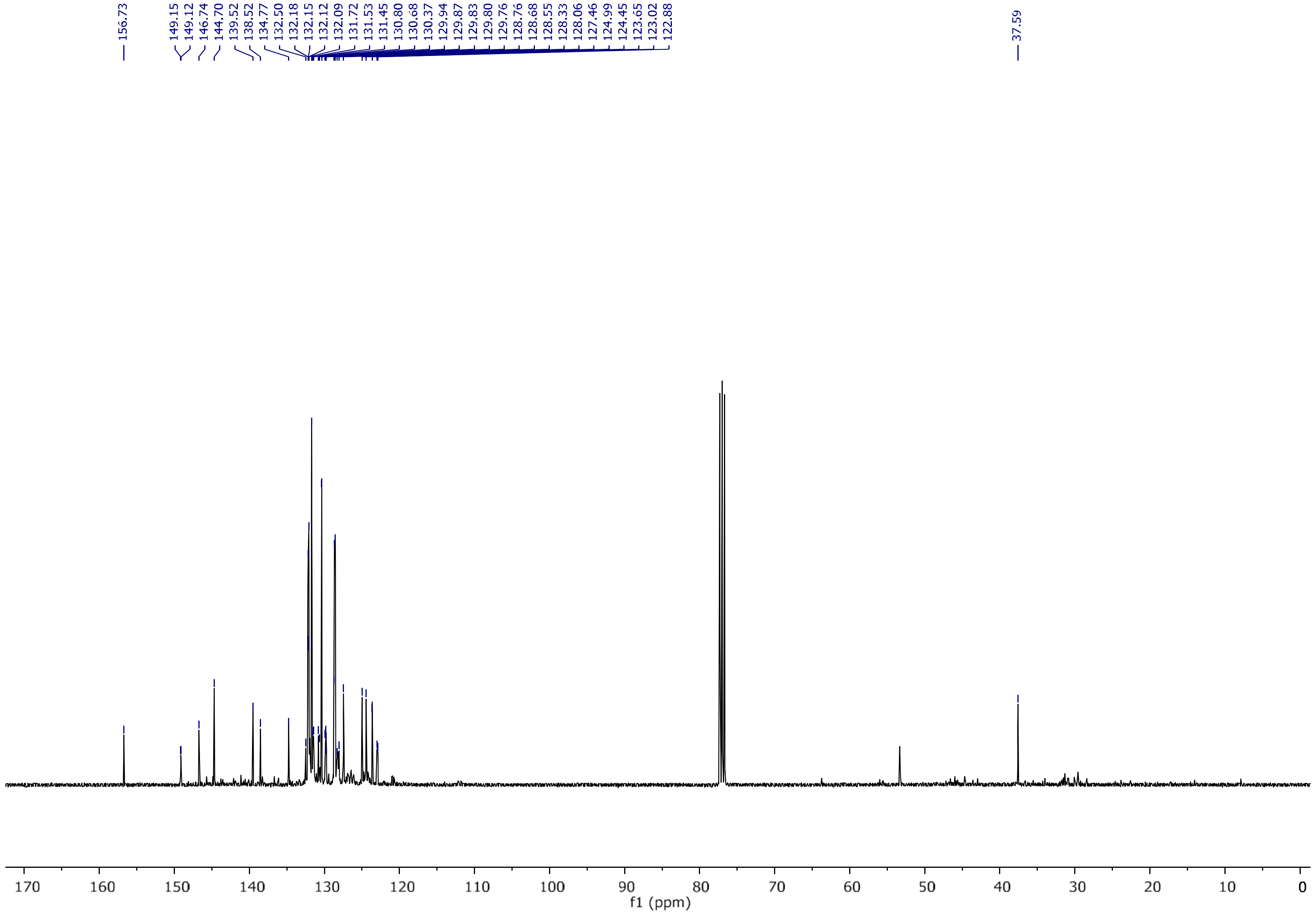
156.73
149.15
149.12
146.74
144.70
139.52
138.52
134.77
132.50
132.18
132.15
132.12
132.09
131.72
131.53
131.45
130.80
130.68
130.37
129.94
129.87
129.83
129.80
129.76
128.76
128.68
128.55
128.33
128.06
127.46
124.99
124.45
123.65
123.02
122.88
37.59
170
160
150
140
130
120
110
100
90
80
70
60
50
40
30
20
10
0
f1 (ppm)

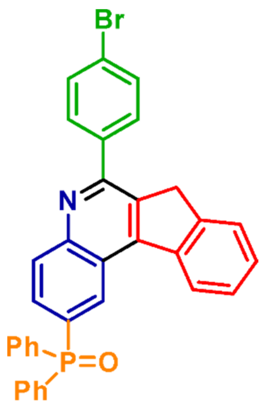
Br
N
Ph
P=O
Ph

9k

^{1}H-RMN

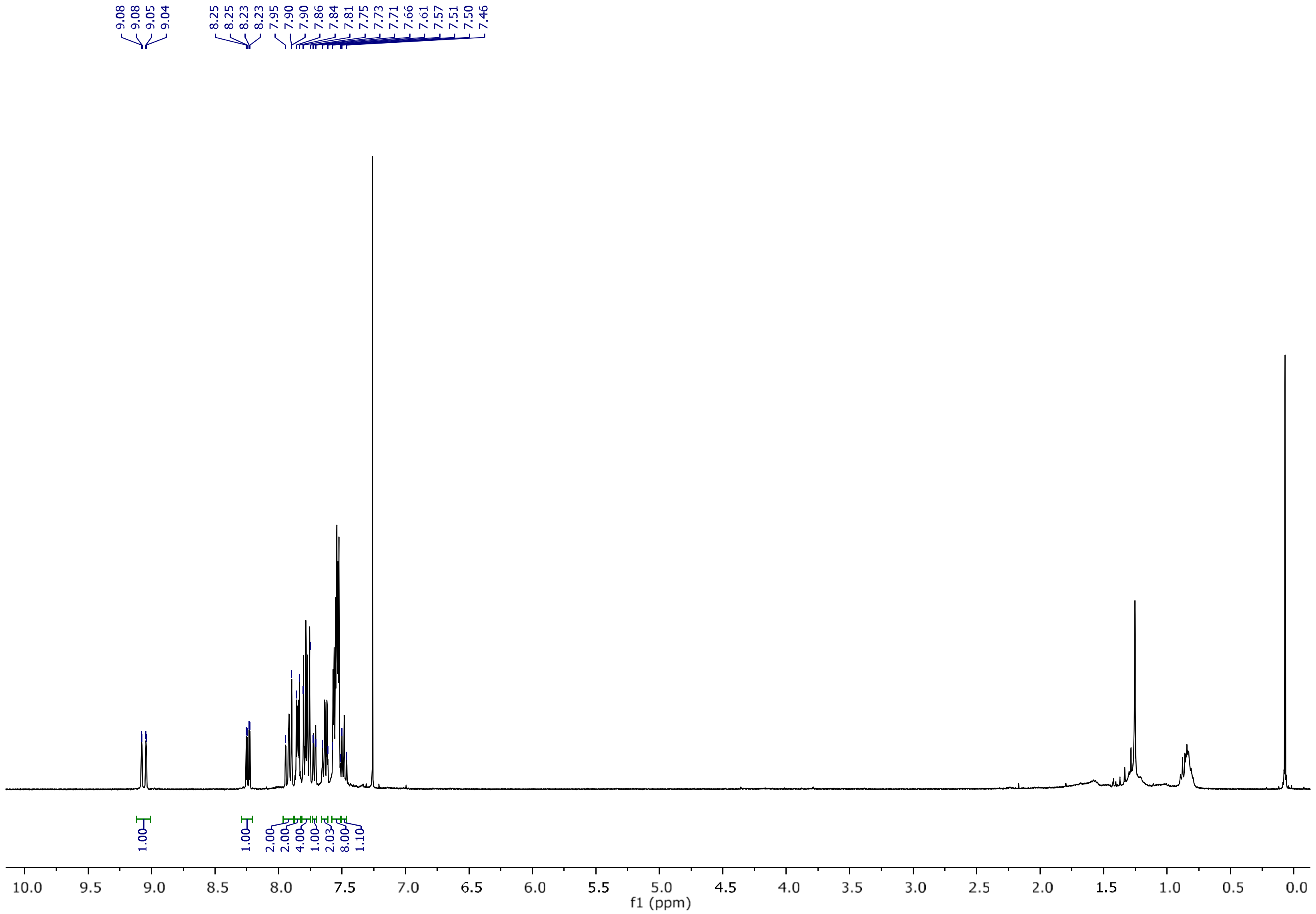

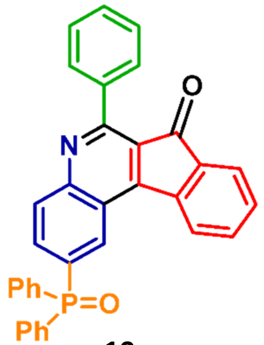

10a

^{31}P-RMN

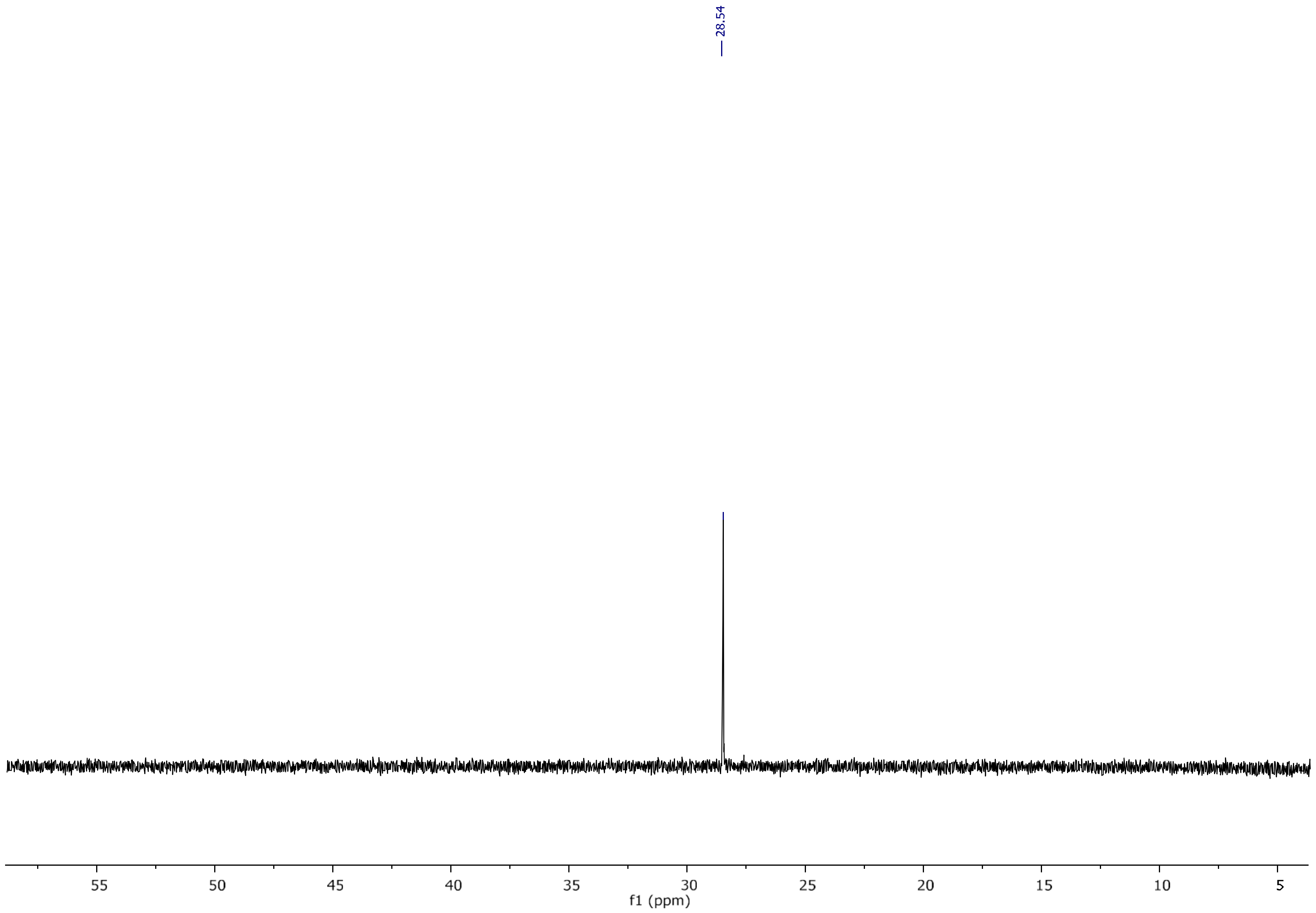

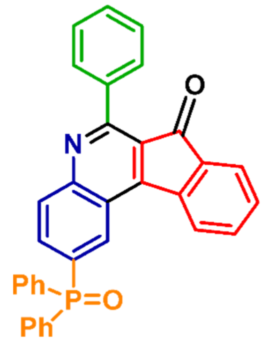

10a

^{13}C-RMN

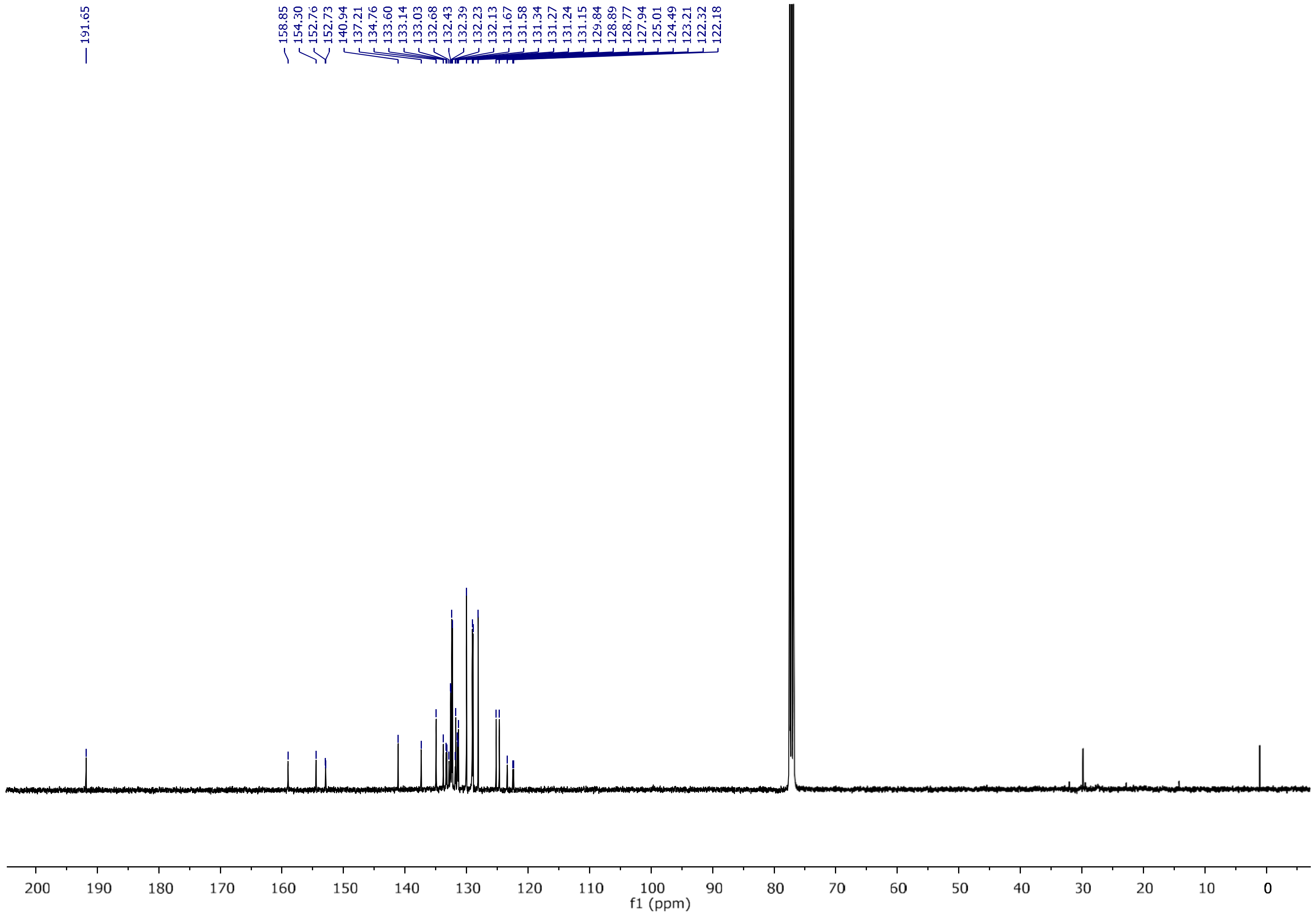
191.65
158.85
154.30
152.76
152.73
140.94
137.21
134.76
133.60
133.14
133.03
132.68
132.43
132.39
132.23
132.13
131.67
131.58
131.34
131.27
131.24
131.15
129.84
128.89
128.77
127.94
125.01
124.49
123.21
122.32
122.18
200 190 180 170 160 150 140 130 120 110 100 90 80 70 60 50 40 30 20 10 0
f1 (ppm)

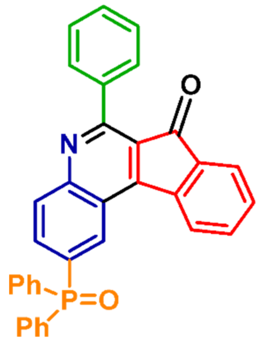
N
O
Ph
P=O
Ph

10a

^{1}H-RMN

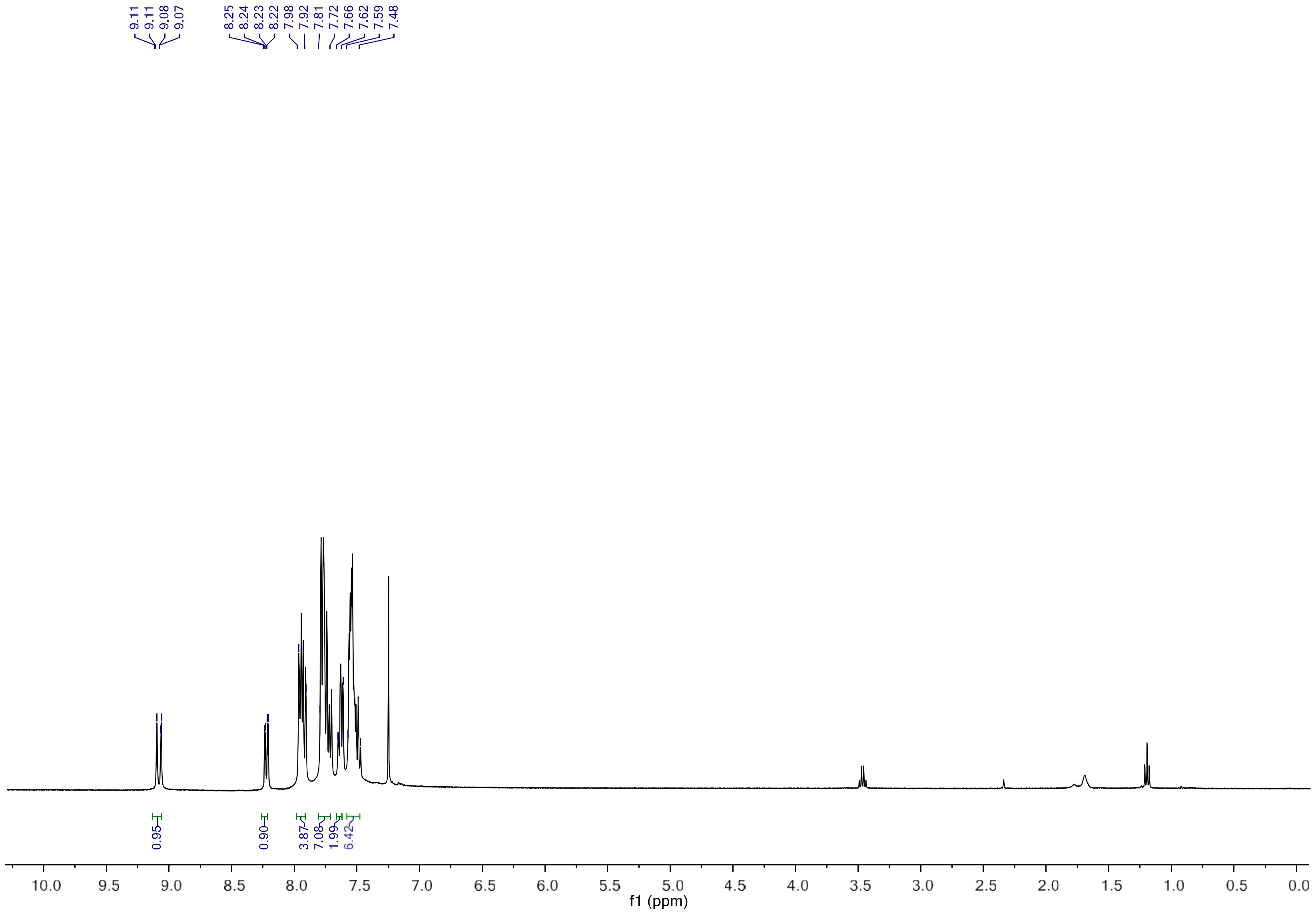

9.11
9.11
9.08
9.07
8.25
8.24
8.23
8.22
7.98
7.92
7.81
7.72
7.66
7.62
7.59
7.48
0.95
0.90
3.87
7.08
1.99
6.42
10.0
9.5
9.0
8.5
8.0
7.5
7.0
6.5
6.0
5.5
5.0
4.5
4.0
3.5
3.0
2.5
2.0
1.5
1.0
0.5
0.0
f1 (ppm)

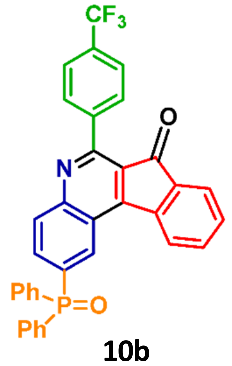

CF3
O
N
Ph
P=O
Ph

10b

^{31}P-RMN

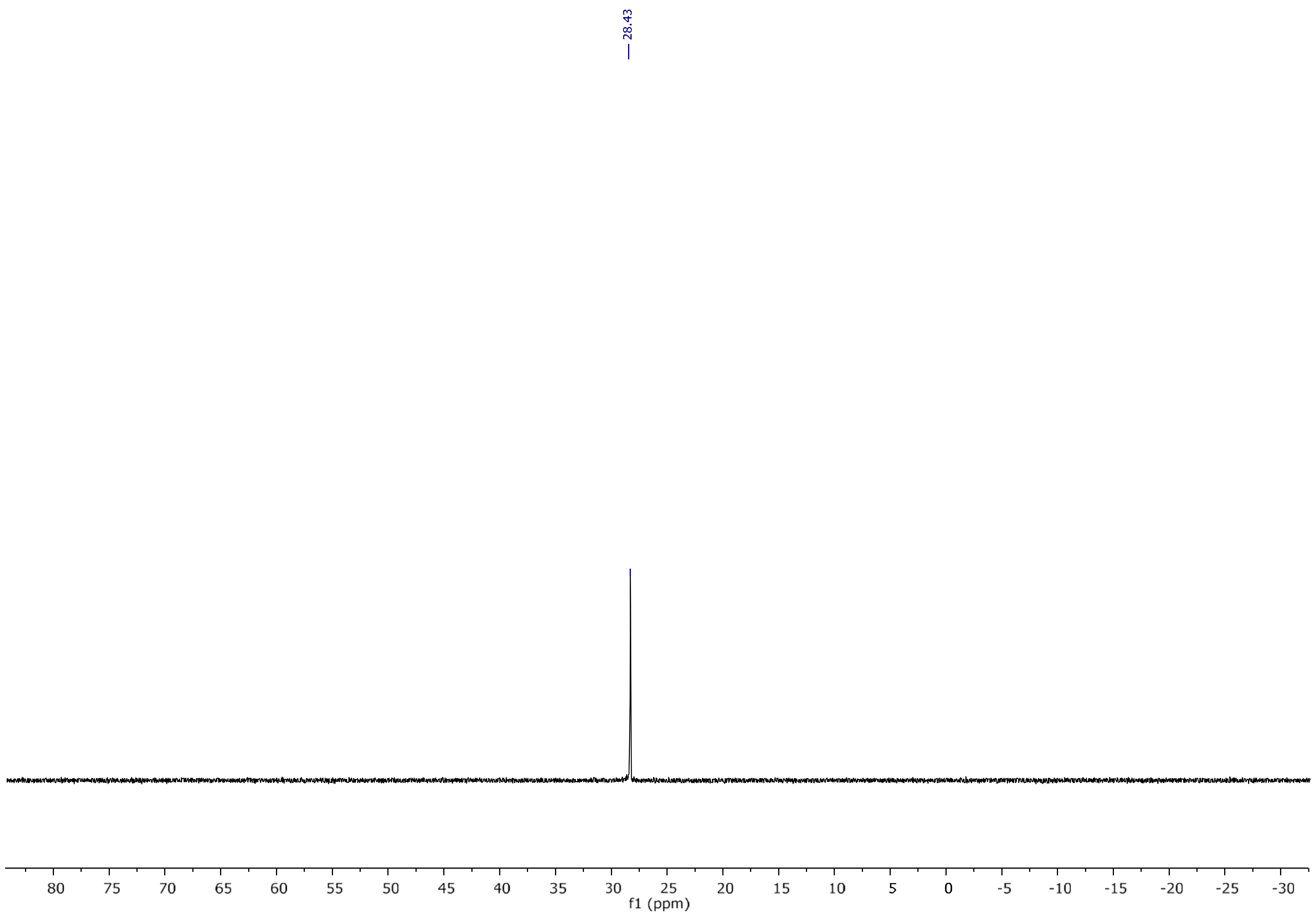

28.43
80
75
70
65
60
55
50
45
40
35
30
25
20
15
10
5
0
-5
-10
-15
-20
-25
-30
f1 (ppm)

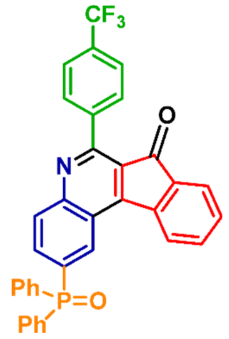

CF3
O
N
Ph
P=O
Ph

10b

^{19}F-RMN

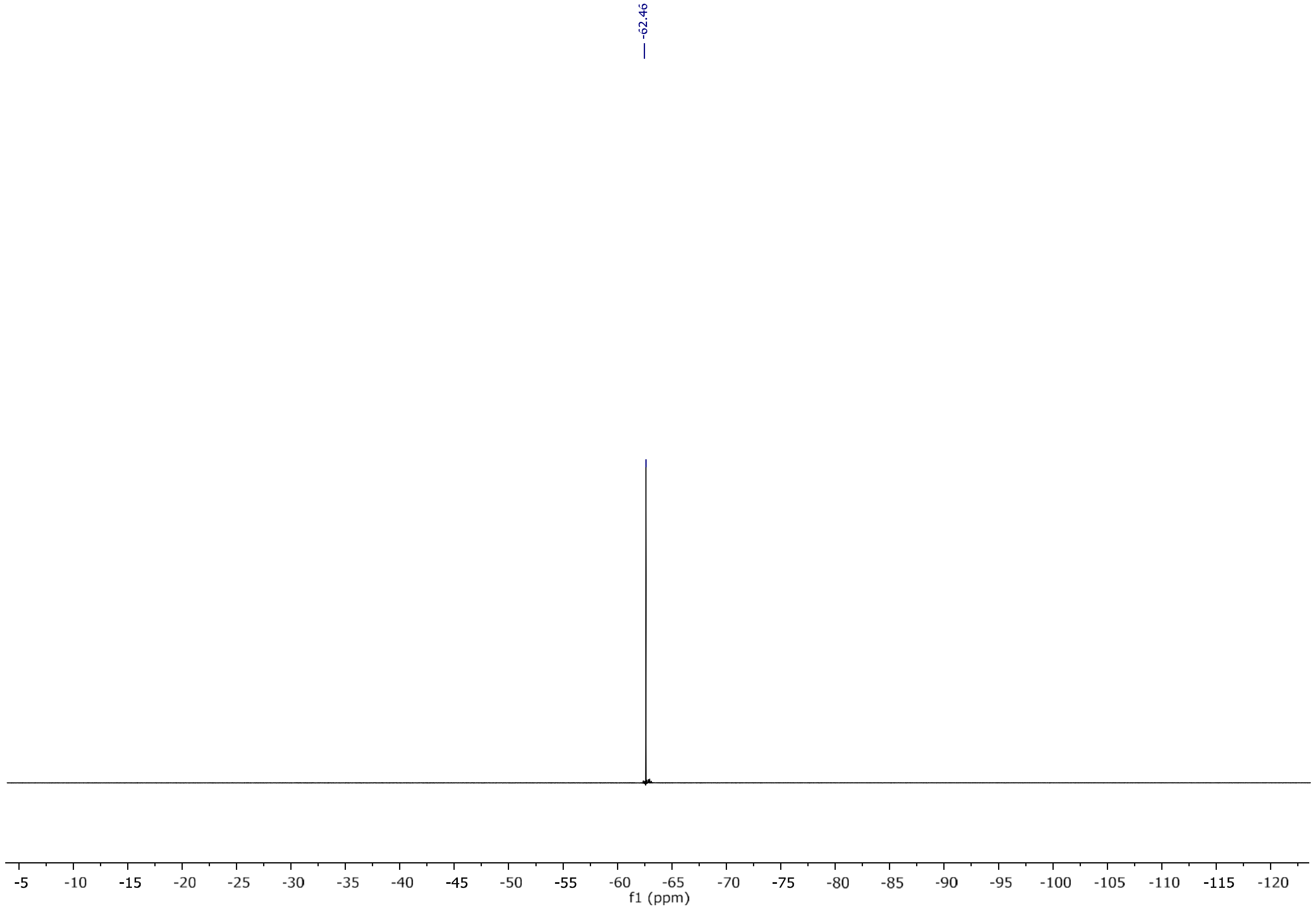
-62.46
-5 -10 -15 -20 -25 -30 -35 -40 -45 -50 -55 -60 -65 -70 -75 -80 -85 -90 -95 -100 -105 -110 -115 -120
f1 (ppm)

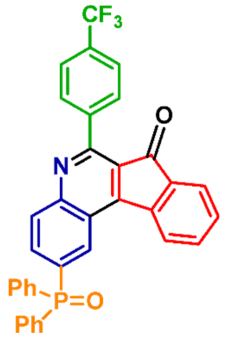
CF3
O
N
Ph
P=O
Ph

10b

^{13}C-RMN

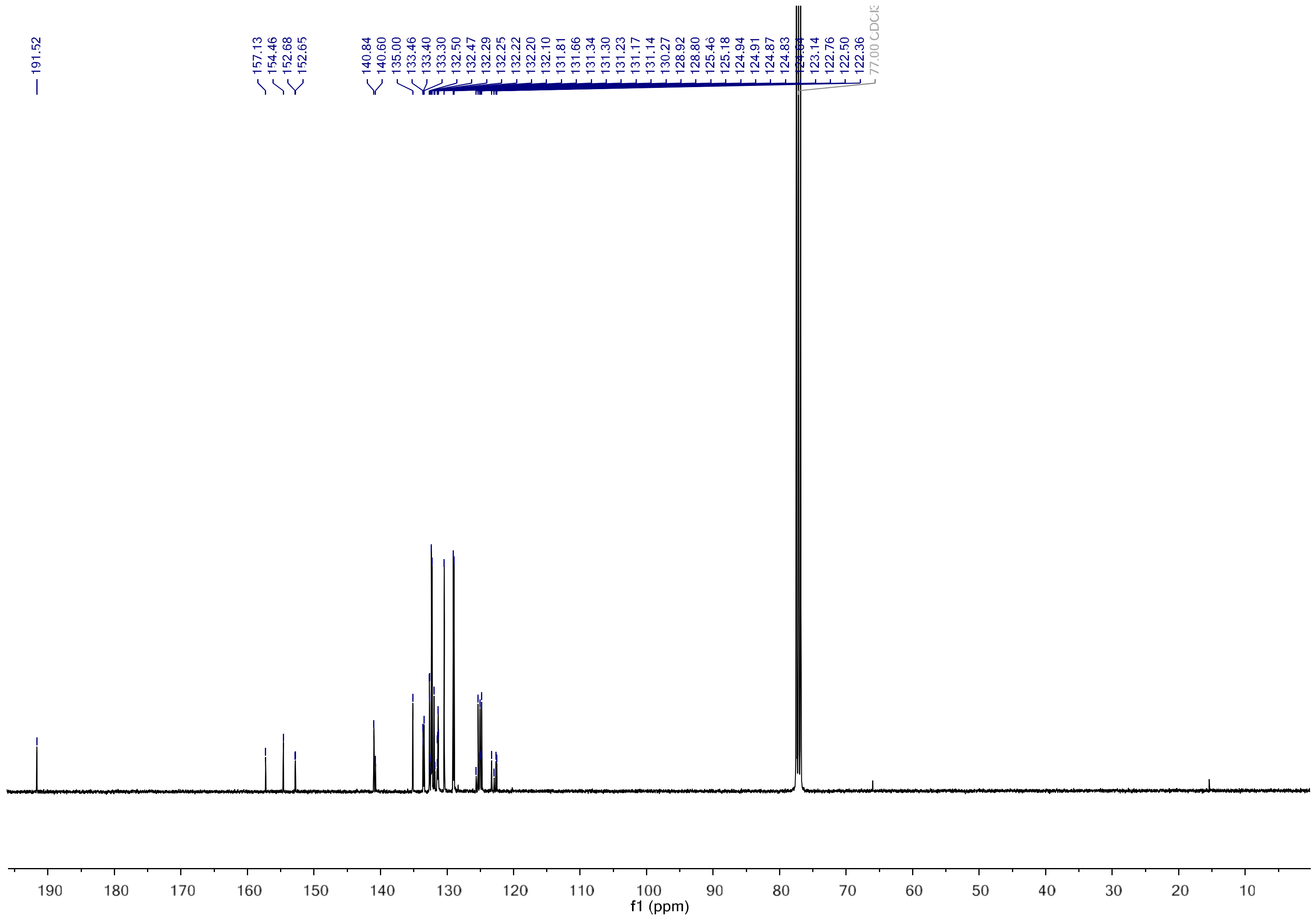
191.52
157.13
154.46
152.68
152.65
140.84
140.60
135.00
133.46
133.40
133.30
132.50
132.47
132.29
132.25
132.22
132.20
132.10
131.81
131.66
131.34
131.30
131.23
131.17
131.14
130.27
128.92
128.80
125.46
125.18
124.94
124.91
124.87
124.83
124.64
123.14
122.76
122.50
122.36
77.00 CDCl3
190
180
170
160
150
140
130
120
110
100
90
80
70
60
50
40
30
20
10
f1 (ppm)

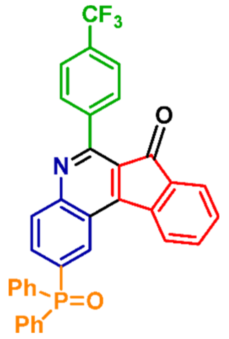
CF3
O
N
Ph
P=O
Ph

10b

^{1}H-RMN

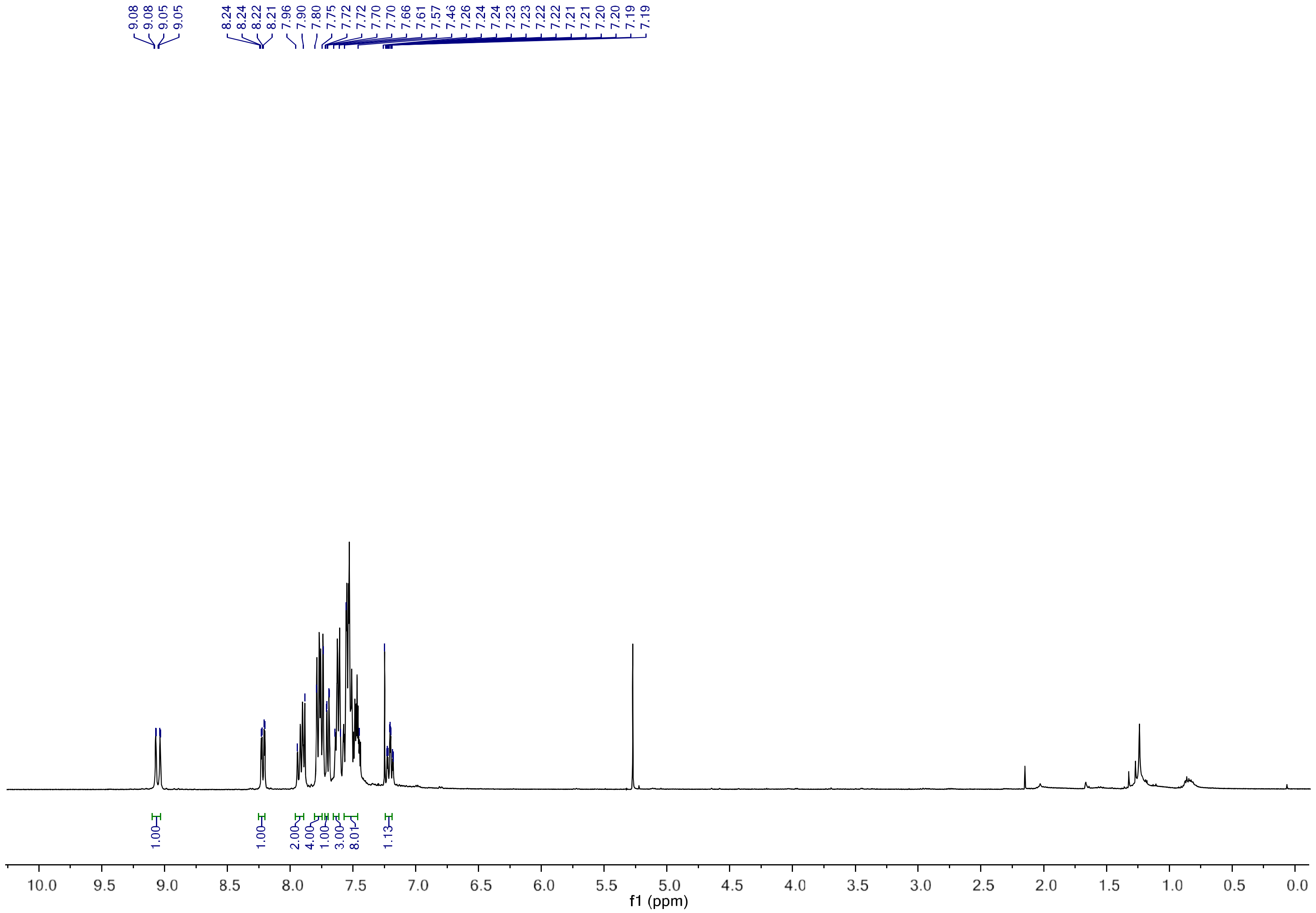

9.08
9.08
9.05
9.05
8.24
8.24
8.22
8.21
7.96
7.90
7.80
7.75
7.72
7.72
7.70
7.66
7.61
7.57
7.46
7.26
7.24
7.24
7.23
7.23
7.22
7.22
7.21
7.21
7.20
7.20
7.19
7.19
1.00
1.00
2.00
4.00
1.00
3.00
8.01
1.13
10.0
9.5
9.0
8.5
8.0
7.5
7.0
6.5
6.0
5.5
5.0
4.5
4.0
3.5
3.0
2.5
2.0
1.5
1.0
0.5
0.0
f1 (ppm)

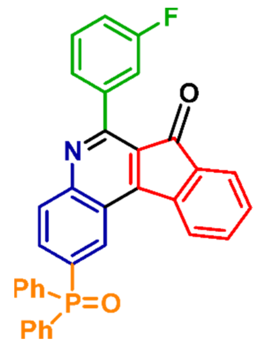

F
O
N
Ph
P=O
Ph

10d

^{31}P-RMN

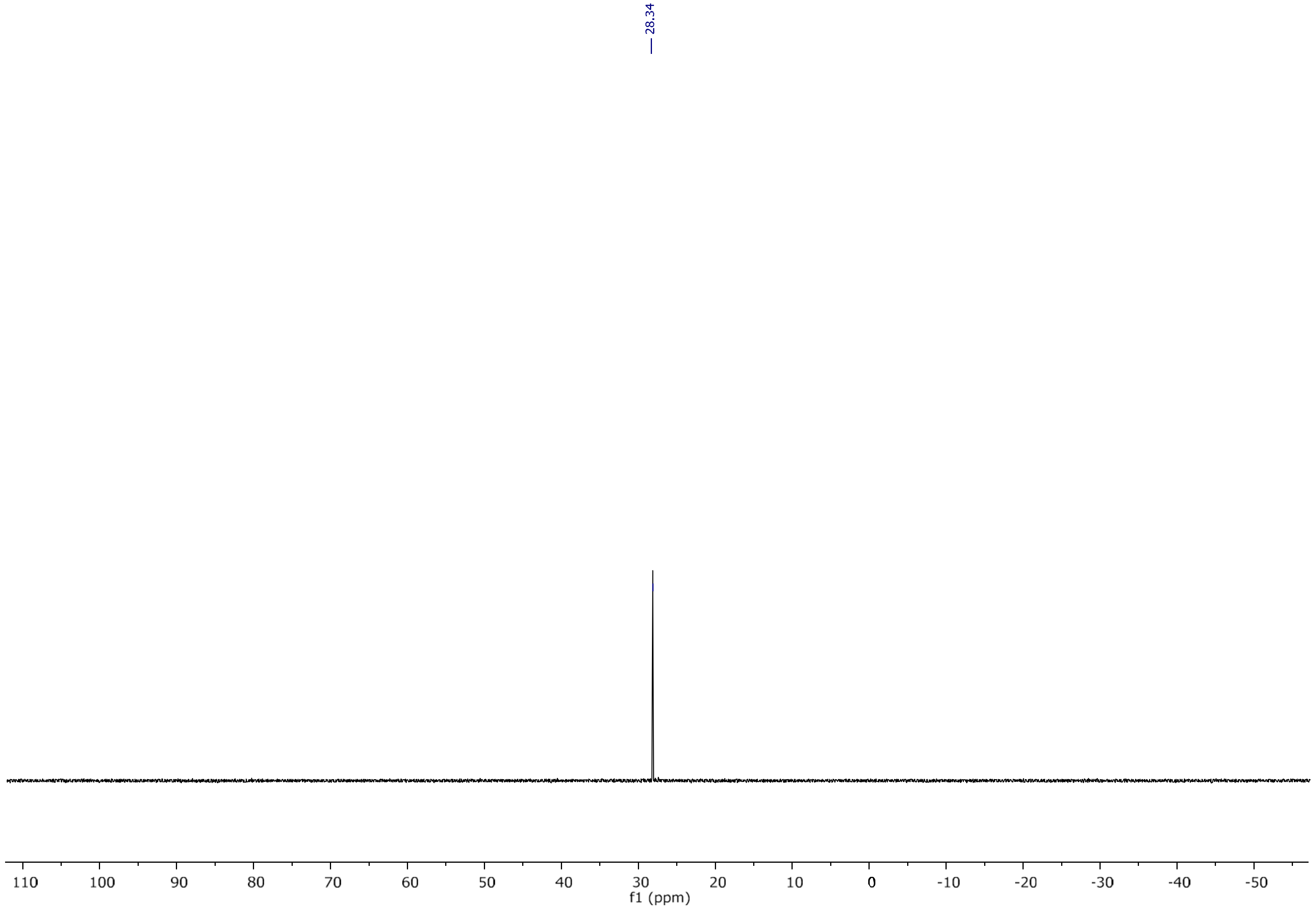
28.34
110
100
90
80
70
60
50
40
30
20
10
0
-10
-20
-30
-40
-50
f1 (ppm)

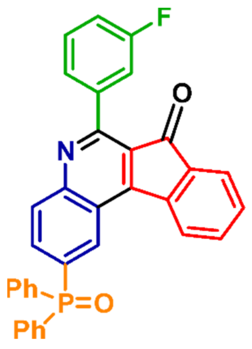
F
O
N
Ph
P=O
Ph

10d

^{19}F-RMN

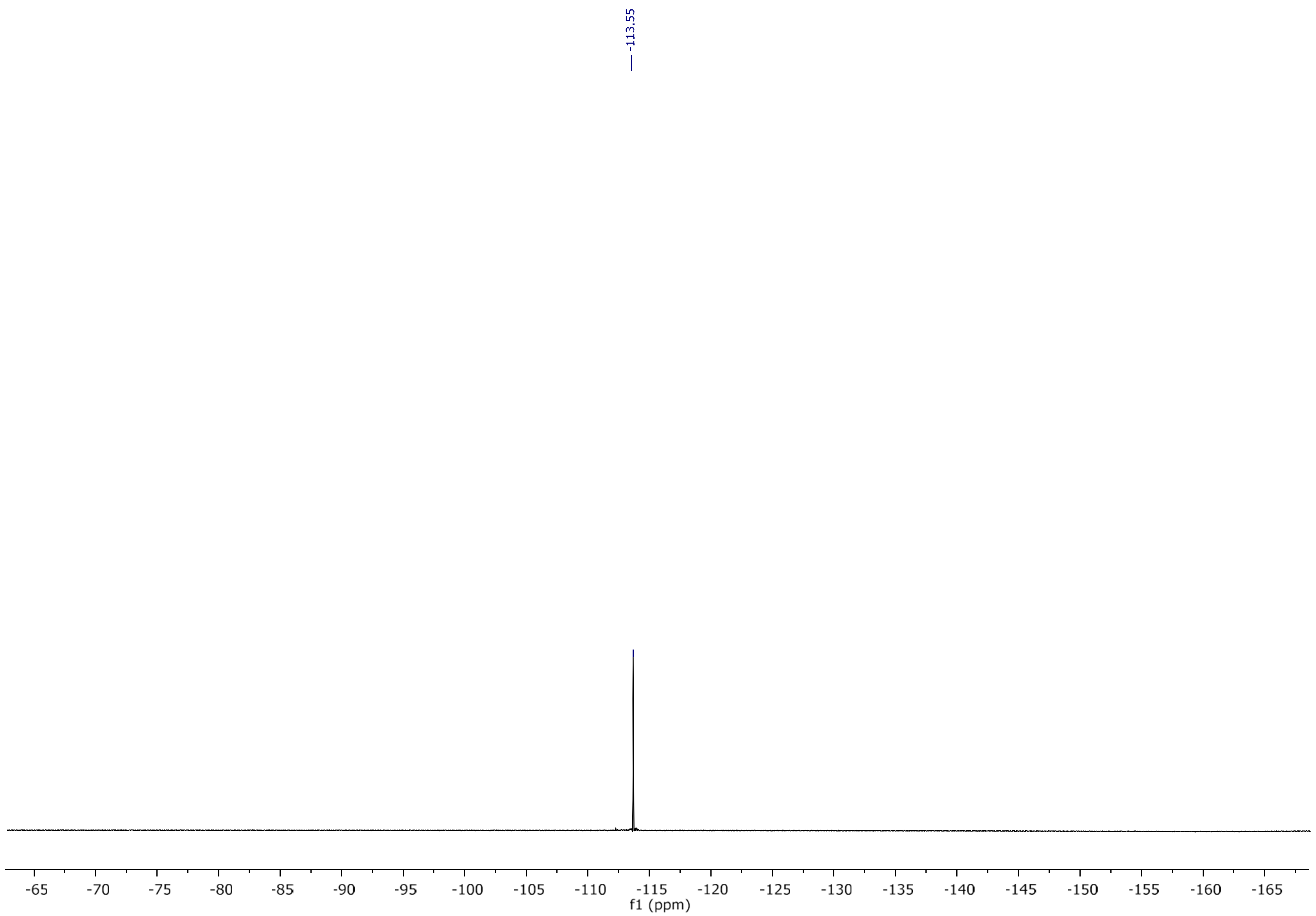

-113.55
-65 -70 -75 -80 -85 -90 -95 -100 -105 -110 -115 -120 -125 -130 -135 -140 -145 -150 -155 -160 -165
f1 (ppm)

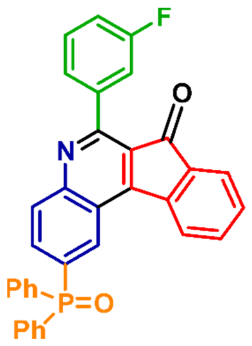

F
O
N
Ph
P=O
Ph

10d

^{13}C-RMN

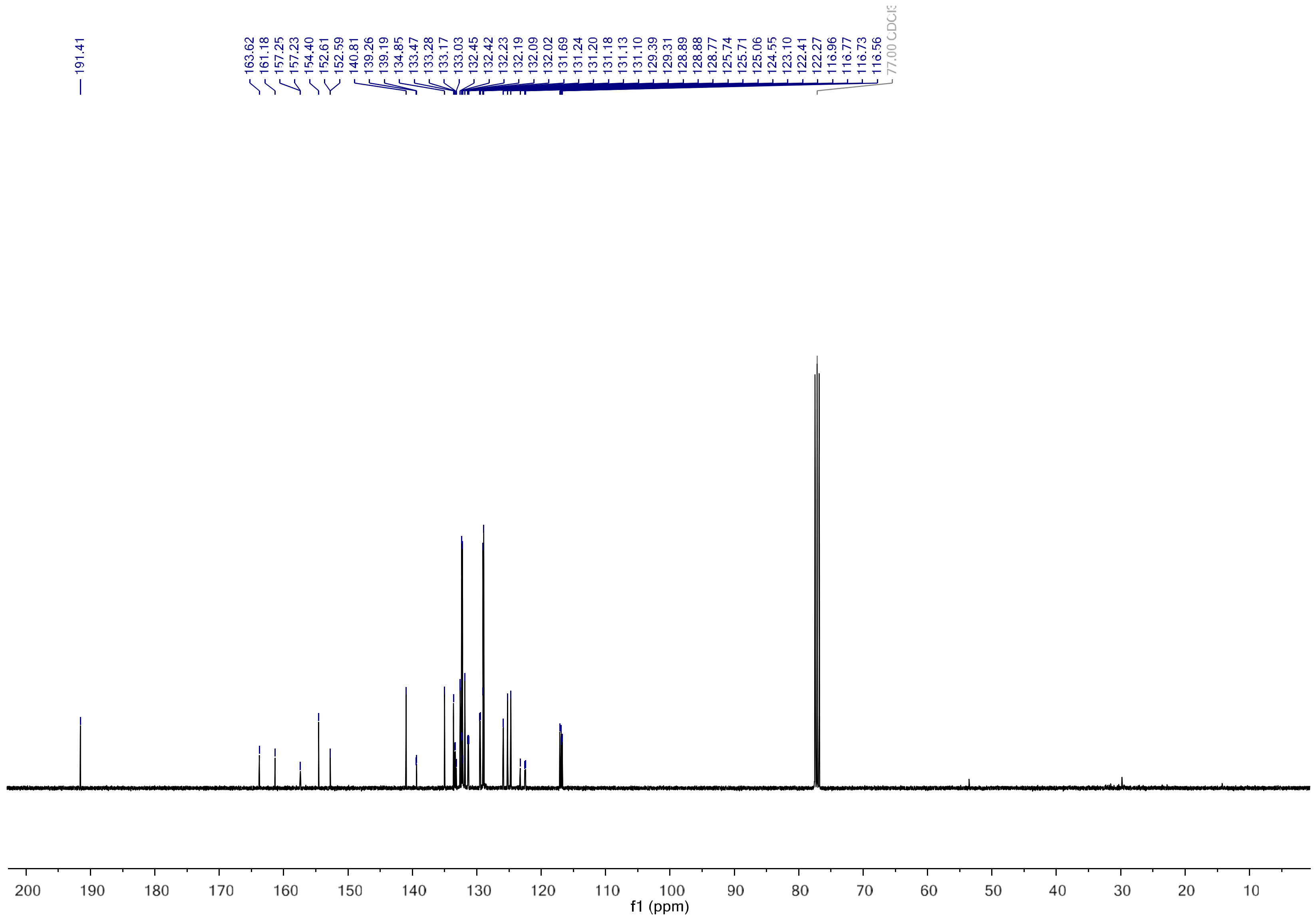
191.41
163.62
161.18
157.25
157.23
154.40
152.61
152.59
140.81
139.26
139.19
134.85
133.47
133.28
133.17
133.03
132.45
132.42
132.23
132.19
132.09
132.02
131.69
131.24
131.20
131.18
131.13
131.10
129.39
129.31
128.89
128.88
128.77
125.74
125.71
125.06
124.55
123.10
122.41
122.27
116.96
116.77
116.73
116.56
77.00 CDCl3
f1 (ppm)

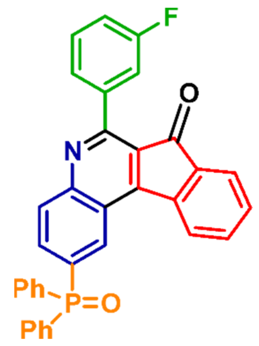
F
O
N
Ph
P=O
Ph

10d

^{1}H-RMN

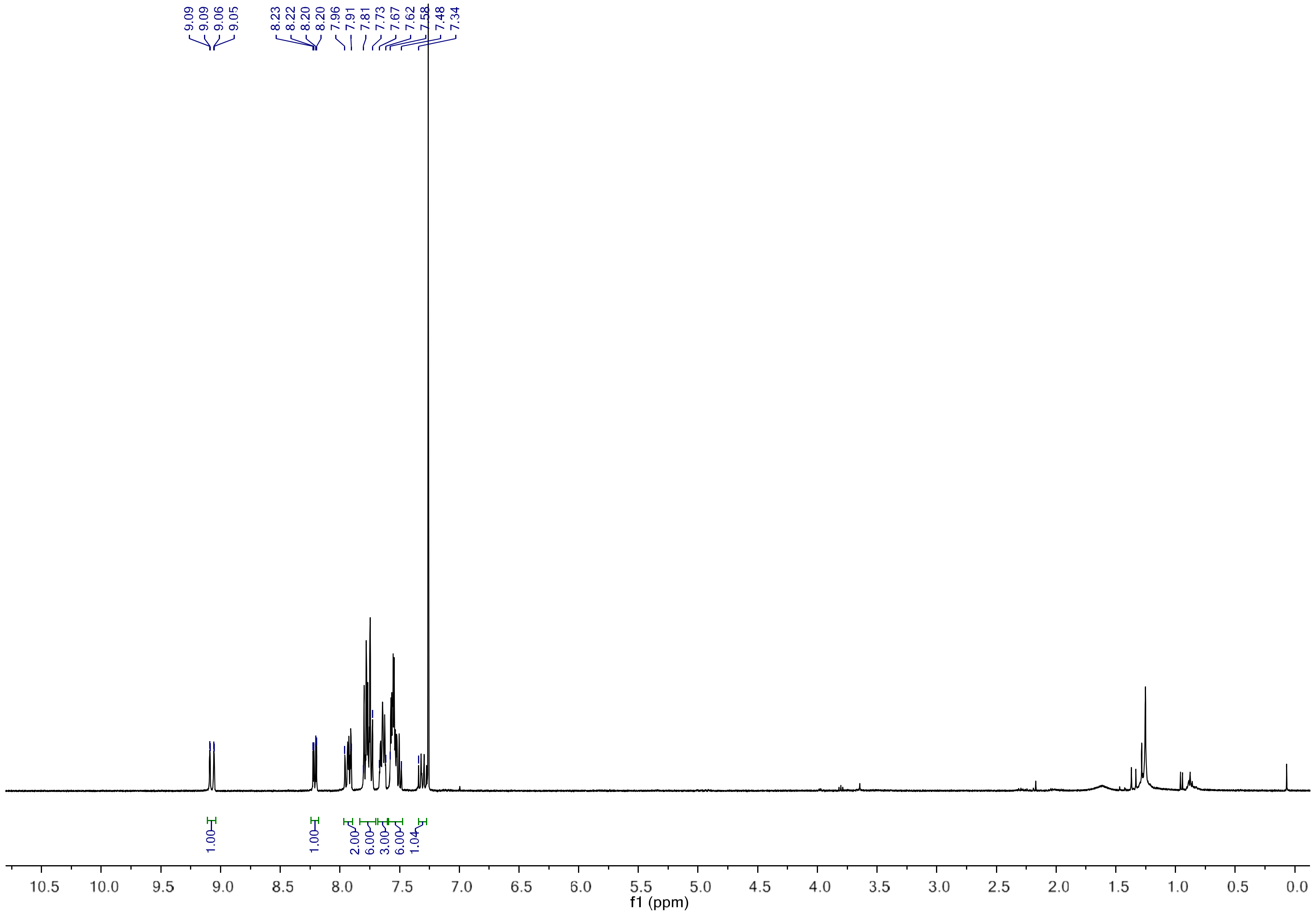

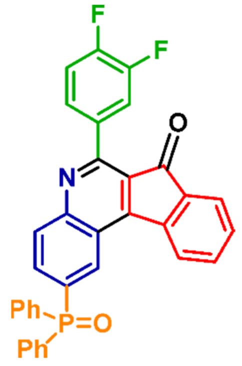

10e

^{31}P-RMN

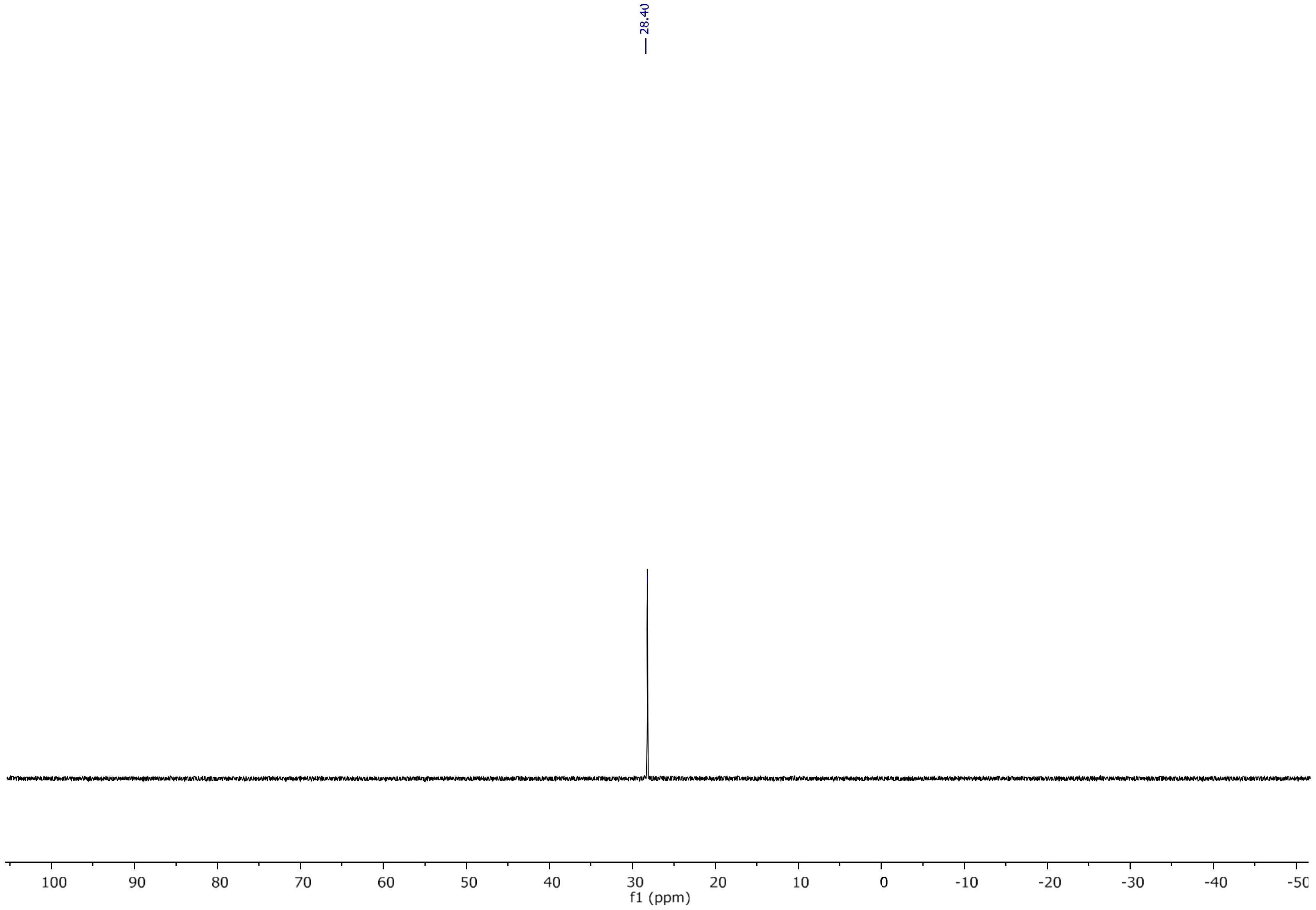
28.40
100 90 80 70 60 50 40 30 20 10 0 -10 -20 -30 -40 -50
f1 (ppm)

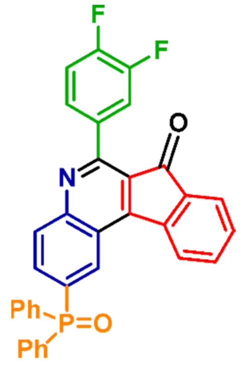
F
F
O
N
Ph
P=O
Ph

10e

^{19}F-RMN

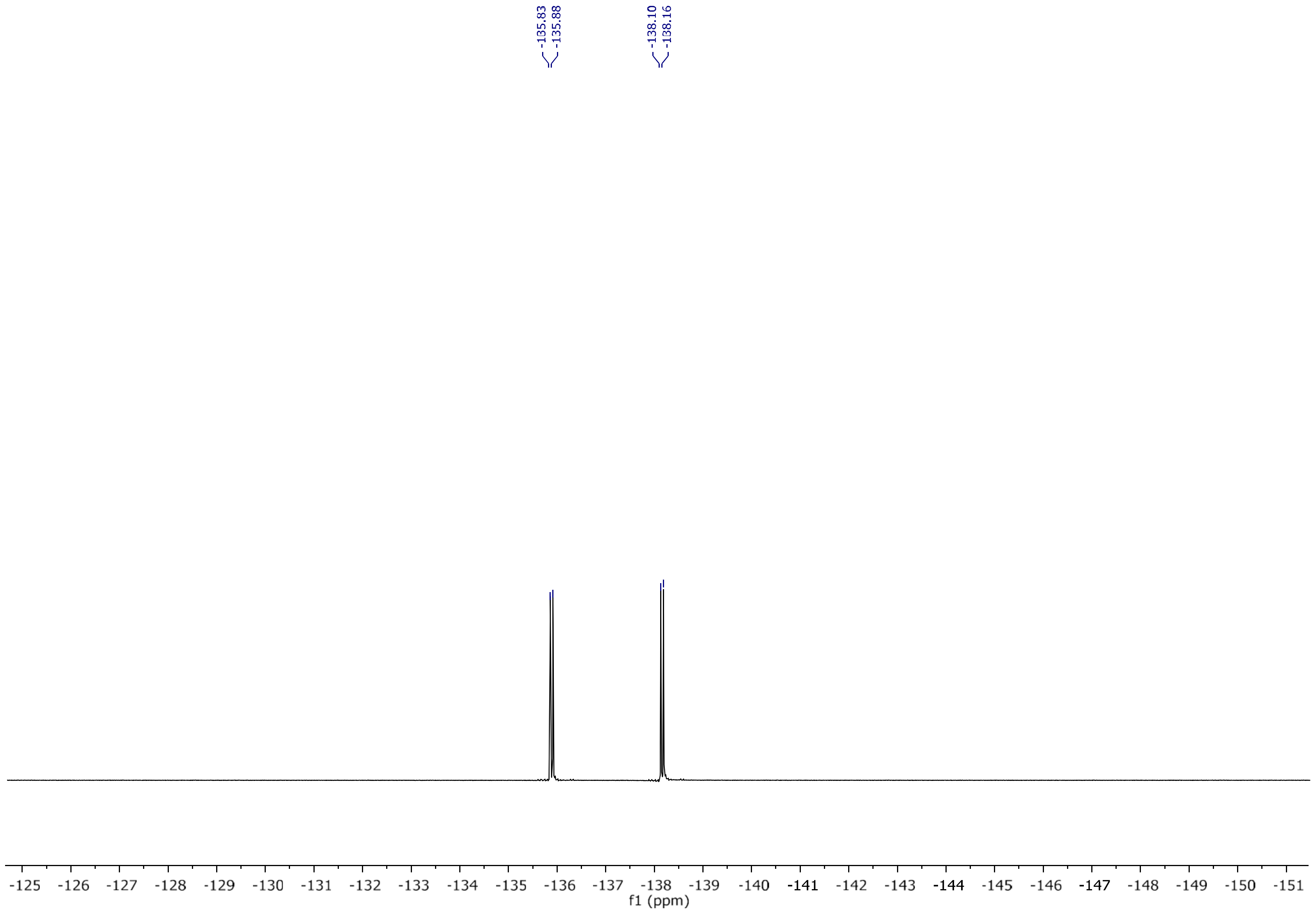

-135.83
-135.88
-138.10
-138.16
-125 -126 -127 -128 -129 -130 -131 -132 -133 -134 -135 -136 -137 -138 -139 -140 -141 -142 -143 -144 -145 -146 -147 -148 -149 -150 -151
f1 (ppm)

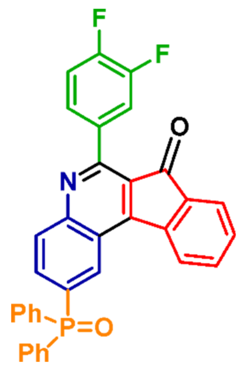

F
F
O
N
Ph
P=O
Ph

10e

^{13}C-RMN

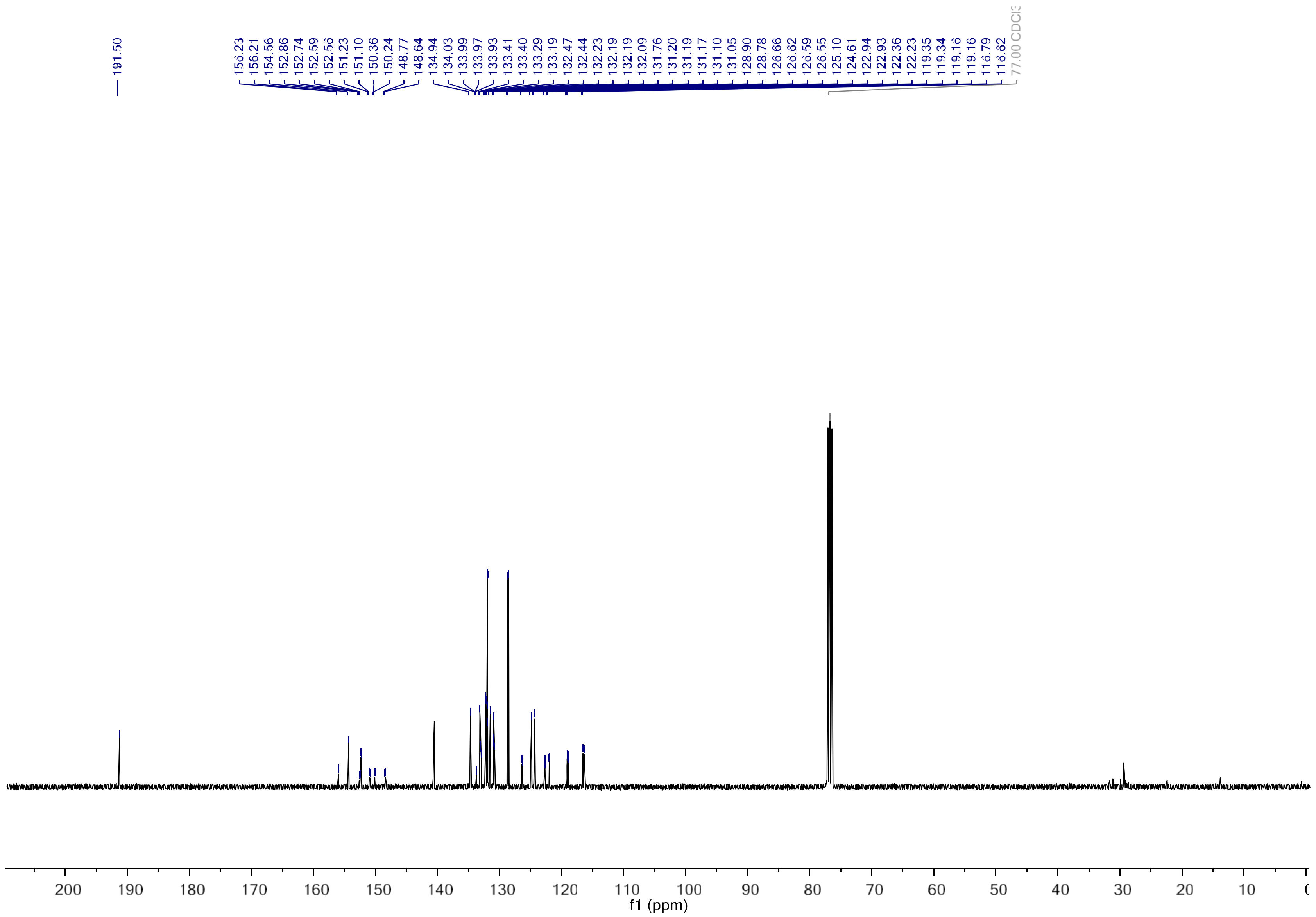

191.50
156.23
156.21
154.56
152.86
152.74
152.59
152.56
151.23
151.10
150.36
150.24
148.77
148.64
134.94
134.03
133.99
133.97
133.93
133.41
133.40
133.29
133.19
132.47
132.44
132.23
132.19
132.19
132.09
131.76
131.20
131.19
131.17
131.10
131.05
128.90
128.78
126.66
126.62
126.59
126.55
125.10
124.61
122.94
122.93
122.36
122.23
119.35
119.34
119.16
119.16
116.79
116.62
77.00 CDCl3
200 190 180 170 160 150 140 130 120 110 100 90 80 70 60 50 40 30 20 10 0
f1 (ppm)

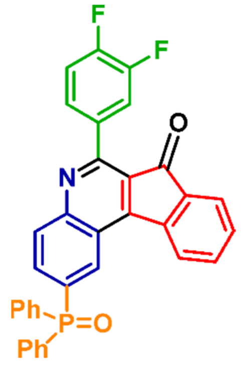

F
F
O
N
Ph
P=O
Ph

10e

^{1}H-RMN

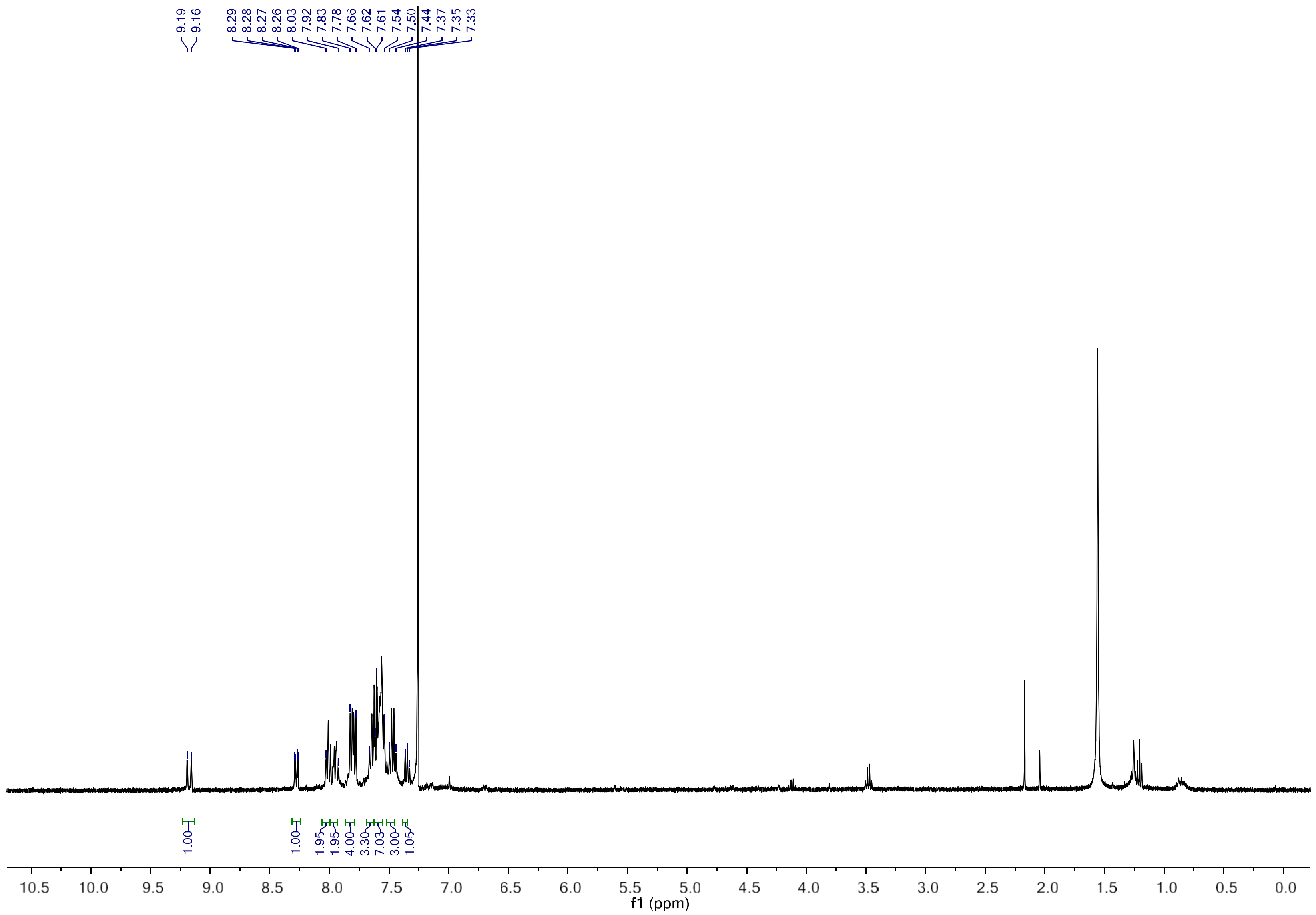

9.19
9.16
8.29
8.28
8.27
8.26
8.03
7.92
7.83
7.78
7.66
7.62
7.61
7.54
7.50
7.44
7.37
7.35
7.33
1.00
1.00
1.95
1.95
4.00
3.30
7.03
3.00
1.05
10.5 10.0 9.5 9.0 8.5 8.0 7.5 7.0 6.5 6.0 5.5 5.0 4.5 4.0 3.5 3.0 2.5 2.0 1.5 1.0 0.5 0.0
f1 (ppm)

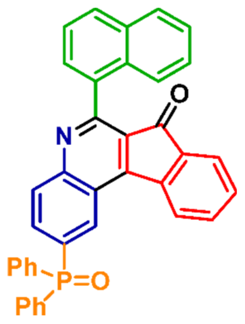

O
N
Ph
P=O
Ph

10g

^{31}P-RMN

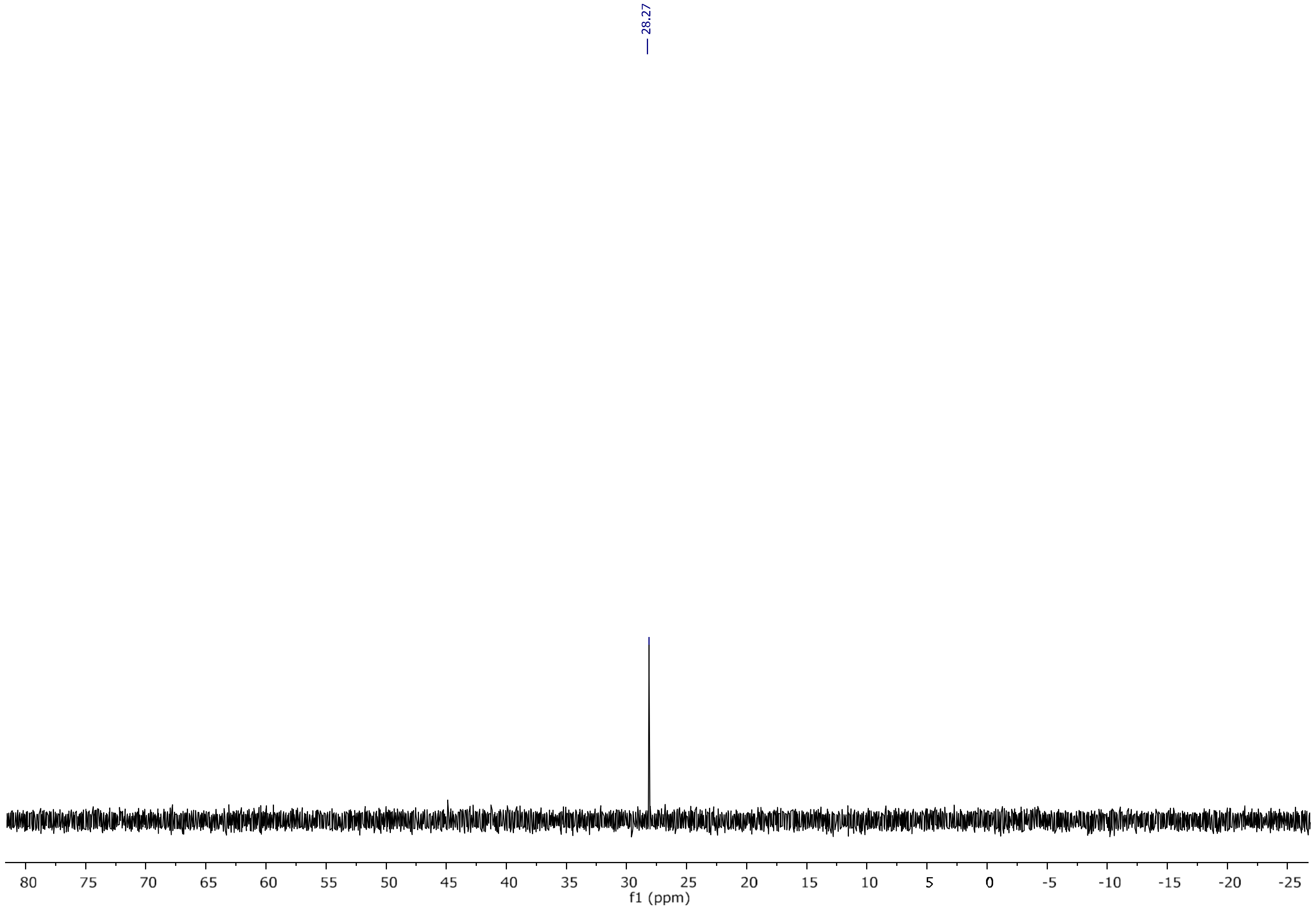
28.27
80 75 70 65 60 55 50 45 40 35 30 25 20 15 10 5 0 -5 -10 -15 -20 -25
f1 (ppm)

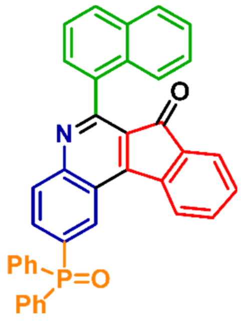
O
N
Ph
P=O
Ph

10g

^{13}C-RMN

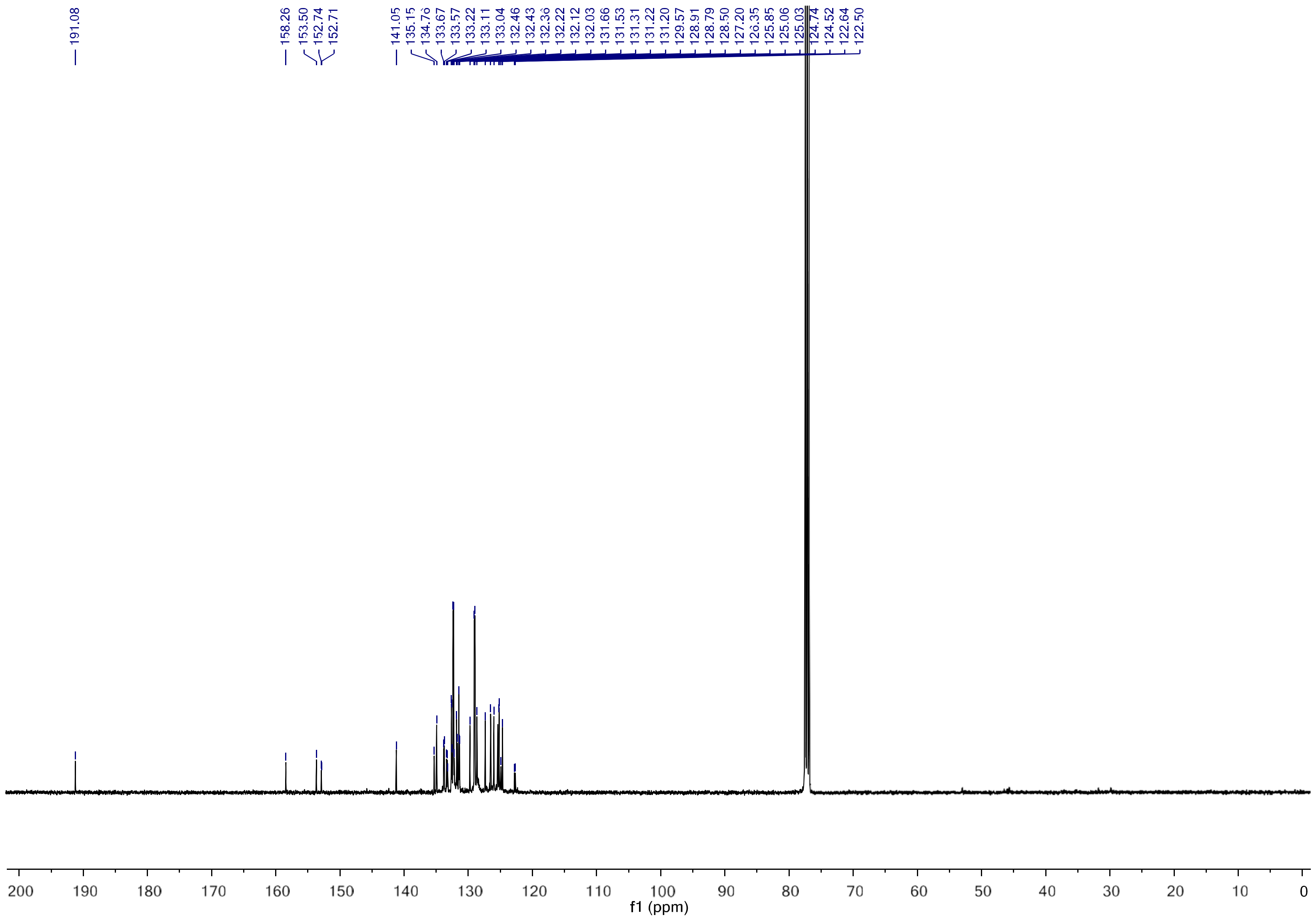

191.08
158.26
153.50
152.74
152.71
141.05
135.15
134.76
133.67
133.57
133.22
133.11
133.04
132.46
132.43
132.36
132.22
132.12
132.03
131.66
131.53
131.31
131.22
131.20
129.57
128.91
128.79
128.50
127.20
126.35
125.85
125.06
125.03
124.74
124.52
122.64
122.50
200 190 180 170 160 150 140 130 120 110 100 90 80 70 60 50 40 30 20 10 0
f1 (ppm)

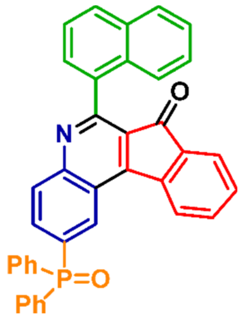

N
O
Ph
P=O
Ph

10g
